# Library Preparation and Sequencing Platform Introduce Bias in Metagenomic-Based Characterizations of Microbiomes

**DOI:** 10.1128/spectrum.00090-22

**Published:** 2022-03-15

**Authors:** Casper S. Poulsen, Claus T. Ekstrøm, Frank M. Aarestrup, Sünje J. Pamp

**Affiliations:** a Research Group for Genomic Epidemiology, National Food Institute, Technical University of Denmark, Kongens Lyngby, Denmark; b Section of Biostatistics, Department of Public Health, University of Copenhagen, Copenhagen, Denmark; c Novo Nordisk Foundation Center for Biosustainability, Technical University of Denmark, Kongens Lyngby, Denmark; Lerner Research Institute

**Keywords:** microbial communities, metagenomics, library preparation, DNA sequencing, microbiome, metadata

## Abstract

Metagenomics is increasingly used to describe microbial communities in biological specimens. Ideally, the steps involved in the processing of the biological specimens should not change the microbiome composition in a way that it could lead to false interpretations of inferred microbial community composition. Common steps in sample preparation include sample collection, storage, DNA isolation, library preparation, and DNA sequencing. Here, we assess the effect of three library preparation kits and two DNA sequencing platforms. Of the library preparation kits, one involved a PCR step (Nextera), and two were PCR free (NEXTflex and KAPA). We sequenced the libraries on Illumina HiSeq and NextSeq platforms. As example microbiomes, two pig fecal samples and two sewage samples of which aliquots were stored at different storage conditions (immediate processing and storage at −80°C) were assessed. All DNA isolations were performed in duplicate, totaling 80 samples, excluding controls. We found that both library preparation and sequencing platform had systematic effects on the inferred microbial community composition. The different sequencing platforms introduced more variation than library preparation and freezing the samples. The results highlight that all sample processing steps need to be considered when comparing studies. Standardization of sample processing is key to generating comparable data within a study, and comparisons of differently generated data, such as in a meta-analysis, should be performed cautiously.

**IMPORTANCE** Previous research has reported effects of sample storage conditions and DNA isolation procedures on metagenomics-based microbiome composition; however, the effect of library preparation and DNA sequencing in metagenomics has not been thoroughly assessed. Here, we provide evidence that library preparation and sequencing platform introduce systematic biases in the metagenomic-based characterization of microbial communities. These findings suggest that library preparation and sequencing are important parameters to keep consistent when aiming to detect small changes in microbiome community structure. Overall, we recommend that all samples in a microbiome study are processed in the same way to limit unwanted variations that could lead to false conclusions. Furthermore, if we are to obtain a more holistic insight from microbiome data generated around the world, we will need to provide more detailed sample metadata, including information about the different sample processing procedures, together with the DNA sequencing data at the public repositories.

## INTRODUCTION

Microbes are omnipresent and inhabit even the most extreme environments on Earth. Metagenomics-based analyses have provided unprecedented insight into these microbial communities. Metagenomics is applied heavily to human microbiomes, as well as animal and environmental microbiomes, and is being implemented to understand disease states (1–4), for diagnostic purposes (5), and for surveillance of pathogens and antimicrobial resistance (6–9). The data from such studies are a growing resource that can be utilized in meta-analysis and data mining, revolutionizing medicine, agriculture, and food production (6, 9–12).

Findings from microbiome studies can be difficult to replicate as observed in different meta-analyses of 16S rRNA gene amplicon studies (13–16). Considering the large number of features (including functional and taxonomic) under investigation in metagenomics, it is not surprising that studies do not seem to lack significant results (17). Data dredging is a real concern in metagenomics, which brings to mind the “replication crisis” that has been highlighted in the field of psychology (18, 19). Due to the challenges of replicating results, one must not overemphasize the results from exploratory research and keep in mind that there is a need to continually validate the robustness and ability to replicate results in microbiome studies (20, 21). With the improvement of genome reference databases and bioinformatics tools, the validation is an ongoing process (22–25).

Technical variation due to sample processing is an important factor that researchers have to minimize to make proper inferences in metagenomics studies. For example, the DNA isolation procedure has been shown to impact microbiome composition (26–28). The effect of library preparation and sequencing platform has been investigated in metagenomics primarily on human fecal samples. Library preparation was found to affect taxonomic and functional characterization of human fecal samples and *in silico*-constructed mock communities (21, 29). In a study by Costea et al. (26), the effect of library preparation was found to be lower than DNA isolation and intra- and intersample variation in general. The choice of sequencing platform also appears to have an effect on the characterization of microbiomes (30).

The aim of the present study was to assess the effect of library preparation (Nextera, KAPA PCR free, NEXTflex PCR-Free) and sequencing platform (Illumina HiSeq and NextSeq) on the metagenomics-based inference on DNA samples from two different pig feces and two different sewage microbiomes from a previous study (31). We show that library preparation and sequencing platform introduce systematic bias in the inferred microbial community composition for both sample types and that this effect is important when comparing similar samples, such as pig feces, in the present study. This highlights the need for consistent sample processing and demonstration of caution when comparing data from different studies.

## RESULTS

**FIG 1:**
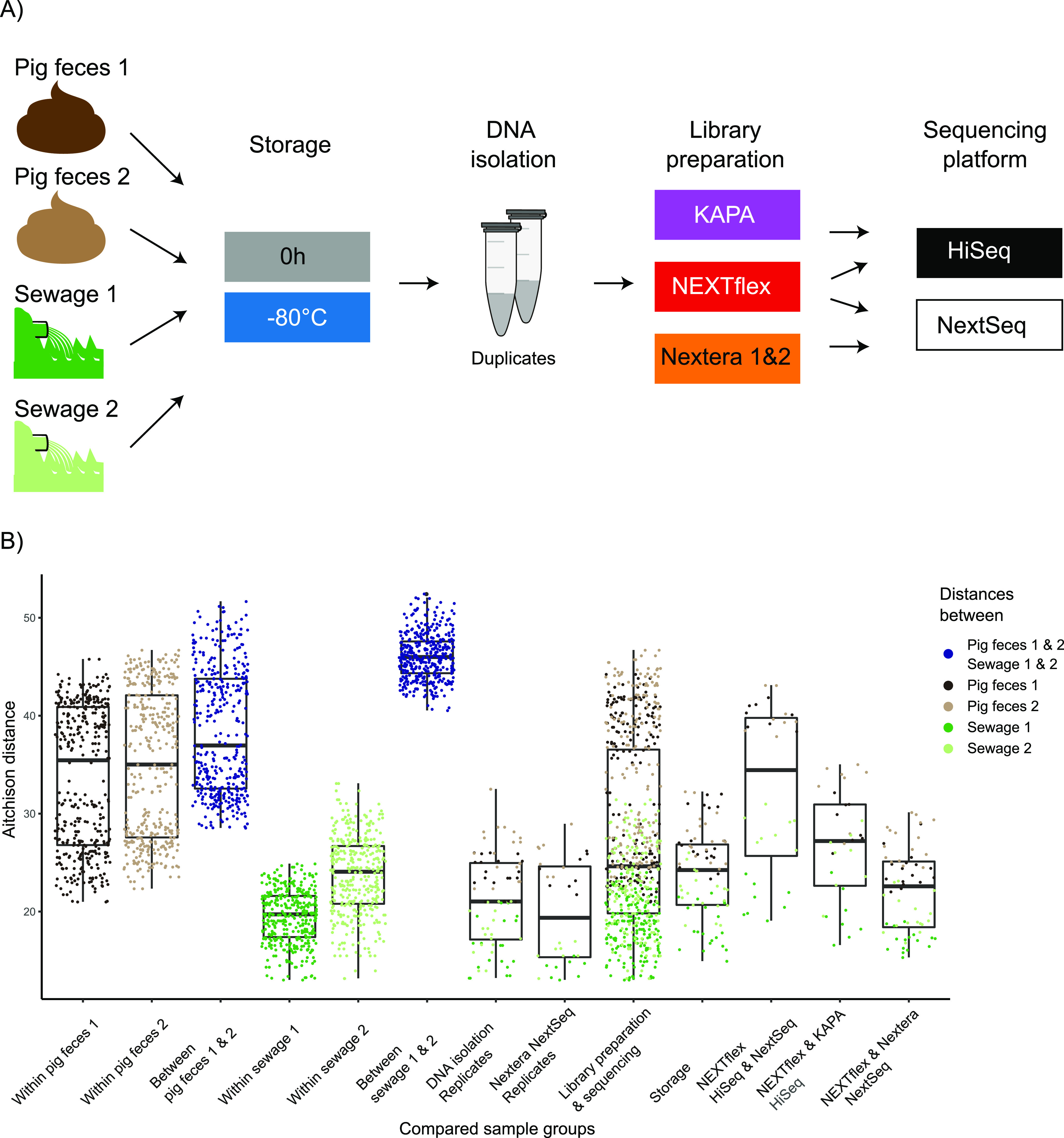
Study design and comparison between sample groups. (A) Two pig feces samples and two sewage samples were processed directly or after storage at −80°C for 64 h. The DNA isolation was performed in duplicates, respectively. Library preparation and sequencing were performed in four different combinations, NEXTflex PCR-Free library preparation with sequencing on a HiSeq (NEXTflex HiSeq), KAPA PCR-free library preparation with sequencing on a HiSeq (KAPA HiSeq), NEXTflex PCR-Free library preparation with sequencing on a NextSeq (NEXTflex NextSeq), and Nextera library preparation with sequencing on a NextSeq (Nextera NextSeq). The latter sequencing strategy was performed twice (Nextera 1 NextSeq and Nextera 2 NextSeq). The setup resulted in a total of 80 metagenomes plus 5 negative controls (i.e., DNA extraction controls). (B) Boxplots display pairwise Aitchison distances between different groupings of samples. Within the different groups, dots representing the distances were colored according to which sample the comparison was made in. Blue dots represent a distance between two different samples.

A subset of DNA samples was selected from a large-scale study (31) to assess the effect of library preparation and DNA sequencing on inferred microbiome composition based on metagenomics. The DNA samples originated from two pig fecal samples (pig feces 1 and pig feces 2) and two sewage samples (sewage 1 and sewage 2). For the present study, we selected DNA aliquots from fecal and sewage samples that originally were processed immediately (time point 0) and were subjected to storage at 80°C, respectively, to not only assess whether one can distinguish different samples but also samples that have the same origin but exhibit differences due to different handling conditions. The DNA aliquots underwent a total of four different strategies for library preparation and DNA sequencing (Fig. 1A), namely, KAPA PCR-free library preparation with sequencing on a HiSeq (KAPA HiSeq), NEXTflex PCR-Free library preparation with sequencing on a HiSeq (NEXTflex HiSeq), NEXTflex PCR-Free library preparation with sequencing on a NextSeq (NEXTflex NextSeq), and Nextera library preparation with sequencing on a NextSeq (Nextera NextSeq). The latter sequencing strategy was performed twice (Nextera 1 NextSeq and Nextera 2 NextSeq).

### Quality control of sequencing output.

The number of raw reads from the different library preparations and sequencing platforms was similar with about a factor of 2 difference when comparing the medians. The highest number of reads was obtained from the NEXTflex HiSeq run (median, 12.1; range, 6.3 to 30.8 million reads) and the lowest from the NEXTflex NextSeq run (median, 7.6, range; 2.7 to 9.4 million reads) (Table S1 in the supplemental material). The outputs from the KAPA HiSeq run (median, 9.4; range, 7.8 to 17.4 million reads) and the Nextera NextSeq runs (median, 10.2; range, 6.5 to 16.5 million reads) were about the same. More reads were obtained from the pig fecal samples than the sewage, but a larger proportion of the sewage reads mapped to the reference databases. The microbial community of the sewage samples exhibited a higher Simpson diversity than the pig feces (Table S1). The number of mapped reads was higher for the sewage samples, and many of the samples had reached a plateau as observed when drawing a rarefaction curve (Fig. S1). Similar results were obtained when comparing the mean of the percentage of unmapped reads of the same sample across the different library preparation and sequencing platform runs (pig feces 1, 87.4 to 88.4; pig feces 2, 89.7 to 90.5; sewage 1, 70.1 to 74.1; sewage 2, 54.2 to 59.3) (Table S1).

### Sample processing impacts on inferred microbiome structure.

Considering the compositionality of data, the taxonomy table was centered log ratio (CLR) transformed before calculating Euclidean distances to have data not confined to the simplex. The resulting pairwise Aitchison distances (corresponding to Euclidean distances between CLR-transformed compositions) were calculated between all the samples and visualized using principal-component analysis (PCA) (Fig. S2A). The sample type explained the greatest variance, and pig feces and sewage samples were clearly separated on the first axis. A clear separation of the two sewage samples was observed on the second axis, while the two pig fecal samples clustered together. Ordination of the pig feces and sewage samples separately revealed that it was possible to differentiate the two pig fecal samples (Fig. S2B). However, there were also two clusters within each pig fecal sample. A clear separation of the two sewage samples was still observed (Fig. S2C). Also in a boxplot visualization, library preparation, sequencing platform, and storage condition did not hamper the ability to differentiate between the two sewage samples (Fig. 1B). However, we observed an overlap between pig feces 1 and 2 comparisons relative to comparing within the two samples, representing the effect of the different sample processing parameters. Nevertheless, the median suggested there is a difference between pig feces 1 and 2 (Fig. 1B). In general, larger distances were calculated for the comparisons of sample processing parameters in pig fecal samples than sewage. The shortest distances were observed when comparing the DNA isolation replicates and the replicates of the Nextera NextSeq runs, respectively. The distances between samples that differed in library preparation and sequencing platform were greater than samples that differed in storage conditions (i.e., whether they were processed directly or after freezing at −80°C for 64 h). The sequencing platform appeared to be a major contributor of variation when comparing the samples that were prepared with NEXTflex and sequenced on both an Illumina HiSeq and an Illumina NextSeq platform, respectively (Fig. 1B, third box from the right), whereas using two different preparation kits, i.e., NEXTflex and KAPA sequenced on an Illumina HiSeq, introduced a relatively lower variation (Fig. 1B, second box from the right). The differences were observed to be even lower when samples were prepared with the two library preparation kits, NEXTflex and Nextera, and sequenced on an Illumina NextSeq (Fig. 1B, first box from the right).

**FIG 2:**
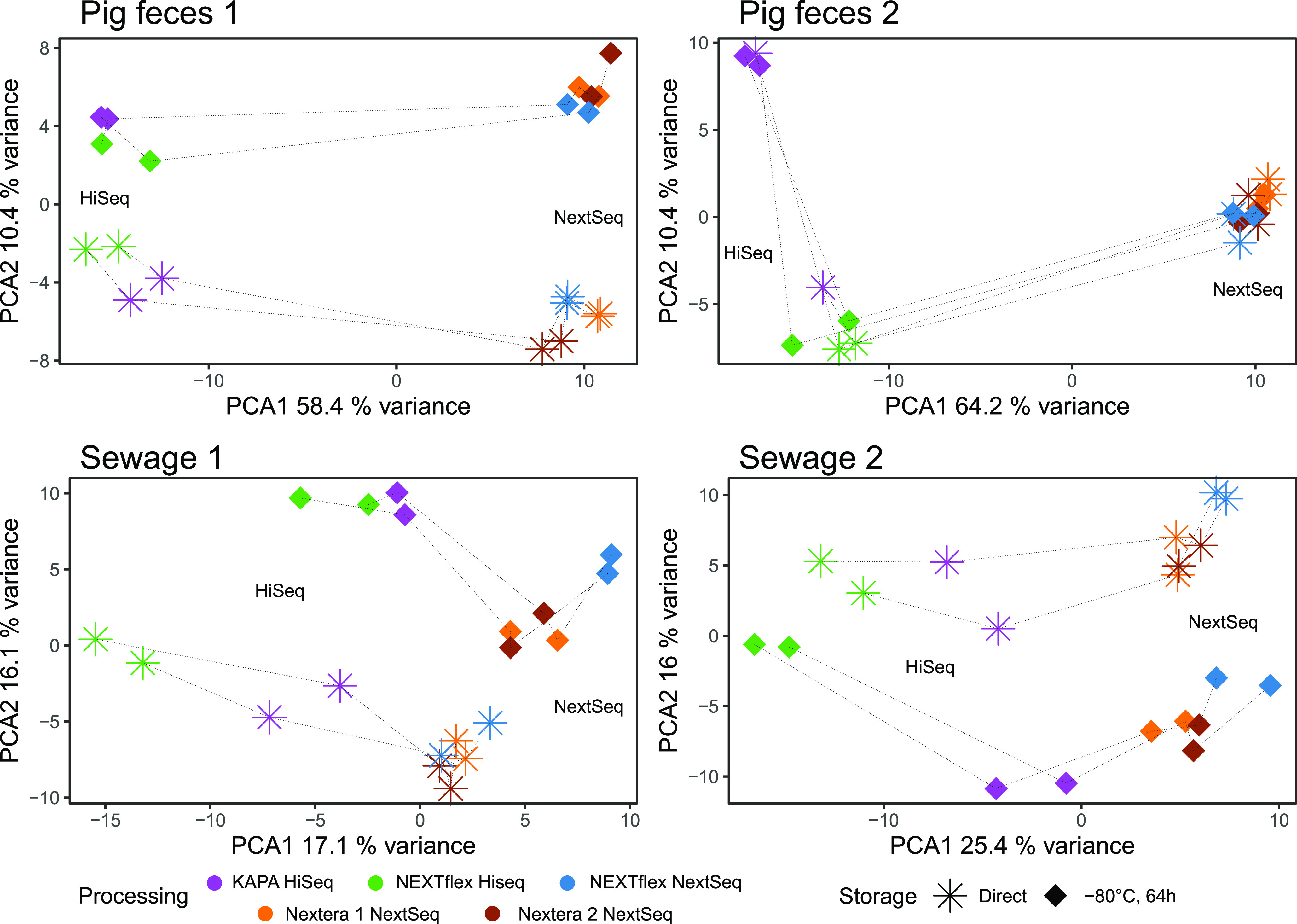
Principal-component analysis (PCA) subset to the different sample matrices. Euclidean distances were calculated after performing centered log-ratio transformation (CLR) of the count data (Aitchison distances). Variance explained by the two first axes are included in their labels. The same DNA samples processed differently are connected with dotted lines.

To investigate the effect of sample processing further, PCAs were performed for the individual samples (pig feces 1, pig feces 2, sewage 1, and sewage 2). Similar patterns were observed in all samples, indicating that there was a systematic effect from sequencing platform, library preparation, and storage condition (Fig. 2). The samples clustered primarily according to sequencing platform and library preparation along the *x* axis that represents most of the variation. On the *y* axis, samples clustered according to storage condition. In general, the DNA isolation replicates were similar, as well as the two Nextera NextSeq runs (Fig. 2). All the parameters had a significant effect based on permutational multivariate analysis of variance (PERMANOVA) except for storage when comparing all of the samples and in pig feces 2 (Table 1). The percent variations in pig feces attributed to sample (pig feces 1 and pig feces 2) (21.1%) and sequencing platform (19.1%) were at similar levels, further emphasizing the importance of sample processing when comparing communities that are more similar to each other (Table 1).

**TABLE 1:** Effect of sample origin (pig feces 1, pig feces 2, sewage 1, and sewage 2) and different parameters in sample processing (library preparation, DNA sequencing)^*a*^

Sample(s) included	Sample *P* value (%)^*b*^	Storage *P* value (%)^*b*^	Library prepn *P* value (%)^*b*^	Sequencing platform *P* value (%)^*b*^
All	<10^−5^ (81.9)	6.4 × 10^−2^ (0.5)	4.2 × 10^−2^ (1.0)	3.0 × 10^−4^ (1.8)
Pig feces	<10^−5^ (21.1)	3.8 × 10^−3^ (3.3)	5.7 × 10^−4^ (6.2)	<10^−5^ (19.1)
Sewage	<10^−5^ (61.7)	2.5 × 10^−2^ (2.9)	3.0 × 10^−2^ (4.1)	4.4 × 10^−3^ (4.5)
Pig feces 1	NA^*c*^	2.8 × 10^−3^ (9.7)	2.8 × 10^−2^ (8.9)	<10^−5^ (26.2)
Pig feces 2	NA	0.17 (2.7)	5.4 × 10^−3^ (12.3)	<10^−5^ (25.3)
Sewage 1	NA	<10^−5^ (15.1)	3.6 × 10^−4^ (14.4)	<10^−5^ (12.8)
Sewage 2	NA	<10^−5^ (14.0)	6.0 × 10^−5^ (17.8)	<10^−5^ (19.6)

a Statistical tests were performed by multiple permutations partitioning sum of squares (PERMANOVA). The *P* value, as well as the percentage of variation explained by the parameters, is reported, testing different sample sets (all, pig feces, sewage, pig feces 1, pig feces 2, sewage 1, and sewage 2).

b Proportion of the variation explained in the PERMANOVA.

c NA, not applicable; no *P* value obtained when variable subset to a single sample (pig feces 1, pig feces 2, sewage 1, and sewage 2).

### Sample processing impacts on inferred microbial abundances.

**FIG 3:**
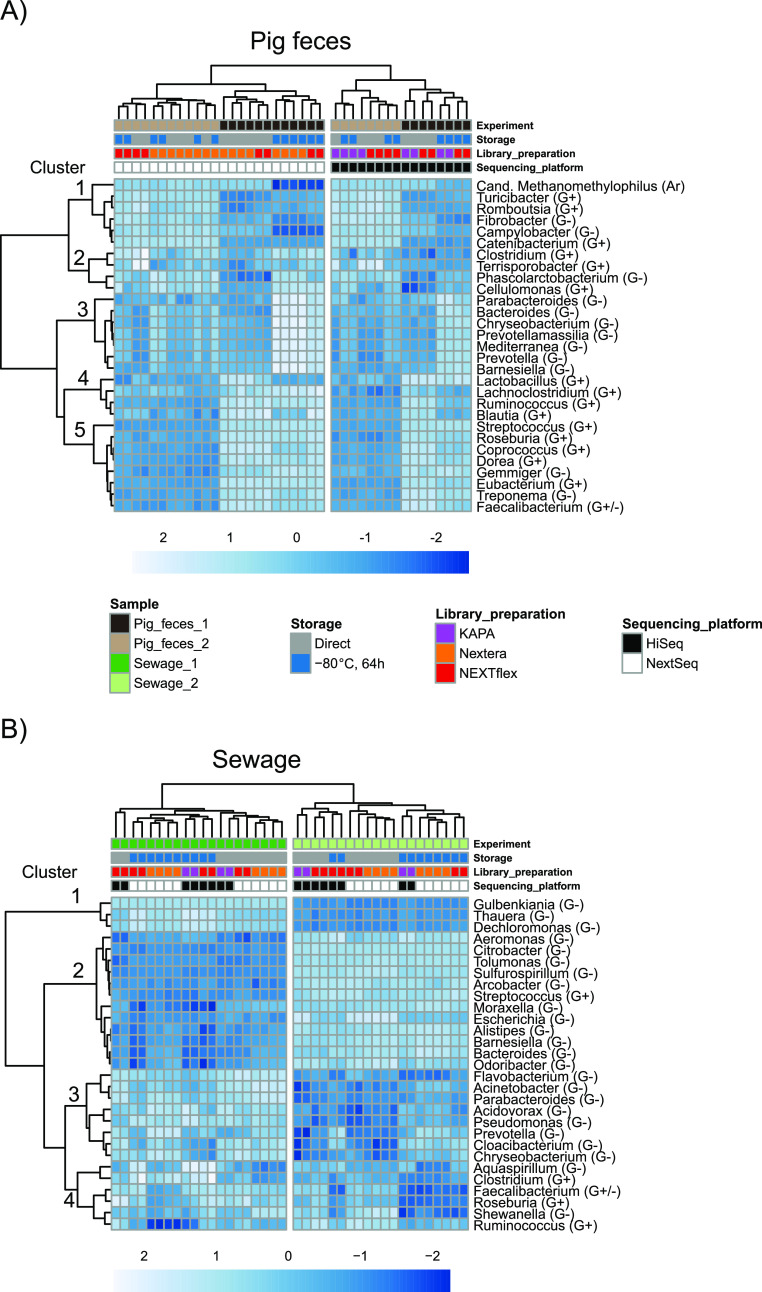
Heatmaps of pig feces and sewage samples separately with the 30 most abundant genera. Complete-linkage clustering was performed to create dendrograms for both genera and samples. Spearman correlation was used to cluster the genera, and Aitchison distances were used to cluster the samples. Genera abundance depicted in the cells were CLR-transformed counts standardized to zero mean and unit variance. Grouping of organisms were included in genera names according to cell wall structure based on Gram-positive staining (G+), Gram-negative staining (G−), or belonging to *Archaea* (Ar). (A) Heatmap of all pig feces samples, where the first branching was according to sequencing platform. The third cluster of genera exclusively contained Gram negatives. (B) Heatmap of all sewage samples. The fourth cluster mainly consisted of Gram positives. A few Gram positives were also present in the other clusters. For explanation of colours, see panel A.

To investigate the effect of library preparation and sequencing platform on the abundance of specific microorganisms, an overview of the 30 most abundant genera was visualized in heatmaps (Fig. 3). For the pig samples, the aliquots appeared to cluster mainly based on the sequencing platform (NextSeq versus HiSeq) (Fig. 3A). In contrast, it was possible to distinguish the two sewage samples, which clustered according to sample origin (sewage 1 versus sewage 2) (Fig. 3B). A clustering of samples was also observed to a certain degree for both pig feces and sewage according to storage condition and library preparation.

The pig feces contained both Gram-negative and Gram-positive bacteria, and cluster 3 exclusively consisted of Gram negatives. There were a few Gram negatives in the other clusters, indicating that sample processing shifts the abundance profiles for specific groups of organisms; in this case, it appeared to be associated with cell wall structure (Fig. 3A). A similar pattern was observed for sewage that mainly consisted of Gram negatives. The majority of Gram positives were part of cluster 4, including *Clostridium*, *Faecalibacterium*, *Roseburia*, and *Ruminococcus*. However, this cluster also contained Gram-negative genera (Fig. 3B).

One explanation for the community differences observed by sample processing could be a possible contamination during the library preparation and sequencing steps. To elucidate this, sparse partial least-squares discriminant analysis (sPLS-DA) was performed, assessing which genera best characterize the library preparation and sequencing platform processing methods. Components 1, 2, and 3 were included in the model containing 5, 50, and 20 different genera, respectively (Fig. S3). The majority of microorganisms were abundant organisms observed across all of the sample processing methods. However, a few were clear indicators of contamination during library preparation and sequencing and were mainly present in a single processing method. This included *Methylobacterium* in the KAPA HiSeq run and *Cutibacterium* in the second Nextera NextSeq run, bacteria that previously have been associated with kit contamination (32). A heatmap of the 30 most abundant genera in the blank controls additionally revealed a high abundance of *Ralstonia* in the Nextera NextSeq runs that were performed with the same kit reagents (Fig. S4). Overall, the organisms associated with contamination were limited. The separation of the samples according to the different processing parameters therefore appeared to be real changes to the relative abundances between organisms inherently present in the microbiomes and not due to contamination.

A constrained ordination analysis (here, redundancy analysis [rda]), also subset according to whether samples were processed directly or after freezing, was performed to assess whether groups of organisms at a taxonomically higher level were associated with a specific library preparation and sequencing method. In the pig feces, *Proteobacteria* seemed associated with the HiSeq runs (Fig. S5). However, this was not observed for sewage. For sewage, *Archaea* were associated with the HiSeq runs, but also *Eukaryotes* consisting of fungi and *Cryptosporidium* seemed associated with the HiSeq runs in sewage 1 (Fig. S5). Overall, it was difficult to observe a pattern when assessing this grouping of genera, highlighting that it might be difficult to generalize the effect of sample processing in different sample types and different samples of the same type.

## DISCUSSION

With the increasing amount of metagenomic data in public repositories, meta-analysis and pooling of data from different studies are exciting new opportunities to gain further insight into the microbial world (10–12, 24, 33). Data generation is usually not performed with a standard procedure across studies, and sample processing is an important factor to be aware of when trying to make inferences in these cross-study investigations (21, 26). In the present study, both library preparation and sequencing platform had a significant effect on explaining the variance in the data (Table 1). That these parameters affect the community description has also been observed previously (21, 29, 30). In the study by Costea et al. (26), DNA isolation had the largest effect compared with other technical variations. In the present study, DNA isolation was performed centrally by the same person, while library preparation and sequencing were performed in-house or at external providers, but not in any of the cases by the same person, possibly increasing variation due to DNA shipping and handling in this specific step. When performing a validation study assessing the technical variation of sample processing, the large number of methodologies and variations thereof make it impossible to test all parameters. It is likely that selecting methods that are based on different principles and for specific purposes yield results that highlight the importance of this specific step. Jones et al. (21) investigated the effect of library preparation and observed that members of a mock microbial community became skewed depending on the library preparation kit. While they noted that each method had advantages and disadvantages, they recommended using a PCR-free library preparation approach, as it, for example, reduces PCR bias. Bowers et al. (29) investigated community changes using different amounts of input DNA and observed that this modification had a significant effect on community description. This effect can increase bias associated with library preparation and sequencing platform in other studies where starting material is of variable quality. In the present study, investigation of sequencing platforms was limited to the NextSeq and HiSeq, which are both Illumina platforms resembling each other in technology and which were selected due to their popularity in metagenomics with low cost relative to output (34). However, the platforms have been reported to exhibit differences in index hopping (35). In the present study, a large effect was attributed to the sequencing platform, and that was also observed when using the same library preparation kit (NEXTflex PCR-Free) (Fig. 1B). The library preparation included two methods that required prefragmented DNA that was prepared PCR-free (KAPA and NEXTflex). It was decided to include the Illumina Nextera library preparation as well to compare with a technique that does not resemble the others in having enzymatic fragmentation and which involved a PCR step that is commonly applied when not enough DNA is available to prepare DNA for sequencing PCR free. However, the two Nextera runs were relatively similar to the NEXTflex run when sequenced on the NextSeq (Fig. 2). The present study was not a full factorial experiment, and this should be emphasized when comparing the effect sizes of specific processing parameters.

One explanation for the differences observed between the processing runs can be contamination bias. When designing a metagenomics study, it is, to some extent, possible to remove kit contaminations or carryover between sequencing runs from the data *in silico*, if, for instance, blank controls are included or by rotating indexing primers between adjacent runs, respectively (36). In the present study, comparing the sPLS-DA results with the blank controls rarely identified the same genera, indicating that the genera reported to explain the specific sample processing the most were not due to contamination during DNA extraction. The general variation associated with redoing the library preparation and sequencing was low when comparing the two Nextera sequencing runs (Fig. 1 and 2). The differences observed are therefore most likely due to true variation associated with the sample processing. Furthermore, it was possible to detect that these patterns were systematic in the different samples (Fig. 2) and that this could be partly explained with some crude features such as distinguishing between Gram-negative and Gram-positive bacteria or at a higher taxonomic classification (Fig. 3 and Fig. S5 in the supplemental material). The grouping of genera into Gram negative and Gram positive might be confounders of an underlying explanation that could be associated with DNA characteristics such as guanine-cytosine percent (GC%) or other specific DNA patterns. Another possibility is that DNA fragmentation during sampling, storage, and DNA isolation provided DNA of different quality for specific organism groups. A shift in community structure is then reflected in the selection of different fragment sizes during the library preparation and sequencing. Practical limitations were also an issue when designing the study. To reduce the bias associated with DNA extraction, the QIAamp Fast DNA stool minikits were all ordered together, ensuring that kits were from the same manufacturing batch. Another possible bias might arise from DNA samples that were frozen in between processing them for sequencing. However, only small changes were observed between the two Nextera NextSeq runs.

The Aitchison distances obtained from comparing within the two pig fecal samples separately relative to within the two sewage samples also revealed that storage, library preparation, and sequencing platform has a larger effect in pig feces (Fig. 1B). Since the distances between the two pig fecal samples were smaller than the distances between the two sewage samples, it was difficult to discern the two pig fecal samples when samples were processed differently (Fig. 3). It is concerning that the variation due to sample processing might hamper the ability to differentiate between two different pig fecal samples, and this might obstruct the ability to draw meaningful conclusions when technical variations cannot be distinguished from true changes. These results should, on the other hand, not be overstated; the two pig fecal samples were obtained from an in-bred race raised under very similar conditions, including feeding, and even though they were obtained from two different healthy pigs at two different farms, the two communities are relatively similar. The finding highlights that the importance of technical variation depends on the differences that one is trying to detect (16). The technical variation did not hamper the ability to differentiate between the two sewage samples.

We show that library preparation and sequencing platform introduce systematic bias in the metagenomic-based characterization of microbial communities. These findings suggest that library preparation and sequencing are important parameters to keep consistent when aiming to detect small changes in community structure. In the present study, the bias was somewhat dependent on sample type, highlighting the importance of assessing the effect of sample processing in the specific sample type under investigation.

## MATERIALS AND METHODS

### Sample processing.

A subset of 85 DNA samples was selected from a large-scale study examining the effect of sample storage conditions on inferred microbiome composition (31). The DNA samples originated from two pig fecal samples (pig feces 1 and pig feces 2) and two sewage samples (sewage 1 and sewage 2). The two pig fecal samples were collected on different occasions from different conventional pig production farms near the laboratory. The two sewage samples were collected at a local wastewater treatment facility on different occasions. DNA isolation was performed in duplicate with a modified QIAamp Fast DNA stool minikit (Qiagen) protocol, including an initial bead-beating step (Mo Bio garnet beads) (27) (Fig. 1B). A DNA extraction (blank) control was included at each time of DNA isolation (5 controls as part of the present study). For a detailed list of all samples included in this study, see Table S1 (column H, “Other study [library preparation]”) in Poulsen et al. (31). The concentration of DNA samples was measured with the Qubit double-stranded DNA (dsDNA) high-sensitivity (HS) assay kit on a Qubit 2.0 fluorometer (Invitrogen, Carlsbad, CA) before storing the DNA at −20°C.

### Library preparation and sequencing.

Library preparation and sequencing were performed in the order described below, and the DNA was frozen between the sequencing runs:

### (i) KAPA PCR-free on a HiSeq.

DNA was shipped for sequencing to an external provider (Admera Health, NJ, USA). The DNA (500 ng) was fragmented mechanically (Covaris E220 evolution; aimed insert size, 350 bp) using ultrasonication. The KAPA library preparation was run PCR free according to the manufacturer’s recommendations (KAPA HyperPrep kit; catalog no. KR0961 v6.17). Sequencing was performed on an Illumina HiSeq 4000 (2 × 150 cycles, paired end).

### (ii) NEXTflex PCR-Free on a HiSeq.

DNA was shipped for sequencing to an external provider (Oklahoma Medical Research Foundation, OK, USA). The DNA (500 ng) was fragmented mechanically (Covaris E220evolution; aimed insert size, 350 bp) using ultrasonication. The NEXTflex library preparation was run PCR free according to the manufacturer’s recommendations (Bioo Scientific NEXTflex PCR-Free DNA sequencing kit; catalog no. 5142-01). Sequencing was performed on an Illumina HiSeq 4000 (2 × 150 cycles, paired end).

**(iii) NEXTflex PCR-Free on a NextSeq.** The DNA (500 ng) was fragmented with mechanical fragmentation (Covaris E210, aimed insert size, 350 bp, duty factor, 10%; intensity, 5; cycle burst, 200; treatment time, 240 s) using ultrasonication. The NEXTflex library preparation was run PCR free with NEXTflex barcodes (NEXTflex-96 DNA barcodes) according to the manufacturer’s recommendations (Bioo Scientific NEXTflex PCR-Free DNA sequencing kit; catalog no. 5142-01). Sequencing was performed in-house on an Illumina NextSeq 500 (midoutput v2, 2 × 150 cycles, paired end).

**(iv) Nextera 1 and 2 on a NextSeq.** The Nextera XT library preparation was performed twice. The Nextera XT protocol was carried out according to the manufacturer’s recommendations (Nextera XT DNA library prep kit; document no. 15031942v02). This included a tagmentation step that fragments the DNA (1 ng) and ligates adaptors and a PCR step amplifying DNA and adding indexing primers. Library cleanup was performed with AMPure XP beads and normalized before sequencing was performed in-house on an Illumina NextSeq 500 (midoutput v2, 2 × 150 cycles, paired end). The bioanalyzer results revealed that the aimed insert size of 350 bp was larger than expected (File S6 in the supplemental material).

### Bioinformatics and statistical analysis.

Preprocessing of raw reads included trimming (Phred quality score, 20) and removal of reads shorter than 50 bp (BBduk2) (37). Mapping was performed with a Burrows-Wheeler aligner (BWA-mem) as implemented in MGmapper (22). Mapping was performed in the default “best mode” to 11 databases, first filtering against the human database and then extracting the number of raw reads mapping to the genomes of bacteria, fungi, archaea, viruses, and *Cryptosporidium*. A read count correction was implemented to adjust large hit counts to specific contigs as implemented in Hendriksen et al. (9). All counts in the count table were divided by 2 to account for reads mapping as proper pairs and then aggregating to genus level. The raw reads are deposited in the European Nucleotide Archive (ENA) (BioProject accession no. PRJEB31650).

All statistical analyses adhered to the compositional data analysis framework and were performed in R version 3.5.2 (38–40). Alpha diversity was calculated based on the raw count table estimating richness (Chao1), evenness (Pielou’s), and diversity (Simpson) using the diversity function in vegan. Initial filtering of the count matrix was performed by removing all genera below an average count of 5. The estimation of zeroes was performed using simple multiplicative replacement (41). Centered log-ratio transformation (CLR), where the log of each feature is compared relative to the geometric mean, was used to enable real-space calculations (40, 42, 43). CLR was used in principal-component analysis (PCA), heatmaps to perform complete-linkage clustering analysis of the samples, boxplots to calculate pairwise Euclidean distances between samples (Aitchison distance), and permutational multivariate analysis of variance (PERMANOVA; a nonparametric multivariate statistical test, with 99,999 permutations assessing the marginal effects of the terms [44]), sparse partial least-squares discriminant analysis (sPLS-DA; a multivariate dimensionality-reduction tool, with 5-fold cross-validation repeated 10 times [38]), and constrained ordination with redundancy analysis (rda) (38, 40, 42, 45). Spearman correlation on CLR-transformed data was used to cluster the genera visualized in the heatmap, but the genera abundance depicted in the cells was standardized to zero mean and unit variance after CLR transformation for comparability. Analyses performed are included (File S7), and the code is available from https://github.com/csapou/LibraryPreparationandSequencingPlatform.

### Data availability.

The raw reads supporting the conclusions of this article are available at the European Nucleotide Archive (ENA) (BioProject accession no. PRJEB31650). The statistical analyses performed are provided in File S7. The statistical analysis is furthermore available at https://github.com/csapou/LibraryPreparationandSequencingPlatform.
