# Supplementary material for: Library Preparation and Sequencing Platform Introduce Bias in Metagenomic-Based Characterizations of Microbiomes
Source: Microbiol Spectr. 2022 Mar 15;10(2):e00090-22. doi: 10.1128/spectrum.00090-22 (PMC9045301; doi:10.1128/spectrum.00090-22)
Supplement: SUPPLEMENTAL FILE 1 — Supplemental material. Download SPECTRUM00090-22_Supp_1_seq7.pdf, PDF file, 6.5 MB [file spectrum00090-22_supp_1_seq7.pdf]

Supplemental Material

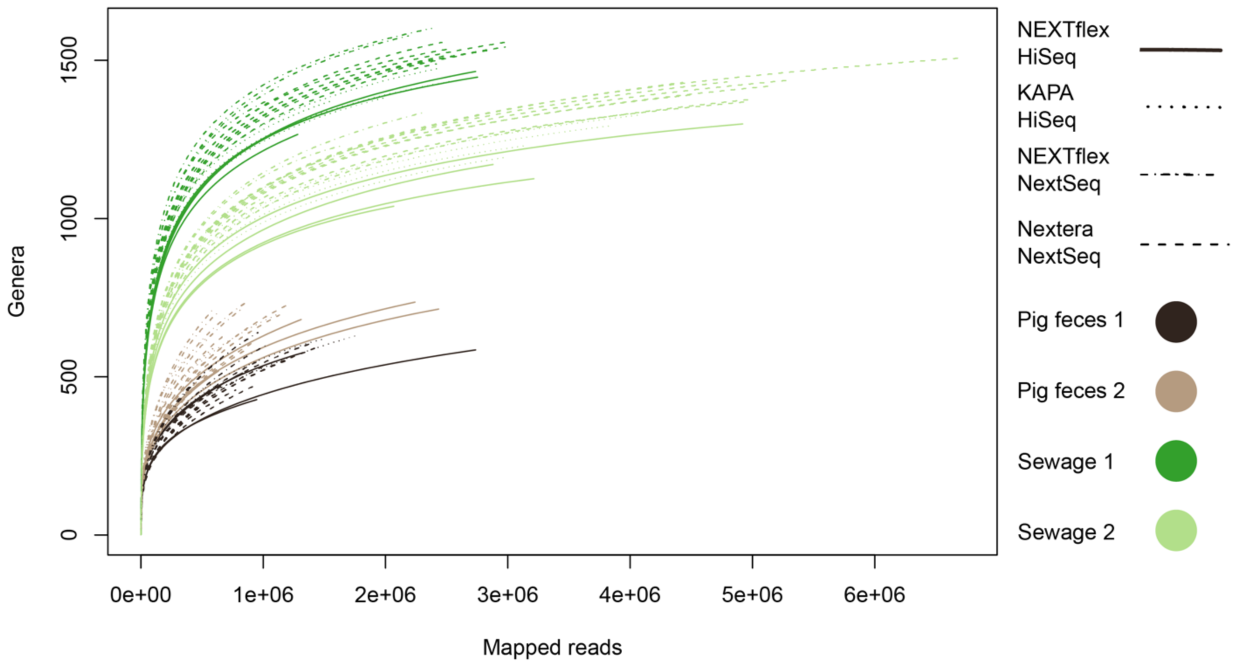

Figure S1. Rarefaction curves.

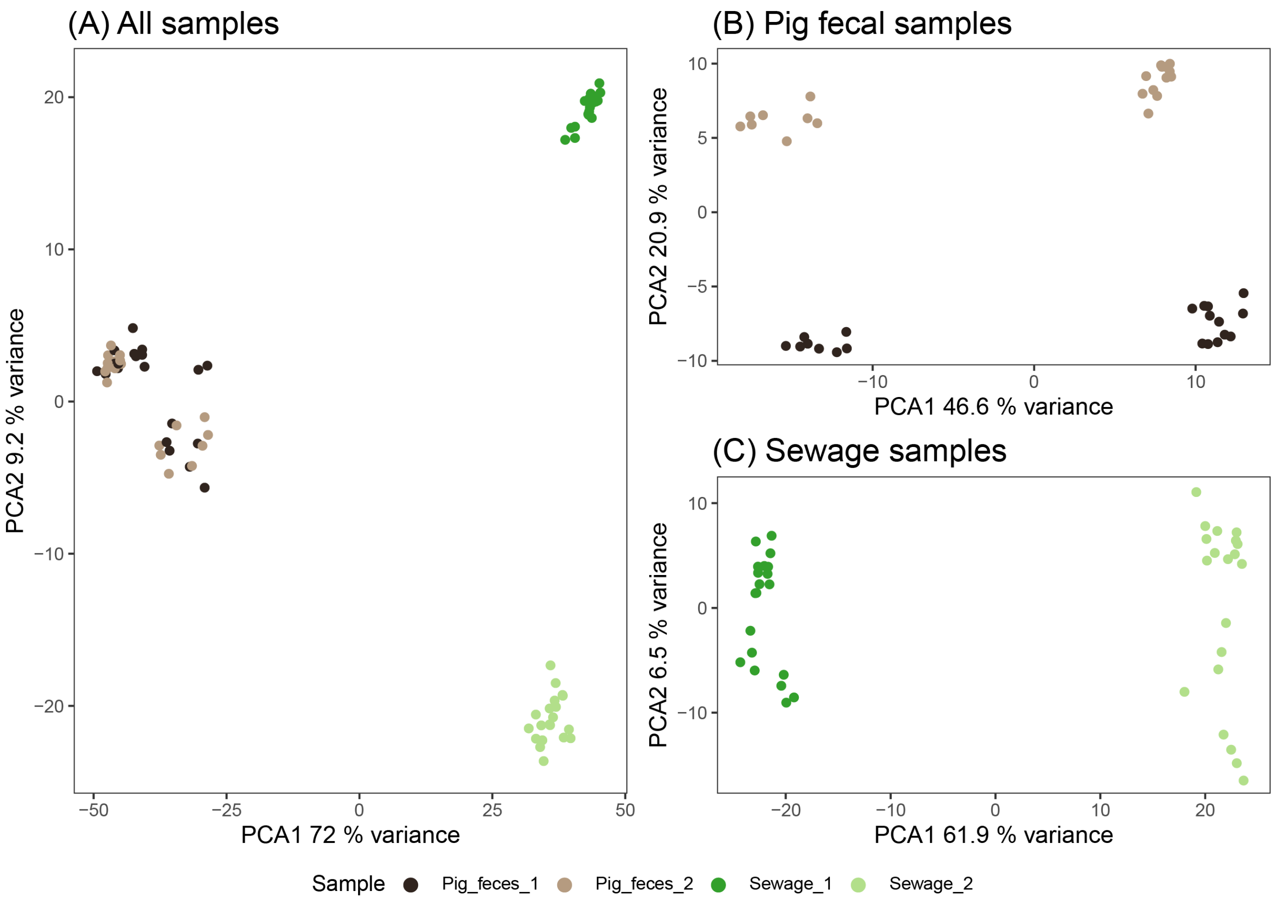

Figure S2. Principal component analysis (PCA) for all samples and subsetted to pig feces and sewage. Variance explained by the two first axes are included in their labels. (A) PCA were generated for all samples forming three clusters with pig feces together and sewage 1 and sewage 2 seperately, (B) PCA were generated for all pig feces samples separation were now observed between pig feces 1 and pig feces 2, and (C) PCA were generated for all sewage samples that were still easy to discriminate.

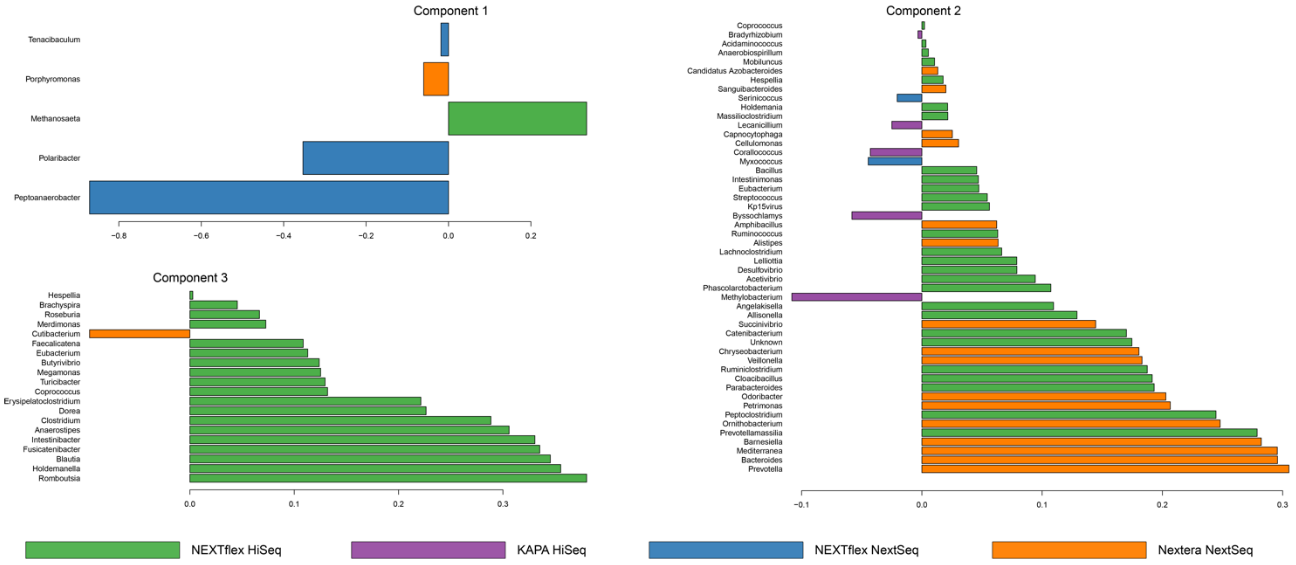

Figure S3. Sparse partial least square discriminant analysis (sPLS-DA). The sPLS-DA were run with a unique identifier for a specific DNA sample that were then processed differently in the generation of libraries and sequencing to select the most discriminative genera in explaining this aspect of sample processing.

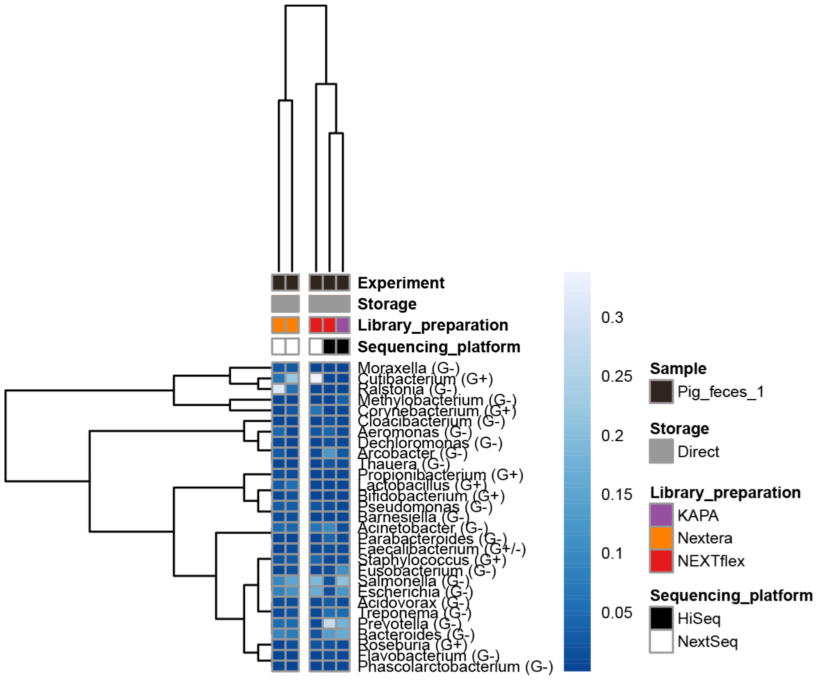

Figure S4. Heatmap of negative controls with the thirty most abundant genera. Complete-linkage clustering was performed to create dendograms for both genera and samples. Spearman correlation was used to cluster the genera and Aitchison distances were used to cluster the samples. Genera abundance depicted in the cells were CLR transformed counts standardized to zero mean and unit variance. Grouping of organisms were included in genera names according to cell wall structure based on Gram-positive staining (G+), Gram-negative staining (G-) or belonging to Archaea (Ar).

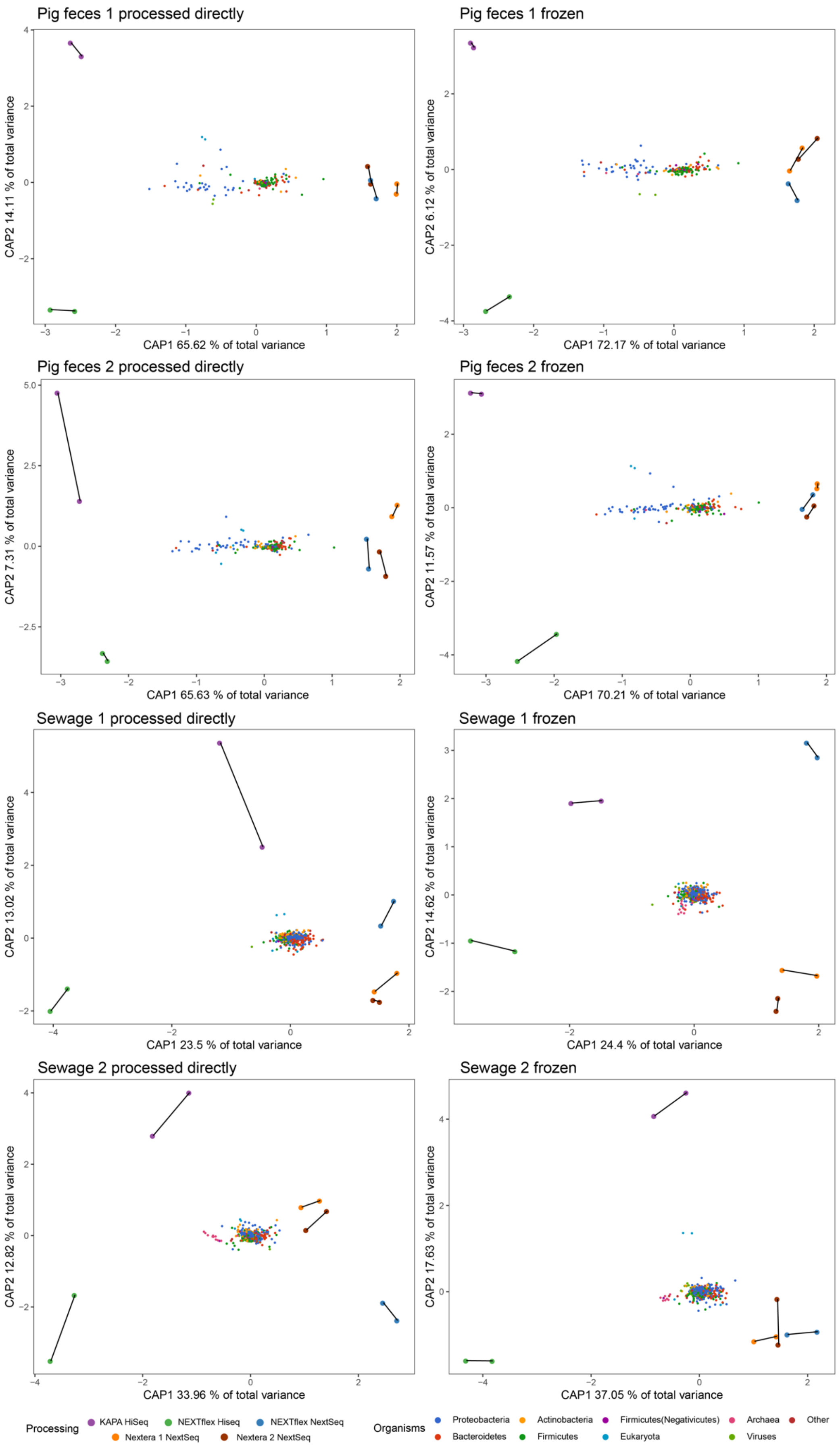
Pig feces 1 processed directly
CAP1 65.62 % of total variance
CAP2 14.11 % of total variance
Pig feces 1 frozen
CAP1 72.17 % of total variance
CAP2 6.12 % of total variance
Pig feces 2 processed directly
CAP1 65.63 % of total variance
CAP2 7.31 % of total variance
Pig feces 2 frozen
CAP1 70.21 % of total variance
CAP2 11.57 % of total variance
Sewage 1 processed directly
CAP1 23.5 % of total variance
CAP2 13.02 % of total variance
Sewage 1 frozen
CAP1 24.4 % of total variance
CAP2 14.62 % of total variance
Sewage 2 processed directly
CAP1 33.96 % of total variance
CAP2 12.82 % of total variance
Sewage 2 frozen
CAP1 37.05 % of total variance
CAP2 17.63 % of total variance
Processing
KAPA HiSeq
NEXTflex Hiseq
NEXTflex NextSeq
Nextera 1 NextSeq
Nextera 2 NextSeq
Organisms
Proteobacteria
Actinobacteria
Firmicutes(Negativicutes)
Archaea
Other
Bacteroidetes
Firmicutes
Eukaryota
Viruses

Figure S5. Redundancy analysis (rda) subsetted to sample matrix and if samples were frozen or processed directly. Taxonomic patterns were investigated by plotting genera coloured according to different taxonomic groups. DNA isolation replicates were connected with lines.

File S6. Bioanalyzer electropherograms (Nextera).

Assay Class: High Sensitivity DNA Assay
Data Path: C:\...gh Sensitivity DNA Assay_DE24802594_2018-08-22_12-28-24.xad

Created: 22-08-2018 12:28:24
Modified: 22-08-2018 13:09:44

Electrophoresis File Run Summary

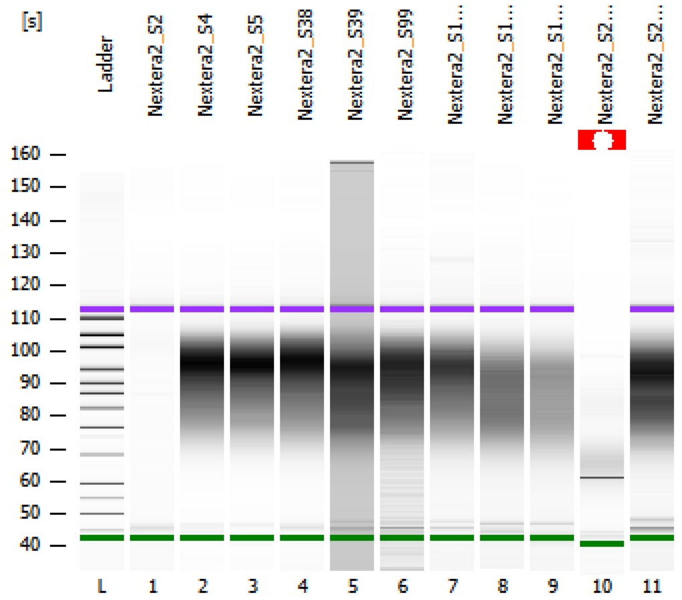

Instrument Information:

Instrument Name: DE24802594
Serial#: DE24802594
Firmware: C.01.069
Type: G2938B

Assay Information:

Assay Origin Path: C:\Program Files (x86)\Agilent\2100 bioanalyzer\2100 expert\assays\dsDNA\High Sensitivity DNA.xsy

Assay Class: High Sensitivity DNA Assay

Version: 1.03

Assay Comments: Copyright © 2003-2010 Agilent Technologies

Chip Information:

Chip Lot #:

Reagent Kit Lot #:

Chip Comments:

Nextera2_S2

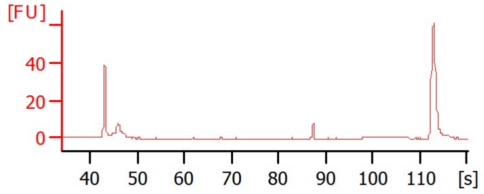

Nextera2_S4

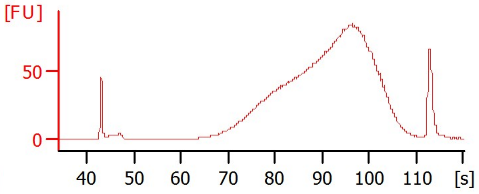

Nextera2_S5

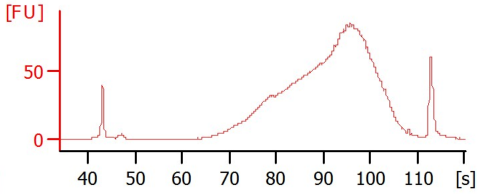

Nextera2_S38

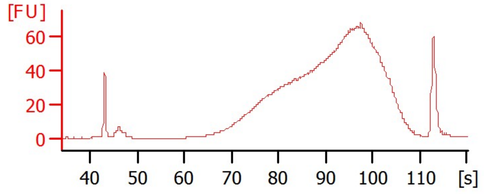

Nextera2_S39

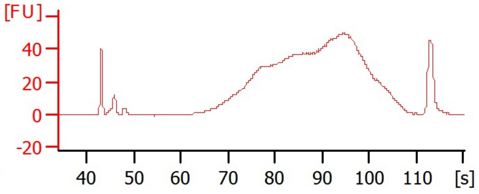

Nextera2_S99

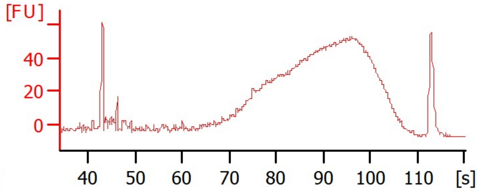

Nextera2_S101

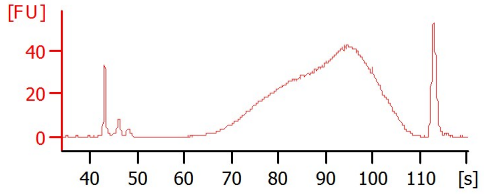

Nextera2_S160

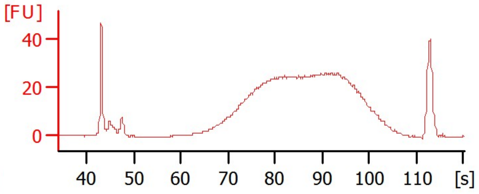

Nextera2_S161

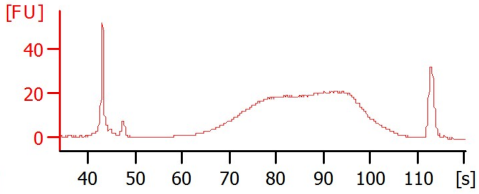

Nextera2_S222

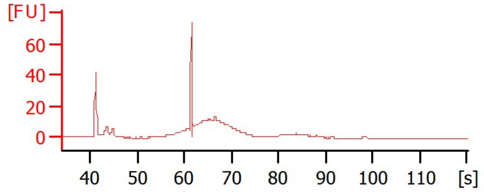

Nextera2_S223

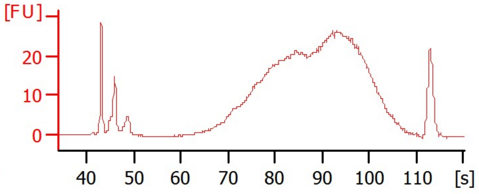

 © Copyright 2003 - 2017 Agilent Technologies, Inc.

Assay Class: High Sensitivity DNA Assay
Data Path: C:\...gh Sensitivity DNA Assay_DE24802594_2018-08-22_12-28-24.xad
Created: 22-08-2018 12:28:24
Modified: 22-08-2018 13:09:44

Electrophoresis File Run Summary (Chip Summary)

Sample Name	Sample Comment	Rest. Digest	Status	Observation	Result Label	Result Color
Nextera2_S2		☐	✓			
Nextera2_S4		☐	✓			
Nextera2_S5		☐	✓			
Nextera2_S38		☐	✓			
Nextera2_S39		☐	✓			
Nextera2_S99		☐	✓			
Nextera2_S101		☐	✓			
Nextera2_S160		☐	✓			
Nextera2_S161		☐	✓			
Nextera2_S222		☐	✓			
Nextera2_S223		☐	✓			
Ladder		☐	✓			

Chip Lot # **Reagent Kit Lot #**

Chip Comments :

© Copyright 2003 - 2017 Agilent Technologies, Inc.

Electrophoresis Assay Details

General Analysis Settings

Number of Available Sample and Ladder Wells (Max.) : 12
Minimum Visible Range [s] : 32
Maximum Visible Range [s] : 138
Start Analysis Time Range [s] : 33
End Analysis Time Range [s] : 137,5
Ladder Concentration [pg/µl] : 1950
Uses Standard Area for Ladder Fragments
Lower Marker Concentration [pg/µl] : 125
Upper Marker Concentration [pg/µl] : 75
Used Upper Marker for Quantitation
Standard Curve Fit is Point to Point
Show Data Aligned to Lower and Upper Marker

Integrator Settings

Integration Start Time [s] : 33,05
Integration End Time [s] : 137
Slope Threshold : 0,8
Height Threshold [FU] : 5
Area Threshold : 0,1
Width Threshold [s] : 0,6
Baseline Plateau [s] : 0,5

Filter Settings

Filter Width [s] : 0,5
Polynomial Order : 4

Ladder

Ladder Peak	Size	Area
1	35	160
2	50	210
3	100	208
4	150	221
5	200	242
6	300	270
7	400	305
8	500	306
9	600	336
10	700	321
11	1000	366
12	2000	413
13	3000	411
14	7000	400
15	10380	214

 © Copyright 2003 - 2017 Agilent Technologies, Inc.

Assay Class: High Sensitivity DNA Assay
Data Path: C:\...gh Sensitivity DNA Assay_DE24802594_2018-08-22_12-28-24.xad
Created: 22-08-2018 12:28:24
Modified: 22-08-2018 13:09:44

Electropherogram Summary

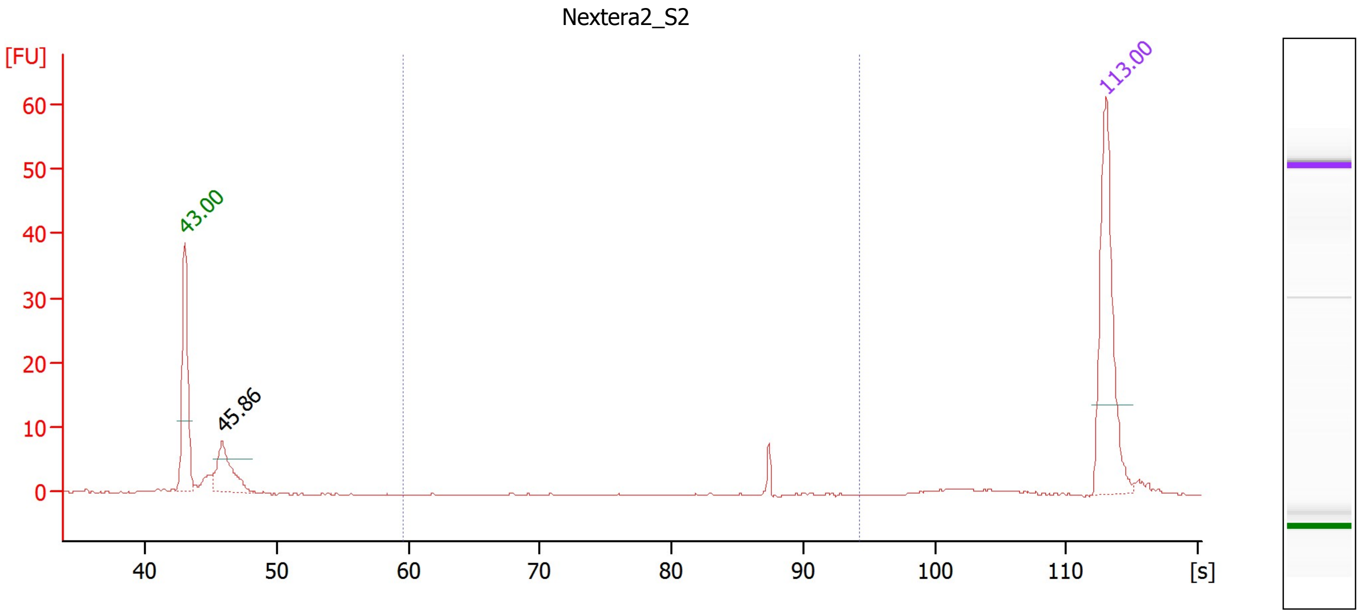

Overall Results for sample 1 : Nextera2 S2

Number of peaks found: 1
Corr. Area 1: 2,5
Noise: 0,1

Peak table for sample 1 : Nextera2 S2

Peak		Size [bp]	Conc. [pg/µl]	Molarity [pmol/l]	Observations
1	◀	35	125,00	5.411,3	Lower Marker
2		56	51,43	1.379,9	
3	▶	10.380	75,00	10,9	Upper Marker

© Copyright 2003 - 2017 Agilent Technologies, Inc.

Assay Class: High Sensitivity DNA Assay
Data Path: C:\...gh Sensitivity DNA Assay_DE24802594_2018-08-22_12-28-24.xad
Created: 22-08-2018 12:28:24
Modified: 22-08-2018 13:09:44

Electropherogram Summary Continued ...

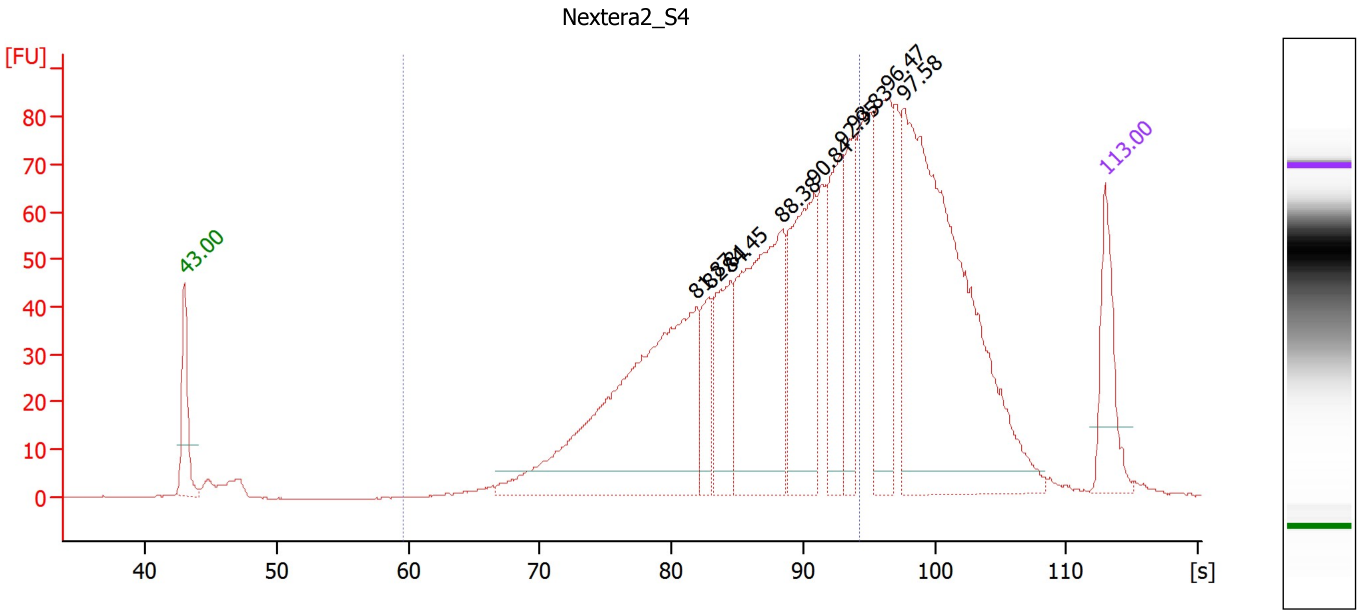

Overall Results for sample 2 : Nextera2_S4

Number of peaks found: 9
Corr. Area 1: 1.103,5
Noise: 0,1

Peak table for sample 2 : Nextera2_S4

Peak		Size [bp]	Conc. [pg/µl]	Molarity [pmol/l]	Observations
1	◀	35	125,00	5.411,3	Lower Marker
2		490	562,74	1.741,0	
3		507	67,48	201,5	
4		544	117,72	327,8	
5		646	332,01	778,7	
6		757	219,14	438,8	
7		908	131,62	219,7	
8		971	86,95	135,7	
9		1.324	159,46	182,5	
10		1.486	575,07	586,5	
11	▶	10.380	75,00	10,9	Upper Marker

 © Copyright 2003 - 2017 Agilent Technologies, Inc.

Assay Class: High Sensitivity DNA Assay
Data Path: C:\...gh Sensitivity DNA Assay_DE24802594_2018-08-22_12-28-24.xad
Created: 22-08-2018 12:28:24
Modified: 22-08-2018 13:09:44

Electropherogram Summary Continued ...

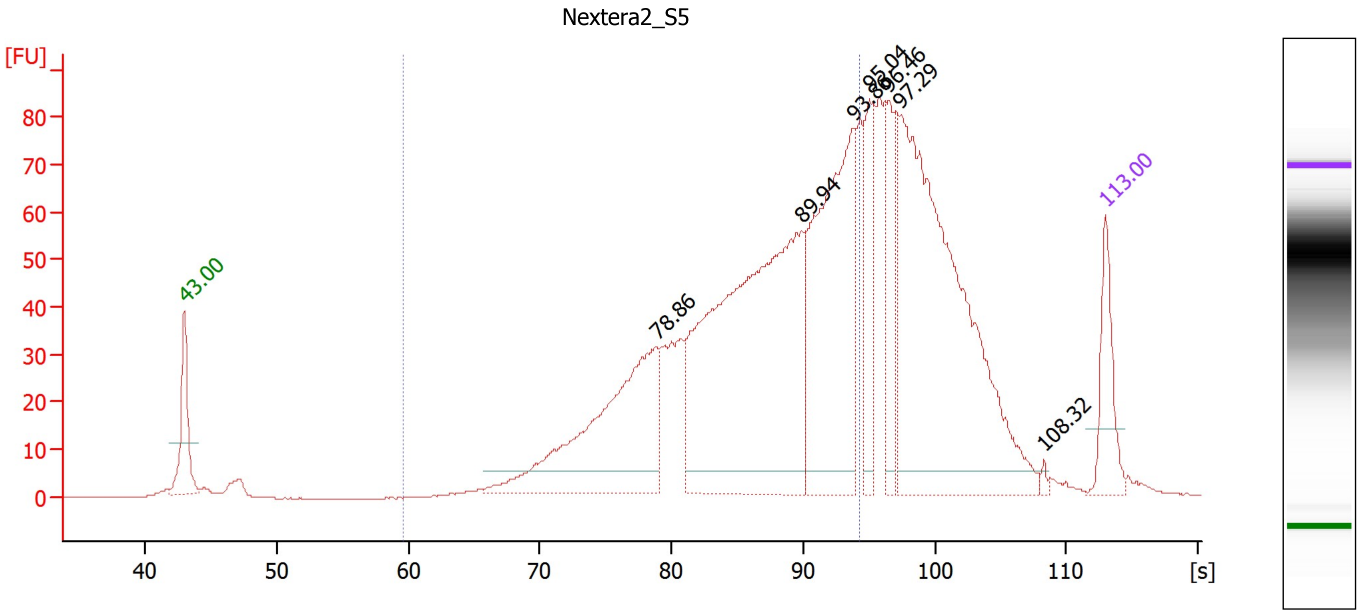

Overall Results for sample 3 : __Nextera2_S5__

Number of peaks found: 7
Corr. Area 1: 1.042,1
Noise: 0,1

Peak table for sample 3 : __Nextera2_S5__

Peak		Size [bp]	Conc. [pg/µl]	Molarity [pmol/l]	Observations
1	◀	35	125,00	5.411,3	Lower Marker
2		439	437,12	1.509,3	
3		697	841,87	1.831,2	
4		973	429,48	669,1	
5		1.117	106,56	144,6	
6		1.323	102,76	117,7	
7		1.444	677,90	711,4	
8		5.745	5,51	1,5	
9	▶	10.380	75,00	10,9	Upper Marker

© Copyright 2003 - 2017 Agilent Technologies, Inc.

Assay Class: High Sensitivity DNA Assay
Data Path: C:\...gh Sensitivity DNA Assay_DE24802594_2018-08-22_12-28-24.xad
Created: 22-08-2018 12:28:24
Modified: 22-08-2018 13:09:44

Electropherogram Summary Continued ...

Nextera2_S38

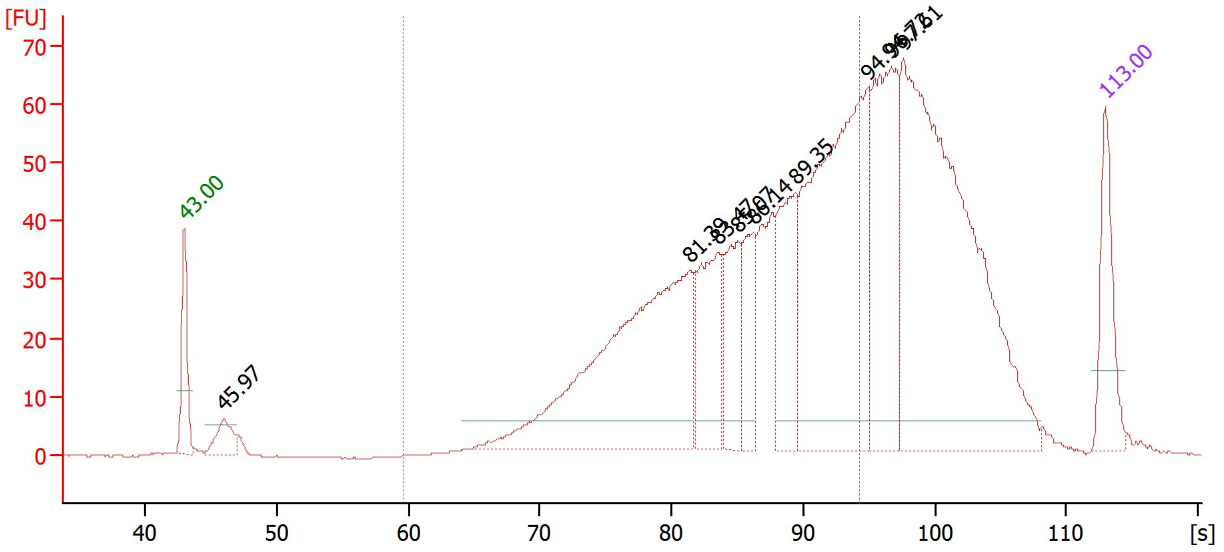

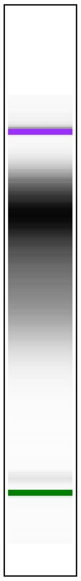

Overall Results for sample 4 : Nextera2_S38

Number of peaks found: 9
Corr. Area 1: 898,8
Noise: 0,1

Peak table for sample 4 : Nextera2_S38

Peak		Size [bp]	Conc. [pg/µl]	Molarity [pmol/l]	Observations
1	◀	35	125,00	5.411,3	Lower Marker
2		58	48,53	1.277,2	
3		482	530,43	1.668,8	
4		522	139,82	405,7	
5		558	101,30	275,0	
6		582	77,36	201,4	
7		677	137,82	308,3	
8		1.101	462,75	636,6	
9		1.360	209,87	233,8	
10		1.490	604,37	614,7	
11	▶	10.380	75,00	10,9	Upper Marker

 © Copyright 2003 - 2017 Agilent Technologies, Inc.

Assay Class: High Sensitivity DNA Assay
Data Path: C:\...gh Sensitivity DNA Assay_DE24802594_2018-08-22_12-28-24.xad
Created: 22-08-2018 12:28:24
Modified: 22-08-2018 13:09:44

Electropherogram Summary Continued ...

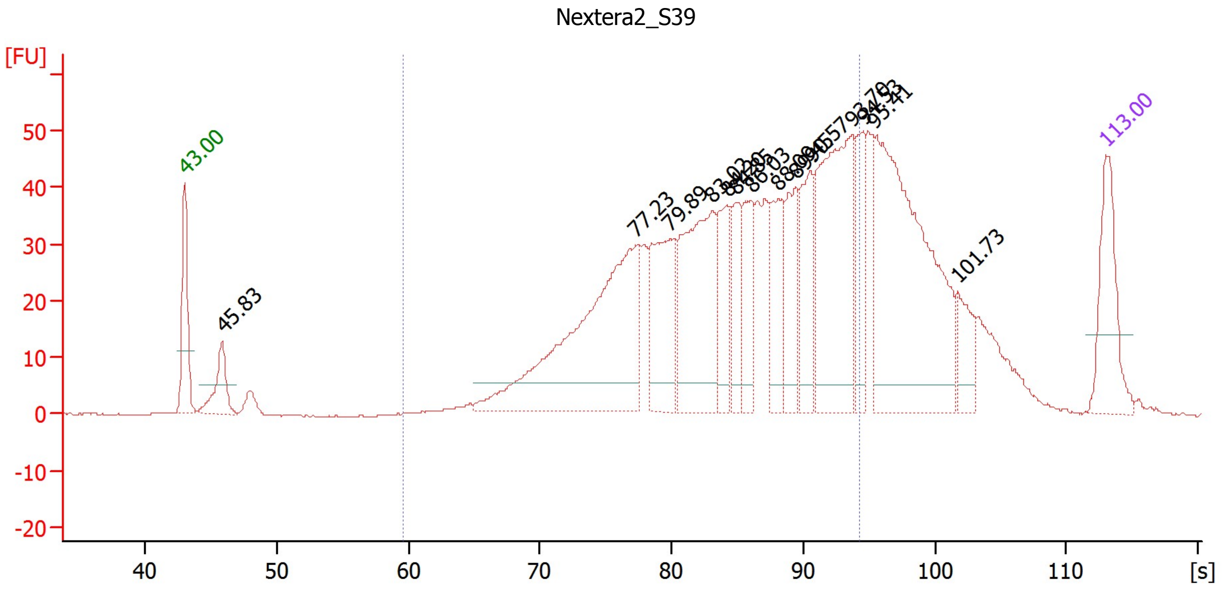

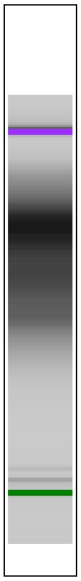

Overall Results for sample 5 : Nextera2_S39

Number of peaks found: 14
Corr. Area 1: 1.105,1
Noise: 0,1

Peak table for sample 5 : Nextera2_S39

Peak		Size [bp]	Conc. [pg/µl]	Molarity [pmol/l]	Observations
1	◄	35	125,00	5.411,3	Lower Marker
2		56	63,68	1.715,2	
3		411	375,50	1.383,3	
4		456	128,98	428,4	
5		512	209,31	619,3	
6		538	68,66	193,2	
7		553	55,87	153,1	
8		579	62,63	163,8	
9		637	73,90	175,8	
10		681	76,93	171,2	
11		737	84,68	174,0	
12		961	223,27	351,8	
13		1.042	59,15	86,0	
14		1.170	335,70	434,6	
15		2.162	33,77	23,7	
16	►	10.380	75,00	10,9	Upper Marker

 © Copyright 2003 - 2017 Agilent Technologies, Inc.

Assay Class: High Sensitivity DNA Assay
Data Path: C:\...gh Sensitivity DNA Assay_DE24802594_2018-08-22_12-28-24.xad

Created: 22-08-2018 12:28:24
Modified: 22-08-2018 13:09:44

Electropherogram Summary Continued ...

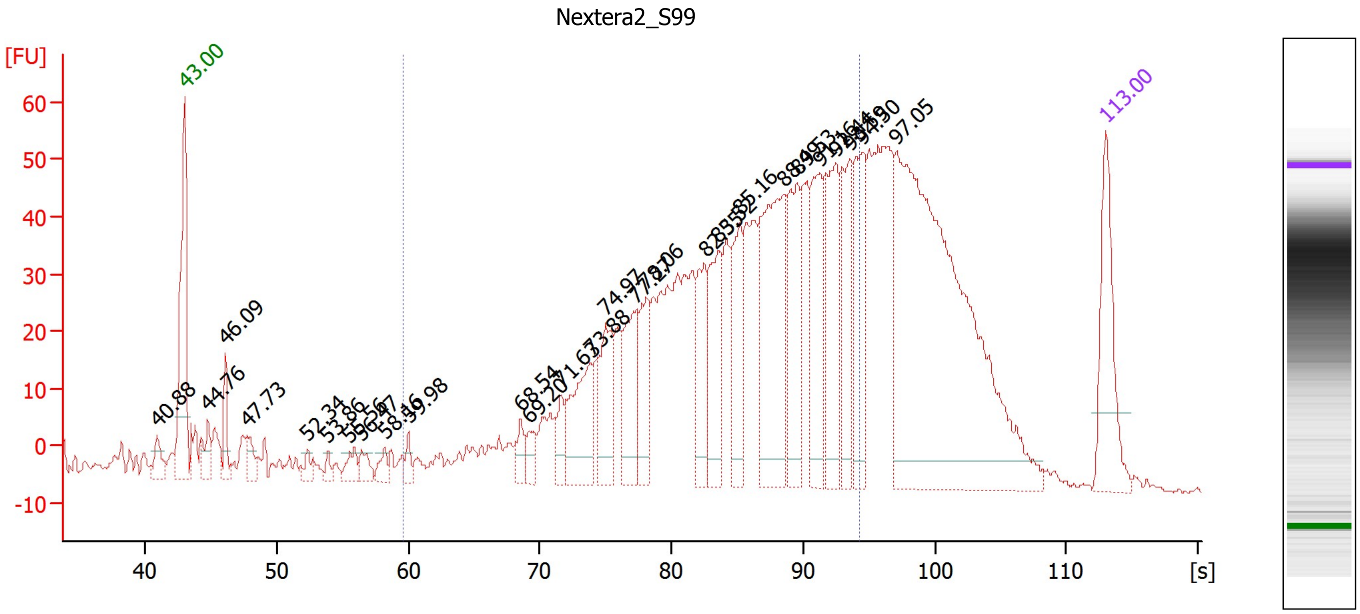

Overall Results for sample 6 : Nextera2_S99

Number of peaks found: 27

Corr. Area 1: 1.048,4

Noise: 0,2

Peak table for sample 6 : Nextera2_S99

Peak	Size [bp]	Conc. [pg/µl]	Molarity [pmol/l]	Observations
1	28	0,00	0,0	
2	35	125,00	5.411,3	Lower Marker
3	47	32,41	1.042,6	
4	59	36,20	933,8	
5	74	23,67	481,9	
6	121	13,58	169,8	
7	138	10,12	111,4	
8	156	20,69	200,6	
9	166	17,71	161,4	
10	185	14,62	119,7	
11	205	14,08	103,9	
12	302	19,69	98,8	
13	310	18,94	92,5	
14	340	27,45	122,4	
15	367	84,22	347,5	
16	381	67,33	268,1	
17	412	72,90	268,1	
18	425	66,32	236,3	
19	502	70,08	211,7	
20	523	80,85	234,1	
21	560	74,98	202,9	
22	650	181,24	422,6	
23	683	101,63	225,4	
24	780	95,54	185,6	
25	871	103,69	180,4	
26	954	68,78	109,3	

 © Copyright 2003 - 2017 Agilent Technologies, Inc.

Assay Class:	High Sensitivity DNA Assay	Created:	22-08-2018 12:28:24
Data Path:	C:\...gh Sensitivity DNA Assay_DE24802594_2018-08-22_12-28-24.xad	Modified:	22-08-2018 13:09:44

Electropherogram Summary Continued ...

... Peak table for sample 6 : Nextera2_S99

Peak		Size [bp]	Conc. [pg/µl]	Molarity [pmol/l]	Observations
27		1.038	73,82	107,8	
28		1.408	495,06	532,7	
29	▶	10.380	75,00	10,9	Upper Marker

 © Copyright 2003 - 2017 Agilent Technologies, Inc.

Assay Class: High Sensitivity DNA Assay
Data Path: C:\...gh Sensitivity DNA Assay_DE24802594_2018-08-22_12-28-24.xad
Created: 22-08-2018 12:28:24
Modified: 22-08-2018 13:09:44

Electropherogram Summary Continued ...

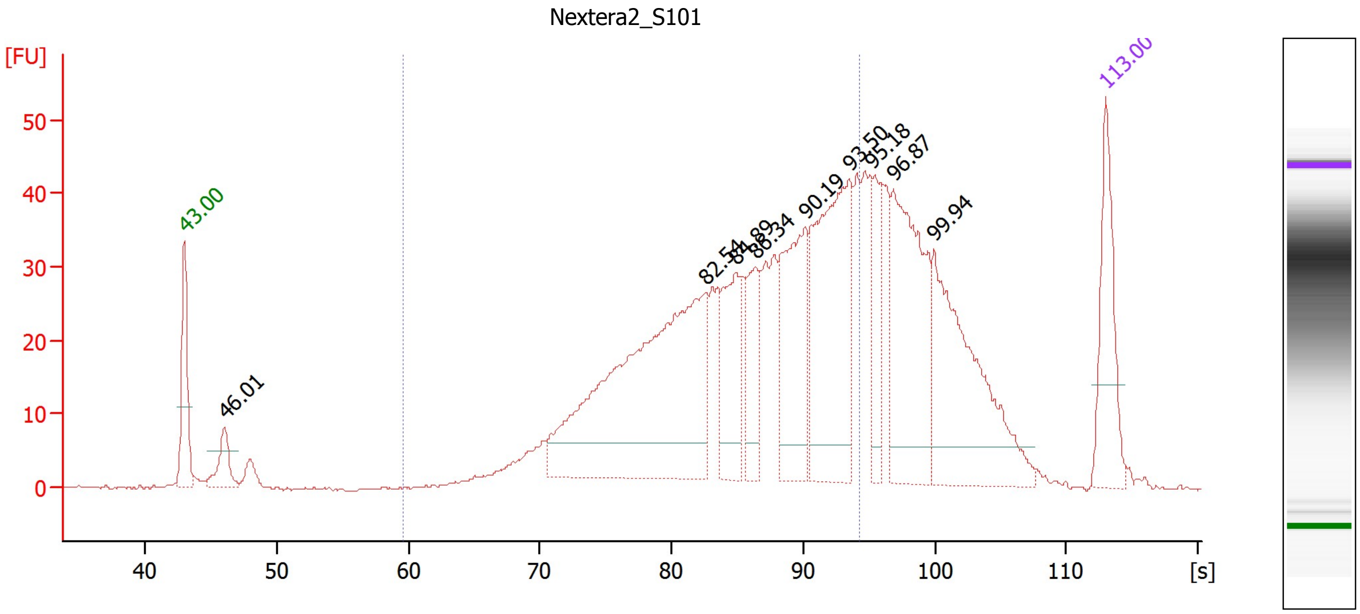

Overall Results for sample 7 : Nextera2_S101

Number of peaks found: 9
Corr. Area 1: 695,7
Noise: 0,2

Peak table for sample 7 : Nextera2_S101

Peak	Size [bp]	Conc. [pg/µl]	Molarity [pmol/l]	Observations
1	35	125,00	5.411,3	Lower Marker
2	58	44,60	1.166,2	
3	502	446,23	1.348,1	
4	554	96,81	264,8	
5	586	59,66	154,2	
6	710	141,68	302,4	
7	947	219,69	351,5	
8	1.137	53,89	71,8	
9	1.382	171,92	188,5	
10	1.828	175,73	145,6	
11	10.380	75,00	10,9	Upper Marker

 © Copyright 2003 - 2017 Agilent Technologies, Inc.

Assay Class: High Sensitivity DNA Assay
Data Path: C:\...gh Sensitivity DNA Assay_DE24802594_2018-08-22_12-28-24.xad

Created: 22-08-2018 12:28:24
Modified: 22-08-2018 13:09:44

Electropherogram Summary Continued ...

Nextera2_S160

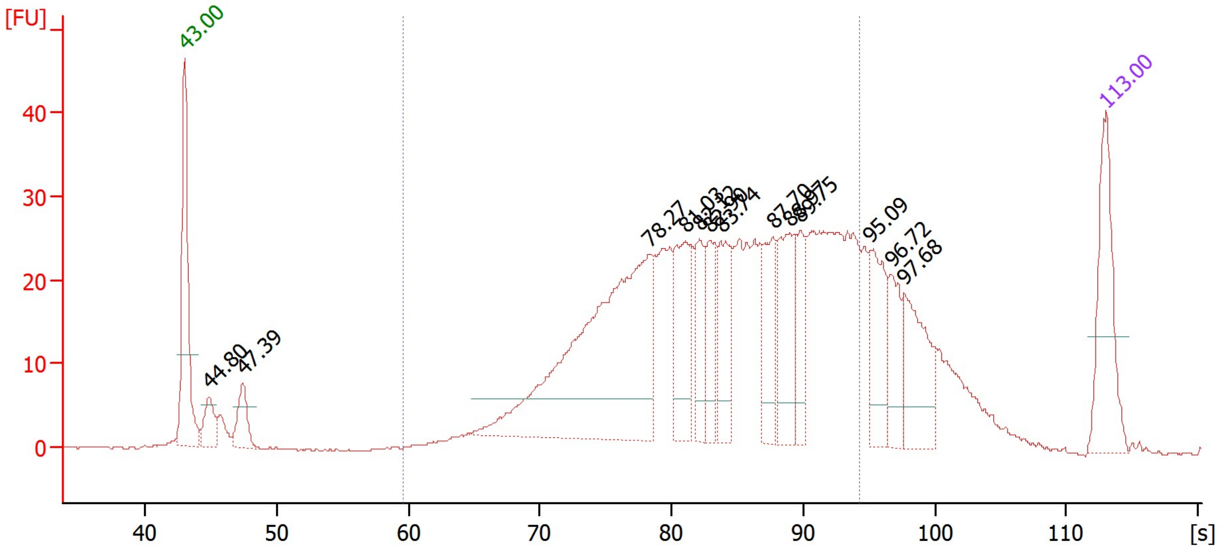

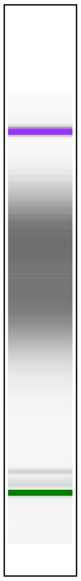

Overall Results for sample 8 : Nextera2_S160

Number of peaks found: 13 Corr. Area 1: 651,1

Noise: 0,1

Peak table for sample 8 : Nextera2_S160

Peak		Size [bp]	Conc. [pg/µl]	Molarity [pmol/l]	Observations
1	◀	35	125,00	5.411,3	Lower Marker
2		47	37,93	1.212,4	
3		71	43,98	936,8	
4		429	400,48	1.414,9	
5		476	92,17	293,6	
6		494	49,89	153,1	
7		509	49,23	146,4	
8		528	65,96	189,2	
9		624	64,22	155,9	
10		665	77,09	175,6	
11		690	46,40	101,9	
12		1.124	62,07	83,6	
13		1.360	39,18	43,6	
14		1.500	65,91	66,6	
15	▶	10.380	75,00	10,9	Upper Marker

 © Copyright 2003 - 2017 Agilent Technologies, Inc.

Assay Class: High Sensitivity DNA Assay
Data Path: C:\...gh Sensitivity DNA Assay_DE24802594_2018-08-22_12-28-24.xad
Created: 22-08-2018 12:28:24
Modified: 22-08-2018 13:09:44

Electropherogram Summary Continued ...

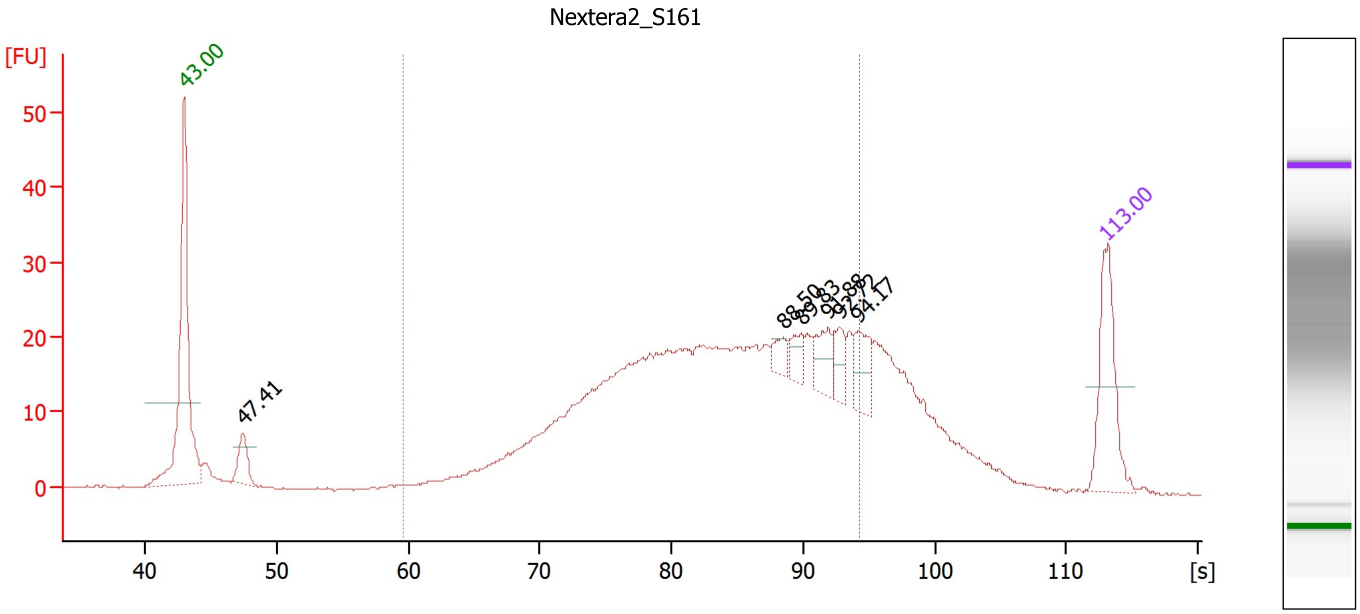

Overall Results for sample 9 : Nextera2_S161

Number of peaks found: 6
Corr. Area 1: 522,7
Noise: 0,1

Peak table for sample 9 : Nextera2_S161

Peak		Size [bp]	Conc. [pg/µl]	Molarity [pmol/l]	Observations
1	◀	35	125,00	5.411,3	Lower Marker
2		71	40,98	870,8	
3		650	15,23	35,5	
4		693	17,11	37,4	
5		831	30,81	56,2	
6		892	21,04	35,7	
7		995	33,32	50,7	
8	▶	10.380	75,00	10,9	Upper Marker

© Copyright 2003 - 2017 Agilent Technologies, Inc.

Assay Class:	High Sensitivity DNA Assay	Created:	22-08-2018 12:28:24
Data Path:	C:\...gh Sensitivity DNA Assay_DE24802594_2018-08-22_12-28-24.xad	Modified:	22-08-2018 13:09:44

Electropherogram Summary Continued ...

Nextera2_S222

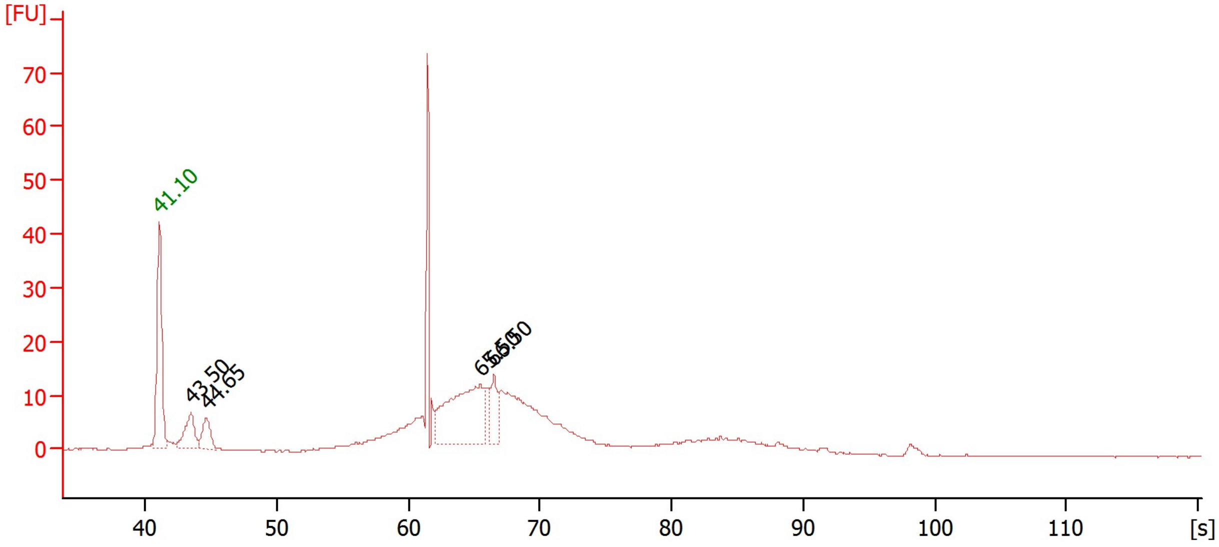

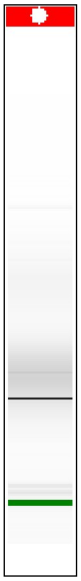

Overall Results for sample 10 : **Nextera2_S222**

Number of peaks found: 4 Noise: 0,1

Peak table for sample 10 : **Nextera2_S222**

Peak		Size [bp]	Conc. [pg/µl]	Molarity [pmol/l]	Observations
1	◀	0	0,00	0,0	Lower Marker
2		0	0,00	0,0	
3		0	0,00	0,0	
4		0	0,00	0,0	
5		0	0,00	0,0	

 © Copyright 2003 - 2017 Agilent Technologies, Inc.

Assay Class:	High Sensitivity DNA Assay	Created:	22-08-2018 12:28:24
Data Path:	C:\...gh Sensitivity DNA Assay_DE24802594_2018-08-22_12-28-24.xad	Modified:	22-08-2018 13:09:44

Electropherogram Summary Continued ...

Nextera2_S223

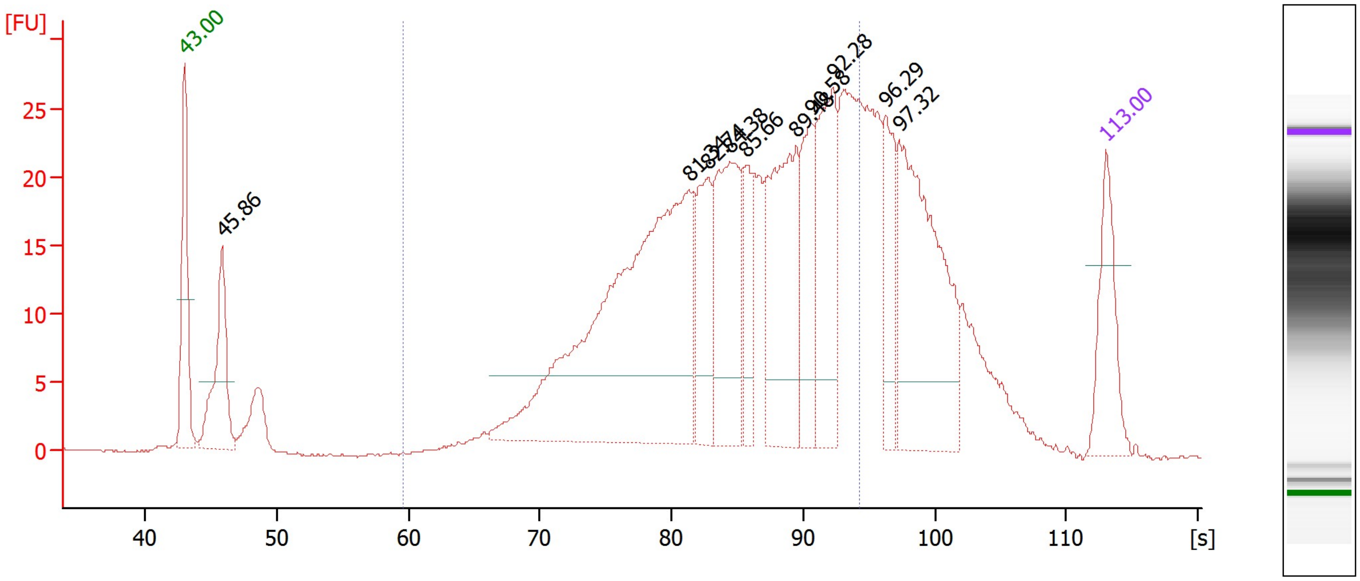

Overall Results for sample 11 : Nextera2_S223

Number of peaks found:	10	Corr. Area 1:	493,8
Noise:	0,1		

Peak table for sample 11 : Nextera2_S223

Peak		Size [bp]	Conc. [pg/µl]	Molarity [pmol/l]	Observations
1	◀	35	125,00	5.411,3	Lower Marker
2		56	163,78	4.394,5	
3		481	668,15	2.105,4	
4		506	111,13	332,8	
5		543	181,12	505,8	
6		571	66,65	176,8	
7		682	222,09	493,6	
8		738	99,76	204,8	
9		860	156,68	276,1	
10		1.298	73,06	85,3	
11		1.448	249,94	261,5	
12	▶	10.380	75,00	10,9	Upper Marker

 © Copyright 2003 - 2017 Agilent Technologies, Inc.

Assay Class: High Sensitivity DNA Assay
Data Path: C:\...gh Sensitivity DNA Assay_DE24802594_2018-08-22_12-28-24.xad
Created: 22-08-2018 12:28:24
Modified: 22-08-2018 13:09:44

Gel Image

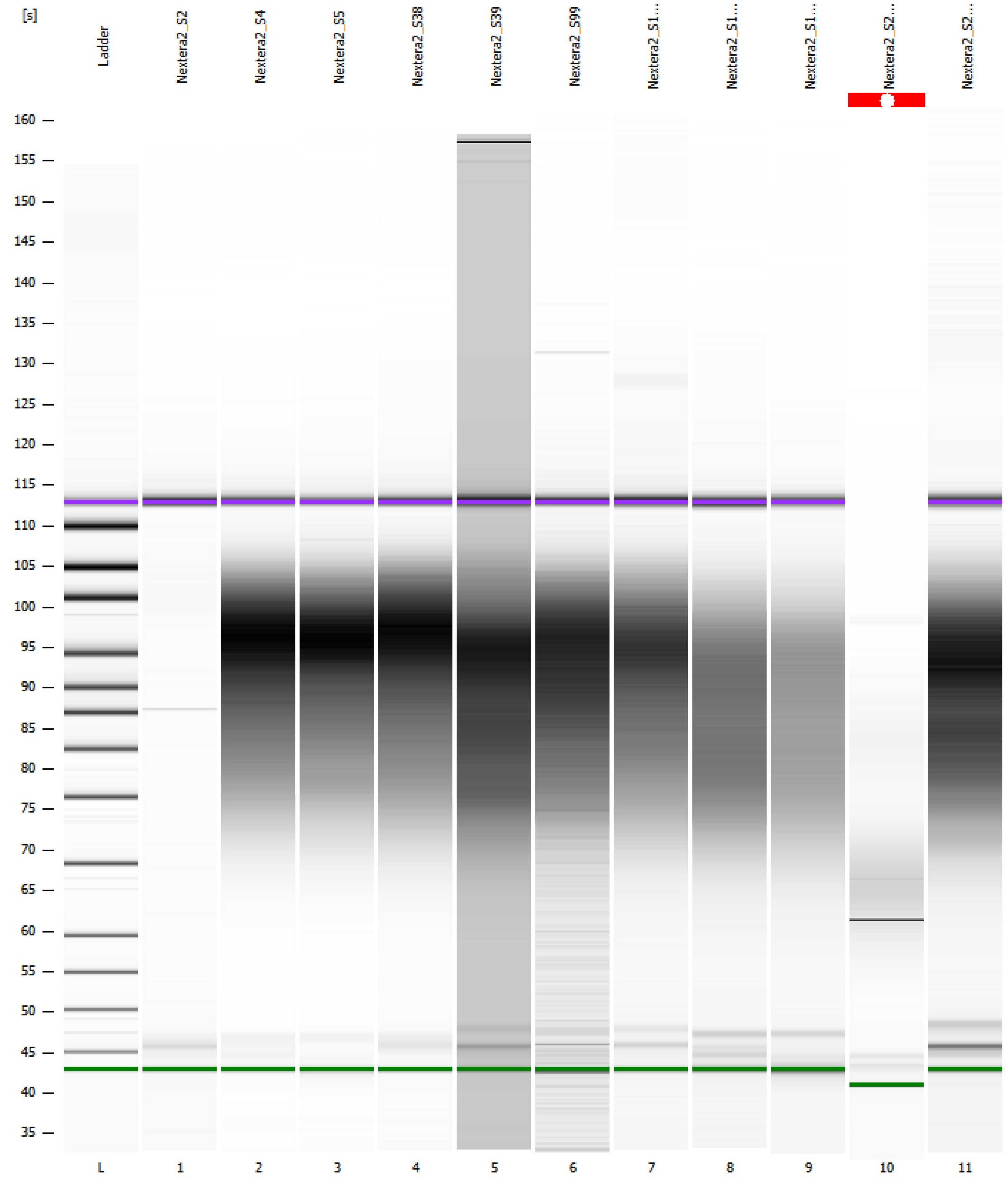

 © Copyright 2003 - 2017 Agilent Technologies, Inc.

Assay Class: High Sensitivity DNA Assay
Data Path: C:\...gh Sensitivity DNA Assay_DE24802594_2018-08-22_12-28-24.xad
Created: 22-08-2018 12:28:24
Modified: 22-08-2018 13:09:44

Curves

Standard Curve

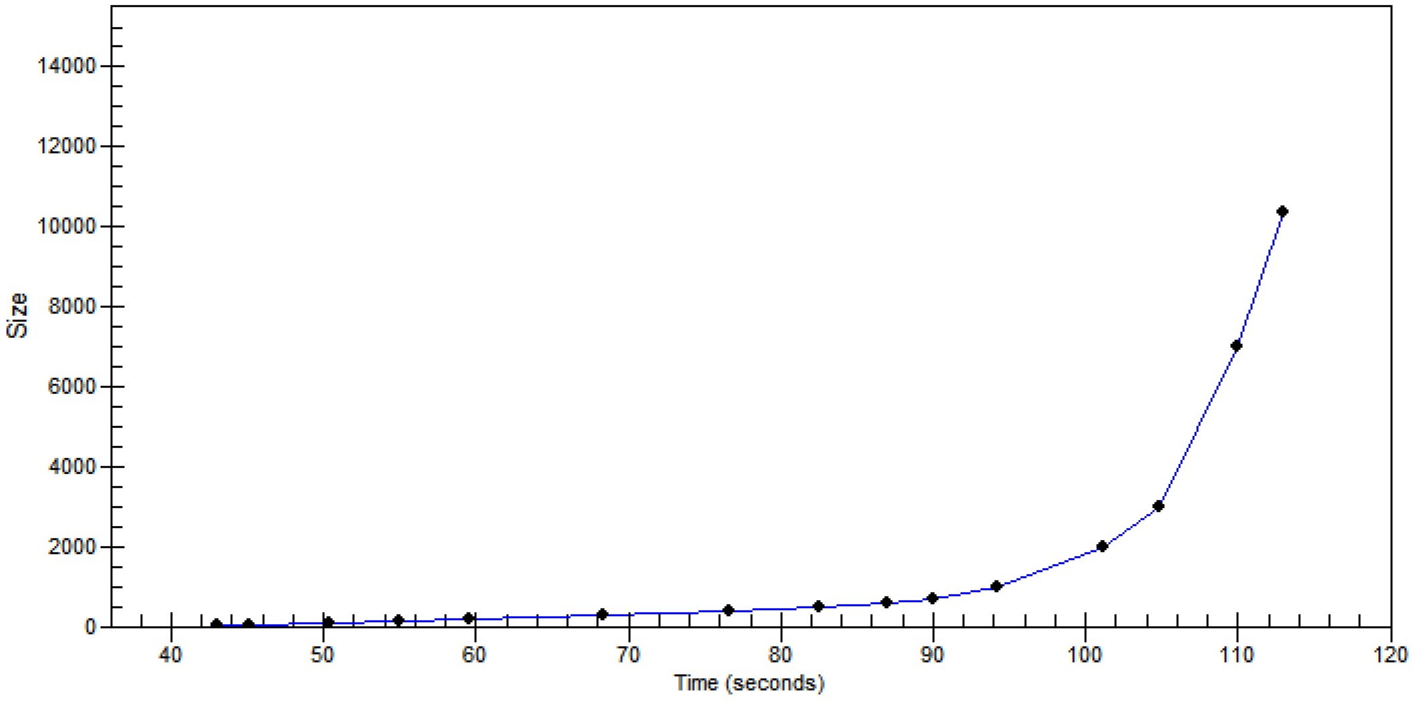

© Copyright 2003 - 2017 Agilent Technologies, Inc.

File S6. Bioanalyzer electropherograms (NextFlex, NextSeq).

Assay Class: High Sensitivity DNA Assay
Data Path: C:\...gh Sensitivity DNA Assay_DE72901621_2018-03-16_14-53-45.xad

Created: 3/16/2018 2:53:45 PM
Modified: 3/16/2018 3:34:15 PM

Electrophoresis File Run Summary

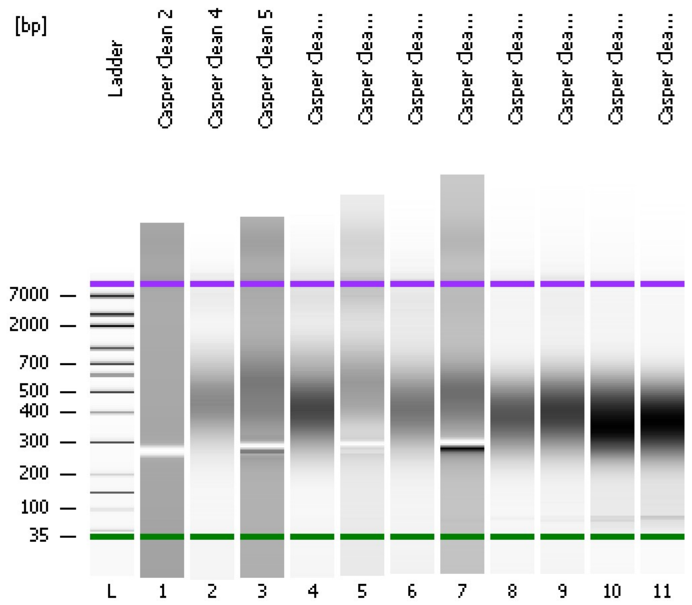

Instrument Information:

Instrument Name: DE72901621
Firmware: C.01.069
Serial#: DE72901621
Type: G2939A

Assay Information:

Assay Origin Path: C:\Program Files\Agilent\2100 bioanalyzer\2100 expert\assays\dsDNA\High Sensitivity DNA.xsy

Assay Class: High Sensitivity DNA Assay

Version: 1.03

Assay Comments: Copyright © 2003-2010 Agilent Technologies

Chip Information:

Chip Lot #:

Reagent Kit Lot #:

Chip Comments:

Casper Clean 2

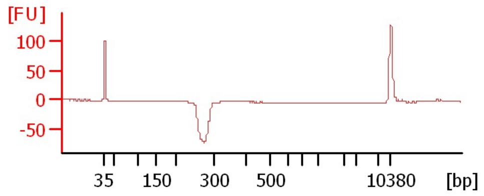

Casper Clean 4

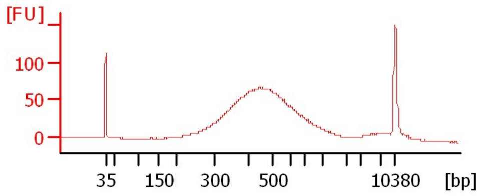

Casper Clean 5

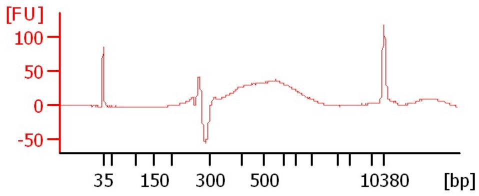

Casper Clean 38

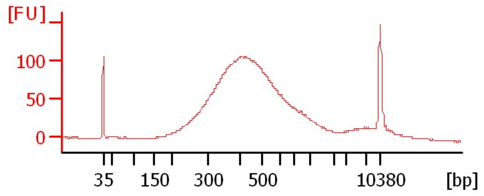

Casper Clean 39

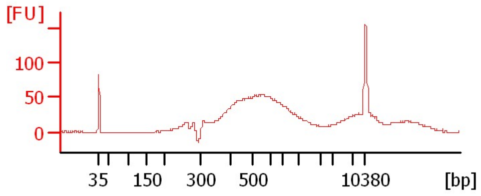

Casper Clean 99

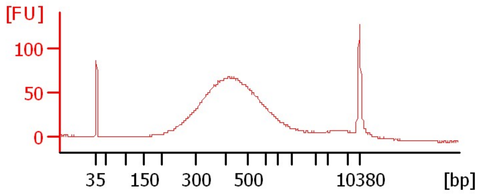

Casper Clean 101

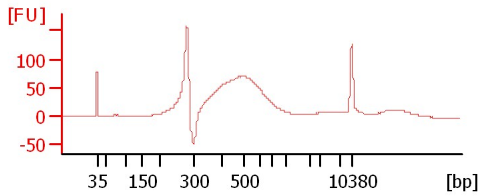

Casper Clean 160

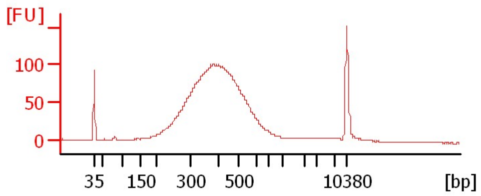

Casper Clean 161

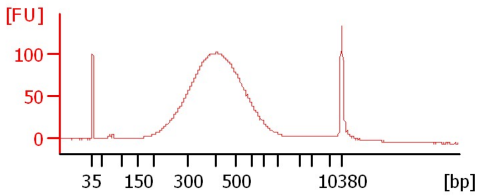

Casper Clean 222

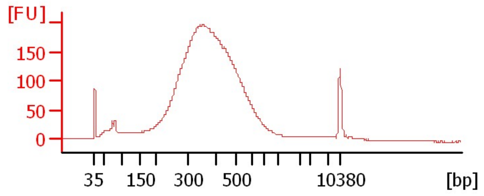

Casper Clean 223

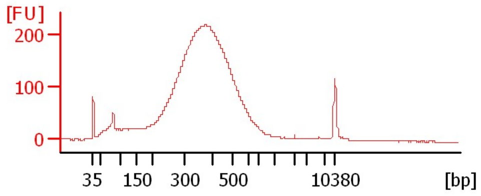

 © Copyright 2003 - 2009 Agilent Technologies, Inc.

Assay Class:	High Sensitivity DNA Assay	Created:	3/16/2018 2:53:45 PM
Data Path:	C:\...gh Sensitivity DNA Assay_DE72901621_2018-03-16_14-53-45.xad	Modified:	3/16/2018 3:34:15 PM

Electrophoresis File Run Summary (Chip Summary)

Sample Name	Sample Comment	Rest. Digest	Status	Observation	Result Label	Result Color
Casper Clean 2		☐	✔			
Casper Clean 4		☐	✔			
Casper Clean 5		☐	✔			
Casper Clean 38		☐	✔			
Casper Clean 39		☐	✔			
Casper Clean 99		☐	✔			
Casper Clean 101		☐	✔			
Casper Clean 160		☐	✔			
Casper Clean 161		☐	✔			
Casper Clean 222		☐	✔			
Casper Clean 223		☐	✔			
Ladder		☐	✔			

Chip Lot # **Reagent Kit Lot #**

Chip Comments :

 © Copyright 2003 - 2009 Agilent Technologies, Inc.

Assay Class: High Sensitivity DNA Assay Created: 3/16/2018 2:53:45 PM
Data Path: C:\...gh Sensitivity DNA Assay_DE72901621_2018-03-16_14-53-45.xad Modified: 3/16/2018 3:34:15 PM

Electrophoresis Assay Details

General Analysis Settings

Number of Available Sample and Ladder Wells (Max.) : 12
Minimum Visible Range [s] : 32
Maximum Visible Range [s] : 138
Start Analysis Time Range [s] : 33
End Analysis Time Range [s] : 137.5
Ladder Concentration [pg/µl] : 1950
Uses Standard Area for Ladder Fragments
Lower Marker Concentration [pg/µl] : 125
Upper Marker Concentration [pg/µl] : 75
Used Upper Marker for Quantitation
Standard Curve Fit is Point to Point
Show Data Aligned to Lower and Upper Marker

Integrator Settings

Integration Start Time [s] : 33.05
Integration End Time [s] : 137
Slope Threshold : 0.8
Height Threshold [FU] : 5
Area Threshold : 0.1
Width Threshold [s] : 0.6
Baseline Plateau [s] : 0.5

Filter Settings

Filter Width [s] : 0.5
Polynomial Order : 4

Ladder

Ladder Peak	Size	Area
1	35	160
2	50	210
3	100	208
4	150	221
5	200	242
6	300	270
7	400	305
8	500	306
9	600	336
10	700	321
11	1000	366
12	2000	413
13	3000	411
14	7000	400
15	10380	214

 © Copyright 2003 - 2009 Agilent Technologies, Inc.

Assay Class: High Sensitivity DNA Assay
Data Path: C:\...gh Sensitivity DNA Assay_DE72901621_2018-03-16_14-53-45.xad

Created: 3/16/2018 2:53:45 PM
Modified: 3/16/2018 3:34:15 PM

Electropherogram Summary

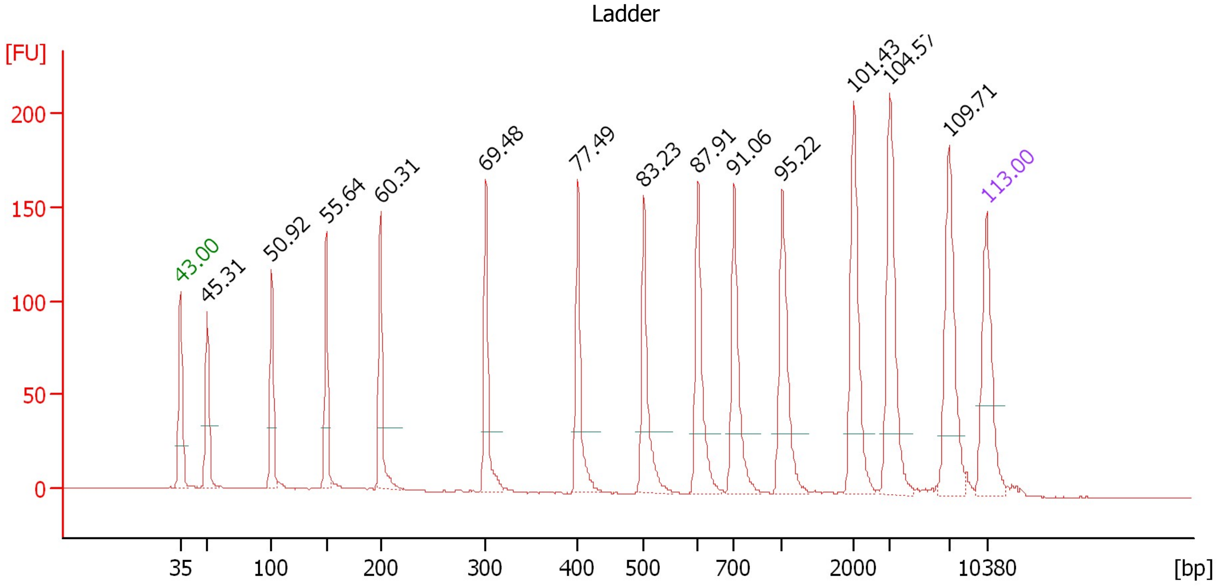

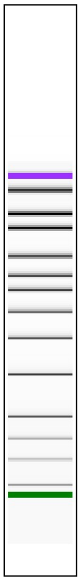

Overall Results for Ladder

Noise: 0.3

Peak table for Ladder

Peak		Size [bp]	Conc. [pg/µl]	Molarity [pmol/l]	Observations
1		35	125.00	5,411.3	Lower Marker
2		50	150.00	4,545.5	Ladder Peak
3		100	150.00	2,272.7	Ladder Peak
4		150	150.00	1,515.2	Ladder Peak
5		200	150.00	1,136.4	Ladder Peak
6		300	150.00	757.6	Ladder Peak
7		400	150.00	568.2	Ladder Peak
8		500	150.00	454.5	Ladder Peak
9		600	150.00	378.8	Ladder Peak
10		700	150.00	324.7	Ladder Peak
11		1,000	150.00	227.3	Ladder Peak
12		2,000	150.00	113.6	Ladder Peak
13		3,000	150.00	75.8	Ladder Peak
14		7,000	150.00	32.5	Ladder Peak
15		10,380	75.00	10.9	Upper Marker

 © Copyright 2003 - 2009 Agilent Technologies, Inc.

Assay Class:	High Sensitivity DNA Assay	Created:	3/16/2018 2:53:45 PM
Data Path:	C:\...gh Sensitivity DNA Assay_DE72901621_2018-03-16_14-53-45.xad	Modified:	3/16/2018 3:34:15 PM

Electropherogram Summary Continued ...

Casper Clean 2

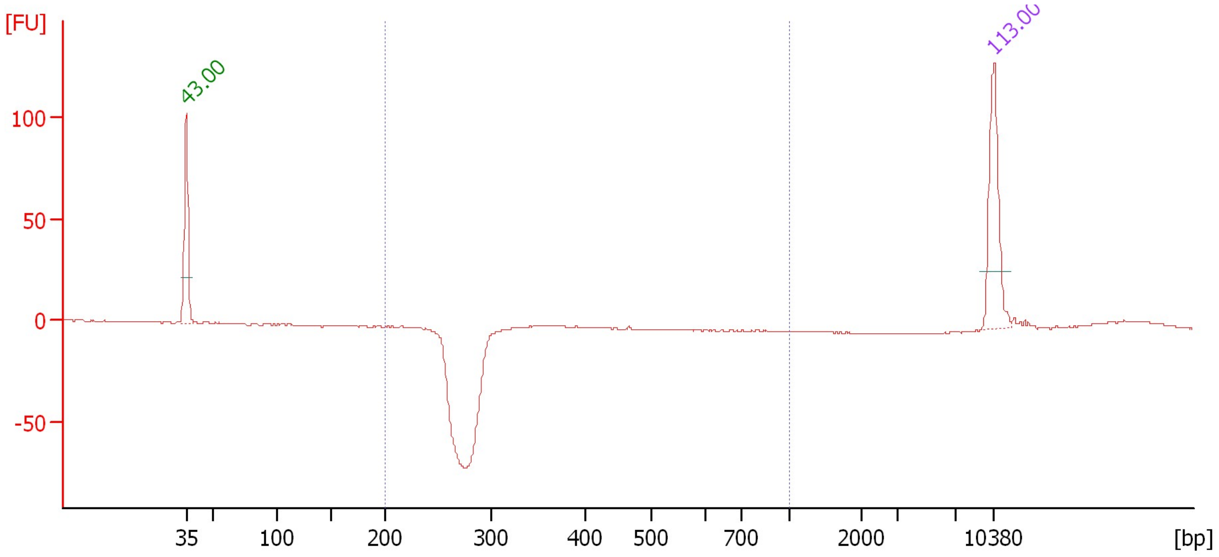

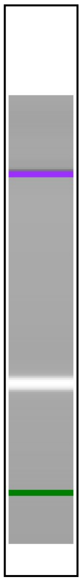

Overall Results for sample 1 : **Casper Clean 2**

Number of peaks found:	0	Corr. Area 1:	0.0
Noise:	0.3		

Peak table for sample 1 : **Casper Clean 2**

Peak	Size [bp]	Conc. [pg/µl]	Molarity [pmol/l]	Observations
1	35	125.00	5,411.3	Lower Marker
2	10,380	75.00	10.9	Upper Marker

Region table for sample 1 : **Casper Clean 2**

From [bp]	To [bp]	Corr. Area	% of Total	Average Size [bp]	Size distribution in CV [%]	Conc. [pg/µl]	Molarity [pmol/l]	Co lor
200	1,000	0.0	0	0	0.0	0.00	0.0	

© Copyright 2003 - 2009 Agilent Technologies, Inc.

Assay Class:	High Sensitivity DNA Assay	Created:	3/16/2018 2:53:45 PM
Data Path:	C:\...gh Sensitivity DNA Assay_DE72901621_2018-03-16_14-53-45.xad	Modified:	3/16/2018 3:34:15 PM

Electropherogram Summary Continued ...

Casper Clean 4

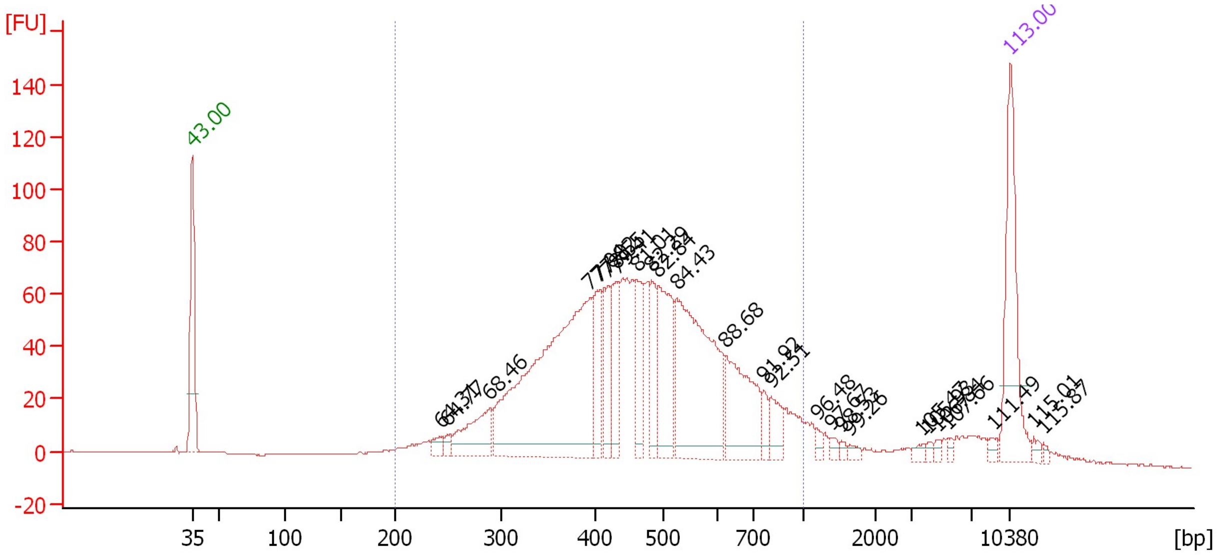

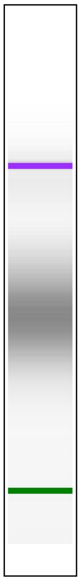

Overall Results for sample 2 : **Casper Clean 4**

Number of peaks found:	25	Corr. Area 1:	1,657.8
Noise:	0.2		

Peak table for sample 2 : **Casper Clean 4**

Peak		Size [bp]	Conc. [pg/µl]	Molarity [pmol/l]	Observations
1	◀	35	125.00	5,411.3	Lower Marker
2		244	10.41	64.7	
3		249	7.05	43.0	
4		289	55.59	291.6	
5		394	342.19	1,314.6	
6		406	42.65	159.3	
7		418	37.67	136.4	
8		434	49.85	174.2	
9		461	46.44	152.5	
10		484	39.42	123.5	
11		493	82.47	253.4	
12		526	187.51	540.4	
13		624	79.71	193.4	
14		762	11.38	22.6	
15		805	20.26	38.1	
16		1,203	4.32	5.4	
17		1,394	4.43	4.8	
18		1,534	2.80	2.8	
19		1,652	3.78	3.5	
20		3,698	3.91	1.6	
21		4,053	2.80	1.0	
22		4,763	2.55	0.8	
23		5,403	2.79	0.8	
24		8,832	3.75	0.6	
25	▶	10,380	75.00	10.9	Upper Marker
26		12,445	0.00	0.0	

 © Copyright 2003 - 2009 Agilent Technologies, Inc.

Assay Class: High Sensitivity DNA Assay
Data Path: C:\...gh Sensitivity DNA Assay_DE72901621_2018-03-16_14-53-45.xad
Created: 3/16/2018 2:53:45 PM
Modified: 3/16/2018 3:34:15 PM

Electropherogram Summary Continued ...

... Peak table for sample 2 : Casper Clean 4

Peak	Size [bp]	Conc. [pg/µl]	Molarity [pmol/l]	Observations
27	13,336	0.00	0.0	

Region table for sample 2 : Casper Clean 4

From [bp]	To [bp]	Corr. Area	% of Total	Average Size [bp]	Size distribution in CV [%]	Conc. [pg/µl]	Molarity [pmol/l]	Co lor
200	1,000	1,657.8	90	477	30.4	1,240.71	4,454.4	

© Copyright 2003 - 2009 Agilent Technologies, Inc.

Assay Class:	High Sensitivity DNA Assay	Created:	3/16/2018 2:53:45 PM
Data Path:	C:\...gh Sensitivity DNA Assay_DE72901621_2018-03-16_14-53-45.xad	Modified:	3/16/2018 3:34:15 PM

Electropherogram Summary Continued ...

Casper Clean 5

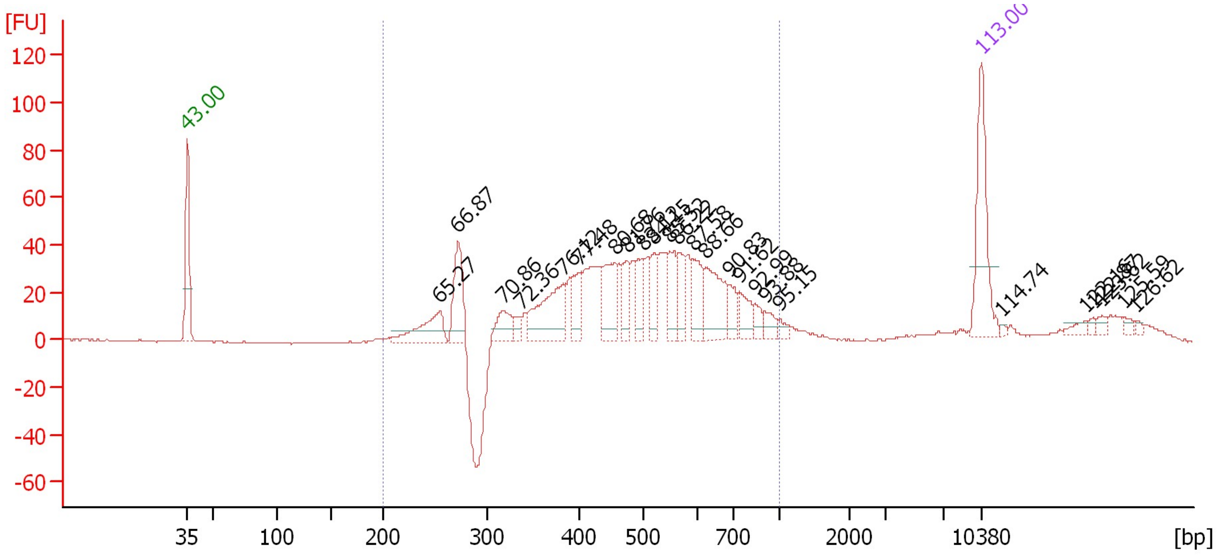

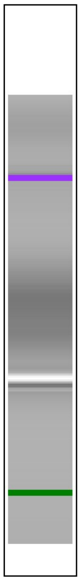

Overall Results for sample 3 : <u>Casper Clean 5</u>

Number of peaks found:	25	Corr. Area 1:	872.4
Noise:	0.3		

Peak table for sample 3 : <u>Casper Clean 5</u>

Peak		Size [bp]	Conc. [pg/µl]	Molarity [pmol/l]	Observations
1	◀	35	125.00	5,411.3	Lower Marker
2		254	60.05	358.1	
3		271	69.91	390.2	
4		317	31.82	152.0	
5		336	10.18	45.9	
6		383	87.48	346.2	
7		400	28.82	109.2	
8		456	53.20	176.9	
9		474	27.94	89.2	
10		498	28.48	86.6	
11		520	29.14	84.9	
12		549	34.67	95.7	
13		564	29.42	79.1	
14		593	35.52	90.8	
15		624	62.77	152.4	
16		693	20.49	44.8	
17		741	24.08	49.2	
18		839	11.82	21.3	
19		903	12.55	21.0	
20		995	6.65	10.1	
21	▶	10,380	75.00	10.9	Upper Marker
22		12,167	0.00	0.0	
23		19,800	0.00	0.0	
24		20,525	0.00	0.0	
25		21,298	0.00	0.0	
26		23,327	0.00	0.0	

 © Copyright 2003 - 2009 Agilent Technologies, Inc.

Assay Class: High Sensitivity DNA Assay
Data Path: C:\...gh Sensitivity DNA Assay_DE72901621_2018-03-16_14-53-45.xad
Created: 3/16/2018 2:53:45 PM
Modified: 3/16/2018 3:34:15 PM

Electropherogram Summary Continued ...

... Peak table for sample 3 : Casper Clean 5

Peak	Size [bp]	Conc. [pg/µl]	Molarity [pmol/l]	Observations
27	24,389	0.00	0.0	

Region table for sample 3 : Casper Clean 5

From [bp]	To [bp]	Corr. Area	% of Total	Average Size [bp]	Size distribution in CV [%]	Conc. [pg/µl]	Molarity [pmol/l]	Co lor
200	1,000	872.4	88	501	30.8	901.76	3,168.7	

 © Copyright 2003 - 2009 Agilent Technologies, Inc.

Assay Class: High Sensitivity DNA Assay
Data Path: C:\...gh Sensitivity DNA Assay_DE72901621_2018-03-16_14-53-45.xad

Created: 3/16/2018 2:53:45 PM
Modified: 3/16/2018 3:34:15 PM

Electropherogram Summary Continued ...

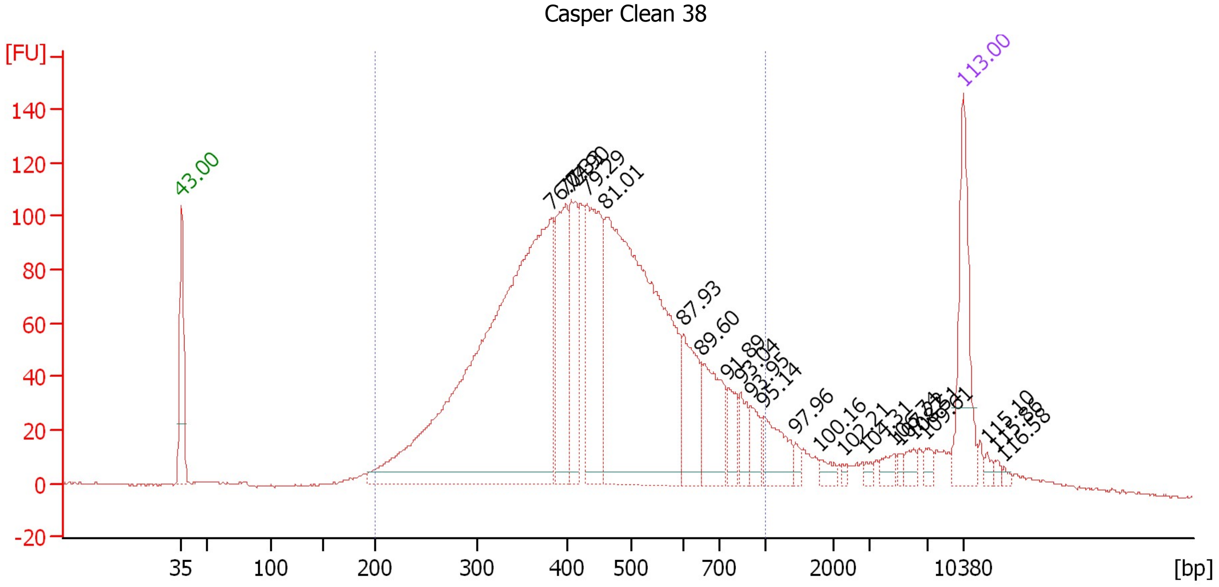

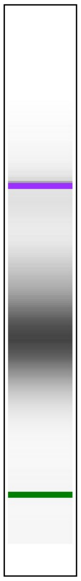

Overall Results for sample 4 : Casper Clean 38

Number of peaks found: 22
Corr. Area 1: 2,752.6
Noise: 0.4

Peak table for sample 4 : Casper Clean 38

Peak		Size [bp]	Conc. [pg/µl]	Molarity [pmol/l]	Observations
1	◀	35	125.00	5,411.3	Lower Marker
2		382	786.04	3,118.4	
3		398	123.88	471.6	
4		407	95.63	355.8	
5		431	168.59	592.2	
6		461	531.19	1,744.8	
7		601	70.22	177.1	
8		654	73.22	169.7	
9		760	27.58	55.0	
10		843	23.67	42.6	
11		908	18.93	31.6	
12		994	38.75	59.0	
13		1,441	5.76	6.1	
14		1,795	8.04	6.8	
15		2,249	2.64	1.8	
16		2,916	3.74	1.9	
17		4,690	9.02	2.9	
18		5,061	3.97	1.2	
19		6,065	7.89	2.0	
20		6,920	6.84	1.5	
21	▶	10,380	75.00	10.9	Upper Marker
22		12,540	0.00	0.0	
23		13,326	0.00	0.0	
24		14,062	0.00	0.0	

 © Copyright 2003 - 2009 Agilent Technologies, Inc.

Assay Class: High Sensitivity DNA Assay
Data Path: C:\...gh Sensitivity DNA Assay_DE72901621_2018-03-16_14-53-45.xad

Created: 3/16/2018 2:53:45 PM
Modified: 3/16/2018 3:34:15 PM

Electropherogram Summary Continued ...

... Region table for sample 4 : **Casper Clean 38**

From [bp]	To [bp]	Corr. Area	% of Total	Average Size [bp]	Size distribution in CV [%]	Conc. [pg/µl]	Molarity [pmol/l]	Co lor
200	1,000	2,752.6	89	457	32.7	2,144.15	8,145.4	

 © Copyright 2003 - 2009 Agilent Technologies, Inc.

Assay Class:	High Sensitivity DNA Assay	Created:	3/16/2018 2:53:45 PM
Data Path:	C:\...gh Sensitivity DNA Assay_DE72901621_2018-03-16_14-53-45.xad	Modified:	3/16/2018 3:34:15 PM

Electropherogram Summary Continued ...

Casper Clean 39

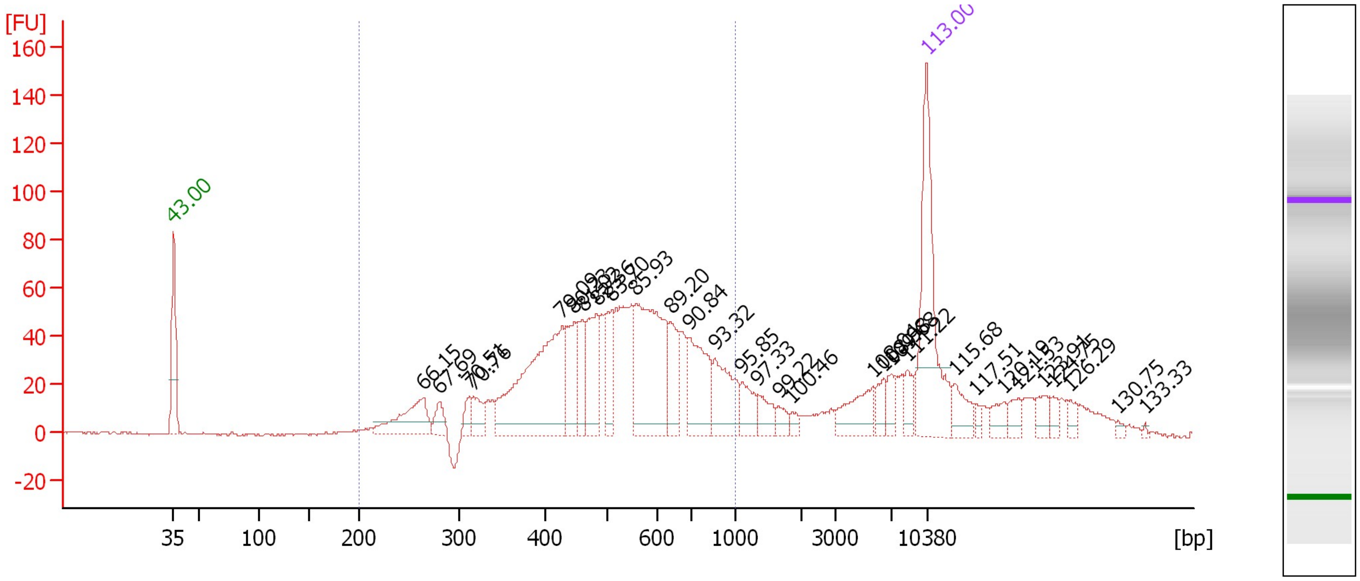

Overall Results for sample 5 : **Casper Clean 39**

Number of peaks found:	30	Corr. Area 1:	1,206.0
Noise:	0.4		

Peak table for sample 5 : **Casper Clean 39**

Peak		Size [bp]	Conc. [pg/µl]	Molarity [pmol/l]	Observations
1	◀	35	125.00	5,411.3	Lower Marker
2		264	45.05	258.9	
3		280	13.10	70.8	
4		313	8.53	41.3	
5		316	17.02	81.6	
6		428	136.40	483.0	
7		448	35.70	120.8	
8		462	24.95	81.9	
9		485	42.11	131.6	
10		510	27.48	81.6	
11		558	102.56	278.6	
12		641	29.43	69.6	
13		693	49.07	107.3	
14		863	37.22	65.3	
15		1,101	14.88	20.5	
16		1,340	11.73	13.3	
17		1,644	5.76	5.3	
18		1,844	4.01	3.3	
19		5,700	22.36	5.9	
20		6,433	8.87	2.1	
21		6,973	7.88	1.7	
22		8,545	9.37	1.7	
23	▶	10,380	75.00	10.9	Upper Marker
24		13,133	0.00	0.0	
25		15,019	0.00	0.0	
26		17,772	0.00	0.0	

 © Copyright 2003 - 2009 Agilent Technologies, Inc.

Assay Class: High Sensitivity DNA Assay
Data Path: C:\...gh Sensitivity DNA Assay_DE72901621_2018-03-16_14-53-45.xad
Created: 3/16/2018 2:53:45 PM
Modified: 3/16/2018 3:34:15 PM

Electropherogram Summary Continued ...

... Peak table for sample 5 : Casper Clean 39

Peak	Size [bp]	Conc. [pg/µl]	Molarity [pmol/l]	Observations
27	19,148	0.00	0.0	
28	21,595	0.00	0.0	
29	22,462	0.00	0.0	
30	24,042	0.00	0.0	
31	28,630	0.00	0.0	
32	31,281	0.00	0.0	

Region table for sample 5 : Casper Clean 39

From [bp]	To [bp]	Corr. Area	% of Total	Average Size [bp]	Size distribution in CV [%]	Conc. [pg/µl]	Molarity [pmol/l]	Co lor
200	1,000	1,206.0	73	529	30.6	675.56	2,235.1	

 © Copyright 2003 - 2009 Agilent Technologies, Inc.

Assay Class: High Sensitivity DNA Assay
Data Path: C:\...gh Sensitivity DNA Assay_DE72901621_2018-03-16_14-53-45.xad

Created: 3/16/2018 2:53:45 PM
Modified: 3/16/2018 3:34:15 PM

Electropherogram Summary Continued ...

Casper Clean 99

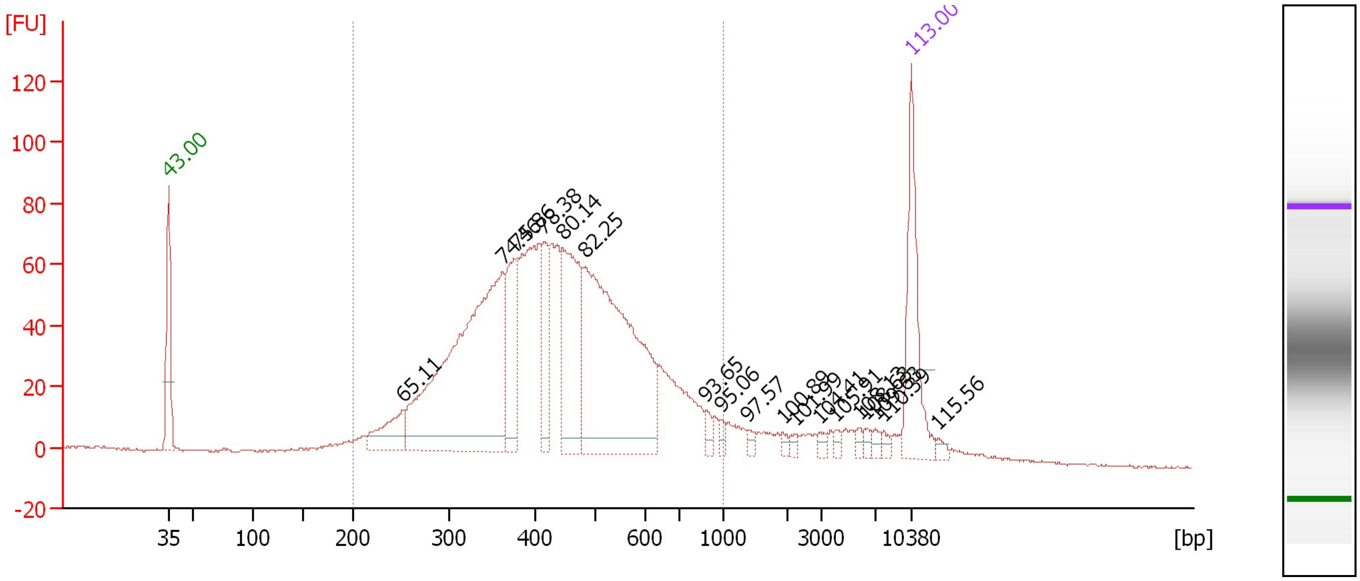

Overall Results for sample 6 : **Casper Clean 99**

Number of peaks found: 18   Corr. Area 1: 1,707.3

Noise: 0.4

Peak table for sample 6 : **Casper Clean 99**

Peak		Size [bp]	Conc. [pg/µl]	Molarity [pmol/l]	Observations
1	◀	35	125.00	5,411.3	Lower Marker
2		252	51.65	310.2	
3		362	416.56	1,742.9	
4		380	85.82	342.4	
5		415	54.64	199.3	
6		446	139.46	473.7	
7		483	353.90	1,110.5	
8		887	8.05	13.8	
9		988	5.83	8.9	
10		1,379	5.09	5.6	
11		1,914	3.86	3.1	
12		2,181	3.25	2.3	
13		2,947	4.52	2.3	
14		4,043	4.14	1.6	
15		5,764	4.03	1.1	
16		6,156	4.03	1.0	
17		6,938	4.72	1.0	
18		7,693	4.31	0.8	
19	▶	10,380	75.00	10.9	Upper Marker
20		13,015	0.00	0.0	

Region table for sample 6 : **Casper Clean 99**

From [bp]	To [bp]	Corr. Area	% of Total	Average Size [bp]	Size distribution in CV [%]	Conc. [pg/µl]	Molarity [pmol/l]	Co lor
200	1,000	1,707.3	90	451	31.0	1,534.71	5,839.9	■

 © Copyright 2003 - 2009 Agilent Technologies, Inc.

Assay Class:	High Sensitivity DNA Assay	Created:	3/16/2018 2:53:45 PM
Data Path:	C:\...gh Sensitivity DNA Assay_DE72901621_2018-03-16_14-53-45.xad	Modified:	3/16/2018 3:34:15 PM

Electropherogram Summary Continued ...

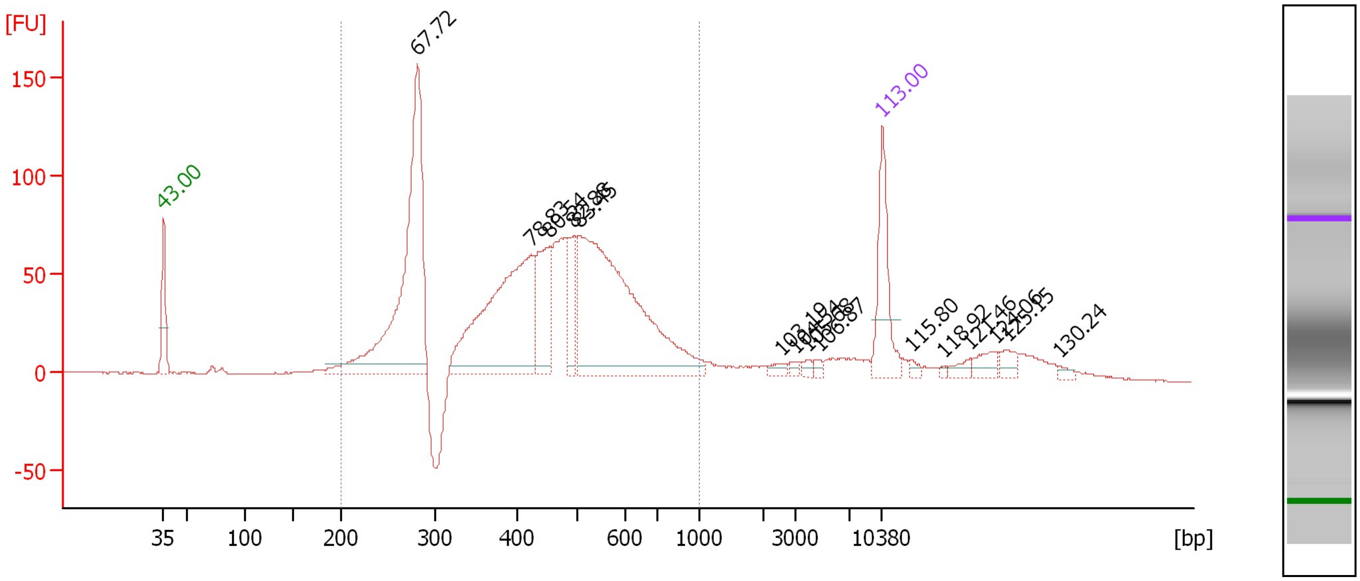

Overall Results for sample 7 : Casper Clean 101

Number of peaks found:	15	Corr. Area 1:	1,787.3
Noise:	0.4		

Peak table for sample 7 : Casper Clean 101

Peak	Size [bp]	Conc. [pg/µl]	Molarity [pmol/l]	Observations
1	35	125.00	5,411.3	Lower Marker
2	281	460.71	2,486.2	
3	423	332.09	1,188.6	
4	453	102.59	343.0	
5	494	51.39	157.7	
6	505	463.83	1,392.4	
7	2,559	7.21	4.3	
8	2,988	4.80	2.4	
9	3,859	5.54	2.2	
10	4,789	4.67	1.5	
11	10,380	75.00	10.9	Upper Marker
12	13,263	0.00	0.0	
13	16,467	0.00	0.0	
14	19,084	0.00	0.0	
15	21,754	0.00	0.0	
16	22,875	0.00	0.0	
17	28,108	0.00	0.0	

Region table for sample 7 : Casper Clean 101

From [bp]	To [bp]	Corr. Area	% of Total	Average Size [bp]	Size distribution in CV [%]	Conc. [pg/µl]	Molarity [pmol/l]	Co lor
200	1,000	1,787.3	86	449	32.6	1,544.05	6,133.7	

 © Copyright 2003 - 2009 Agilent Technologies, Inc.

Assay Class: High Sensitivity DNA Assay
Data Path: C:\...gh Sensitivity DNA Assay_DE72901621_2018-03-16_14-53-45.xad

Created: 3/16/2018 2:53:45 PM
Modified: 3/16/2018 3:34:15 PM

Electropherogram Summary Continued ...

Casper Clean 160

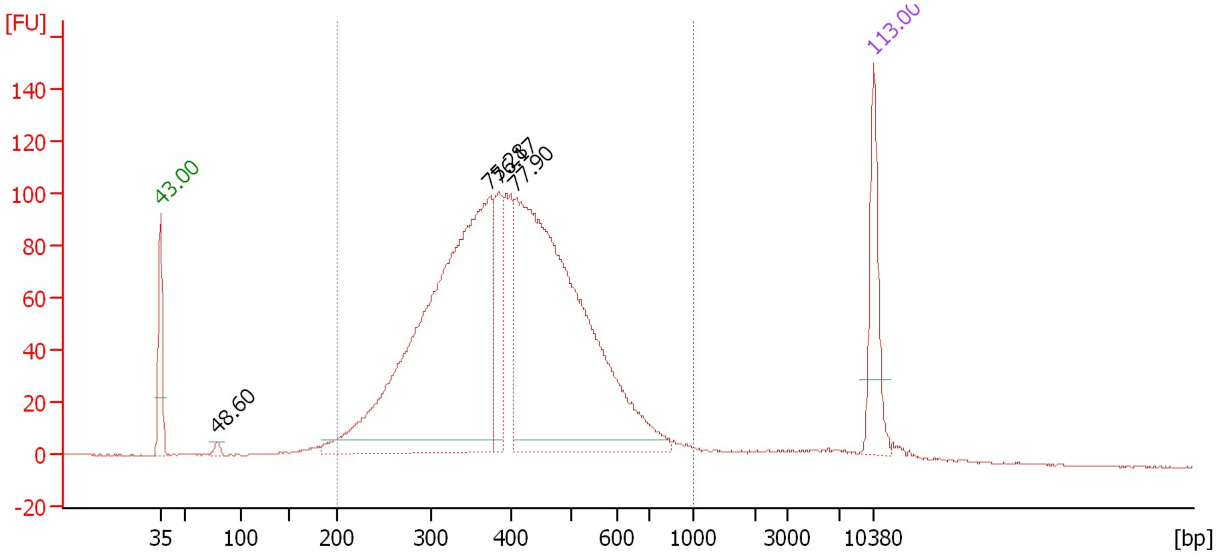

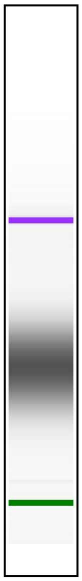

Overall Results for sample 8 : **Casper Clean 160**

Number of peaks found:	4	Corr. Area 1:	2,364.4
Noise:	0.4		

Peak table for sample 8 : **Casper Clean 160**

Peak		Size [bp]	Conc. [pg/µl]	Molarity [pmol/l]	Observations
1	◀	35	125.00	5,411.3	Lower Marker
2		79	9.26	176.9	
3		372	842.14	3,426.4	
4		383	102.04	403.2	
5		407	792.56	2,950.1	
6	▶	10,380	75.00	10.9	Upper Marker

Region table for sample 8 : **Casper Clean 160**

From [bp]	To [bp]	Corr. Area	% of Total	Average Size [bp]	Size distribution in CV [%]	Conc. [pg/µl]	Molarity [pmol/l]	Color
200	1,000	2,364.4	94	411	27.9	2,019.67	8,235.0	■

© Copyright 2003 - 2009 Agilent Technologies, Inc.

Assay Class: High Sensitivity DNA Assay
Data Path: C:\...gh Sensitivity DNA Assay_DE72901621_2018-03-16_14-53-45.xad
Created: 3/16/2018 2:53:45 PM
Modified: 3/16/2018 3:34:15 PM

Electropherogram Summary Continued ...

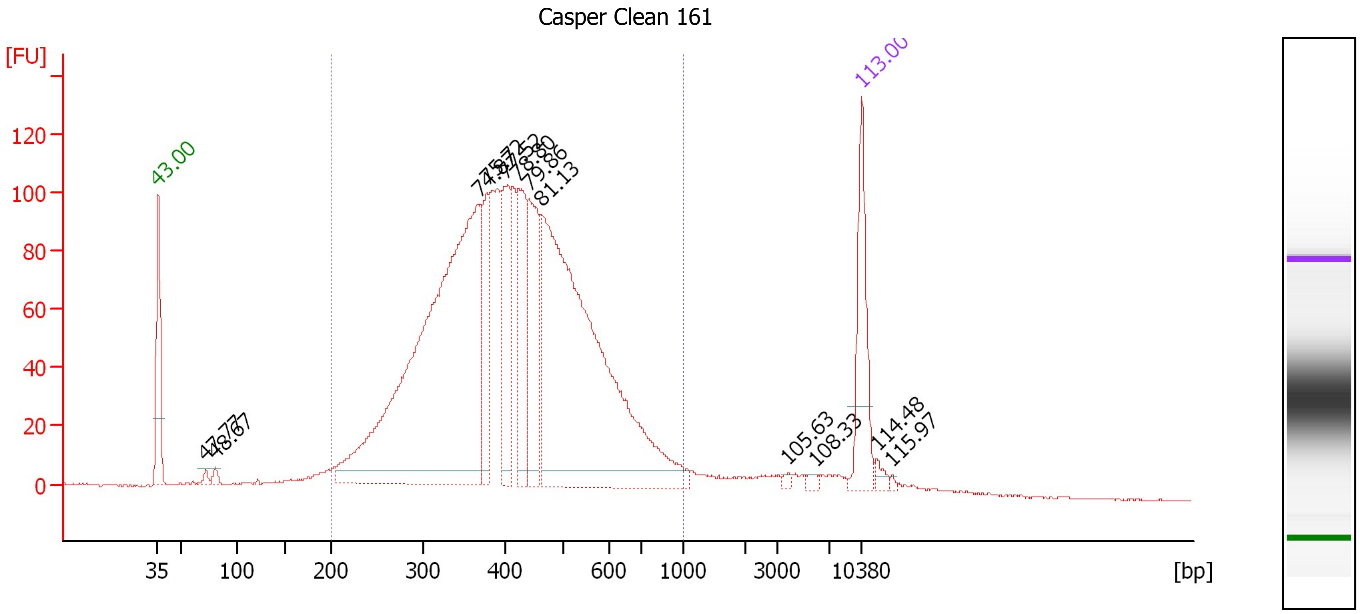

Overall Results for sample 9 : Casper Clean 161

Number of peaks found:	12	Corr. Area 1:	2,459.1
Noise:	0.3		

Peak table for sample 9 : Casper Clean 161

Peak		Size [bp]	Conc. [pg/µl]	Molarity [pmol/l]	Observations
1	◀	35	125.00	5,411.3	Lower Marker
2		72	7.64	160.9	
3		80	8.47	160.5	
4		367	799.98	3,300.1	
5		378	93.44	374.7	
6		401	123.15	465.8	
7		423	113.23	405.8	
8		441	134.47	461.8	
9		463	665.28	2,175.4	
10		3,821	3.08	1.2	
11		5,926	4.79	1.2	
12	▶	10,380	75.00	10.9	Upper Marker
13		11,907	0.00	0.0	
14		13,434	0.00	0.0	

Region table for sample 9 : Casper Clean 161

From [bp]	To [bp]	Corr. Area	% of Total	Average Size [bp]	Size distribution in CV [%]	Conc. [pg/µl]	Molarity [pmol/l]	Co lor
200	1,000	2,459.1	92	429	29.1	2,250.76	8,876.4	■

 © Copyright 2003 - 2009 Agilent Technologies, Inc.

Assay Class: High Sensitivity DNA Assay
Data Path: C:\...gh Sensitivity DNA Assay_DE72901621_2018-03-16_14-53-45.xad

Created: 3/16/2018 2:53:45 PM
Modified: 3/16/2018 3:34:15 PM

Electropherogram Summary Continued ...

Casper Clean 222

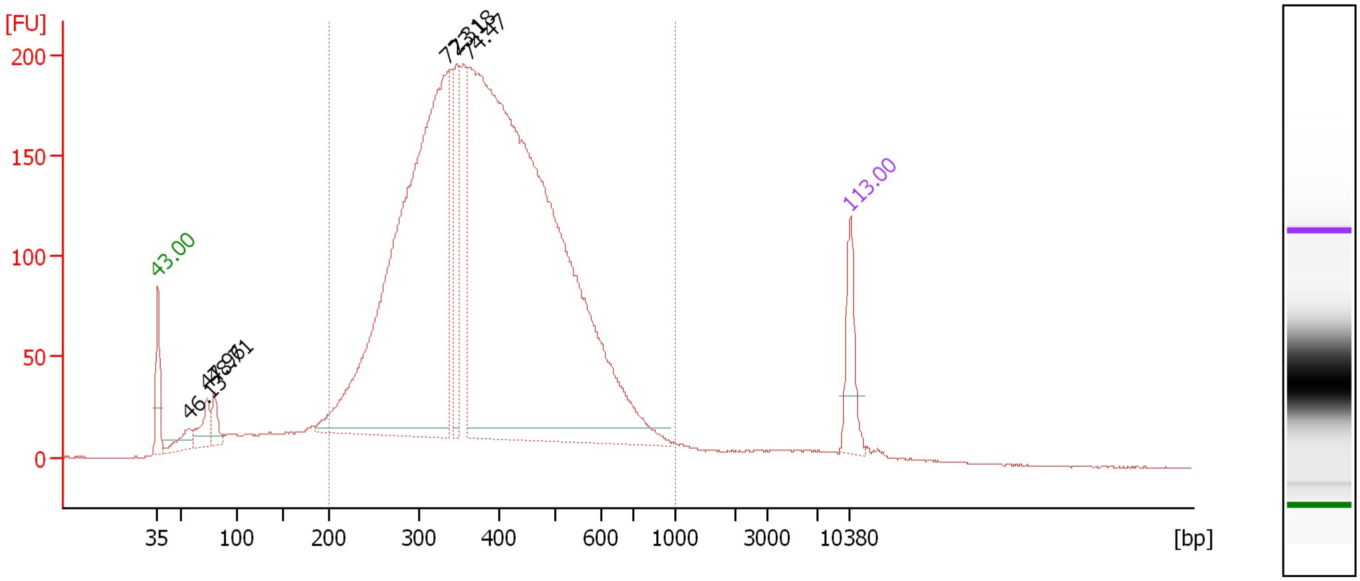

Overall Results for sample 10 : **<u>Casper Clean 222</u>**

Number of peaks found: 6

Corr. Area 1: 4,834.8

Noise: 0.4

Peak table for sample 10 : **<u>Casper Clean 222</u>**

Peak		Size [bp]	Conc. [pg/µl]	Molarity [pmol/l]	Observations
1	◀	35	125.00	5,411.3	Lower Marker
2		57	53.83	1,424.9	
3		74	74.68	1,537.7	
4		80	51.58	973.0	
5		335	1,628.32	7,356.4	
6		346	186.83	817.8	
7		362	2,608.31	10,908.4	
8	▶	10,380	75.00	10.9	Upper Marker

Region table for sample 10 : **<u>Casper Clean 222</u>**

From [bp]	To [bp]	Corr. Area	% of Total	Average Size [bp]	Size distribution in CV [%]	Conc. [pg/µl]	Molarity [pmol/l]	Color
200	1,000	4,834.8	89	398	29.4	5,116.53	21,699.2	■

 © Copyright 2003 - 2009 Agilent Technologies, Inc.

Assay Class: High Sensitivity DNA Assay
Data Path: C:\...gh Sensitivity DNA Assay_DE72901621_2018-03-16_14-53-45.xad

Created: 3/16/2018 2:53:45 PM
Modified: 3/16/2018 3:34:15 PM

Electropherogram Summary Continued ...

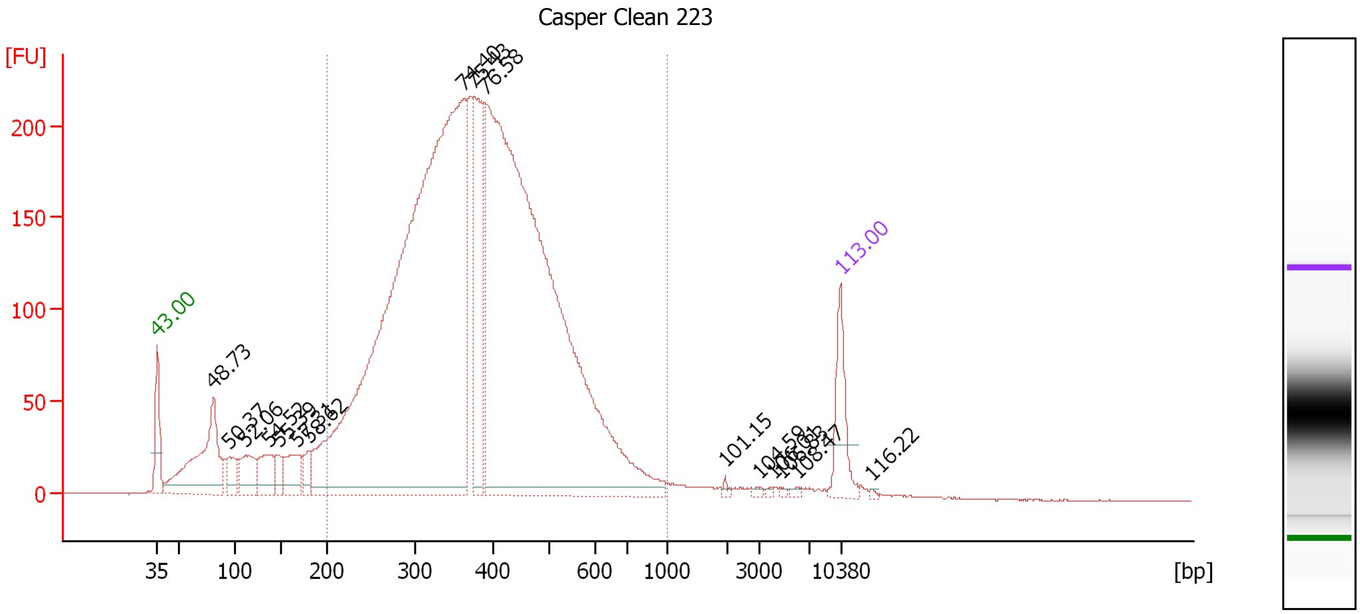

Overall Results for sample 11 : Casper Clean 223

Number of peaks found: 16 Corr. Area 1: 5,055.1

Noise: 0.3

Peak table for sample 11 : Casper Clean 223

Peak		Size [bp]	Conc. [pg/µl]	Molarity [pmol/l]	Observations
1	◀	35	125.00	5,411.3	Lower Marker
2		81	336.01	6,323.4	
3		95	56.87	905.8	
4		112	88.88	1,200.7	
5		138	88.21	967.4	
6		147	39.63	407.3	
7		168	86.80	783.6	
8		182	35.19	293.2	
9		361	2,345.87	9,835.9	
10		374	282.11	1,141.9	
11		389	2,092.27	8,157.1	
12		1,956	4.21	3.3	
13		3,013	4.39	2.2	
14		4,118	3.19	1.2	
15		4,756	2.93	0.9	
16		6,031	4.32	1.1	
17	▶	10,380	75.00	10.9	Upper Marker
18		13,693	0.00	0.0	

Region table for sample 11 : Casper Clean 223

From [bp]	To [bp]	Corr. Area	% of Total	Average Size [bp]	Size distribution in CV [%]	Conc. [pg/µl]	Molarity [pmol/l]	Co lor
200	1,000	5,055.1	87	392	28.0	5,031.84	21,517.5	■

 © Copyright 2003 - 2009 Agilent Technologies, Inc.

Assay Class: High Sensitivity DNA Assay
Data Path: C:\...gh Sensitivity DNA Assay_DE72901621_2018-03-16_14-53-45.xad

Created: 3/16/2018 2:53:45 PM
Modified: 3/16/2018 3:34:15 PM

Gel Image

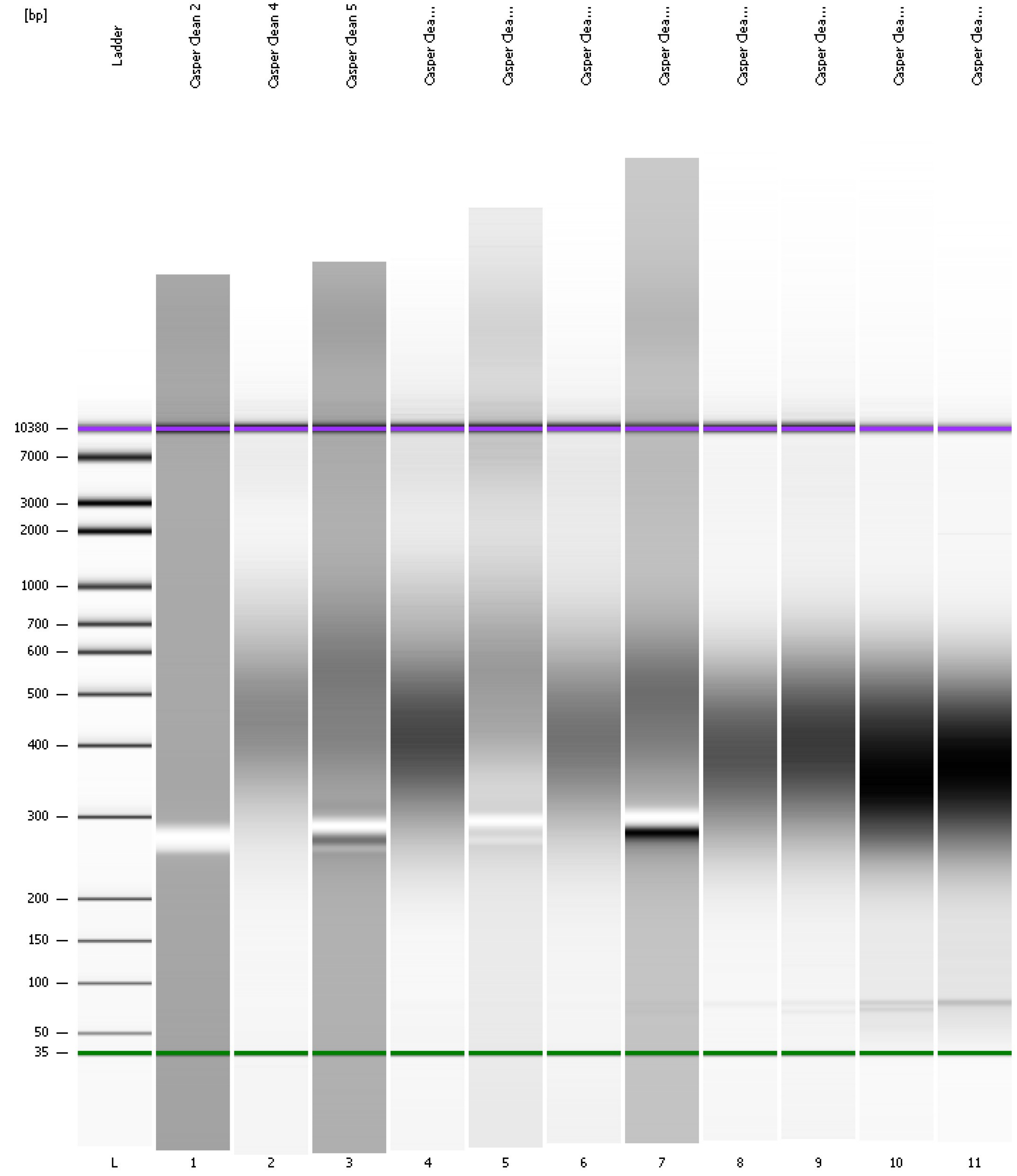

© Copyright 2003 - 2009 Agilent Technologies, Inc.

Assay Class: High Sensitivity DNA Assay
Data Path: C:\...gh Sensitivity DNA Assay_DE72901621_2018-03-16_14-53-45.xad

Created: 3/16/2018 2:53:45 PM
Modified: 3/16/2018 3:34:15 PM

Run Logbook

Description	Number	Source	Category	Sub Category	Time	Time Zone	User	Host
Run ended on port 1 (Number of wells acquired: 12)		Instrument	Run		3/16/2018 3:34:12 PM	(GMT +01:00) Romance Standard Time	Lab	W-LAB02
Run started on port 1 (File: C:\Program Files\Agilent\2100 bioanalyzer\2100 expert\Data\2018-03-16\2100 expert_High Sensitivity DNA Assay_DE72901621_2018-03-16_14-53-45.xad)		Instrument	Run		3/16/2018 2:53:51 PM	(GMT +01:00) Romance Standard Time	Lab	W-LAB02
Product Number : G2939A		Instrument	Run		3/16/2018 2:53:51 PM	(GMT +01:00) Romance Standard Time	Lab	W-LAB02
Name :		Instrument	Run		3/16/2018 2:53:51 PM	(GMT +01:00) Romance Standard Time	Lab	W-LAB02
Vendor : Agilent Technologies		Instrument	Run		3/16/2018 2:53:51 PM	(GMT +01:00) Romance Standard Time	Lab	W-LAB02
Serial# : DE72901621		Instrument	Run		3/16/2018 2:53:51 PM	(GMT +01:00) Romance Standard Time	Lab	W-LAB02
Firmware : C.01.069		Instrument	Run		3/16/2018 2:53:51 PM	(GMT +01:00) Romance Standard Time	Lab	W-LAB02
Cartridge : Electrode		Instrument	Run		3/16/2018 2:53:50 PM	(GMT +01:00) Romance Standard Time	Lab	W-LAB02

 © Copyright 2003 - 2009 Agilent Technologies, Inc.

File S7. R markdown file containing the R analysis.

Analysis: Experiments on the effect of sequencing platform and library preparation

Casper Sahl Poulsen

08092021

Contents

Introduction . . . 1
Packages . . . 2
Read in data . . . 3
Change naming . . . 3
Analysis . . . 3
Quality control of different sequencing specific parameters . . . 3
PCA . . . 24
PCA Fig_2 . . . 29
Heatmaps . . . 35
Boxplots of distances between samples . . . 49
sPLS-DA . . . 92
Multivariate ANOVA based on dissimilarities (Adonis) . . . 111
Constrained ordination with redundancy analysis (rda) . . . 123
Additional . . . 144
Session information . . . 144
This document was processed on: . . . 145

Introduction

The purpose of the following code is to assess the effect of library preparation using KAPA (PCR free), NEXTflex (PCR free), and Nextera (PCR step), and Illumina sequencing platforms (HiSeq and NextSeq) on two different microbial communities. Raw data is available online (Include the ENA reference). Samples have been quality processed and mapped with MGmapper extracting read counts mapping to the different genera.

Explanation of samples naming in the taxonomy table
DTU: Performed at the Technical university of Denmark followed by the year of sampling. LPSX: Part of the study on library preparation and sequencing platform study. HX: Part of the handling experiment study included here to represent one library preparation sequencing platform method. P1 & P2: Pig feces 1 and 2. S1 and S2: Sewage 1 and 2. KA: Kapa library preparation. NF: NEXTflex library preparation. NX: Nextera library preparation (Can both be NX1 and NX2 representing the same process to investigate variance associated with redoing library preparation and sequencing). HI: HiSeq. NS: NextSeq. 0h: Direct processing after sample collection, samples were not stored. 64h_80C: After sample collection alliquouts of the same sample as the one processed directly were stored at -80?C for 64 h. a, b, c: Indicating storage replicates, only duplicates where included in this study. MG_XXX: Internal metagenomics sample number.

Metadata
Contains information on how the samples were processed, sequencing performance, and info on mapping to different databases

Feature Contains taxonomic feature information

###Analysis notes All analysis adhere to the compositional data analysis framework by including an isometric log ratio transformation (ILR) to calculate Euclidean distances used to perform prinicipal component analysis (PCA), Heatmap sample clustering and boxplots. A centered log ratio transformation (CLR) were used when keeping genera information was important in sparse partial least square discriminant analysis (sPLS-DA) and redundancy analysis (rda). Important factors to be aware of in this analysis is that raw data is used, filtered according to an average count of 5, and zeroes are estimated with simple multiplicative replacement.

Colors In study design and heatmaps KAPA is purple (#984ea3), Nextera is orange (#ff7f00), and NEXTflex is red (#e41a1c). When performing sPLS-DA, PCA, rda the NEXTflex is run either with NextSeq is blue (#377eb8) or HiSeq is green (#4daf4a). Finally in PCA and rda Nextera is divided in 1 and 2, where 1 remains orange and 2 is brown (#993300)
. . .

Packages

```
#Install knitr package
#install.packages("knitr")

#Update bioconductor
#if (!requireNamespace("BiocManager"))
#    install.packages("BiocManager")
#BiocManager::install()

#install.packages("ggplot2")
library(ggplot2) #Data visualization, based on grammar of graphics.
  #help(package="ggplot2")
#install.packages("ggthemes")
library(ggthemes) #Extra themes, scales and geoms for ggplot2. help(package="ggthemes")
#install.packages("vegan")
library(vegan) #Community ecology package, ordination methods, diversity analysis
  #and other functions for community and vegetation ecologists. help(package="vegan")
library(gridExtra)
#install.packages("reshape2")
library(reshape2)
#install.packages("tidyr")
library(tidyr)
#install.packages("knitr")
library(knitr)
#install.packages("stringr")
library(stringr)
#install.packages("cowplot")
library(cowplot)
#install.packages("compositions")
library(compositions) #The package provides functions for the consistent analysis of
  #compositional data (e.g. portions of substances) and positive numbers
  #(e.g. concentrations) in the way proposed by Aitchison and Pawlowsky-Glahn.
  #Includes the clr function. help(package="compositions")
#install.packages("zCompositions")
library(zCompositions) #Include cmultRepl function to estimate zeroes
#install.packages("car")
library(car) # Companion to applied regression includes Levene's test. help(package="car")
#install.packages("robCompositions")
library(robCompositions) #help(package="robCompositions")
#install.packages("psych") #describeBy function
```

```
library(psych)

#if (!requireNamespace("BiocManager", quietly = TRUE))
#    install.packages("BiocManager")
#BiocManager::install("mixOmics", version = "3.8")
#install.packages("mixOmics") #Extends upon some of the function from robCompositions
library(mixOmics)
#install.packages("pheatmap")
library(pheatmap) # Implementation of heatmaps that offers more control over dimensions
  #and appearance help(package="pheatmap")
#install.packages("RColorBrewer")
library(RColorBrewer) #Used to make the Heatmap colors
#install.packages("FSA")
library(FSA) #A variety of simple fish stock assessment methods. Includes the Dunne
  #non-parametric follow-up test help(package="FSA")
#install.packages("Rmisc")
library(Rmisc)
#install.packages("dplyr")
library(dplyr) #A grammar of data manipulation. A fast, consistent tool for working with
  #data frame like objects. help(package="dplyr")
#install.packages("gridExtra")
library(amap) #Calculating spearman correlations
```

Read in data

```
Tax <- read.delim(file="../File_S2/TaxonomyRaw20180925.txt", check.names=FALSE,
                  stringsAsFactors=FALSE, strip.white=TRUE)
Metadata <- read.delim(file="../File_S2/Metadata20180925.txt", check.names=FALSE,
                       stringsAsFactors=FALSE, strip.white=TRUE)
Feature <- read.delim(file="../File_S2/FeatureShort20180925.txt", check.names=FALSE,
                      stringsAsFactors=FALSE, strip.white=TRUE)
```

Change naming

```
colnames(Metadata)[14] <- "Storage"
```

Analysis

Chunks can be run by themselves or consecutively

Quality control of different sequencing specific parameters

Include QC on sequencing output and alpha-diversity summarized in S1_Table and rarefaction curves S2_Fig

```
rm(list=setdiff(ls(), c("Metadata", "Feature", "Tax")))

#Subset data pig feces 1 and 2 (PF)
Subset<- "All" #All, Allspiked, Allunspiked, PF, SW, P1, P1spiked, P1unspiked, P2,
  #P2spiked, P2unspiked, S1, S1spiked, S1unspiked, S2, S2spiked, S2unspiked

#Subset experiment, Experiment_Type. (Meaningfull combinations of subset and SubExp:
  #HX=All, FTX=P1&S1, LTX=P1&S1, LPSX=All unspiked, HXFTX=P1&S1, HXLTX=P1&S1,
  #HXLPSX=All unspiked, HXLTXLPSX=P1&S1 unspiked)
```

```r
SubExp<-"LPSX" #HX, FTX, LTX, LPSX, HXFTX, HXLTX, HXLPSX, HXLTXLPSX, HXFTXLTX, All

#Subset Frozen Unfrozen
SubFre<-"Both" #Frozen, Unfrozen, Both

#Removing negative and positive controls
Metadata2<-dplyr::filter(Metadata, Sample_type_simple=="Sample")

#Subsetting Metadata2
if (Subset=="Allspiked") {
  Metadata2<-filter(Metadata2, SpikedUnspiked == "Spiked")
} else if (Subset=="Allunspiked") {
  Metadata2<-filter(Metadata2, SpikedUnspiked == "Unspiked")
} else if (Subset=="PF") {
  Metadata2<-filter(Metadata2, Experiment == "Pig_feces_1" | Experiment == "Pig_feces_2")
} else if (Subset=="PFspiked") {
  Metadata2<-filter(Metadata2, Sample_type == "Pig_feces_1_spiked" |
                    Sample_type == "Pig_feces_2_spiked")
} else if (Subset=="PFunspiked") {
  Metadata2<-filter(Metadata2, Sample_type == "Pig_feces_1" |
                    Sample_type == "Pig_feces_2")
} else if (Subset=="SW") {
  Metadata2<-filter(Metadata2, Experiment == "Sewage_1" | Experiment == "Sewage_2")
} else if (Subset=="SWspiked") {
  Metadata2<-filter(Metadata2, Sample_type == "Sewage_1_spiked" |
                    Sample_type == "Sewage_2_spiked")
} else if (Subset=="SWunspiked") {
  Metadata2<-filter(Metadata2, Sample_type == "Sewage_1" | Sample_type == "Sewage_2")
} else if (Subset=="P1") {
  Metadata2<-filter(Metadata2, Experiment == "Pig_feces_1")
} else if (Subset=="P1spiked") {
  Metadata2<-filter(Metadata2, Sample_type == "Pig_feces_1_spiked")
} else if (Subset=="P1unspiked") {
  Metadata2<-filter(Metadata2, Sample_type == "Pig_feces_1")
} else if (Subset=="P2") {
  Metadata2<-filter(Metadata2, Experiment == "Pig_feces_2")
} else if (Subset=="P2spiked") {
  Metadata2<-filter(Metadata2, Sample_type == "Pig_feces_2_spiked")
} else if (Subset=="P2unspiked") {
  Metadata2<-filter(Metadata2, Sample_type == "Pig_feces_2")
} else if (Subset=="S1") {
  Metadata2<-filter(Metadata2, Experiment == "Sewage_1")
} else if (Subset=="S1spiked") {
  Metadata2<-filter(Metadata2, Sample_type == "Sewage_1_spiked")
} else if (Subset=="S1unspiked") {
  Metadata2<-filter(Metadata2, Sample_type == "Sewage_1")
} else if (Subset=="S2") {
  Metadata2<-filter(Metadata2, Experiment == "Sewage_2")
} else if (Subset=="S2spiked") {
  Metadata2<-filter(Metadata2, Sample_type == "Sewage_2_spiked")
} else if (Subset=="S2unspiked") {
  Metadata2<-filter(Metadata2, Sample_type == "Sewage_2")
} else if (Subset=="All") {
```

```
  print("No subsetting, all included")
} else {
  print("Subset defined not valid")
}
```

```
## [1] "No subsetting, all included"
```

```
#Further subsetting Metadata2
if (SubExp=="HX") {
  Metadata2<-filter(Metadata2, Experiment_type == "Handling_experiment")
} else if (SubExp=="FTX") {
  Metadata2<-filter(Metadata2, Experiment_type == "Freeze_thaw_experiment")
} else if (SubExp=="LTX") {
  Metadata2<-filter(Metadata2, Experiment_type == "Long_term_storage_experiment")
} else if (SubExp=="LPSX") {
  Metadata22<-filter(Metadata2, Experiment_type == "Library_prep_seq_platform_experiment")
  VectorLPSX <- unique(Metadata22$Matching_samples)
  Metadata2<-filter(Metadata2, Matching_samples %in% VectorLPSX)
  rm(VectorLPSX, Metadata22)
} else if (SubExp=="HXFTX") {
  Metadata2<-filter(Metadata2, Experiment_type == "Handling_experiment" |
                    Experiment_type == "Freeze_thaw_experiment")
} else if (SubExp=="HXLTX") {
  Metadata2<-filter(Metadata2, Experiment_type == "Handling_experiment" |
                    Experiment_type == "Long_term_storage_experiment")
} else if (SubExp=="HXLPSX") {
  Metadata2<-filter(Metadata2, Experiment_type == "Handling_experiment" |
                    Experiment_type == "Library_prep_seq_platform_experiment")
} else if (SubExp=="HXLTXLPSX") {
  Metadata2<-filter(Metadata2, Experiment_type == "Handling_experiment" |
                    Experiment_type == "Long_term_storage_experiment" |
                    Experiment_type == "Library_prep_seq_platform_experiment")
} else if (SubExp=="HXFTXLTX") {
  Metadata2<-filter(Metadata2, Experiment_type == "Handling_experiment" |
                    Experiment_type == "Freeze_thaw_experiment" |
                    Experiment_type == "Long_term_storage_experiment")
} else if (SubExp=="All") {
  print("No subsetting, all included")
} else {
  print("Subset defined not valid")
}

#Further subsetting Metadata2
if (SubFre=="Frozen") {
  Metadata2<-filter(Metadata2, FrozenUnfrozenSimple == "Freezer")
} else if (SubFre=="Unfrozen") {
  Metadata2<-filter(Metadata2, FrozenUnfrozenSimple == "Unfrozen")
} else if (SubFre=="Both") {
  print("No subsetting, all included")
} else {
  print("Subset defined not valid")
}
```

```
## [1] "No subsetting, all included"
```

```r
#Removing the Kappa NextSeq run
Metadata2<-Metadata2[grep("\\KANS.*", Metadata2$Sample, invert=TRUE),]

#Applying subsetting to OTU tables
Tax2<-dplyr::select(Tax, one_of(Metadata2$Sample))

#Remove orgs that are not present after subsetting.
Tax2 <- Tax2[rowSums(Tax2)>0,]

#Simple summary statistics
summary(Metadata2)
```

```
##     Sample           Sample_name        Sample_number        Number
##  Length:80          Length:80          Min.   :  4.00   Min.   :  4.0
##  Class :character   Class :character   1st Qu.: 57.75   1st Qu.:359.8
##  Mode  :character   Mode  :character   Median :113.00   Median :382.5
##                                        Mean   :112.94   Mean   :336.0
##                                        3rd Qu.:167.50   3rd Qu.:405.2
##                                        Max.   :223.00   Max.   :428.0
##
##  Seq_sample_name_SameassampleFormergingin R Experiment_type
##  Length:80                               Length:80
##  Class :character                        Class :character
##  Mode  :character                        Mode  :character
##
##
##
##
##   Experiment           Colors          Sample_type        Sample_type_simple
##  Length:80          Length:80          Length:80          Length:80
##  Class :character   Class :character   Class :character   Class :character
##  Mode  :character   Mode  :character   Mode  :character   Mode  :character
##
##
##
##
##  Sample_type_new    Sample_type_RF          Time       Storage
##  Length:80          Length:80          Min.   : 0   Min.   :-80
##  Class :character   Class :character   1st Qu.: 0   1st Qu.:-80
##  Mode  :character   Mode  :character   Median :32   Median :-80
##                                        Mean   :32   Mean   :-80
##                                        3rd Qu.:64   3rd Qu.:-80
##                                        Max.   :64   Max.   :-80
##                                                     NA's   :40
##  FrozenUnfrozenSimple FrozenUnfrozen     StoragePlacementType
##  Length:80            Length:80          Length:80
##  Class :character     Class :character   Class :character
##  Mode  :character     Mode  :character   Mode  :character
##
##
##
##
##   Replicate          Flow_cell_lane   Concentration_DTU  ConcentrationNum_DTU
##  Length:80          Min.   : 1.000   Length:80          Length:80
```

```
##  Class :character   1st Qu.: 9.000   Class :character   Class :character  
##  Mode  :character   Median :10.000   Mode  :character   Mode  :character  
##                     Mean   : 9.275                                        
##                     3rd Qu.:11.000                                        
##                     Max.   :12.000                                        
##                                                                           
##  Date_of_collection Freeze_thaw_cycles Temperature_sampling_day Precipitation
##  Length:80          Min.   :0.0        Min.   : 7.00            Min.   : 0.0
##  Class :character   1st Qu.:0.0        1st Qu.:10.75            1st Qu.: 0.0
##  Mode  :character   Median :0.5        Median :13.50            Median : 0.0
##                     Mean   :0.5        Mean   :12.75            Mean   : 3.5
##                     3rd Qu.:1.0        3rd Qu.:15.50            3rd Qu.: 3.5
##                     Max.   :1.0        Max.   :17.00            Max.   :14.0
##                                                                           
##  Precipitation_the_day_before Replicate_Groups   SpikedUnspiked    
##  Length:80                    Length:80          Length:80         
##  Class :character             Class :character   Class :character  
##  Mode  :character             Mode  :character   Mode  :character  
##                                                                    
##                                                                    
##                                                                    
##                                                                    
##  Replicate_Boxplot  Matching_samples   Library_preparation Sequencing_platform
##  Length:80          Length:80          Length:80           Length:80          
##  Class :character   Class :character   Class :character    Class :character   
##  Mode  :character   Mode  :character   Mode  :character    Mode  :character   
##                                                                               
##                                                                               
##                                                                               
##                                                                               
##   Reads_Total        Bases_Total        Reads_AfterTrim    Bases_AfterTrim    
##  Min.   : 2710623   Min.   :4.083e+08   Min.   : 2594599   Min.   :3.643e+08  
##  1st Qu.: 8454797   1st Qu.:1.272e+09   1st Qu.: 7628501   1st Qu.:1.047e+09  
##  Median : 9458488   Median :1.420e+09   Median : 8867728   Median :1.232e+09  
##  Mean   :10702254   Mean   :1.597e+09   Mean   : 9744984   Mean   :1.334e+09  
##  3rd Qu.:12032974   3rd Qu.:1.772e+09   3rd Qu.:11203132   3rd Qu.:1.516e+09  
##  Max.   :30764150   Max.   :4.645e+09   Max.   :27349015   Max.   :3.526e+09  
##                                                                               
##     Archaea           Bacteria       Bacteria_draft    Common_animals  
##  Min.   :    6.0   Min.   :  66553   Min.   : 378113   Min.   :     0  
##  1st Qu.:  394.8   1st Qu.: 177899   1st Qu.: 663736   1st Qu.:     1  
##  Median : 5690.0   Median : 421225   Median :1056719   Median :   354  
##  Mean   : 6259.7   Mean   : 680137   Mean   :1249109   Mean   : 26519  
##  3rd Qu.: 8473.5   3rd Qu.:1048261   3rd Qu.:1573516   3rd Qu.: 23313  
##  Max.   :31029.0   Max.   :2529946   Max.   :3738745   Max.   :284238  
##                                                                        
##  Common_plants        Fungi           Human          HumanMicrobiome 
##  Min.   :   1.0   Min.   :   14   Min.   :    0.0   Min.   : 21956  
##  1st Qu.:  46.0   1st Qu.:  121   1st Qu.:    0.0   1st Qu.: 37859  
##  Median : 168.5   Median : 2685   Median :   72.5   Median :102841  
##  Mean   : 637.3   Mean   : 3711   Mean   : 6061.8   Mean   :190112  
##  3rd Qu.: 984.0   3rd Qu.: 6579   3rd Qu.:11423.0   3rd Qu.:291209  
##  Max.   :5672.0   Max.   :16380   Max.   :61495.0   Max.   :856979  
##                                                                     
```

```
##  Parasites_other    Parasites_vertebrates   ResFinder        Unmapped
##  Min.   :    1.00   Min.   : 1471          Min.   :  457   Min.   : 1496139
##  1st Qu.:    8.75   1st Qu.: 5808          1st Qu.: 1748   1st Qu.: 5832961
##  Median : 5253.50   Median : 8175          Median : 3013   Median : 6833336
##  Mean   :19597.78   Mean   : 9912          Mean   : 3585   Mean   : 7515692
##  3rd Qu.:36720.25   3rd Qu.:12636          3rd Qu.: 4619   3rd Qu.: 8058677
##  Max.   :86532.00   Max.   :35914          Max.   :13107   Max.   :24250760
##
##    Virulence VirulenceFactors      Virus              Mapped
##  Min.   :0   Min.   :   0.00   Min.   :    0.0   Min.   : 519830
##  1st Qu.:0   1st Qu.:   1.75   1st Qu.:  222.8   1st Qu.: 992124
##  Median :0   Median : 647.50   Median :  823.0   Median :1710584
##  Mean   :0   Mean   : 854.94   Mean   : 2620.8   Mean   :2194676
##  3rd Qu.:0   3rd Qu.:1468.00   3rd Qu.: 3505.2   3rd Qu.:2960078
##  Max.   :0   Max.   :3506.00   Max.   :14298.0   Max.   :7243363
##
##   MappedParsed      ReadsNotPhiX      PercentUnmapped  PercentPhiX
##  Min.   : 486505   Min.   : 2594567   Min.   :51.67   Min.   :0.0003660
##  1st Qu.: 977481   1st Qu.: 7628373   1st Qu.:66.91   1st Qu.:0.0008363
##  Median :1633466   Median : 8841916   Median :80.25   Median :0.0011655
##  Mean   :2161458   Mean   : 9710368   Mean   :76.45   Mean   :0.2824303
##  3rd Qu.:2925106   3rd Qu.:11178469   3rd Qu.:88.84   3rd Qu.:0.0018101
##  Max.   :7243155   Max.   :27087990   Max.   :91.65   Max.   :2.3724866
##
```

```
#Using the psych library to create summary for specific parameters. Used to create S1 Table.
#Make column that combine lib prep and seq platform
Metadata2$LPSP<-paste(Metadata2$Library_preparation, Metadata2$Sequencing_platform,
                      sep="_") #Be aware that Nextera 1 & 2 are analyzed as one group.
Metadata3 <- dplyr::select(Metadata2, one_of(c("LPSP", "Reads_Total", "Reads_AfterTrim",
                                               "Mapped", "PercentUnmapped")))
describeBy(Metadata3, Metadata3$LPSP)
```

```
##
##  Descriptive statistics by group
## group: Kappa_Hiseq
##                 vars  n        mean         sd     median    trimmed        mad
## LPSP*              1 16        1.00       0.00       1.00        1.0       0.00
## Reads_Total        2 16 10593744.12 2827116.71 9429034.50 10310564.4 2223801.41
## Reads_AfterTrim    3 16  9569830.19 2795390.14 8203492.00  9293024.4 1946476.63
## Mapped             4 16  2090665.31 1211173.94 1851270.50  2028484.7 1484352.43
## PercentUnmapped    5 16       77.58      13.09      80.57       78.1      12.88
##                        min         max      range  skew kurtosis        se
## LPSP*                 1.00        1.00       0.00   NaN      NaN      0.00
## Reads_Total     7770085.00 17381919.00 9611834.00  1.08     0.06 706779.18
## Reads_AfterTrim 6880135.00 16134806.00 9254671.00  1.04    -0.09 698847.53
## Mapped           723943.00  4327916.00 3603973.00  0.42    -1.23 302793.49
## PercentUnmapped      56.72       91.08      34.37 -0.43    -1.54      3.27
## ------------------------------------------------------------
## group: Nextera_Nextseq
##                 vars  n        mean         sd      median     trimmed
## LPSP*              1 32        1.00       0.00        1.00        1.00
## Reads_Total        2 32 10568702.84 2171725.13 10159631.50 10540565.77
## Reads_AfterTrim    3 32  9776944.94 2023848.62  9385629.00  9744943.23
## Mapped             4 32  2592690.12 1902833.80  2078232.00  2411158.19
```

```
## PercentUnmapped    5 32       75.36      14.66       78.79       76.22
##                         mad        min         max       range  skew kurtosis
## LPSP*                  0.00       1.00        1.00        0.00   NaN      NaN
## Reads_Total     2231840.06 6497278.00 16510194.00 10012916.00  0.34     0.04
## Reads_AfterTrim 2143004.90 6022970.00 15338228.00  9315258.00  0.37     0.03
## Mapped          1681296.57  519830.00  7243363.00  6723533.00  0.73    -0.76
## PercentUnmapped      14.55      51.67       91.42       39.75 -0.40    -1.50
##                        se
## LPSP*                0.00
## Reads_Total     383910.39
## Reads_AfterTrim 357769.27
## Mapped          336376.67
## PercentUnmapped      2.59
## ------------------------------------------------------------
## group: Nextflex_Hiseq
##                 vars  n        mean         sd     median     trimmed
## LPSP*              1 16        1.00       0.00        1.0        1.00
## Reads_Total        2 16 14893656.44 8208573.04 12104649.5 14374253.43
## Reads_AfterTrim    3 16 13036491.12 7295407.53 10620802.0 12565742.64
## Mapped             4 16  2337912.56 1160028.02  2423194.0  2239130.71
## PercentUnmapped    5 16       78.07      13.05       80.9       78.59
##                         mad        min         max       range  skew kurtosis
## LPSP*                  0.00       1.00        1.00        0.00   NaN      NaN
## Reads_Total     5170282.10 6294805.00 30764150.00 24469345.00  0.85    -0.83
## Reads_AfterTrim 4614286.34 5314446.00 27349015.00 22034569.00  0.85    -0.82
## Mapped          1056514.84  820592.00  5238179.00  4417587.00  0.66     0.00
## PercentUnmapped      12.94      57.23       91.65       34.42 -0.42    -1.54
##                         se
## LPSP*                 0.00
## Reads_Total     2052143.26
## Reads_AfterTrim 1823851.88
## Mapped           290007.01
## PercentUnmapped       3.26
## ------------------------------------------------------------
## group: Nextflex_Nextseq
##                 vars  n      mean         sd     median    trimmed        mad
## LPSP*              1 16       1.0       0.00       1.00       1.00       0.00
## Reads_Total        2 16 6886462.2 2308294.12 7630467.00 7002553.29 2633588.34
## Reads_AfterTrim    3 16 6564709.8 2194564.68 7266381.50 6671380.29 2450715.56
## Mapped             4 16 1359420.0  637118.33 1085288.00 1322755.93  333316.65
## PercentUnmapped    5 16      75.9      14.49      78.91      76.55      14.67
##                         min        max      range  skew kurtosis        se
## LPSP*                  1.00       1.00       0.00   NaN      NaN      0.00
## Reads_Total      2710623.00 9437027.00 6726404.00 -0.33    -1.57 577073.53
## Reads_AfterTrim  2594599.00 9041433.00 6446834.00 -0.33    -1.57 548641.17
## Mapped            679273.00 2552864.00 1873591.00  0.79    -1.05 159279.58
## PercentUnmapped       52.01      90.79      38.78 -0.42    -1.53      3.62
```

```
Metadata3 <- dplyr::select(Metadata2, one_of(c("Experiment", "Reads_Total",
                                               "Reads_AfterTrim", "Mapped",
                                               "PercentUnmapped")))
describeBy(Metadata3, Metadata3$Experiment)
```

```
##
##  Descriptive statistics by group
```

```
## group: Pig_feces_1
##                 vars  n        mean         sd     median     trimmed
## Experiment*        1 20        1.00       0.00       1.00        1.00
## Reads_Total        2 20 10778441.00 4670236.87 9430178.50 10009687.56
## Reads_AfterTrim    3 20  9802157.00 4099187.25 8571205.00  9098167.44
## Mapped             4 20  1206140.90  539057.43 1041951.50  1111624.25
## PercentUnmapped    5 20       87.73       1.23      87.76       87.72
##                        mad        min         max      range skew kurtosis
## Experiment*           0.00       1.00        1.00        0.0  NaN      NaN
## Reads_Total     2230748.87 6176773.00 26738368.00 20561595.0 1.97     4.09
## Reads_AfterTrim 2129123.31 5903434.00 23594456.00 17691022.0 1.90     3.69
## Mapped           321251.99  679273.00  3007972.00  2328699.0 1.87     3.59
## PercentUnmapped       1.48      85.84       89.74        3.9 0.05    -1.53
##                         se
## Experiment*           0.00
## Reads_Total     1044296.71
## Reads_AfterTrim  916606.13
## Mapped           120536.91
## PercentUnmapped       0.28
## ------------------------------------------------------------
## group: Pig_feces_2
##                 vars  n        mean         sd     median     trimmed
## Experiment*        1 20        1.00       0.00       1.00        1.00
## Reads_Total        2 20 12509469.65 7126520.82 9406461.00 10925461.44
## Reads_AfterTrim    3 20 11336395.85 6178439.00 8736953.50 10001248.31
## Mapped             4 20  1134489.50  624486.61  873942.00  1001051.88
## PercentUnmapped    5 20       89.96       1.15      90.23       90.01
##                        mad        min         max       range  skew kurtosis
## Experiment*           0.00       1.00        1.00        0.00   NaN      NaN
## Reads_Total     1267688.23 6497278.00 30764150.00 24266872.00  1.66     1.35
## Reads_AfterTrim 1418566.51 6022970.00 27349015.00 21326045.00  1.64     1.30
## Mapped           217198.68  519830.00  2837230.00  2317400.00  1.62     1.58
## PercentUnmapped       1.34      87.92       91.65        3.73 -0.21    -1.39
##                         se
## Experiment*           0.00
## Reads_Total     1593538.50
## Reads_AfterTrim 1381540.96
## Mapped           139639.45
## PercentUnmapped       0.26
## ------------------------------------------------------------
## group: Sewage_1
##                 vars  n       mean         sd     median    trimmed        mad
## Experiment*        1 20       1.00       0.00       1.00       1.00       0.00
## Reads_Total        2 20 9482881.20 2420426.96 9893242.00 9679118.81 2027319.84
## Reads_AfterTrim    3 20 8685531.75 2194112.26 9106574.00 8873042.19 1692583.60
## Mapped             4 20 2445230.20  653288.97 2694864.00 2496994.31  468380.03
## PercentUnmapped    5 20      71.82       1.93      71.44      71.84       2.72
##                        min         max      range  skew kurtosis        se
## Experiment*           1.00        1.00       0.00   NaN      NaN      0.00
## Reads_Total     4237170.00 13149234.00 8912064.00 -0.55    -0.39 541223.92
## Reads_AfterTrim 4026411.00 11680638.00 7654227.00 -0.63    -0.57 490618.42
## Mapped          1127982.00  3306581.00 2178599.00 -0.70    -0.85 146079.85
## PercentUnmapped      68.81       74.67       5.86 -0.07    -1.42      0.43
## ------------------------------------------------------------
```

```
## group: Sewage_2
##                 vars  n       mean         sd      median     trimmed
## Experiment*        1 20        1.0       0.00        1.00        1.00
## Reads_Total        2 20 10038222.9 3817314.50 11071639.00 10129225.06
## Reads_AfterTrim    3 20  9155852.2 3511934.45 10083087.50  9228667.19
## Mapped             4 20  3992841.9 1593867.50  4211995.00  4005999.12
## PercentUnmapped    5 20       56.3       3.01       56.48       56.29
##                        mad        min         max       range  skew kurtosis
## Experiment*           0.00       1.00        1.00        0.00   NaN      NaN
## Reads_Total     2929857.78 2710623.00 16510194.00 13799571.00 -0.32    -0.95
## Reads_AfterTrim 3082931.04 2594599.00 15338228.00 12743629.00 -0.25    -1.08
## Mapped          1652136.05 1098428.00  7243363.00  6144935.00 -0.04    -0.95
## PercentUnmapped       3.39      51.67       61.21        9.54 -0.08    -1.23
##                        se
## Experiment*          0.00
## Reads_Total     853577.47
## Reads_AfterTrim 785292.42
## Mapped          356399.61
## PercentUnmapped      0.67
```

```
#Combining both LPSP and Experiment
Metadata2$QCperf<-paste(Metadata2$LPSP, Metadata2$Experiment, sep="_")
Metadata3 <- dplyr::select(Metadata2, one_of(c("QCperf", "Reads_Total",
                                               "Reads_AfterTrim", "Mapped",
                                               "PercentUnmapped")))
describeBy(Metadata3, Metadata3$QCperf)
```

```
##
##  Descriptive statistics by group
## group: Kappa_Hiseq_Pig_feces_1
##                 vars n        mean        sd      median     trimmed        mad
## QCperf*            1 4        1.00       0.0        1.00        1.00       0.00
## Reads_Total        2 4 11651621.25 3461471.8 11254465.50 11651621.25 3550220.62
## Reads_AfterTrim    3 4 10560302.00 3568012.5 10196601.00 10560302.00 3793518.98
## Mapped             4 4  1309658.50  571317.0  1257220.50  1309658.50  634166.58
## PercentUnmapped    5 4       87.92       1.4       87.92       87.92       1.79
##                        min         max      range skew kurtosis        se
## QCperf*               1.00        1.00       0.00  NaN      NaN       0.0
## Reads_Total     8239800.00 15857754.00 7617954.00 0.16    -2.17 1730735.9
## Reads_AfterTrim 7065998.00 14782008.00 7716010.00 0.13    -2.22 1784006.3
## Mapped           774764.00  1949429.00 1174665.00 0.09    -2.31  285658.5
## PercentUnmapped      86.62       89.23       2.61 0.00    -2.42       0.7
## ------------------------------------------------------------
## group: Kappa_Hiseq_Pig_feces_2
##                 vars n       mean         sd     median    trimmed        mad
## QCperf*            1 4        1.0       0.00       1.00        1.0       0.00
## Reads_Total        2 4 10844635.8 4401150.04 9057953.00 10844635.8 1110024.10
## Reads_AfterTrim    3 4  9711395.5 4314942.75 7904844.00  9711395.5  960783.36
## Mapped             4 4   950466.2  389276.21  773461.50   950466.2   68226.29
## PercentUnmapped    5 4       90.1       0.85      90.01       90.1       0.91
##                        min         max     range skew kurtosis         se
## QCperf*               1.00        1.00       0.0  NaN      NaN       0.00
## Reads_Total     7880718.00 17381919.00 9501201.0 0.71    -1.72 2200575.02
## Reads_AfterTrim 6901088.00 16134806.00 9233718.0 0.72    -1.71 2157471.38
## Mapped           723943.00  1530999.00  807056.0 0.72    -1.71  194638.10
```

```
## PercentUnmapped      89.28       91.08       1.8 0.12    -2.25       0.42
## ------------------------------------------------------------
## group: Kappa_Hiseq_Sewage_1
##                 vars n       mean         sd     median    trimmed        mad
## QCperf*            1 4       1.00       0.00       1.00       1.00       0.00
## Reads_Total        2 4 9657517.25 1736074.40 9749314.50 9657517.25 2033616.44
## Reads_AfterTrim    3 4 8866168.25 1671035.05 9137876.00 8866168.25 1646175.26
## Mapped             4 4 2342512.25  472515.07 2435971.00 2342512.25  427269.75
## PercentUnmapped    5 4      73.63       0.67      73.48      73.63       0.53
##                       min         max      range  skew kurtosis        se
## QCperf*              1.00        1.00       0.00   NaN      NaN      0.00
## Reads_Total     7770085.00 11361355.00 3591270.00 -0.05    -2.33 868037.20
## Reads_AfterTrim 6880135.00 10308786.00 3428651.00 -0.16    -2.25 835517.52
## Mapped          1753112.00  2744995.00  991883.00 -0.23    -2.17 236257.54
## PercentUnmapped      73.06       74.52       1.46  0.35    -2.04      0.33
## ------------------------------------------------------------
## group: Kappa_Hiseq_Sewage_2
##                 vars n        mean         sd      median     trimmed
## QCperf*            1 4        1.00       0.00        1.00        1.00
## Reads_Total        2 4 10221202.25 1633177.93 10271664.50 10221202.25
## Reads_AfterTrim    3 4  9141455.00 1640000.09  9216352.00  9141455.00
## Mapped             4 4  3760024.25  535306.49  3746326.50  3760024.25
## PercentUnmapped    5 4       58.65       1.73       58.66       58.65
##                       mad        min         max      range  skew kurtosis
## QCperf*              0.00       1.00        1.00       0.00   NaN      NaN
## Reads_Total     1982074.60 8512911.00 11828569.00 3315658.00 -0.03    -2.36
## Reads_AfterTrim 1964324.91 7438475.00 10694641.00 3256166.00 -0.03    -2.37
## Mapped           649783.55 3219528.00  4327916.00 1108388.00  0.02    -2.33
## PercentUnmapped       2.07      56.72       60.57       3.85 -0.01    -2.19
##                       se
## QCperf*             0.00
## Reads_Total     816588.96
## Reads_AfterTrim 820000.04
## Mapped          267653.25
## PercentUnmapped      0.86
## ------------------------------------------------------------
## group: Nextera_Nextseq_Pig_feces_1
##                 vars n       mean         sd     median    trimmed        mad
## QCperf*            1 8       1.00       0.00       1.00       1.00       0.00
## Reads_Total        2 8 9457361.88 2298440.95 8938264.00 9457361.88 1501436.43
## Reads_AfterTrim    3 8 8740719.12 2120294.27 8190030.50 8740719.12 1318636.30
## Mapped             4 8 1102437.00  287114.11 1045559.50 1102437.00  321251.99
## PercentUnmapped    5 8      87.42       1.07      87.44      87.42       1.39
##                       min         max      range  skew kurtosis        se
## QCperf*              1.00        1.00       0.00   NaN      NaN      0.00
## Reads_Total     6640924.00 13364493.00 6723569.00  0.52    -1.35 812621.59
## Reads_AfterTrim 6192563.00 12326109.00 6133546.00  0.53    -1.37 749637.23
## Mapped           700156.00  1499122.00  798966.00  0.14    -1.59 101510.17
## PercentUnmapped      86.06       88.69       2.63 -0.03    -1.97      0.38
## ------------------------------------------------------------
## group: Nextera_Nextseq_Pig_feces_2
##                 vars n       mean         sd     median    trimmed        mad
## QCperf*            1 8       1.00       0.00       1.00       1.00       0.00
## Reads_Total        2 8 9458107.38 1713168.81 9157529.50 9458107.38 1108069.29
```

```
## Reads_AfterTrim    3 8 8760619.62 1598181.40 8491885.00 8760619.62 1040381.93
## Mapped             4 8  920752.38  281463.43  885319.00  920752.38  266939.16
## PercentUnmapped    5 8      89.69       1.46      89.72      89.69       2.01
##                        min         max      range  skew kurtosis        se
## QCperf*               1.00        1.00       0.00   NaN      NaN      0.00
## Reads_Total     6497278.00 12178541.00 5681263.00 -0.05    -0.98 605696.64
## Reads_AfterTrim 6022970.00 11295881.00 5272911.00 -0.03    -1.01 565042.45
## Mapped           519830.00  1335944.00  816114.00  0.14    -1.58  99512.35
## PercentUnmapped      87.92       91.42       3.49  0.02    -2.02      0.52
## ------------------------------------------------------------
## group: Nextera_Nextseq_Sewage_1
##                 vars n        mean         sd      median     trimmed       mad
## QCperf*            1 8        1.00       0.00        1.00        1.00      0.00
## Reads_Total        2 8 10519889.38 1009113.05 10156389.00 10519889.38 729330.23
## Reads_AfterTrim    3 8  9701089.88  930914.52  9376177.50  9701089.88 646300.92
## Mapped             4 8  2897175.50  263040.45  2768608.50  2897175.50 138119.02
## PercentUnmapped    5 8       70.11       1.14       69.96       70.11      1.48
##                        min         max      range skew kurtosis        se
## QCperf*               1.00        1.00       0.00  NaN      NaN      0.00
## Reads_Total     9403237.00 12542662.00 3139425.00 0.83    -0.70 356775.34
## Reads_AfterTrim 8720989.00 11604674.00 2883685.00 0.90    -0.57 329127.99
## Mapped          2657342.00  3306581.00  649239.00 0.55    -1.67  92998.84
## PercentUnmapped      68.81       71.51       2.69 0.08    -2.06      0.40
## ------------------------------------------------------------
## group: Nextera_Nextseq_Sewage_2
##                 vars n        mean         sd      median     trimmed
## QCperf*            1 8        1.00       0.00        1.00        1.00
## Reads_Total        2 8 12839452.75 1712890.98 12706266.00 12839452.75
## Reads_AfterTrim    3 8 11905351.12 1622143.30 11805688.00 11905351.12
## Mapped             4 8  5450395.62  795053.24  5332496.00  5450395.62
## PercentUnmapped    5 8       54.21       2.05       54.48       54.21
##                        mad         min         max      range  skew kurtosis
## QCperf*               0.00        1.00        1.00       0.00   NaN      NaN
## Reads_Total     1007403.72 10754736.00 16510194.00 5755458.00  0.95    -0.05
## Reads_AfterTrim  917257.93  9778274.00 15338228.00 5559954.00  0.86    -0.09
## Mapped           445674.75  4725786.00  7243363.00 2517577.00  1.25     0.39
## PercentUnmapped       2.51       51.67       56.24       4.57 -0.10    -2.10
##                        se
## QCperf*              0.00
## Reads_Total     605598.41
## Reads_AfterTrim 573514.26
## Mapped          281093.77
## PercentUnmapped      0.72
## ------------------------------------------------------------
## group: Nextflex_Hiseq_Pig_feces_1
##                 vars n        mean         sd      median     trimmed       mad
## QCperf*            1 4        1.00       0.00        1.00        1.00       0.0
## Reads_Total        2 4 15621734.75 7884993.68 13184081.50 15621734.75 4643123.7
## Reads_AfterTrim    3 4 13628208.00 7066040.60 11261099.50 13628208.00 3930482.3
## Mapped             4 4  1596917.25  967465.87  1239669.00  1596917.25  396462.8
## PercentUnmapped    5 4       88.44       1.34       88.45       88.44       1.7
##                        min         max       range skew kurtosis         se
## QCperf*               1.00        1.00         0.0  NaN      NaN       0.00
## Reads_Total     9380408.00 26738368.00 17357960.0 0.52    -1.88 3942496.84
```

```
## Reads_AfterTrim 8396177.00 23594456.00 15198279.0 0.52    -1.88 3533020.30
## Mapped           900359.00  3007972.00  2107613.0 0.63    -1.77  483732.94
## PercentUnmapped      87.13       89.74        2.6 0.00    -2.40       0.67
## ------------------------------------------------------------
## group: Nextflex_Hiseq_Pig_feces_2
##                 vars n        mean         sd      median     trimmed
## QCperf*            1 4        1.00       0.00        1.00        1.00
## Reads_Total        2 4 23770332.75 9017670.84 26435597.50 23770332.75
## Reads_AfterTrim    3 4 20861852.50 8043898.59 23089103.50 20861852.50
## Mapped             4 4  2005047.75  925004.42  2181184.50  2005047.75
## PercentUnmapped    5 4       90.54       0.98       90.49       90.54
##                       mad         min         max       range  skew kurtosis
## QCperf*              0.00        1.00        1.00        0.00   NaN      NaN
## Reads_Total     6028858.72 11445986.00 30764150.00 19318164.00 -0.43    -1.97
## Reads_AfterTrim 5638089.84  9920188.00 27349015.00 17428827.00 -0.42    -1.98
## Mapped           821714.00   820592.00  2837230.00  2016638.00 -0.27    -2.11
## PercentUnmapped       1.12       89.53       91.65        2.13  0.07    -2.23
##                        se
## QCperf*              0.00
## Reads_Total     4508835.42
## Reads_AfterTrim 4021949.30
## Mapped           462502.21
## PercentUnmapped       0.49
## ------------------------------------------------------------
## group: Nextflex_Hiseq_Sewage_1
##                 vars n        mean         sd      median     trimmed
## QCperf*            1 4        1.00       0.00        1.00        1.00
## Reads_Total        2 4 10102549.00 3310976.59 10178643.50 10102549.00
## Reads_AfterTrim    3 4  8855355.25 3079809.85  8987149.00  8855355.25
## Mapped             4 4  2264576.50  818886.31  2322481.50  2264576.50
## PercentUnmapped    5 4       74.05       0.54       74.08       74.05
##                       mad        min         max      range  skew kurtosis
## QCperf*              0.00       1.00        1.00       0.00   NaN      NaN
## Reads_Total     4118114.24 6903675.00 13149234.00 6245559.00 -0.01    -2.42
## Reads_AfterTrim 3727075.52 5766485.00 11680638.00 5914153.00 -0.02    -2.40
## Mapped           956465.29 1430673.00  2982670.00 1551997.00 -0.03    -2.40
## PercentUnmapped       0.46      73.36       74.67       1.31 -0.14    -1.87
##                        se
## QCperf*              0.00
## Reads_Total     1655488.30
## Reads_AfterTrim 1539904.92
## Mapped           409443.16
## PercentUnmapped       0.27
## ------------------------------------------------------------
## group: Nextflex_Hiseq_Sewage_2
##                 vars n        mean         sd    median     trimmed        mad
## QCperf*            1 4        1.00       0.00       1.0        1.00       0.00
## Reads_Total        2 4 10080009.25 3896249.17 9242607.0 10080009.25 2194594.93
## Reads_AfterTrim    3 4  8800548.75 3556361.41 8059941.5  8800548.75 2107428.43
## Mapped             4 4  3485108.75 1274511.58 3244742.0  3485108.75  899142.79
## PercentUnmapped    5 4       59.26       1.94      59.3       59.26       2.38
##                        min         max      range  skew kurtosis         se
## QCperf*               1.00        1.00       0.00   NaN      NaN       0.00
## Reads_Total     6294805.00 15540018.00 9245213.00  0.45    -1.81 1948124.58
```

```
## Reads_AfterTrim 5314446.00 13767866.00 8453420.00  0.44    -1.82 1778180.71
## Mapped          2212772.00  5238179.00 3025407.00  0.39    -1.85  637255.79
## PercentUnmapped      57.23       61.21       3.98 -0.02    -2.35       0.97
## ------------------------------------------------------------
## group: Nextflex_Nextseq_Pig_feces_1
##                 vars n       mean         sd     median    trimmed        mad
## QCperf*            1 4       1.00       0.00       1.00       1.00       0.00
## Reads_Total        2 4 7704125.25 1451339.96 7630467.00 7704125.25 1644849.07
## Reads_AfterTrim    3 4 7340836.75 1380281.27 7266381.50 7340836.75 1565567.78
## Mapped             4 4  919254.75  171186.87  962799.00  919254.75  110503.37
## PercentUnmapped    5 4      87.43       1.43      87.56      87.43       1.59
##                       min        max      range  skew kurtosis        se
## QCperf*              1.00       1.00       0.00   NaN      NaN      0.00
## Reads_Total     6176773.00 9378794.00 3202021.00  0.07    -2.21 725669.98
## Reads_AfterTrim 5903434.00 8927150.00 3023716.00  0.07    -2.22 690140.64
## Mapped           679273.00 1072148.00  392875.00 -0.48    -1.86  85593.43
## PercentUnmapped      85.84      88.77       2.93 -0.08    -2.32      0.71
## ------------------------------------------------------------
## group: Nextflex_Nextseq_Pig_feces_2
##                 vars n       mean        sd     median    trimmed      mad
## QCperf*            1 4       1.00      0.00       1.00       1.00      0.0
## Reads_Total        2 4 9016165.00 494799.79 9076559.00 9016165.00 532780.5
## Reads_AfterTrim    3 4 8587492.00 461154.61 8610059.50 8587492.00 543296.6
## Mapped             4 4  875428.75 104506.11  873942.00  875428.75 123471.7
## PercentUnmapped    5 4      89.81      1.02      89.81      89.81      1.3
##                       min        max     range  skew kurtosis        se
## QCperf*              1.00       1.00      0.00   NaN      NaN      0.00
## Reads_Total     8474515.00 9437027.00 962512.00 -0.08    -2.35 247399.90
## Reads_AfterTrim 8088416.00 9041433.00 953017.00 -0.05    -2.33 230577.30
## Mapped           760471.00  993360.00 232889.00  0.02    -2.20  52253.05
## PercentUnmapped      88.84      90.79      1.95  0.00    -2.41      0.51
## ------------------------------------------------------------
## group: Nextflex_Nextseq_Sewage_1
##                 vars n       mean         sd     median    trimmed        mad
## QCperf*            1 4       1.00       0.00       1.00       1.00       0.00
## Reads_Total        2 4 6614561.00 2522106.72 6616340.00 6614561.00 3221977.42
## Reads_AfterTrim    3 4 6303955.50 2407287.79 6310983.00 6303955.50 3066912.29
## Mapped             4 4 1824711.25  729409.98 1808999.50 1824711.25  904035.37
## PercentUnmapped    5 4      71.21       0.74      71.33      71.21       0.49
##                      min        max      range  skew kurtosis         se
## QCperf*              1.0       1.00       0.00   NaN      NaN       0.00
## Reads_Total     4237170.0 8988394.00 4751224.00  0.00    -2.42 1261053.36
## Reads_AfterTrim 4026411.0 8567445.00 4541034.00  0.00    -2.42 1203643.89
## Mapped          1127982.0 2552864.00 1424882.00  0.01    -2.39  364704.99
## PercentUnmapped      70.2      71.99       1.78 -0.35    -1.84       0.37
## ------------------------------------------------------------
## group: Nextflex_Nextseq_Sewage_2
##                 vars n       mean        sd     median    trimmed        mad
## QCperf*            1 4       1.00       0.0       1.00       1.00       0.00
## Reads_Total        2 4 4210997.75 1160693.4 4385553.00 4210997.75 1057270.97
## Reads_AfterTrim    3 4 4026554.75 1108468.9 4196444.00 4026554.75 1008951.55
## Mapped             4 4 1818285.25  563268.8 1859071.00 1818285.25  522223.61
## PercentUnmapped    5 4      55.16       2.9      55.48      55.16       3.18
##                       min        max      range  skew kurtosis        se
```

```
## QCperf*                 1.00       1.00       0.00   NaN      NaN       0.00
## Reads_Total      2710623.00 5362262.00 2651639.00 -0.26    -2.04 580346.68
## Reads_AfterTrim 2594599.00 5118732.00 2524133.00 -0.26    -2.04 554234.43
## Mapped           1098428.00 2456571.00 1358143.00 -0.15    -1.92 281634.41
## PercentUnmapped       52.01      57.66       5.66 -0.07    -2.36       1.45
```

```
#Create rarefaction curves
#Adding colors to the rarecurves according to experiment, P1=#663300 (blue),
  #P2=#FF9900 (red), S1=#006600 (forestgreen), S2=#33FF33 (gold)
rare<-data.frame(t(Tax2))
rare<-add_rownames(rare, "Sample")
rare$colors<-ifelse(grepl("_P1", rare$Sample), "#30241E",
                    ifelse(grepl("_P2", rare$Sample), "#B59B80",
                           ifelse(grepl("_S1", rare$Sample), "#33a02c",
                                  ifelse(grepl("_S2", rare$Sample), "#B2DF8A",
                                         "pink"))))
rare$line<-ifelse(grepl("HX", rare$Sample), "solid",
                  ifelse(grepl("NX", rare$Sample), "dashed",
                         ifelse(grepl("KAHI", rare$Sample), "dotted",
                                ifelse(grepl("NFNS", rare$Sample), "dotdash",
                                       "longdash"))))

#Plot rarefaction curves can decrease step for final plotting
set.seed(31)
rarecurve(t(Tax2), step=10000, xlab="Mapped reads", ylab="Genera", col=rare$colors,
          lty=rare$line, label=FALSE)
```

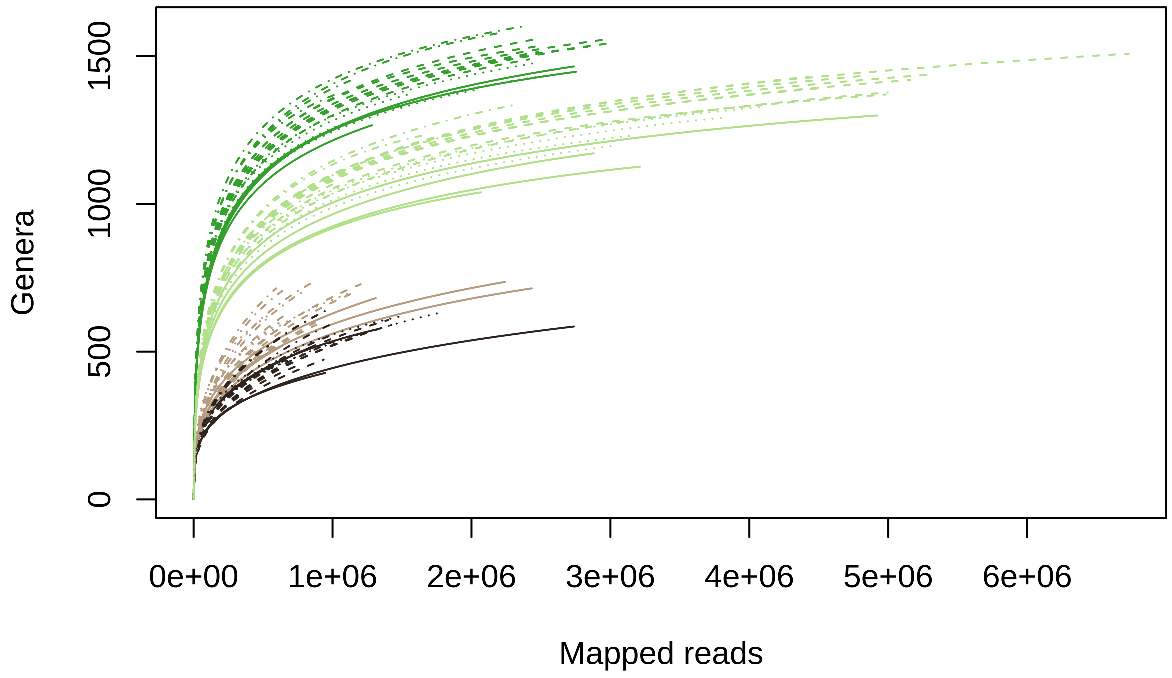

```r
pdf(paste("S2_Fig_Rarecurve.pdf", sep=""), height=6, width=8)
rarecurve(t(Tax2), step=10000, xlab="Mapped reads", ylab="Genera", col=rare$colors,
          lty=rare$line, label=FALSE)
dev.off()
```

```
## pdf
##   2
```

```r
#Calculate alpha diversity stats
#Use vegan to calculate various diversity and richness indexes for each sample
set.seed(32)
diversityCalc <- data.frame(Shannon=diversity(t(Tax2), index="shannon"),
                            Simpson=diversity(t(Tax2), index="simpson"),
                            invSimpson=diversity(t(Tax2), index="invsimpson"),
                            fisher=fisher.alpha(t(Tax2)), richness=specnumber(t(Tax2)),
                            rarefy_min_count=rarefy(t(Tax2), sample=min(rowSums(t(Tax2)))),
                            chao1=estimateR(t(Tax2))["S.chao1",],
                            chao1SE=estimateR(t(Tax2))["se.chao1",],
                            ShannonRar=diversity(rrarefy(data.frame(t(Tax2)),
                                                         min(rowSums(t(Tax2)))),
                                                 index="shannon"),
                            SimpsonRar=diversity(rrarefy(data.frame(t(Tax2)),
                                                         min(rowSums(t(Tax2)))),
                                                 index="simpson"),
                            invSimpsonRar=diversity(rrarefy(data.frame(t(Tax2)),
                                                            min(rowSums(t(Tax2)))),
                                                    index="invsimpson"),
                            Pielou=diversity(t(Tax2))/log(specnumber(t(Tax2))))
#Merge with metadata and make S2 Table.
diversityCalc<-add_rownames(diversityCalc, "Sample")
Metadata2<-merge(Metadata2, diversityCalc, by="Sample")

alpha <- dplyr::select(Metadata2, one_of(c("Sample", "Experiment", "LPSP", "QCperf",
                                           "chao1", "Pielou", "Simpson")))
describeBy(alpha, alpha$LPSP)
```

```
##
##  Descriptive statistics by group
## group: Kappa_Hiseq
##              vars  n    mean     sd  median trimmed    mad    min     max
## Sample*         1 16    8.50   4.76    8.50    8.50   5.93   1.00   16.00
## Experiment*     2 16    2.50   1.15    2.50    2.50   1.48   1.00    4.00
## LPSP*           3 16    1.00   0.00    1.00    1.00   0.00   1.00    1.00
## QCperf*         4 16    2.50   1.15    2.50    2.50   1.48   1.00    4.00
## chao1           5 16 1142.76 383.38 1146.41 1143.62 534.46 614.76 1658.75
## Pielou          6 16    0.40   0.08    0.40    0.40   0.10   0.27    0.50
## Simpson         7 16    0.79   0.13    0.82    0.80   0.15   0.57    0.94
##                range  skew kurtosis    se
## Sample*        15.00  0.00    -1.43  1.19
## Experiment*     3.00  0.00    -1.56  0.29
## LPSP*           0.00   NaN      NaN  0.00
## QCperf*         3.00  0.00    -1.56  0.29
## chao1        1043.99  0.00    -1.88 95.84
## Pielou          0.23 -0.12    -1.25  0.02
## Simpson         0.37 -0.41    -1.43  0.03
```

```
## ------------------------------------------------------------
## group: Nextera_Nextseq
##              vars  n    mean     sd  median trimmed    mad    min     max
## Sample*         1 32   16.50   9.38   16.50   16.50  11.86   1.00   32.00
## Experiment*     2 32    2.50   1.14    2.50    2.50   1.48   1.00    4.00
## LPSP*           3 32    1.00   0.00    1.00    1.00   0.00   1.00    1.00
## QCperf*         4 32    2.50   1.14    2.50    2.50   1.48   1.00    4.00
## chao1           5 32 1237.49 416.70 1254.07 1243.28 597.91 650.45 1721.46
## Pielou          6 32    0.37   0.09    0.38    0.37   0.11   0.22    0.48
## Simpson         7 32    0.75   0.17    0.79    0.76   0.17   0.44    0.93
##                range  skew kurtosis    se
## Sample*        31.00  0.00    -1.31  1.66
## Experiment*     3.00  0.00    -1.46  0.20
## LPSP*           0.00   NaN      NaN  0.00
## QCperf*         3.00  0.00    -1.46  0.20
## chao1        1071.00 -0.03    -1.93 73.66
## Pielou          0.26 -0.33    -1.22  0.02
## Simpson         0.49 -0.51    -1.28  0.03
## ------------------------------------------------------------
## group: Nextflex_Hiseq
##              vars  n    mean     sd  median trimmed    mad    min     max
## Sample*         1 16    8.50   4.76    8.50    8.50   5.93   1.00   16.00
## Experiment*     2 16    2.50   1.15    2.50    2.50   1.48   1.00    4.00
## LPSP*           3 16    1.00   0.00    1.00    1.00   0.00   1.00    1.00
## QCperf*         4 16    2.50   1.15    2.50    2.50   1.48   1.00    4.00
## chao1           5 16 1106.89 348.63 1052.48 1105.69 429.56 581.72 1648.99
## Pielou          6 16    0.38   0.08    0.40    0.39   0.11   0.25    0.49
## Simpson         7 16    0.78   0.14    0.80    0.78   0.14   0.55    0.94
##                range  skew kurtosis    se
## Sample*        15.00  0.00    -1.43  1.19
## Experiment*     3.00  0.00    -1.56  0.29
## LPSP*           0.00   NaN      NaN  0.00
## QCperf*         3.00  0.00    -1.56  0.29
## chao1        1067.27  0.12    -1.62 87.16
## Pielou          0.23 -0.15    -1.30  0.02
## Simpson         0.39 -0.43    -1.35  0.03
## ------------------------------------------------------------
## group: Nextflex_Nextseq
##              vars  n    mean     sd  median trimmed    mad    min     max
## Sample*         1 16    8.50   4.76    8.50    8.50   5.93   1.00   16.00
## Experiment*     2 16    2.50   1.15    2.50    2.50   1.48   1.00    4.00
## LPSP*           3 16    1.00   0.00    1.00    1.00   0.00   1.00    1.00
## QCperf*         4 16    2.50   1.15    2.50    2.50   1.48   1.00    4.00
## chao1           5 16 1221.88 347.83 1171.35 1213.03 429.22 775.58 1792.15
## Pielou          6 16    0.38   0.09    0.39    0.38   0.11   0.24    0.50
## Simpson         7 16    0.77   0.15    0.80    0.77   0.16   0.53    0.94
##                range  skew kurtosis    se
## Sample*        15.00  0.00    -1.43  1.19
## Experiment*     3.00  0.00    -1.56  0.29
## LPSP*           0.00   NaN      NaN  0.00
## QCperf*         3.00  0.00    -1.56  0.29
## chao1        1016.57  0.24    -1.65 86.96
## Pielou          0.25 -0.09    -1.34  0.02
## Simpson         0.42 -0.39    -1.38  0.04
```

```
describeBy(alpha, alpha$QCperf)
```

```
##
##  Descriptive statistics by group
## group: Kappa_Hiseq_Pig_feces_1
##             vars n   mean     sd median trimmed    mad    min    max  range
## Sample*        1 4   2.50   1.29   2.50    2.50   1.48   1.00   4.00   3.00
## Experiment*    2 4   1.00   0.00   1.00    1.00   0.00   1.00   1.00   0.00
## LPSP*          3 4   1.00   0.00   1.00    1.00   0.00   1.00   1.00   0.00
## QCperf*        4 4   1.00   0.00   1.00    1.00   0.00   1.00   1.00   0.00
## chao1          5 4 730.66 115.57 715.12  730.66 109.74 614.76 877.65 262.89
## Pielou         6 4   0.31   0.05   0.31    0.31   0.06   0.27   0.37   0.10
## Simpson        7 4   0.66   0.10   0.66    0.66   0.12   0.57   0.76   0.19
##             skew kurtosis    se
## Sample*     0.00    -2.08  0.65
## Experiment*  NaN      NaN  0.00
## LPSP*        NaN      NaN  0.00
## QCperf*      NaN      NaN  0.00
## chao1       0.23    -2.07 57.78
## Pielou      0.03    -2.39  0.03
## Simpson     0.02    -2.41  0.05
## ------------------------------------------------------------
## group: Kappa_Hiseq_Pig_feces_2
##             vars n   mean    sd median trimmed   mad    min    max  range skew
## Sample*        1 4   2.50  1.29   2.50    2.50  1.48   1.00   4.00   3.00 0.00
## Experiment*    2 4   1.00  0.00   1.00    1.00  0.00   1.00   1.00   0.00  NaN
## LPSP*          3 4   1.00  0.00   1.00    1.00  0.00   1.00   1.00   0.00  NaN
## QCperf*        4 4   1.00  0.00   1.00    1.00  0.00   1.00   1.00   0.00  NaN
## chao1          5 4 840.68 75.04 822.19  840.68 39.32 771.09 947.26 176.17 0.51
## Pielou         6 4   0.36  0.04   0.36    0.36  0.06   0.31   0.40   0.08 0.00
## Simpson        7 4   0.69  0.09   0.69    0.69  0.11   0.61   0.78   0.17 0.00
##             kurtosis    se
## Sample*        -2.08  0.65
## Experiment*      NaN  0.00
## LPSP*            NaN  0.00
## QCperf*          NaN  0.00
## chao1          -1.80 37.52
## Pielou         -2.42  0.02
## Simpson        -2.40  0.04
## ------------------------------------------------------------
## group: Kappa_Hiseq_Sewage_1
##             vars n    mean    sd  median trimmed   mad     min     max  range
## Sample*        1 4    2.50  1.29    2.50    2.50  1.48    1.00    4.00   3.00
## Experiment*    2 4    1.00  0.00    1.00    1.00  0.00    1.00    1.00   0.00
## LPSP*          3 4    1.00  0.00    1.00    1.00  0.00    1.00    1.00   0.00
## QCperf*        4 4    1.00  0.00    1.00    1.00  0.00    1.00    1.00   0.00
## chao1          5 4 1584.50 51.81 1567.07 1584.50 26.50 1545.11 1658.75 113.64
## Pielou         6 4    0.50  0.01    0.50    0.50  0.00    0.49    0.50   0.01
## Simpson        7 4    0.94  0.01    0.94    0.94  0.01    0.93    0.94   0.01
##              skew kurtosis    se
## Sample*      0.00    -2.08  0.65
## Experiment*   NaN      NaN  0.00
## LPSP*         NaN      NaN  0.00
## QCperf*       NaN      NaN  0.00
```

```
## chao1        0.57    -1.83 25.90
## Pielou      -0.62    -1.78  0.00
## Simpson     -0.05    -2.38  0.00
## ------------------------------------------------------------
## group: Kappa_Hiseq_Sewage_2
##             vars n    mean    sd  median trimmed   mad     min     max  range
## Sample*        1 4    2.50  1.29    2.50    2.50  1.48    1.00    4.00   3.00
## Experiment*    2 4    1.00  0.00    1.00    1.00  0.00    1.00    1.00   0.00
## LPSP*          3 4    1.00  0.00    1.00    1.00  0.00    1.00    1.00   0.00
## QCperf*        4 4    1.00  0.00    1.00    1.00  0.00    1.00    1.00   0.00
## chao1          5 4 1415.20 77.31 1411.59 1415.20 93.26 1345.56 1492.07 146.51
## Pielou         6 4    0.42  0.01    0.42    0.42  0.01    0.41    0.42   0.01
## Simpson        7 4    0.87  0.00    0.87    0.87  0.01    0.87    0.87   0.01
##              skew kurtosis    se
## Sample*      0.00    -2.08  0.65
## Experiment*   NaN      NaN  0.00
## LPSP*         NaN      NaN  0.00
## QCperf*       NaN      NaN  0.00
## chao1        0.02    -2.41 38.66
## Pielou      -0.20    -1.94  0.00
## Simpson     -0.07    -2.31  0.00
## ------------------------------------------------------------
## group: Nextera_Nextseq_Pig_feces_1
##             vars n   mean    sd median trimmed   mad    min    max  range  skew
## Sample*        1 8   4.50  2.45   4.50    4.50  2.97   1.00   8.00   7.00  0.00
## Experiment*    2 8   1.00  0.00   1.00    1.00  0.00   1.00   1.00   0.00   NaN
## LPSP*          3 8   1.00  0.00   1.00    1.00  0.00   1.00   1.00   0.00   NaN
## QCperf*        4 8   1.00  0.00   1.00    1.00  0.00   1.00   1.00   0.00   NaN
## chao1          5 8 799.48 75.68 814.84  799.48 66.04 650.45 884.51 234.05 -0.71
## Pielou         6 8   0.27  0.06   0.27    0.27  0.07   0.22   0.34   0.12  0.02
## Simpson        7 8   0.58  0.14   0.57    0.58  0.18   0.44   0.72   0.28  0.01
##             kurtosis    se
## Sample*        -1.65  0.87
## Experiment*      NaN  0.00
## LPSP*            NaN  0.00
## QCperf*          NaN  0.00
## chao1          -0.81 26.76
## Pielou         -2.20  0.02
## Simpson        -2.21  0.05
## ------------------------------------------------------------
## group: Nextera_Nextseq_Pig_feces_2
##             vars n   mean    sd median trimmed   mad    min    max  range  skew
## Sample*        1 8   4.50  2.45   4.50    4.50  2.97   1.00   8.00   7.00  0.00
## Experiment*    2 8   1.00  0.00   1.00    1.00  0.00   1.00   1.00   0.00   NaN
## LPSP*          3 8   1.00  0.00   1.00    1.00  0.00   1.00   1.00   0.00   NaN
## QCperf*        4 8   1.00  0.00   1.00    1.00  0.00   1.00   1.00   0.00   NaN
## chao1          5 8 869.76 96.33 883.46  869.76 94.13 712.63 982.58 269.95 -0.33
## Pielou         6 8   0.31  0.05   0.31    0.31  0.06   0.26   0.36   0.10  0.00
## Simpson        7 8   0.61  0.09   0.62    0.61  0.11   0.53   0.70   0.17 -0.01
##             kurtosis    se
## Sample*        -1.65  0.87
## Experiment*      NaN  0.00
## LPSP*            NaN  0.00
## QCperf*          NaN  0.00
```

```
## chao1          -1.60 34.06
## Pielou         -2.20  0.02
## Simpson        -2.22  0.03
## ------------------------------------------------------------
## group: Nextera_Nextseq_Sewage_1
##             vars n    mean    sd  median trimmed   mad     min     max range
## Sample*        1 8    4.50  2.45    4.50    4.50  2.97    1.00    8.00  7.00
## Experiment*    2 8    1.00  0.00    1.00    1.00  0.00    1.00    1.00  0.00
## LPSP*          3 8    1.00  0.00    1.00    1.00  0.00    1.00    1.00  0.00
## QCperf*        4 8    1.00  0.00    1.00    1.00  0.00    1.00    1.00  0.00
## chao1          5 8 1689.45 35.69 1704.10 1689.45 25.44 1642.44 1721.46 79.02
## Pielou         6 8    0.47  0.01    0.47    0.47  0.00    0.46    0.48  0.02
## Simpson        7 8    0.92  0.00    0.92    0.92  0.00    0.92    0.93  0.01
##              skew kurtosis    se
## Sample*      0.00    -1.65  0.87
## Experiment*   NaN      NaN  0.00
## LPSP*         NaN      NaN  0.00
## QCperf*       NaN      NaN  0.00
## chao1       -0.28    -2.01 12.62
## Pielou      -0.56    -1.25  0.00
## Simpson     -0.28    -1.72  0.00
## ------------------------------------------------------------
## group: Nextera_Nextseq_Sewage_2
##             vars n    mean    sd  median trimmed   mad     min     max  range
## Sample*        1 8    4.50  2.45    4.50    4.50  2.97    1.00    8.00   7.00
## Experiment*    2 8    1.00  0.00    1.00    1.00  0.00    1.00    1.00   0.00
## LPSP*          3 8    1.00  0.00    1.00    1.00  0.00    1.00    1.00   0.00
## QCperf*        4 8    1.00  0.00    1.00    1.00  0.00    1.00    1.00   0.00
## chao1          5 8 1591.28 49.18 1581.63 1591.28 50.83 1525.56 1664.41 138.85
## Pielou         6 8    0.41  0.01    0.41    0.41  0.01    0.39    0.42   0.02
## Simpson        7 8    0.87  0.01    0.87    0.87  0.01    0.86    0.88   0.02
##              skew kurtosis    se
## Sample*      0.00    -1.65  0.87
## Experiment*   NaN      NaN  0.00
## LPSP*         NaN      NaN  0.00
## QCperf*       NaN      NaN  0.00
## chao1        0.21    -1.68 17.39
## Pielou       0.01    -1.76  0.00
## Simpson     -0.02    -1.68  0.00
## ------------------------------------------------------------
## group: Nextflex_Hiseq_Pig_feces_1
##             vars n   mean     sd median trimmed   mad    min    max  range
## Sample*        1 4   2.50   1.29   2.50    2.50  1.48   1.00   4.00   3.00
## Experiment*    2 4   1.00   0.00   1.00    1.00  0.00   1.00   1.00   0.00
## LPSP*          3 4   1.00   0.00   1.00    1.00  0.00   1.00   1.00   0.00
## QCperf*        4 4   1.00   0.00   1.00    1.00  0.00   1.00   1.00   0.00
## chao1          5 4 725.50 103.91 748.52  725.50 72.80 581.72 823.25 241.53
## Pielou         6 4   0.30   0.05   0.30    0.30  0.06   0.25   0.35   0.10
## Simpson        7 4   0.64   0.11   0.64    0.64  0.14   0.55   0.75   0.20
##              skew kurtosis    se
## Sample*      0.00    -2.08  0.65
## Experiment*   NaN      NaN  0.00
## LPSP*         NaN      NaN  0.00
## QCperf*       NaN      NaN  0.00
```

```
## chao1       -0.43    -1.88 51.95
## Pielou       0.01    -2.36  0.02
## Simpson      0.01    -2.42  0.06
## ------------------------------------------------------------
## group: Nextflex_Hiseq_Pig_feces_2
##             vars n   mean    sd median trimmed   mad    min    max  range  skew
## Sample*        1 4   2.50  1.29   2.50    2.50  1.48   1.00   4.00   3.00  0.00
## Experiment*    2 4   1.00  0.00   1.00    1.00  0.00   1.00   1.00   0.00   NaN
## LPSP*          3 4   1.00  0.00   1.00    1.00  0.00   1.00   1.00   0.00   NaN
## QCperf*        4 4   1.00  0.00   1.00    1.00  0.00   1.00   1.00   0.00   NaN
## chao1          5 4 868.22 84.51 892.55  868.22 58.26 753.49 934.29 180.79 -0.41
## Pielou         6 4   0.34  0.05   0.34    0.34  0.06   0.29   0.39   0.10  0.02
## Simpson        7 4   0.68  0.09   0.68    0.68  0.11   0.60   0.76   0.16 -0.01
##             kurtosis    se
## Sample*        -2.08  0.65
## Experiment*      NaN  0.00
## LPSP*            NaN  0.00
## QCperf*          NaN  0.00
## chao1          -1.99 42.26
## Pielou         -2.40  0.03
## Simpson        -2.43  0.04
## ------------------------------------------------------------
## group: Nextflex_Hiseq_Sewage_1
##             vars n    mean    sd  median trimmed   mad     min     max  range
## Sample*        1 4    2.50  1.29    2.50    2.50  1.48    1.00    4.00   3.00
## Experiment*    2 4    1.00  0.00    1.00    1.00  0.00    1.00    1.00   0.00
## LPSP*          3 4    1.00  0.00    1.00    1.00  0.00    1.00    1.00   0.00
## QCperf*        4 4    1.00  0.00    1.00    1.00  0.00    1.00    1.00   0.00
## chao1          5 4 1547.13 87.86 1538.30 1547.13 94.88 1462.94 1648.99 186.05
## Pielou         6 4    0.48  0.00    0.48    0.48  0.00    0.48    0.49   0.01
## Simpson        7 4    0.93  0.01    0.93    0.93  0.01    0.92    0.94   0.01
##              skew kurtosis    se
## Sample*      0.00    -2.08  0.65
## Experiment*   NaN      NaN  0.00
## LPSP*         NaN      NaN  0.00
## QCperf*       NaN      NaN  0.00
## chao1        0.11    -2.26 43.93
## Pielou      -0.06    -2.33  0.00
## Simpson      0.00    -2.43  0.00
## ------------------------------------------------------------
## group: Nextflex_Hiseq_Sewage_2
##             vars n    mean     sd  median trimmed    mad     min     max  range
## Sample*        1 4    2.50   1.29    2.50    2.50   1.48    1.00    4.00   3.00
## Experiment*    2 4    1.00   0.00    1.00    1.00   0.00    1.00    1.00   0.00
## LPSP*          3 4    1.00   0.00    1.00    1.00   0.00    1.00    1.00   0.00
## QCperf*        4 4    1.00   0.00    1.00    1.00   0.00    1.00    1.00   0.00
## chao1          5 4 1286.72 103.11 1283.13 1286.72 113.11 1170.68 1409.95 239.27
## Pielou         6 4    0.41   0.01    0.41    0.41   0.00    0.40    0.41   0.01
## Simpson        7 4    0.85   0.01    0.86    0.85   0.01    0.85    0.86   0.01
##              skew kurtosis    se
## Sample*      0.00    -2.08  0.65
## Experiment*   NaN      NaN  0.00
## LPSP*         NaN      NaN  0.00
## QCperf*       NaN      NaN  0.00
```

```
## chao1        0.06    -2.08 51.56
## Pielou      -0.54    -1.84  0.00
## Simpson     -0.13    -2.28  0.00
## ------------------------------------------------------------
## group: Nextflex_Nextseq_Pig_feces_1
##             vars n   mean    sd median trimmed   mad    min    max  range  skew
## Sample*        1 4   2.50  1.29   2.50    2.50  1.48   1.00   4.00   3.00  0.00
## Experiment*    2 4   1.00  0.00   1.00    1.00  0.00   1.00   1.00   0.00   NaN
## LPSP*          3 4   1.00  0.00   1.00    1.00  0.00   1.00   1.00   0.00   NaN
## QCperf*        4 4   1.00  0.00   1.00    1.00  0.00   1.00   1.00   0.00   NaN
## chao1          5 4 850.17 50.86 868.79  850.17 19.36 775.58 887.53 111.95 -0.65
## Pielou         6 4   0.29  0.05   0.28    0.29  0.06   0.24   0.34   0.09  0.03
## Simpson        7 4   0.62  0.11   0.62    0.62  0.14   0.53   0.73   0.21  0.01
##             kurtosis    se
## Sample*        -2.08  0.65
## Experiment*      NaN  0.00
## LPSP*            NaN  0.00
## QCperf*          NaN  0.00
## chao1          -1.75 25.43
## Pielou         -2.40  0.03
## Simpson        -2.43  0.06
## ------------------------------------------------------------
## group: Nextflex_Nextseq_Pig_feces_2
##             vars n   mean    sd median trimmed   mad    min     max  range
## Sample*        1 4   2.50  1.29   2.50    2.50  1.48   1.00    4.00   3.00
## Experiment*    2 4   1.00  0.00   1.00    1.00  0.00   1.00    1.00   0.00
## LPSP*          3 4   1.00  0.00   1.00    1.00  0.00   1.00    1.00   0.00
## QCperf*        4 4   1.00  0.00   1.00    1.00  0.00   1.00    1.00   0.00
## chao1          5 4 970.67 53.81 951.53  970.67 20.30 930.01 1049.61 119.60
## Pielou         6 4   0.33  0.05   0.33    0.33  0.06   0.29    0.37   0.08
## Simpson        7 4   0.67  0.09   0.67    0.67  0.11   0.58    0.74   0.16
##              skew kurtosis    se
## Sample*      0.00    -2.08  0.65
## Experiment*   NaN      NaN  0.00
## LPSP*         NaN      NaN  0.00
## QCperf*       NaN      NaN  0.00
## chao1        0.65    -1.75 26.90
## Pielou      -0.01    -2.43  0.02
## Simpson     -0.01    -2.43  0.04
## ------------------------------------------------------------
## group: Nextflex_Nextseq_Sewage_1
##             vars n    mean     sd  median trimmed    mad     min     max  range
## Sample*        1 4    2.50   1.29    2.50    2.50   1.48    1.00    4.00   3.00
## Experiment*    2 4    1.00   0.00    1.00    1.00   0.00    1.00    1.00   0.00
## LPSP*          3 4    1.00   0.00    1.00    1.00   0.00    1.00    1.00   0.00
## QCperf*        4 4    1.00   0.00    1.00    1.00   0.00    1.00    1.00   0.00
## chao1          5 4 1656.86 126.52 1664.11 1656.86 139.31 1507.06 1792.15 285.09
## Pielou         6 4    0.49   0.00    0.49    0.49   0.00    0.49    0.50   0.01
## Simpson        7 4    0.93   0.01    0.94    0.93   0.01    0.93    0.94   0.01
##              skew kurtosis    se
## Sample*      0.00    -2.08  0.65
## Experiment*   NaN      NaN  0.00
## LPSP*         NaN      NaN  0.00
## QCperf*       NaN      NaN  0.00
```

```
## chao1        -0.09    -2.15 63.26
## Pielou       -0.46    -1.88  0.00
## Simpson      -0.01    -2.42  0.00
## ------------------------------------------------------------
## group: Nextflex_Nextseq_Sewage_2
##             vars n    mean     sd  median trimmed    mad     min     max  range
## Sample*        1 4    2.50   1.29    2.50    2.50   1.48    1.00    4.00   3.00
## Experiment*    2 4    1.00   0.00    1.00    1.00   0.00    1.00    1.00   0.00
## LPSP*          3 4    1.00   0.00    1.00    1.00   0.00    1.00    1.00   0.00
## QCperf*        4 4    1.00   0.00    1.00    1.00   0.00    1.00    1.00   0.00
## chao1          5 4 1409.83 125.32 1388.94 1409.83 116.41 1293.10 1568.33 275.24
## Pielou         6 4    0.41   0.01    0.41    0.41   0.01    0.40    0.42   0.02
## Simpson        7 4    0.85   0.00    0.85    0.85   0.00    0.85    0.86   0.01
##              skew kurtosis    se
## Sample*      0.00    -2.08  0.65
## Experiment*   NaN      NaN  0.00
## LPSP*         NaN      NaN  0.00
## QCperf*       NaN      NaN  0.00
## chao1        0.24    -2.12 62.66
## Pielou      -0.22    -1.94  0.00
## Simpson      0.05    -2.08  0.00
```

```
write.table(alpha, file="S1_Table_alphaOverview.txt", quote=F, sep="\t")
```

PCA

All, pig feces both P1 and P2, and sewage both S1 and S2 creating S3_Fig

```
rm(list=setdiff(ls(), c("Metadata", "Feature", "Tax")))

#Subset data pig feces 1 and 2 (P1, P2), Sewage 1 and 2 (S1, S2), or spiked unspiked
Subset<- c("PF", "SW", "All") #All, Allspiked, Allunspiked, PF, SW, P1, P1spiked,
  #P1unspiked, P2, P2spiked, P2unspiked, S1, S1spiked, S1unspiked, S2, S2spiked,
  #S2unspiked
#i<-c("All")

#Subset experiment, Experiment_Type. (Meaningfull combinations of subset and SubExp:
  #HX=All, FTX=P1&S1, LTX=P1&S1, LPSX=All unspiked, HXFTX=P1&S1, HXLTX=P1&S1,
  #HXLPSX=All unspiked, HXLTXLPSX=P1&S1 unspiked)
SubExp<-"LPSX" #HX, FTX, LTX, LPSX, HXFTX, HXLTX, HXLPSX, HXLTXLPSX, HXFTXLTX, All

#Subset Frozen Unfrozen
SubFre<-"Both" #Frozen, Unfrozen, Both

# Create a list to hold the plot objects.
ScreeList <- list()
StressList <- list()
PCAList <- list()
vec<-vector()

for (i in Subset) {
#Removing negative and positive controls
Metadata2<-filter(Metadata, Sample_type_simple=="Sample")

#Subsetting Metadata2
```

```
if (i=="Allspiked") {
  Metadata2<-filter(Metadata2, SpikedUnspiked == "Spiked")
} else if (i=="Allunspiked") {
  Metadata2<-filter(Metadata2, SpikedUnspiked == "Unspiked")
} else if (i=="PF") {
  Metadata2<-filter(Metadata2, Experiment == "Pig_feces_1" | Experiment == "Pig_feces_2")
} else if (i=="SW") {
  Metadata2<-filter(Metadata2, Experiment == "Sewage_1" | Experiment == "Sewage_2")
} else if (i=="P1") {
  Metadata2<-filter(Metadata2, Experiment == "Pig_feces_1")
} else if (i=="P1spiked") {
  Metadata2<-filter(Metadata2, Sample_type == "Pig_feces_1_spiked")
} else if (i=="P1unspiked") {
  Metadata2<-filter(Metadata2, Sample_type == "Pig_feces_1")
} else if (i=="P2") {
  Metadata2<-filter(Metadata2, Experiment == "Pig_feces_2")
} else if (i=="P2spiked") {
  Metadata2<-filter(Metadata2, Sample_type == "Pig_feces_2_spiked")
} else if (i=="P2unspiked") {
  Metadata2<-filter(Metadata2, Sample_type == "Pig_feces_2")
} else if (i=="S1") {
  Metadata2<-filter(Metadata2, Experiment == "Sewage_1")
} else if (i=="S1spiked") {
  Metadata2<-filter(Metadata2, Sample_type == "Sewage_1_spiked")
} else if (i=="S1unspiked") {
  Metadata2<-filter(Metadata2, Sample_type == "Sewage_1")
} else if (i=="S2") {
  Metadata2<-filter(Metadata2, Experiment == "Sewage_2")
} else if (i=="S2spiked") {
  Metadata2<-filter(Metadata2, Sample_type == "Sewage_2_spiked")
} else if (i=="S2unspiked") {
  Metadata2<-filter(Metadata2, Sample_type == "Sewage_2")
} else if (i=="All") {
  print("No subsetting Subset, all included")
} else {
  print("Subset defined not valid")
}

#Further subsetting Metadata2
if (SubExp=="HX") {
  Metadata2<-filter(Metadata2, Experiment_type == "Handling_experiment")
} else if (SubExp=="FTX") {
  Metadata2<-filter(Metadata2, Experiment_type == "Freeze_thaw_experiment")
} else if (SubExp=="LTX") {
  Metadata2<-filter(Metadata2, Experiment_type == "Long_term_storage_experiment")
} else if (SubExp=="LPSX") {
  Metadata22<-filter(Metadata2, Experiment_type == "Library_prep_seq_platform_experiment")
  VectorLPSX <- unique(Metadata22$Matching_samples)
  Metadata2<-filter(Metadata2, Matching_samples %in% VectorLPSX)
  rm(VectorLPSX, Metadata22)
} else if (SubExp=="HXFTX") {
  Metadata2<-filter(Metadata2, Experiment_type == "Handling_experiment" |
                    Experiment_type == "Freeze_thaw_experiment")
```

```
} else if (SubExp=="HXLTX") {
  Metadata2<-filter(Metadata2, Experiment_type == "Handling_experiment" |
                     Experiment_type == "Long_term_storage_experiment")
} else if (SubExp=="HXLPSX") {
  Metadata2<-filter(Metadata2, Experiment_type == "Handling_experiment" |
                     Experiment_type == "Library_prep_seq_platform_experiment")
} else if (SubExp=="HXLTXLPSX") {
  Metadata2<-filter(Metadata2, Experiment_type == "Handling_experiment" |
                     Experiment_type == "Long_term_storage_experiment" |
                     Experiment_type == "Library_prep_seq_platform_experiment")
} else if (SubExp=="HXFTXLTX") {
  Metadata2<-filter(Metadata2, Experiment_type == "Handling_experiment" |
                     Experiment_type == "Freeze_thaw_experiment" |
                     Experiment_type == "Long_term_storage_experiment")
} else if (SubExp=="All") {
  print("No subsetting SubExp, all included")
} else {
  print("Subset defined not valid")
}

#Further subsetting Metadata2
if (SubFre=="Frozen") {
  Metadata2<-filter(Metadata2, FrozenUnfrozenSimple == "Freezer")
} else if (SubFre=="Unfrozen") {
  Metadata2<-filter(Metadata2, FrozenUnfrozenSimple == "Unfrozen")
} else if (SubFre=="Both") {
  print("No subsetting SubFre, all included")
} else {
  print("Subset defined not valid")
}


#Removing the Kappa NextSeq run
Metadata2<-Metadata2[grep("\\KANS.*", Metadata2$Sample, invert=TRUE),]

#Applying subsetting to OTU tables
Tax2<-dplyr::select(Tax, one_of(Metadata2$Sample))

## Create grouping factor
#colnames(Tax2)==Metadata$Sample #Checking order
#New data adding line between same samples different lib prep and seq plat
Metadata2$Sample_LPSX <-
        ifelse(seq(along=(Metadata2$Sample)) %in% grep("*_HX_*", Metadata2$Sample),
               "NFHI",
        ifelse(seq(along=(Metadata2$Sample)) %in% grep("*KAHI*", Metadata2$Sample),
               "KAHI",
        ifelse(seq(along=(Metadata2$Sample)) %in% grep("*NFNS*", Metadata2$Sample),
               "NFNS",
        ifelse(seq(along=(Metadata2$Sample)) %in% grep("*NX1NS*", Metadata2$Sample),
               "NX1NS",
        ifelse(seq(along=(Metadata2$Sample)) %in% grep("*NX2NS*", Metadata2$Sample),
               "NX2NS",
        ifelse(seq(along=(Metadata2$Sample)) %in% grep("*KANS*", Metadata2$Sample),
```

```
               "KANS",
               "Other")))))) #The seq along returns true or false where the regex
  #is fulfilled and then stores the evaluated string on the new column be aware
  #HX is actually true 1-8 don't know why


## Can use different filtering strategies. 1 Bayseian approach estimating zeroes,
  #2 offset of 1 and removing all rows containing zeroes. Only minor effects observed
# f <- codaSeq.filter(ak_op, min.reads=1000, min.prop=0.005, min.occurrence=0.2,
  #samples.by.row=FALSE) #Implemented in Gloors codaSeq.filter function
#Assessing what is the max value of rows containing a zero
print("Max in rows containing a zero")
row_sub = apply(Tax2, 1, function(row) all(row !=0 ))
Tax2[row_sub,] %>% max() %>% print()
# filtering of the Counttable depending on rowSums.
Tax2 <- Tax2[rowSums(Tax2)>0,] #Removing all rows that only contains zeroes
Tax2 <- Tax2[rowSums(Tax2)>(5*ncol(Tax2)),] #Removing all rows(Species)
  #that is below an average count of 5.
# replace 0 values with an estimate using simple multiplicative replacement
Tax2 <- t(cmultRepl(t(Tax2), method="CZM", label=0))

## Maks TSS
Tax2<-sweep(Tax2, 2, colSums(Tax2), FUN="/")

Y <- Metadata2$Sample_LPSX

PCACoDa = mixOmics::pca(t(Tax2), ncomp = 10, logratio = 'ILR')#Can change CLR and ILR
#plot(PCACoDa)

#plotIndiv(PCACoDa,
#         comp = c(1,2), # the components to plot
#         pch = 16,
#         ind.names = F,
#         group = Y,
#         col.per.group = color.mixo(1:5),
#         legend = TRUE,
#         title = 'PCA comp 1 - 2')

# Calculate the variation explained by PCoA1 and 2
# and use it to generate axis labels
eig_1 <- paste("PCA1", round(PCACoDa$explained_variance[1]*100, digits = 1), "% variance")
eig_2 <- paste("PCA2", round(PCACoDa$explained_variance[2]*100, digits = 1), "% variance")
eig_3 <- paste("PCA3", round(PCACoDa$explained_variance[3]*100, digits = 1), "% variance")
eig_4 <- paste("PCA4", round(PCACoDa$explained_variance[4]*100, digits = 1), "% variance")

##Pull out coordinates for plotting from the ca object
#Structuring to add to metadata
PCAMeta<-data.frame(Sample=PCACoDa$names$sample, PCA1=PCACoDa$variates$X[,1],
                    PCA2=PCACoDa$variates$X[,2], PCA3=PCACoDa$variates$X[,3],
                    PCA4=PCACoDa$variates$X[,4])
#Merge according to
Metadata2<-merge(Metadata2, PCAMeta, by="Sample")
#Creating column in metadata for plotting with lines between replicates
```

```
Metadata2$Sample_name_norep<-gsub("*_a|*_b|*_c", "", Metadata2$Sample_name)
#Change Storage and time to characters for plotting
Metadata2$Storage<-as.character(Metadata2$Storage)
Metadata2$Time<-as.character(Metadata2$Time)
#Change NAs in Storage to Direct
Metadata2$Storage[is.na(Metadata2$Storage)]<-"Direct"
#Change order for coloring
Metadata2$Storage<-ordered(Metadata2$Storage, levels=c("Direct", "-80", "-20", "5", "22"))

#Create plot name
pltName <- paste( 'PCA', i, sep = '' )
#create PCA
PCAList[[ pltName ]] <- ggplot(Metadata2) +
  geom_point(aes(PCA1, PCA2, color = Experiment, group = Sample), size=3) +
  #geom_line(aes(x=PCA1, y=PCA2, group=Replicate_Boxplot)) +
  #geom_line(aes(x=PCA1, y=PCA2, group=Matching_samples), size=0.1, linetype="dotted") +
  scale_color_manual(values=c(Pig_feces_1 = "#30241E", Pig_feces_2 = "#B59B80",
                              Sewage_1 = "#33a02c", Sewage_2 = "#B2DF8A")) +
  ggtitle(paste("PCA", i, sep=" ")) +
  labs(colour="Sample", x = eig_1, y = eig_2) +
  theme_bw() +
  theme(panel.grid.major = element_blank(), panel.grid.minor = element_blank(),
        axis.title=element_text(size=12), legend.position="none")
}
```

```
## [1] "No subsetting SubFre, all included"
## [1] "Max in rows containing a zero"
## [1] 1783518
## No. corrected values:  21
## [1] "No subsetting SubFre, all included"
## [1] "Max in rows containing a zero"
## [1] 1581043
## No. corrected values:  140
## [1] "No subsetting Subset, all included"
## [1] "No subsetting SubFre, all included"
## [1] "Max in rows containing a zero"
## [1] 1783518
## No. corrected values:  4863
```

```
#Draw PCA
#No validation plots
#Have the plots stored in lists
lay <- rbind(c(1,2),
             c(1,3))
#Make pdf
pdf(paste("S3_Fig_PCAAll", ".pdf", sep=""), width=9, height=6)
grid.arrange(PCAList$PCAAll, PCAList$PCAPF, PCAList$PCASW, layout_matrix = lay)
dev.off()
```

```
## pdf
##   2
```

```
#Make format for word
#png(paste("PCA", "GenusTall",  "NoVal", ".png", sep=""), width=900, height=600)
#grid.arrange(PCAList$PCAP1unspiked, PCAList$PCAP2unspiked, PCAList$PCAS1unspiked,
```

```
#             PCAList$PCAS2unspiked, layout_matrix = lay)
#dev.off()

#Extract legend
#legend<-ggplot(Metadata2) +
#  geom_point(aes(PCA1, PCA2, color = Sample_LPSX, group = Sample, shape = Storage),
  #size=1.5) +
#  #geom_line(aes(x=PCA1, y=PCA2, group=Replicate_Boxplot)) +
#  geom_line(aes(x=PCA1, y=PCA2, group=Matching_samples), size=0.1, linetype="dotted") +
#  scale_color_manual(values=c("#377eb8", "#4daf4a", "#984ea3", "#ff7f00", "#e41a1c")) +
#  scale_shape_manual(values=c(8,18,15,16,17,0,1,2)) +
#  ggtitle(paste("PCA", i, sep=" ")) +
#  labs(colour="Storage / ?C", shape="Processing", x = eig_1, y = eig_2) +
#  theme_bw() +
#  theme(panel.grid.major = element_blank(), panel.grid.minor = element_blank(),
  #axis.title=element_text(size=12))
#legendplot<-get_legend(legend)
#pdf(paste("LegendPCA", ".pdf", sep=""), width=24, height=12)
#grid.arrange(legendplot)
#dev.off()
#png(paste("LegendPCA", ".png", sep=""), width=100, height=300)
#grid.arrange(legendplot)
#dev.off()

legend2<-ggplot(Metadata2) +
  geom_point(aes(PCA1, PCA2, color = Experiment, group = Sample), size=5) +
  #geom_line(aes(x=PCA1, y=PCA2, group=Replicate_Boxplot)) +
  #geom_line(aes(x=PCA1, y=PCA2, group=Matching_samples), size=0.1, linetype="dotted") +
  scale_color_manual(values=c(Pig_feces_1 = "#30241E", Pig_feces_2 = "#B59B80",
                              Sewage_1 = "#33a02c", Sewage_2 = "#B2DF8A")) +
  ggtitle(paste("PCA", i, sep=" ")) +
  labs(colour="Sample", x = eig_1, y = eig_2) +
  theme_bw() +
  theme(panel.grid.major = element_blank(), panel.grid.minor = element_blank(),
        axis.title=element_text(size=12), legend.position="bottom")
legendplot<-get_legend(legend2)
pdf(paste("S3_Fig_PCAAllLegend", ".pdf", sep=""), width=10, height=1)
grid.arrange(legendplot)
dev.off()
```

```
## pdf
##   2
```

```
#png(paste("LegendPCAbottom", ".png", sep=""), width=600, height=25)
#grid.arrange(legendplot)
#dev.off()
```

PCA Fig_2

Subset to P1, P2, S1 and S2 creating Fig_2

```
rm(list=setdiff(ls(), c("Metadata", "Feature", "Tax")))

#Subset data pig feces 1 and 2 (P1, P2), Sewage 1 and 2 (S1, S2), or spiked unspiked
Subset<- c("P1unspiked", "P2unspiked", "S1unspiked", "S2unspiked") #All, Allspiked,
```

```
  #Allunspiked, PF, SW, P1, P1spiked, P1unspiked, P2, P2spiked, P2unspiked, S1, S1spiked,
  #S1unspiked, S2, S2spiked, S2unspiked

#Subset experiment, Experiment_Type. (Meaningfull combinations of subset and SubExp:
  #HX=All, FTX=P1&S1, LTX=P1&S1, LPSX=All unspiked, HXFTX=P1&S1, HXLTX=P1&S1,
  #HXLPSX=All unspiked, HXLTXLPSX=P1&S1 unspiked)
SubExp<-"LPSX" #HX, FTX, LTX, LPSX, HXFTX, HXLTX, HXLPSX, HXLTXLPSX, HXFTXLTX, All

#Subset Frozen Unfrozen
SubFre<-"Both" #Frozen, Unfrozen, Both

# Create a list to hold the plot objects.
ScreeList <- list()
StressList <- list()
PCAList <- list()
vec<-vector()

i<-c("P1unspiked")

for (i in Subset) {
#Removing negative and positive controls
Metadata2<-filter(Metadata, Sample_type_simple=="Sample")

#Subsetting Metadata2
if (i=="Allspiked") {
  Metadata2<-filter(Metadata2, SpikedUnspiked == "Spiked")
} else if (i=="Allunspiked") {
  Metadata2<-filter(Metadata2, SpikedUnspiked == "Unspiked")
} else if (i=="PF") {
  Metadata2<-filter(Metadata2, Experiment == "Pig_feces_1" | Experiment == "Pig_feces_2")
} else if (i=="SW") {
  Metadata2<-filter(Metadata2, Experiment == "Sewage_1" | Experiment == "Sewage_2")
} else if (i=="P1") {
  Metadata2<-filter(Metadata2, Experiment == "Pig_feces_1")
} else if (i=="P1spiked") {
  Metadata2<-filter(Metadata2, Sample_type == "Pig_feces_1_spiked")
} else if (i=="P1unspiked") {
  Metadata2<-filter(Metadata2, Sample_type == "Pig_feces_1")
} else if (i=="P2") {
  Metadata2<-filter(Metadata2, Experiment == "Pig_feces_2")
} else if (i=="P2spiked") {
  Metadata2<-filter(Metadata2, Sample_type == "Pig_feces_2_spiked")
} else if (i=="P2unspiked") {
  Metadata2<-filter(Metadata2, Sample_type == "Pig_feces_2")
} else if (i=="S1") {
  Metadata2<-filter(Metadata2, Experiment == "Sewage_1")
} else if (i=="S1spiked") {
  Metadata2<-filter(Metadata2, Sample_type == "Sewage_1_spiked")
} else if (i=="S1unspiked") {
  Metadata2<-filter(Metadata2, Sample_type == "Sewage_1")
} else if (i=="S2") {
  Metadata2<-filter(Metadata2, Experiment == "Sewage_2")
} else if (i=="S2spiked") {
```

```
  Metadata2<-filter(Metadata2, Sample_type == "Sewage_2_spiked")
} else if (i=="S2unspiked") {
  Metadata2<-filter(Metadata2, Sample_type == "Sewage_2")
} else if (i=="All") {
  print("No subsetting Subset, all included")
} else {
  print("Subset defined not valid")
}

#Further subsetting Metadata2
if (SubExp=="HX") {
  Metadata2<-filter(Metadata2, Experiment_type == "Handling_experiment")
} else if (SubExp=="FTX") {
  Metadata2<-filter(Metadata2, Experiment_type == "Freeze_thaw_experiment")
} else if (SubExp=="LTX") {
  Metadata2<-filter(Metadata2, Experiment_type == "Long_term_storage_experiment")
} else if (SubExp=="LPSX") {
  Metadata22<-filter(Metadata2, Experiment_type == "Library_prep_seq_platform_experiment")
  VectorLPSX <- unique(Metadata22$Matching_samples)
  Metadata2<-filter(Metadata2, Matching_samples %in% VectorLPSX)
  rm(VectorLPSX, Metadata22)
} else if (SubExp=="HXFTX") {
  Metadata2<-filter(Metadata2, Experiment_type == "Handling_experiment" |
                      Experiment_type == "Freeze_thaw_experiment")
} else if (SubExp=="HXLTX") {
  Metadata2<-filter(Metadata2, Experiment_type == "Handling_experiment" |
                      Experiment_type == "Long_term_storage_experiment")
} else if (SubExp=="HXLPSX") {
  Metadata2<-filter(Metadata2, Experiment_type == "Handling_experiment" |
                      Experiment_type == "Library_prep_seq_platform_experiment")
} else if (SubExp=="HXLTXLPSX") {
  Metadata2<-filter(Metadata2, Experiment_type == "Handling_experiment" |
                      Experiment_type == "Long_term_storage_experiment" |
                      Experiment_type == "Library_prep_seq_platform_experiment")
} else if (SubExp=="HXFTXLTX") {
  Metadata2<-filter(Metadata2, Experiment_type == "Handling_experiment" |
                      Experiment_type == "Freeze_thaw_experiment" |
                      Experiment_type == "Long_term_storage_experiment")
} else if (SubExp=="All") {
  print("No subsetting SubExp, all included")
} else {
  print("Subset defined not valid")
}

#Further subsetting Metadata2
if (SubFre=="Frozen") {
  Metadata2<-filter(Metadata2, FrozenUnfrozenSimple == "Freezer")
} else if (SubFre=="Unfrozen") {
  Metadata2<-filter(Metadata2, FrozenUnfrozenSimple == "Unfrozen")
} else if (SubFre=="Both") {
  print("No subsetting SubFre, all included")
} else {
  print("Subset defined not valid")
```

```
}


#Removing the Kappa NextSeq run
Metadata2<-Metadata2[grep("\\KANS.*", Metadata2$Sample, invert=TRUE),]

#Applying subsetting to OTU tables
Tax2<-dplyr::select(Tax, one_of(Metadata2$Sample))

## Create grouping factor
#colnames(Tax2)==Metadata$Sample #Checking order
#New data adding line between same samples different lib prep and seq plat
Metadata2$Sample_LPSX <-
        ifelse(seq(along=(Metadata2$Sample)) %in% grep("*_HX_*", Metadata2$Sample),
               "NFHI",
        ifelse(seq(along=(Metadata2$Sample)) %in% grep("*KAHI*", Metadata2$Sample),
               "KAHI",
        ifelse(seq(along=(Metadata2$Sample)) %in% grep("*NFNS*", Metadata2$Sample),
               "NFNS",
        ifelse(seq(along=(Metadata2$Sample)) %in% grep("*NX1NS*", Metadata2$Sample),
               "NX1NS",
        ifelse(seq(along=(Metadata2$Sample)) %in% grep("*NX2NS*", Metadata2$Sample),
               "NX2NS",
        ifelse(seq(along=(Metadata2$Sample)) %in% grep("*KANS*", Metadata2$Sample),
               "KANS",
               "Other")))))) #The seq along returns true or false where the regex is
  #fulfilled and then stores the evaluated string on the new column be aware HX is
  #actually true 1-8 don't know why


## Can use different filtering strategies. 1 Bayseian approach estimating zeroes,
  #2 offset of 1 and removing all rows containing zeroes. Only minor effects observed
# f <- codaSeq.filter(ak_op, min.reads=1000, min.prop=0.005, min.occurrence=0.2,
  #samples.by.row=FALSE) #Implemented in Gloors codaSeq.filter function
#Assessing what is the max value of rows containing a zero
print("Max in rows containing a zero")
row_sub = apply(Tax2, 1, function(row) any(row !=0 ))
Tax2[row_sub,] %>% max() %>% print()
# filtering of the Counttable depending on rowSums.
Tax2 <- Tax2[rowSums(Tax2)>0,] #Removing all rows that only contains zeroes
Tax2 <- Tax2[rowSums(Tax2)>(5*ncol(Tax2)),] #Removing all rows(Species)
  #that is below an average count of 5.
# replace 0 values with an estimate using simple multiplicative replacement
Tax2 <- t(cmultRepl(t(Tax2), method="CZM", label=0))

## Maks TSS
Tax2<-sweep(Tax2, 2, colSums(Tax2), FUN="/")

Y <- Metadata2$Sample_LPSX

PCACoDa = mixOmics::pca(t(Tax2), ncomp = 10, logratio = 'ILR')#Can change CLR and ILR
#plot(PCACoDa)
```

```r
#plotIndiv(PCACoDa,
#         comp = c(1,2), # the components to plot
#         pch = 16,
#         ind.names = F,
#         group = Y,
#         col.per.group = color.mixo(1:5),
#         legend = TRUE,
#         title = 'PCA comp 1 - 2')

# Calculate the variation explained by PCoA1 and 2
# and use it to generate axis labels
eig_1 <- paste("PCA1", round(PCACoDa$explained_variance[1]*100, digits = 1), "% variance")
eig_2 <- paste("PCA2", round(PCACoDa$explained_variance[2]*100, digits = 1), "% variance")
eig_3 <- paste("PCA3", round(PCACoDa$explained_variance[3]*100, digits = 1), "% variance")
eig_4 <- paste("PCA4", round(PCACoDa$explained_variance[4]*100, digits = 1), "% variance")

##Pull out coordinates for plotting from the ca object
#Structuring to add to metadata
PCAMeta<-data.frame(Sample=PCACoDa$names$sample, PCA1=PCACoDa$variates$X[,1],
                    PCA2=PCACoDa$variates$X[,2], PCA3=PCACoDa$variates$X[,3],
                    PCA4=PCACoDa$variates$X[,4])
#Merge according to
Metadata2<-merge(Metadata2, PCAMeta, by="Sample")
#Creating column in metadata for plotting with lines between replicates
Metadata2$Sample_name_norep<-gsub("*_a|*_b|*_c", "", Metadata2$Sample_name)
#Change Storage and time to characters for plotting
Metadata2$Storage<-as.character(Metadata2$Storage)
Metadata2$Time<-as.character(Metadata2$Time)
#Change NAs in Storage to Direct
Metadata2$Storage[is.na(Metadata2$Storage)]<-"Direct"
#Change order for coloring
Metadata2$Storage<-ordered(Metadata2$Storage, levels=c("Direct", "-80", "-20", "5", "22"))

#Create plot name
pltName <- paste( 'PCA', i, sep = '' )
#create PCA
PCAList[[ pltName ]] <- ggplot(Metadata2) +
  geom_point(aes(PCA1, PCA2, color = Sample_LPSX, group = Sample, shape = Storage),
             size=5) +
  #geom_line(aes(x=PCA1, y=PCA2, group=Replicate_Boxplot)) +
  geom_line(aes(x=PCA1, y=PCA2, group=Matching_samples), size=0.1, linetype="dotted") +
  scale_color_manual(values=c("#984ea3", "#4daf4a", "#377eb8", "#ff7f00", "#993300")) +
  scale_shape_manual(values=c(8,18,15,16,17,0,1,2)) +
  ggtitle(paste("PCA", i, sep=" ")) +
  labs(colour="Processing", shape="Storage (?C)", x = eig_1, y = eig_2) +
  theme_bw() +
  theme(panel.grid.major = element_blank(), panel.grid.minor = element_blank(),
        axis.title=element_text(size=12), legend.position="none")
}
```

```
## [1] "No subsetting SubFre, all included"
## [1] "Max in rows containing a zero"
## [1] 1783518
## No. corrected values:  3
```

```
## [1] "No subsetting SubFre, all included"
## [1] "Max in rows containing a zero"
## [1] 1528286
## [1] "No subsetting SubFre, all included"
## [1] "Max in rows containing a zero"
## [1] 538286
## [1] "No subsetting SubFre, all included"
## [1] "Max in rows containing a zero"
## [1] 1581043
## No. corrected values:  24
```

```
#Draw PCA
#No validation plots
#Have the plots stored in lists
#lay <- rbind(c(1,2,3,4))
#Make pdf
#pdf(paste("PCA", "Genus", "NoVal", ".pdf", sep=""), width=24, height=12)
#grid.arrange(PCAList$PCAP1unspiked, PCAList$PCAP2unspiked, PCAList$PCAS1unspiked,
  #PCAList$PCAS2unspiked, layout_matrix = lay)
#dev.off()
#Make format for word
#png(paste("PCA", "Genus",  "NoVal", ".png", sep=""), width=1200, height=600)
#grid.arrange(PCAList$PCAP1unspiked, PCAList$PCAP2unspiked, PCAList$PCAS1unspiked,
  #PCAList$PCAS2unspiked, layout_matrix = lay)
#dev.off()


#No validation plots
#Have the plots stored in lists
lay <- rbind(c(1,2),
             c(3,4))
#Make pdf
pdf(paste("Fig2_PCASubset", ".pdf", sep=""), width=9, height=6)
grid.arrange(PCAList$PCAP1unspiked, PCAList$PCAP2unspiked, PCAList$PCAS1unspiked,
             PCAList$PCAS2unspiked, layout_matrix = lay)
dev.off()
```

```
## pdf
##   2
```

```
#Make format for word
#png(paste("PCA", "GenusTall",  "NoVal", ".png", sep=""), width=900, height=600)
#grid.arrange(PCAList$PCAP1unspiked, PCAList$PCAP2unspiked, PCAList$PCAS1unspiked,
  #PCAList$PCAS2unspiked, layout_matrix = lay)
#dev.off()

#Extract legend
#legend<-ggplot(Metadata2) +
#  geom_point(aes(PCA1, PCA2, color = Sample_LPSX, group = Sample, shape = Storage),
  #size=1.5) +
#  #geom_line(aes(x=PCA1, y=PCA2, group=Replicate_Boxplot)) +
#  geom_line(aes(x=PCA1, y=PCA2, group=Matching_samples), size=0.1, linetype="dotted") +
#  scale_color_manual(values=c("#377eb8", "#4daf4a", "#984ea3", "#ff7f00", "#e41a1c")) +
#  scale_shape_manual(values=c(8,18,15,16,17,0,1,2)) +
#  ggtitle(paste("PCA", i, sep=" ")) +
```

```
#  labs(colour="Storage / ?C", shape="Processing", x = eig_1, y = eig_2) +
#  theme_bw() +
#  theme(panel.grid.major = element_blank(), panel.grid.minor = element_blank(),
  #axis.title=element_text(size=12))
#legendplot<-get_legend(legend)
#pdf(paste("LegendPCA", ".pdf", sep=""), width=24, height=12)
#grid.arrange(legendplot)
#dev.off()
#png(paste("LegendPCA", ".png", sep=""), width=100, height=300)
#grid.arrange(legendplot)
#dev.off()

legend2<-ggplot(Metadata2) +
  geom_point(aes(PCA1, PCA2, color = Sample_LPSX, group = Sample, shape = Storage),
             size=5) +
  #geom_line(aes(x=PCA1, y=PCA2, group=Replicate_Boxplot)) +
  geom_line(aes(x=PCA1, y=PCA2, group=Matching_samples), size=0.1, linetype="dotted") +
  scale_color_manual(values=c("#984ea3", "#4daf4a", "#377eb8", "#ff7f00", "#993300")) +
  scale_shape_manual(values=c(8,18,15,16,17,0,1,2)) +
  ggtitle(paste("PCA", i, sep=" ")) +
  labs(colour="Processing", shape="Storage / ?C", x = eig_1, y = eig_2) +
  theme_bw() +
  theme(panel.grid.major = element_blank(), panel.grid.minor = element_blank(),
        axis.title=element_text(size=12), legend.position="bottom")
legendplot<-get_legend(legend2)
pdf(paste("Fig2_PCASubsetLegend", ".pdf", sep=""), width=10, height=1)
grid.arrange(legendplot)
dev.off()
```

```
## pdf
##   2
```

```
#png(paste("LegendPCAbottom", ".png", sep=""), width=600, height=25)
#grid.arrange(legendplot)
#dev.off()
```

Heatmaps

Pig feces both P1 and P2, and sewage both S1 and S2 creating Fig_3. Have added a heatmap of the negative controls S5_Fig

```
rm(list=setdiff(ls(), c("Metadata", "Feature", "Tax")))

#Subset data pig feces 1 and 2 (PF)
Subset<- "PF" #All, Allspiked, Allunspiked, PF, SW, P1, P1spiked, P1unspiked, P2,
  #P2spiked, P2unspiked, S1, S1spiked, S1unspiked, S2, S2spiked, S2unspiked

#Subset experiment, Experiment_Type. (Meaningfull combinations of subset and SubExp:
  #HX=All, FTX=P1&S1, LTX=P1&S1, LPSX=All unspiked, HXFTX=P1&S1, HXLTX=P1&S1,
  #HXLPSX=All unspiked, HXLTXLPSX=P1&S1 unspiked)
SubExp<-"LPSX" #HX, FTX, LTX, LPSX, HXFTX, HXLTX, HXLPSX, HXLTXLPSX, HXFTXLTX, All

#Subset Frozen Unfrozen
SubFre<-"Both" #Frozen, Unfrozen, Both
```

```r
#How many organisms to include in heatmap
Orgs<-30 #Write a number 30-50 seems appropiate for readability

HeatmapExplainers<-c("Sequencing_platform", "Library_preparation", "Storage","Experiment")

#Make list of annotation colors
annotation_colorsNew = list(Diagosis = c(Handling_experiment = "blue"),
                            Experiment = c(Pig_feces_1 = "#30241E",
                                           Pig_feces_2 = "#B59B80",
                                           Sewage_1 = "#33a02c",
                                           Sewage_2 = "#B2DF8A"),
                            SpikedUnspiked = c(Spiked = "Black",
                                               Unspiked = "White"),
                            Time = c("0" = "#FFFFFF", "16" = "#CCCCCC", "64" = "#666666"),
                            Storage = c(Direct = "#999999", "-80" = "#0571b0",
                                        "-20" = "#92c5de", "5" = "#f4a582",
                                        "22" = "#ca0020"),
                            Sequencing_platform = c(Hiseq = "Black", Nextseq = "White"),
                            Library_preparation = c(Kappa = "#984ea3", Nextera = "#ff7f00",
                                                    Nextflex = "#e41a1c"))

#make empty list for plots
HeatList=list()

#Removing negative and positive controls
Metadata2<-filter(Metadata, Sample_type_simple=="Sample")

#Subsetting Metadata2
if (Subset=="Allspiked") {
  Metadata2<-filter(Metadata2, SpikedUnspiked == "Spiked")
} else if (Subset=="Allunspiked") {
  Metadata2<-filter(Metadata2, SpikedUnspiked == "Unspiked")
} else if (Subset=="PF") {
  Metadata2<-filter(Metadata2, Experiment == "Pig_feces_1" | Experiment == "Pig_feces_2")
} else if (Subset=="PFspiked") {
  Metadata2<-filter(Metadata2, Sample_type == "Pig_feces_1_spiked" |
                    Sample_type == "Pig_feces_2_spiked")
} else if (Subset=="PFunspiked") {
  Metadata2<-filter(Metadata2, Sample_type == "Pig_feces_1" |
                    Sample_type == "Pig_feces_2")
} else if (Subset=="SW") {
  Metadata2<-filter(Metadata2, Experiment == "Sewage_1" | Experiment == "Sewage_2")
} else if (Subset=="SWspiked") {
  Metadata2<-filter(Metadata2, Sample_type == "Sewage_1_spiked" |
                    Sample_type == "Sewage_2_spiked")
} else if (Subset=="SWunspiked") {
  Metadata2<-filter(Metadata2, Sample_type == "Sewage_1" | Sample_type == "Sewage_2")
} else if (Subset=="P1") {
  Metadata2<-filter(Metadata2, Experiment == "Pig_feces_1")
} else if (Subset=="P1spiked") {
  Metadata2<-filter(Metadata2, Sample_type == "Pig_feces_1_spiked")
} else if (Subset=="P1unspiked") {
  Metadata2<-filter(Metadata2, Sample_type == "Pig_feces_1")
```

```r
} else if (Subset=="P2") {
  Metadata2<-filter(Metadata2, Experiment == "Pig_feces_2")
} else if (Subset=="P2spiked") {
  Metadata2<-filter(Metadata2, Sample_type == "Pig_feces_2_spiked")
} else if (Subset=="P2unspiked") {
  Metadata2<-filter(Metadata2, Sample_type == "Pig_feces_2")
} else if (Subset=="S1") {
  Metadata2<-filter(Metadata2, Experiment == "Sewage_1")
} else if (Subset=="S1spiked") {
  Metadata2<-filter(Metadata2, Sample_type == "Sewage_1_spiked")
} else if (Subset=="S1unspiked") {
  Metadata2<-filter(Metadata2, Sample_type == "Sewage_1")
} else if (Subset=="S2") {
  Metadata2<-filter(Metadata2, Experiment == "Sewage_2")
} else if (Subset=="S2spiked") {
  Metadata2<-filter(Metadata2, Sample_type == "Sewage_2_spiked")
} else if (Subset=="S2unspiked") {
  Metadata2<-filter(Metadata2, Sample_type == "Sewage_2")
} else if (Subset=="All") {
  print("No subsetting, all included")
} else {
  print("Subset defined not valid")
}


#Further subsetting Metadata2
if (SubExp=="HX") {
  Metadata2<-filter(Metadata2, Experiment_type == "Handling_experiment")
} else if (SubExp=="FTX") {
  Metadata2<-filter(Metadata2, Experiment_type == "Freeze_thaw_experiment")
} else if (SubExp=="LTX") {
  Metadata2<-filter(Metadata2, Experiment_type == "Long_term_storage_experiment")
} else if (SubExp=="LPSX") {
  Metadata22<-filter(Metadata2, Experiment_type == "Library_prep_seq_platform_experiment")
  VectorLPSX <- unique(Metadata22$Matching_samples)
  Metadata2<-filter(Metadata2, Matching_samples %in% VectorLPSX)
  rm(VectorLPSX, Metadata22)
} else if (SubExp=="HXFTX") {
  Metadata2<-filter(Metadata2, Experiment_type == "Handling_experiment" |
                    Experiment_type == "Freeze_thaw_experiment")
} else if (SubExp=="HXLTX") {
  Metadata2<-filter(Metadata2, Experiment_type == "Handling_experiment" |
                    Experiment_type == "Long_term_storage_experiment")
} else if (SubExp=="HXLPSX") {
  Metadata2<-filter(Metadata2, Experiment_type == "Handling_experiment" |
                    Experiment_type == "Library_prep_seq_platform_experiment")
} else if (SubExp=="HXLTXLPSX") {
  Metadata2<-filter(Metadata2, Experiment_type == "Handling_experiment" |
                    Experiment_type == "Long_term_storage_experiment" |
                    Experiment_type == "Library_prep_seq_platform_experiment")
} else if (SubExp=="HXFTXLTX") {
  Metadata2<-filter(Metadata2, Experiment_type == "Handling_experiment" |
                    Experiment_type == "Freeze_thaw_experiment" |
```

```
                         Experiment_type == "Long_term_storage_experiment")
} else if (SubExp=="All") {
  print("No subsetting, all included")
} else {
  print("Subset defined not valid")
}

#Further subsetting Metadata2
if (SubFre=="Frozen") {
  Metadata2<-filter(Metadata2, FrozenUnfrozenSimple == "Freezer")
} else if (SubFre=="Unfrozen") {
  Metadata2<-filter(Metadata2, FrozenUnfrozenSimple == "Unfrozen")
} else if (SubFre=="Both") {
  print("No subsetting, all included")
} else {
  print("Subset defined not valid")
}
```

```
## [1] "No subsetting, all included"
```

```
#Removing the Kappa NextSeq run
Metadata2<-Metadata2[grep("\\KANS.*", Metadata2$Sample, invert=TRUE),]

#Applying subsetting to OTU tables
Tax2<-dplyr::select(Tax, one_of(Metadata2$Sample))

#Remove orgs that are not present after subsetting. Need for doing standardize
Tax2 <- Tax2[rowSums(Tax2)>0,]

##Hellinger transformation
#TaxHeatmap <- data.frame(t(decostand(t(Tax2), method=Stand)))
TaxHeatmap <- Tax2

#Order genera, based on rowsums
TaxHeatmap <- Tax2[order(rowSums(TaxHeatmap), decreasing = T),]

#Impose a maximum number of plotted Species
TaxHeatmap <- TaxHeatmap[1:min(c(nrow(TaxHeatmap), Orgs)),]

##Remove Unknown from plot
TaxHeatmap <- TaxHeatmap[row.names(TaxHeatmap) != "Unknown",]

#The organisms clustering
OrgCluster<-amap::Dist(TaxHeatmap, method="spearman")

#filtering of the Counttable depending on rowSums.
TaxHeatmap <- TaxHeatmap[rowSums(TaxHeatmap)>0,] #Removing all rows that only contains
  #zeroes
TaxHeatmap <- TaxHeatmap[rowSums(TaxHeatmap)>(5*ncol(TaxHeatmap)),] #Removing all
  #rows(Species)  that is below an average count of 5.
## replace 0 values with an estimate using simple multiplicative replacement
#TaxHeatmap <- t(cmultRepl(t(TaxHeatmap), method="CZM", label=0))
#Maks TSS
TaxHeatmap<-sweep(TaxHeatmap, 2, colSums(TaxHeatmap), FUN="/")
```

```
rn <- row.names(TaxHeatmap)

##Then I standardized the orgs into zero mean and unit variance
TaxHeatmap <- data.frame(t(decostand(clr(t(TaxHeatmap)), method="standardize")),
                        row.names = rn)
  #Can also use scale in pheatmap, but not exactly sure what scaling that is being
  #performed

##This is what I did previously. Now I changed it to do a Hellinger transformation,
  #which is total sum scaling and then square rooting each entry.
  #Doing it on total sum scaled data since then it is also normalized according to
  #genome size so in reality I'm doing total sum scaling twice.
  #Moved to the top to do it before subsetting.
#Log transform OTU table with higher base pick one.
#TaxHeatmap <- log(TaxHeatmap+1, base = exp(200))
#Log 10 transform OTU table
#TaxHeatmap <- log10(TaxHeatmap+1)


#Make dataframe with Metadata2 for heatmap annotation
colannodf <- data.frame(Metadata2[, HeatmapExplainers], row.names = Metadata2$Sample)
#Depending on if time and Storage are part of the plots make them into characters
#colannodf$Time <- as.character(colannodf$Time)
colannodf$Storage <- as.character(colannodf$Storage)
colannodf$Storage[is.na(colannodf$Storage)] <- "Direct" #Change NA to Direct

#Calculate sample-distance matrix
#Note, that this is done on the full set, not just the shown. Makes sense eventhough not
  #show in heatmap they can be in the clustering calculations, this also means samples
  #can look more similar in the heatmap but not cluster as closely. Also calculates on
  #the not log transformed data.
#filtering of the Counttable depending on rowSums.
Tax2 <- Tax2[rowSums(Tax2)>0,] #Removing all rows that only contains zeroes
Tax2 <- Tax2[rowSums(Tax2)>(5*ncol(Tax2)),] #Removing all rows(Species)
  #that is below an average count of 5.
# replace 0 values with an estimate using simple multiplicative replacement
Tax2 <- t(cmultRepl(t(Tax2), method="CZM", label=0))
```

```
## No. corrected values:  21
```

```
#Maks TSS
Tax2<-sweep(Tax2, 2, colSums(Tax2), FUN="/")
#Calculate sample-distance matrix
#Note, that this is done on the full set, not just the shown. Makes sense eventhough not
  #show in heatmap they can be in the clustering calculations, this also means samples
  #can look more similar in the heatmap but not cluster as closely. Also calculates on
  #the not log transformed data.
distmatrix_Species <- vegdist(ilr(t(Tax2)), method="euclidean") #Previously
  #vegdist(decostand(t(Tax2), method="hellinger"), method="bray")



#Draw the heatmap
plot<-pheatmap(TaxHeatmap,
```

```
color = colorRampPalette(rev(brewer.pal(n = 7, name = "Blues")))(100),
margins=c(8,8),
treeheight_row = 100,
treeheight_col = 100,
scale="none",
clustering_distance_cols = distmatrix_Species,
clustering_distance_rows = OrgCluster,
annotation_col = colannodf,
cutree_cols = 2,
show_colnames = FALSE,
cellwidth=5,
cellheight=4,
fontsize=6,
annotation_colors = annotation_colorsNew[1:7],
annotation_legend = TRUE)
```

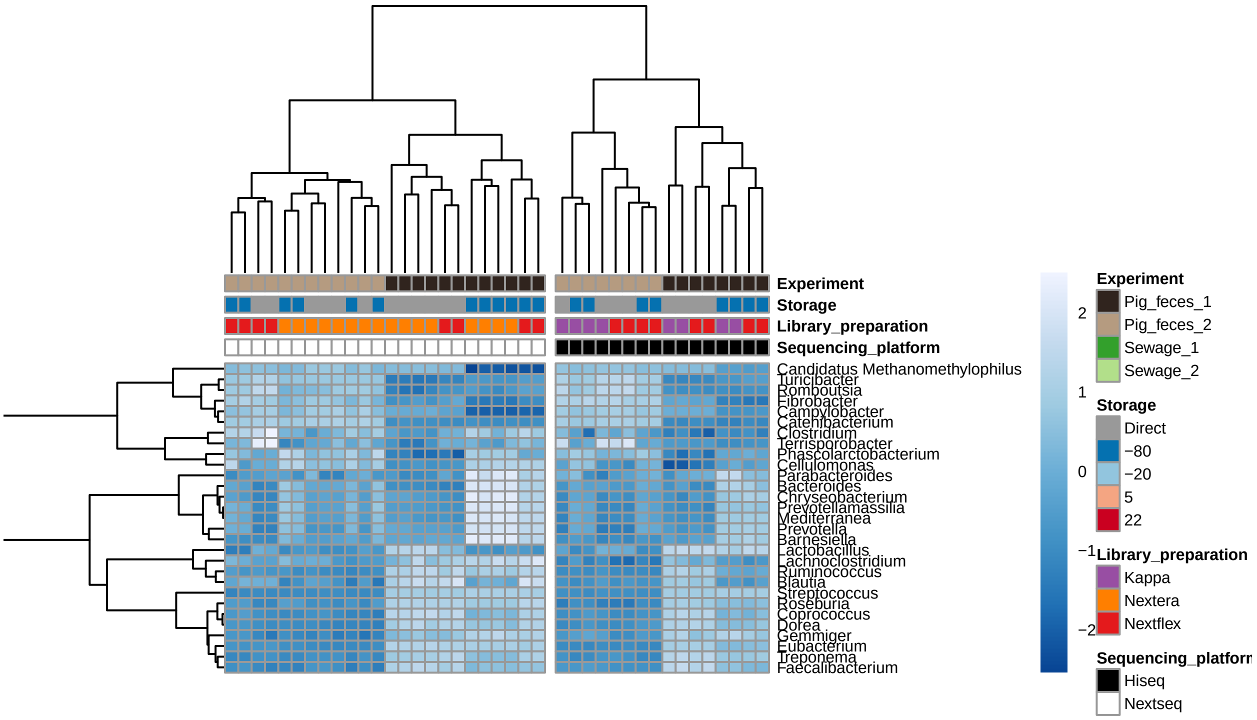


```
HeatList[[Subset]] = plot[[4]]
```

```
#Subset data sewage 1 and 2 (SW)
Subset<- "SW" #All, Allspiked, Allunspiked, PF, SW, P1, P1spiked, P1unspiked, P2,
  #P2spiked, P2unspiked, S1, S1spiked, S1unspiked, S2, S2spiked, S2unspiked
```

```r
#Removing negative and positive controls
Metadata2<-filter(Metadata, Sample_type_simple=="Sample")

#Subsetting Metadata2
if (Subset=="Allspiked") {
  Metadata2<-filter(Metadata2, SpikedUnspiked == "Spiked")
} else if (Subset=="Allunspiked") {
  Metadata2<-filter(Metadata2, SpikedUnspiked == "Unspiked")
} else if (Subset=="PF") {
  Metadata2<-filter(Metadata2, Experiment == "Pig_feces_1" | Experiment == "Pig_feces_2")
} else if (Subset=="PFspiked") {
  Metadata2<-filter(Metadata2, Sample_type == "Pig_feces_1_spiked" |
                      Sample_type == "Pig_feces_2_spiked")
} else if (Subset=="PFunspiked") {
  Metadata2<-filter(Metadata2, Sample_type == "Pig_feces_1" |
                      Sample_type == "Pig_feces_2")
} else if (Subset=="SW") {
  Metadata2<-filter(Metadata2, Experiment == "Sewage_1" | Experiment == "Sewage_2")
} else if (Subset=="SWspiked") {
  Metadata2<-filter(Metadata2, Sample_type == "Sewage_1_spiked" |
                      Sample_type == "Sewage_2_spiked")
} else if (Subset=="SWunspiked") {
  Metadata2<-filter(Metadata2, Sample_type == "Sewage_1" | Sample_type == "Sewage_2")
} else if (Subset=="P1") {
  Metadata2<-filter(Metadata2, Experiment == "Pig_feces_1")
} else if (Subset=="P1spiked") {
  Metadata2<-filter(Metadata2, Sample_type == "Pig_feces_1_spiked")
} else if (Subset=="P1unspiked") {
  Metadata2<-filter(Metadata2, Sample_type == "Pig_feces_1")
} else if (Subset=="P2") {
  Metadata2<-filter(Metadata2, Experiment == "Pig_feces_2")
} else if (Subset=="P2spiked") {
  Metadata2<-filter(Metadata2, Sample_type == "Pig_feces_2_spiked")
} else if (Subset=="P2unspiked") {
  Metadata2<-filter(Metadata2, Sample_type == "Pig_feces_2")
} else if (Subset=="S1") {
  Metadata2<-filter(Metadata2, Experiment == "Sewage_1")
} else if (Subset=="S1spiked") {
  Metadata2<-filter(Metadata2, Sample_type == "Sewage_1_spiked")
} else if (Subset=="S1unspiked") {
  Metadata2<-filter(Metadata2, Sample_type == "Sewage_1")
} else if (Subset=="S2") {
  Metadata2<-filter(Metadata2, Experiment == "Sewage_2")
} else if (Subset=="S2spiked") {
  Metadata2<-filter(Metadata2, Sample_type == "Sewage_2_spiked")
} else if (Subset=="S2unspiked") {
  Metadata2<-filter(Metadata2, Sample_type == "Sewage_2")
} else if (Subset=="All") {
  print("No subsetting, all included")
} else {
  print("Subset defined not valid")
}
```

```r
#Further subsetting Metadata2
if (SubExp=="HX") {
  Metadata2<-filter(Metadata2, Experiment_type == "Handling_experiment")
} else if (SubExp=="FTX") {
  Metadata2<-filter(Metadata2, Experiment_type == "Freeze_thaw_experiment")
} else if (SubExp=="LTX") {
  Metadata2<-filter(Metadata2, Experiment_type == "Long_term_storage_experiment")
} else if (SubExp=="LPSX") {
  Metadata22<-filter(Metadata2, Experiment_type == "Library_prep_seq_platform_experiment")
  VectorLPSX <- unique(Metadata22$Matching_samples)
  Metadata2<-filter(Metadata2, Matching_samples %in% VectorLPSX)
  rm(VectorLPSX, Metadata22)
} else if (SubExp=="HXFTX") {
  Metadata2<-filter(Metadata2, Experiment_type == "Handling_experiment" |
                    Experiment_type == "Freeze_thaw_experiment")
} else if (SubExp=="HXLTX") {
  Metadata2<-filter(Metadata2, Experiment_type == "Handling_experiment" |
                    Experiment_type == "Long_term_storage_experiment")
} else if (SubExp=="HXLPSX") {
  Metadata2<-filter(Metadata2, Experiment_type == "Handling_experiment" |
                    Experiment_type == "Library_prep_seq_platform_experiment")
} else if (SubExp=="HXLTXLPSX") {
  Metadata2<-filter(Metadata2, Experiment_type == "Handling_experiment" |
                    Experiment_type == "Long_term_storage_experiment" |
                    Experiment_type == "Library_prep_seq_platform_experiment")
} else if (SubExp=="HXFTXLTX") {
  Metadata2<-filter(Metadata2, Experiment_type == "Handling_experiment" |
                    Experiment_type == "Freeze_thaw_experiment" |
                    Experiment_type == "Long_term_storage_experiment")
} else if (SubExp=="All") {
  print("No subsetting, all included")
} else {
  print("Subset defined not valid")
}

#Further subsetting Metadata2
if (SubFre=="Frozen") {
  Metadata2<-filter(Metadata2, FrozenUnfrozenSimple == "Freezer")
} else if (SubFre=="Unfrozen") {
  Metadata2<-filter(Metadata2, FrozenUnfrozenSimple == "Unfrozen")
} else if (SubFre=="Both") {
  print("No subsetting, all included")
} else {
  print("Subset defined not valid")
}
```

```
## [1] "No subsetting, all included"
```

```r
#Removing the Kappa NextSeq run
Metadata2<-Metadata2[grep("\\KANS.*", Metadata2$Sample, invert=TRUE),]

#Applying subsetting to OTU tables
Tax2<-dplyr::select(Tax, one_of(Metadata2$Sample))
```

```r
#Remove orgs that are not present after subsetting. Need for doing standardize
Tax2 <- Tax2[rowSums(Tax2)>0,]

##Hellinger transformation
#TaxHeatmap <- data.frame(t(decostand(t(Tax2), method=Stand)))
TaxHeatmap <- Tax2

#Order genera, based on rowsums
TaxHeatmap <- Tax2[order(rowSums(TaxHeatmap), decreasing = T),]

#Impose a maximum number of plotted Species
TaxHeatmap <- TaxHeatmap[1:min(c(nrow(TaxHeatmap), Orgs)),]

##Remove Unknown from plot
TaxHeatmap <- TaxHeatmap[row.names(TaxHeatmap) != "Unknown",]

#The organisms clustering
OrgCluster<-amap::Dist(TaxHeatmap, method="spearman")

##Then I standardized the orgs into zero mean and unit variance
TaxHeatmap <- data.frame(t(decostand(t(TaxHeatmap), method="standardize"))) #Can also use
  #scale in pheatmap, but not exactly sure what scaling that is being performed


#Make dataframe with Metadata2 for heatmap annotation
colannodf <- data.frame(Metadata2[, HeatmapExplainers], row.names = Metadata2$Sample)
#Depending on if time and Storage are part of the plots make them into characters
#colannodf$Time <- as.character(colannodf$Time)
colannodf$Storage <- as.character(colannodf$Storage)
colannodf$Storage[is.na(colannodf$Storage)] <- "Direct" #Change NA to Direct

#Calculate sample-distance matrix
#Note, that this is done on the full set, not just the shown. Makes sense eventhough not
  #show in heatmap they can be in the clustering calculations, this also means samples
  #can look more similar in the heatmap but not cluster as closely. Also calculates on
  #the not log transformed data.
#filtering of the Counttable depending on rowSums.
Tax2 <- Tax2[rowSums(Tax2)>0,] #Removing all rows that only contains zeroes
Tax2 <- Tax2[rowSums(Tax2)>(5*ncol(Tax2)),] #Removing all rows(Species)  that is below an
  #average count of 5.
# replace 0 values with an estimate using simple multiplicative replacement
Tax2 <- t(cmultRepl(t(Tax2), method="CZM", label=0))
```

```
## No. corrected values:  140
```

```r
#Maks TSS
Tax2<-sweep(Tax2, 2, colSums(Tax2), FUN="/")
#Calculate sample-distance matrix
#Note, that this is done on the full set, not just the shown. Makes sense eventhough not
  #show in heatmap they can be in the clustering calculations, this also means samples
  #can look more similar in the heatmap but not cluster as closely. Also calculates on
  #the not log transformed data.
distmatrix_Species <- vegdist(ilr(t(Tax2)), method="euclidean") #Previously
  #vegdist(decostand(t(Tax2), method="hellinger"), method="bray")
```

```
#Draw the heatmap
plot<-pheatmap(TaxHeatmap,
         color = colorRampPalette(rev(brewer.pal(n = 7, name = "Blues")))(100),
         margins=c(8,8),
         treeheight_row = 100,
         treeheight_col = 100,
         scale="none",
         clustering_distance_cols = distmatrix_Species,
         clustering_distance_rows = OrgCluster,
         annotation_col = colannodf,
         cutree_cols = 2,
         show_colnames = FALSE,
         cellwidth=5,
         cellheight=4,
         fontsize=6,
         annotation_colors = annotation_colorsNew[1:7],
         annotation_legend = TRUE)
```

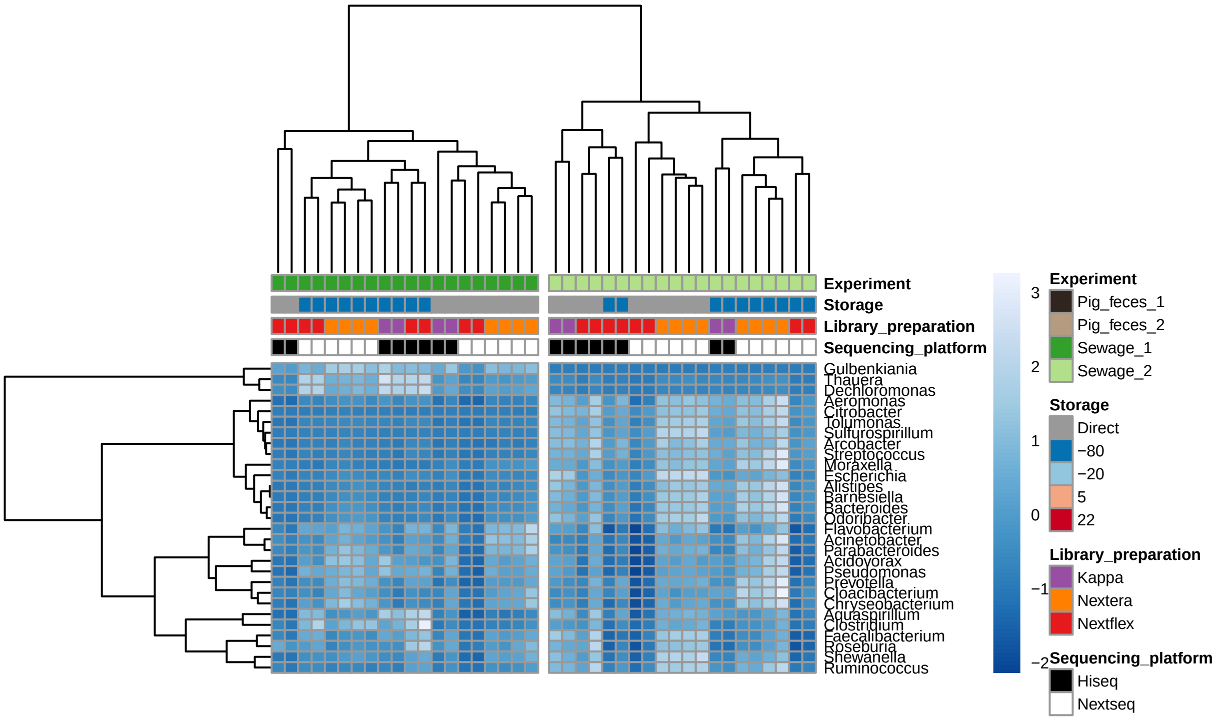


```
HeatList[[Subset]] = plot[[4]]

#grid.arrange(HeatList$PF)
#grid.arrange(HeatList$SW)

#Have the plots stored in lists
lay <- rbind(c(1,2))
```

```
pdf(paste("Fig3_Heatmap", ".pdf", sep=""), width=15, height=5)
grid.arrange(HeatList$PF, HeatList$SW, layout_matrix = lay)
dev.off()
```

```
## pdf
##   2
```

```
#Make heatmap of negative controls
#Subset data sewage 1 and 2 (SW)
Subset<- "All" #All, Allspiked, Allunspiked, PF, SW, P1, P1spiked, P1unspiked, P2,
  #P2spiked, P2unspiked, S1, S1spiked, S1unspiked, S2, S2spiked, S2unspiked

#Selecting negative controls
Metadata2<-filter(Metadata, Sample_type_simple=="Negative_control")

#Subsetting Metadata2
if (Subset=="Allspiked") {
  Metadata2<-filter(Metadata2, SpikedUnspiked == "Spiked")
} else if (Subset=="Allunspiked") {
  Metadata2<-filter(Metadata2, SpikedUnspiked == "Unspiked")
} else if (Subset=="PF") {
  Metadata2<-filter(Metadata2, Experiment == "Pig_feces_1" |
                     Experiment == "Pig_feces_2")
} else if (Subset=="PFspiked") {
  Metadata2<-filter(Metadata2, Sample_type == "Pig_feces_1_spiked" |
                     Sample_type == "Pig_feces_2_spiked")
} else if (Subset=="PFunspiked") {
  Metadata2<-filter(Metadata2, Sample_type == "Pig_feces_1" |
                     Sample_type == "Pig_feces_2")
} else if (Subset=="SW") {
  Metadata2<-filter(Metadata2, Experiment == "Sewage_1" | Experiment == "Sewage_2")
} else if (Subset=="SWspiked") {
  Metadata2<-filter(Metadata2, Sample_type == "Sewage_1_spiked" |
                     Sample_type == "Sewage_2_spiked")
} else if (Subset=="SWunspiked") {
  Metadata2<-filter(Metadata2, Sample_type == "Sewage_1" | Sample_type == "Sewage_2")
} else if (Subset=="P1") {
  Metadata2<-filter(Metadata2, Experiment == "Pig_feces_1")
} else if (Subset=="P1spiked") {
  Metadata2<-filter(Metadata2, Sample_type == "Pig_feces_1_spiked")
} else if (Subset=="P1unspiked") {
  Metadata2<-filter(Metadata2, Sample_type == "Pig_feces_1")
} else if (Subset=="P2") {
  Metadata2<-filter(Metadata2, Experiment == "Pig_feces_2")
} else if (Subset=="P2spiked") {
  Metadata2<-filter(Metadata2, Sample_type == "Pig_feces_2_spiked")
} else if (Subset=="P2unspiked") {
  Metadata2<-filter(Metadata2, Sample_type == "Pig_feces_2")
} else if (Subset=="S1") {
  Metadata2<-filter(Metadata2, Experiment == "Sewage_1")
} else if (Subset=="S1spiked") {
  Metadata2<-filter(Metadata2, Sample_type == "Sewage_1_spiked")
} else if (Subset=="S1unspiked") {
  Metadata2<-filter(Metadata2, Sample_type == "Sewage_1")
```

```r
} else if (Subset=="S2") {
  Metadata2<-filter(Metadata2, Experiment == "Sewage_2")
} else if (Subset=="S2spiked") {
  Metadata2<-filter(Metadata2, Sample_type == "Sewage_2_spiked")
} else if (Subset=="S2unspiked") {
  Metadata2<-filter(Metadata2, Sample_type == "Sewage_2")
} else if (Subset=="All") {
  print("No subsetting, all included")
} else {
  print("Subset defined not valid")
}
```

```
## [1] "No subsetting, all included"
```

```r
#Further subsetting Metadata2
if (SubExp=="HX") {
  Metadata2<-filter(Metadata2, Experiment_type == "Handling_experiment")
} else if (SubExp=="FTX") {
  Metadata2<-filter(Metadata2, Experiment_type == "Freeze_thaw_experiment")
} else if (SubExp=="LTX") {
  Metadata2<-filter(Metadata2, Experiment_type == "Long_term_storage_experiment")
} else if (SubExp=="LPSX") {
  Metadata22<-filter(Metadata2, Experiment_type == "Library_prep_seq_platform_experiment")
  VectorLPSX <- unique(Metadata22$Matching_samples)
  Metadata2<-filter(Metadata2, Matching_samples %in% VectorLPSX)
  rm(VectorLPSX, Metadata22)
} else if (SubExp=="HXFTX") {
  Metadata2<-filter(Metadata2, Experiment_type == "Handling_experiment" |
                    Experiment_type == "Freeze_thaw_experiment")
} else if (SubExp=="HXLTX") {
  Metadata2<-filter(Metadata2, Experiment_type == "Handling_experiment" |
                    Experiment_type == "Long_term_storage_experiment")
} else if (SubExp=="HXLPSX") {
  Metadata2<-filter(Metadata2, Experiment_type == "Handling_experiment" |
                    Experiment_type == "Library_prep_seq_platform_experiment")
} else if (SubExp=="HXLTXLPSX") {
  Metadata2<-filter(Metadata2, Experiment_type == "Handling_experiment" |
                    Experiment_type == "Long_term_storage_experiment" |
                    Experiment_type == "Library_prep_seq_platform_experiment")
} else if (SubExp=="HXFTXLTX") {
  Metadata2<-filter(Metadata2, Experiment_type == "Handling_experiment" |
                    Experiment_type == "Freeze_thaw_experiment" |
                    Experiment_type == "Long_term_storage_experiment")
} else if (SubExp=="All") {
  print("No subsetting, all included")
} else {
  print("Subset defined not valid")
}

#Further subsetting Metadata2
if (SubFre=="Frozen") {
  Metadata2<-filter(Metadata2, FrozenUnfrozenSimple == "Freezer")
} else if (SubFre=="Unfrozen") {
  Metadata2<-filter(Metadata2, FrozenUnfrozenSimple == "Unfrozen")
```

```
} else if (SubFre=="Both") {
  print("No subsetting, all included")
} else {
  print("Subset defined not valid")
}
```

```
## [1] "No subsetting, all included"
```

```
#Removing the Kappa NextSeq run
Metadata2<-Metadata2[grep("\\KANS.*", Metadata2$Sample, invert=TRUE),]

#Applying subsetting to OTU tables
Tax2<-dplyr::select(Tax, one_of(Metadata2$Sample))

#Remove orgs that are not present after subsetting. Need for doing standardize
Tax2 <- Tax2[rowSums(Tax2)>0,]
#Add row names to samples to manually search the organisms observed in sPLS-DA
searchMan <- add_rownames(Tax2, "Orgs")

##Hellinger transformation
#TaxHeatmap <- data.frame(t(decostand(t(Tax2), method=Stand)))
TaxHeatmap <- Tax2

#Order genera, based on rowsums
TaxHeatmap <- Tax2[order(rowSums(TaxHeatmap), decreasing = T),]

#Impose a maximum number of plotted Species
TaxHeatmap <- TaxHeatmap[1:min(c(nrow(TaxHeatmap), Orgs)),]

##Remove Unknown from plot
TaxHeatmap <- TaxHeatmap[row.names(TaxHeatmap) != "Unknown",]

#The organisms clustering
OrgCluster<-amap::Dist(TaxHeatmap, method="spearman")

##Then I standardized the orgs into zero mean and unit variance
TaxHeatmap <- data.frame(t(decostand(t(TaxHeatmap), method="standardize"))) #Can also use
  #scale in pheatmap, but not exactly sure what scaling that is being performed


#Make dataframe with Metadata2 for heatmap annotation
colannodf <- data.frame(Metadata2[, HeatmapExplainers], row.names = Metadata2$Sample)
#Depending on if time and Storage are part of the plots make them into characters
#colannodf$Time <- as.character(colannodf$Time)
colannodf$Storage <- as.character(colannodf$Storage)
colannodf$Storage[is.na(colannodf$Storage)] <- "Direct" #Change NA to Direct

#Calculate sample-distance matrix
#Note, that this is done on the full set, not just the shown. Makes sense eventhough not
  #show in heatmap they can be in the clustering calculations, this also means samples can
  #look more similar in the heatmap but not cluster as closely. Also calculates on the not
  #log transformed data.
#filtering of the Counttable depending on rowSums.
Tax2 <- Tax2[rowSums(Tax2)>0,] #Removing all rows that only contains zeroes
```

```r
Tax2 <- Tax2[rowSums(Tax2)>(5*ncol(Tax2)),] #Removing all rows(Species)  that is below an
  #average count of 5.
# replace 0 values with an estimate using simple multiplicative replacement
Tax2 <- t(cmultRepl(t(Tax2), method="CZM", label=0))
```

```
## No. corrected values:  42
```

```r
#Maks TSS
Tax2<-sweep(Tax2, 2, colSums(Tax2), FUN="/")
#Calculate sample-distance matrix
#Note, that this is done on the full set, not just the shown. Makes sense eventhough not
  #show in heatmap they can be in the clustering calculations, this also means samples can
  #look more similar in the heatmap but not cluster as closely. Also calculates on the not
  #log transformed data.
distmatrix_Species <- vegdist(ilr(t(Tax2)), method="euclidean") #Previously
  #vegdist(decostand(t(Tax2), method="hellinger"), method="bray")

#Draw the heatmap
plot<-pheatmap(TaxHeatmap,
         color = colorRampPalette(rev(brewer.pal(n = 7, name = "Blues")))(100),
         margins=c(8,8),
         treeheight_row = 100,
         treeheight_col = 100,
         scale="none",
         clustering_distance_cols = distmatrix_Species,
         clustering_distance_rows = OrgCluster,
         annotation_col = colannodf,
         cutree_cols = 2,
         show_colnames = FALSE,
         cellwidth=5,
         cellheight=4,
         fontsize=6,
         annotation_colors = annotation_colorsNew[1:7],
         annotation_legend = TRUE)
```

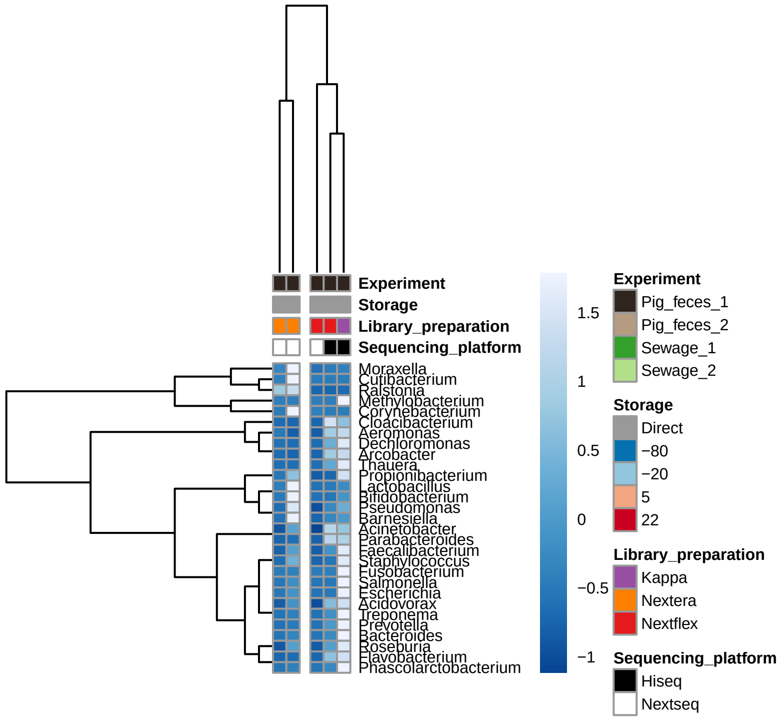


```
HeatList[[Subset]] = plot[[4]]

#grid.arrange(HeatList$PF)
#grid.arrange(HeatList$SW)

#Have the plots stored in lists
lay <- rbind(c(1))
pdf(paste("S5_Fig_HeatmapNegCon", ".pdf", sep=""), width=7.5, height=5)
grid.arrange(HeatList$All, layout_matrix = lay)
dev.off()
```

```
## pdf
##   2
```

Boxplots of distances between samples

Grouped according to different parameters (Sample, Storage, Library preparation, sequencing platform, Replicates) to create Fig_1

```
rm(list=setdiff(ls(), c("Metadata", "Feature", "Tax")))

Subset <- "All" #All, Allspiked, Allunspiked, PF, SW, P1, P1spiked, P1unspiked, P2,
  #P2spiked, P2unspiked, S1, S1spiked, S1unspiked, S2, S2spiked, S2unspiked

#Subset experiment, Experiment_Type. (Meaningfull combinations of subset and SubExp:
  #HX=All, FTX=P1&S1, LTX=P1&S1, LPSX=All unspiked, HXFTX=P1&S1, HXLTX=P1&S1,
  #HXLPSX=All unspiked, HXLTXLPSX=P1&S1 unspiked)
```

```r
SubExp<-"LPSX" #HX, FTX, LTX, LPSX, HXFTX, HXLTX, HXLPSX, HXLTXLPSX, HXFTXLTX, All

#Subset Frozen Unfrozen
SubFre<-"Both" #Frozen, Unfrozen, Both

#Removing negative and positive controls
Metadata2<-filter(Metadata, Sample_type_simple=="Sample")

#Subsetting Metadata2
if (Subset=="Allspiked") {
  Metadata2<-filter(Metadata2, SpikedUnspiked == "Spiked")
} else if (Subset=="Allunspiked") {
  Metadata2<-filter(Metadata2, SpikedUnspiked == "Unspiked")
} else if (Subset=="PF") {
  Metadata2<-filter(Metadata2, Experiment == "Pig_feces_1" | Experiment == "Pig_feces_2")
} else if (Subset=="PFspiked") {
  Metadata2<-filter(Metadata2, Sample_type == "Pig_feces_1_spiked" |
                    Sample_type == "Pig_feces_2_spiked")
} else if (Subset=="PFunspiked") {
  Metadata2<-filter(Metadata2, Sample_type == "Pig_feces_1" |
                    Sample_type == "Pig_feces_2")
} else if (Subset=="SW") {
  Metadata2<-filter(Metadata2, Experiment == "Sewage_1" | Experiment == "Sewage_2")
} else if (Subset=="SWspiked") {
  Metadata2<-filter(Metadata2, Sample_type == "Sewage_1_spiked" |
                    Sample_type == "Sewage_2_spiked")
} else if (Subset=="SWunspiked") {
  Metadata2<-filter(Metadata2, Sample_type == "Sewage_1" | Sample_type == "Sewage_2")
} else if (Subset=="P1") {
  Metadata2<-filter(Metadata2, Experiment == "Pig_feces_1")
} else if (Subset=="P1spiked") {
  Metadata2<-filter(Metadata2, Sample_type == "Pig_feces_1_spiked")
} else if (Subset=="P1unspiked") {
  Metadata2<-filter(Metadata2, Sample_type == "Pig_feces_1")
} else if (Subset=="P2") {
  Metadata2<-filter(Metadata2, Experiment == "Pig_feces_2")
} else if (Subset=="P2spiked") {
  Metadata2<-filter(Metadata2, Sample_type == "Pig_feces_2_spiked")
} else if (Subset=="P2unspiked") {
  Metadata2<-filter(Metadata2, Sample_type == "Pig_feces_2")
} else if (Subset=="S1") {
  Metadata2<-filter(Metadata2, Experiment == "Sewage_1")
} else if (Subset=="S1spiked") {
  Metadata2<-filter(Metadata2, Sample_type == "Sewage_1_spiked")
} else if (Subset=="S1unspiked") {
  Metadata2<-filter(Metadata2, Sample_type == "Sewage_1")
} else if (Subset=="S2") {
  Metadata2<-filter(Metadata2, Experiment == "Sewage_2")
} else if (Subset=="S2spiked") {
  Metadata2<-filter(Metadata2, Sample_type == "Sewage_2_spiked")
} else if (Subset=="S2unspiked") {
  Metadata2<-filter(Metadata2, Sample_type == "Sewage_2")
} else if (Subset=="All") {
```

```r
  print("No subsetting, all included")
} else {
  print("Subset defined not valid")
}
```

```
## [1] "No subsetting, all included"
```

```r
#Further subsetting Metadata2
if (SubExp=="HX") {
  Metadata2<-filter(Metadata2, Experiment_type == "Handling_experiment")
} else if (SubExp=="FTX") {
  Metadata2<-filter(Metadata2, Experiment_type == "Freeze_thaw_experiment")
} else if (SubExp=="LTX") {
  Metadata2<-filter(Metadata2, Experiment_type == "Long_term_storage_experiment")
} else if (SubExp=="LPSX") {
  Metadata22<-filter(Metadata2, Experiment_type == "Library_prep_seq_platform_experiment")
  VectorLPSX <- unique(Metadata22$Matching_samples)
  Metadata2<-filter(Metadata2, Matching_samples %in% VectorLPSX)
  rm(VectorLPSX, Metadata22)
} else if (SubExp=="HXFTX") {
  Metadata2<-filter(Metadata2, Experiment_type == "Handling_experiment" |
                      Experiment_type == "Freeze_thaw_experiment")
} else if (SubExp=="HXLTX") {
  Metadata2<-filter(Metadata2, Experiment_type == "Handling_experiment" |
                      Experiment_type == "Long_term_storage_experiment")
} else if (SubExp=="HXLPSX") {
  Metadata2<-filter(Metadata2, Experiment_type == "Handling_experiment" |
                      Experiment_type == "Library_prep_seq_platform_experiment")
} else if (SubExp=="HXLTXLPSX") {
  Metadata2<-filter(Metadata2, Experiment_type == "Handling_experiment" |
                      Experiment_type == "Long_term_storage_experiment" |
                      Experiment_type == "Library_prep_seq_platform_experiment")
} else if (SubExp=="HXFTXLTX") {
  Metadata2<-filter(Metadata2, Experiment_type == "Handling_experiment" |
                      Experiment_type == "Freeze_thaw_experiment" |
                      Experiment_type == "Long_term_storage_experiment")
} else if (SubExp=="All") {
  print("No subsetting, all included")
} else {
  print("Subset defined not valid")
}

#Further subsetting Metadata2
if (SubFre=="Frozen") {
  Metadata2<-filter(Metadata2, FrozenUnfrozenSimple == "Freezer")
} else if (SubFre=="Unfrozen") {
  Metadata2<-filter(Metadata2, FrozenUnfrozenSimple == "Unfrozen")
} else if (SubFre=="Both") {
  print("No subsetting, all included")
} else {
  print("Subset defined not valid")
}
```

```
## [1] "No subsetting, all included"
```

```r
#Removing the Kappa NextSeq run
Metadata2<-Metadata2[grep("\\KANS.*", Metadata2$Sample, invert=TRUE),]

#Applying subsetting to OTU tables
Tax2<-dplyr::select(Tax, one_of(Metadata2$Sample))

#Calculate sample-distance matrix
#Note, that this is done on the full set, not just the shown. Makes sense eventhough not
  #show in heatmap they can be in the clustering calculations, this also means samples can
  #look more similar in the heatmap but not cluster as closely. Also calculates on the not
  #log transformed data.
#filtering of the Counttable depending on rowSums.
Tax2 <- Tax2[rowSums(Tax2)>0,] #Removing all rows that only contains zeroes
Tax2 <- Tax2[rowSums(Tax2)>(5*ncol(Tax2)),] #Removing all rows(Species)  that is below an average count
# replace 0 values with an estimate using simple multiplicative replacement
Tax2 <- t(cmultRepl(t(Tax2), method="CZM", label=0))
```

```
## No. corrected values:  4863
```

```r
#Maks TSS
Tax2<-sweep(Tax2, 2, colSums(Tax2), FUN="/")
#Calculate sample-distance matrix
#Note, that this is done on the full set, not just the shown. Makes sense eventhough not
  #show in heatmap they can be in the clustering calculations, this also means samples can
  #look more similar in the heatmap but not cluster as closely. Also calculates on the not
  #log transformed data.
distmatrix <- vegdist(ilr(t(Tax2)), method="euclidean") #Previously
  #vegdist(decostand(t(Tax2), method="hellinger"), method="bray")

#Make distances into matrix
distmatrix<-as.matrix(distmatrix)

#Contains all values except the ones equal to 0
meltdist<-subset(melt(distmatrix), value!=0)
rm(distmatrix)

##Extract info on specific comparisons
#Distance between pig feces and sewage
#Select comparisons where PF is part of the first variable
PFvsSW<-filter(meltdist, grepl('P1|P2', Var1))
#From this exclude comparisons to other pig feces samples
PFvsSW<-filter(PFvsSW, !grepl('P1|P2', Var2))
#Histogram to look at distribution
hist(PFvsSW$value)
```

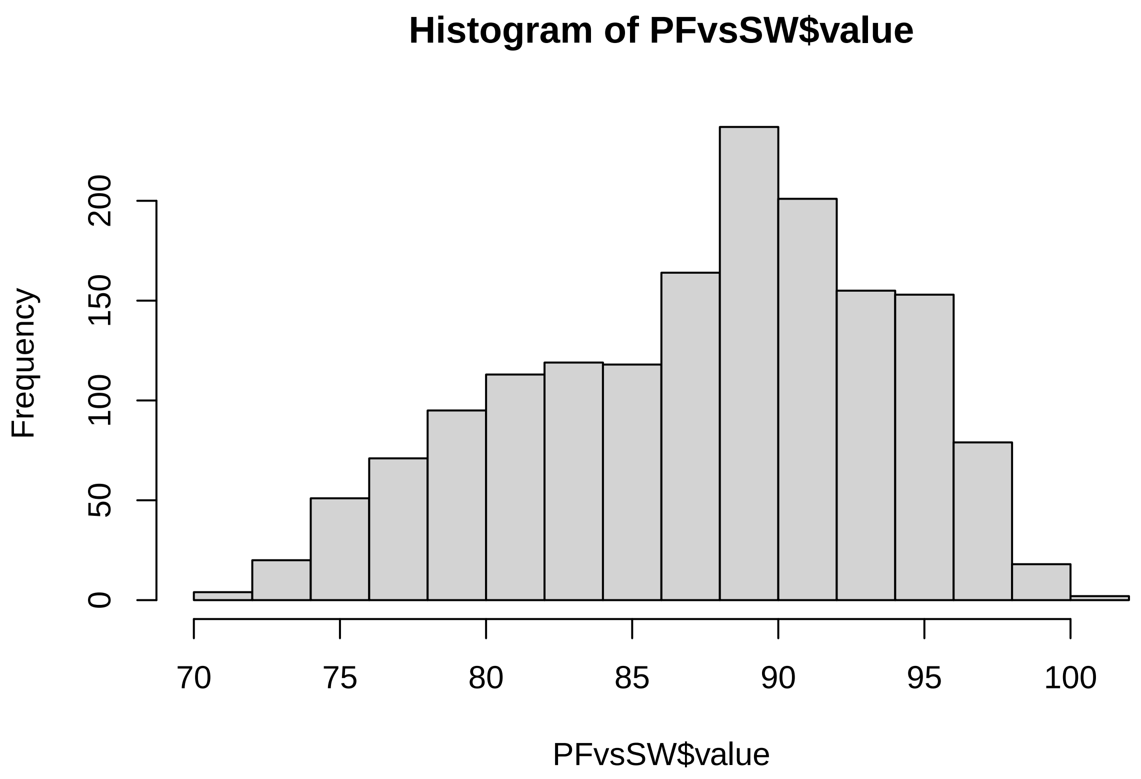


```
#Add jitter column
PFvsSW<-mutate(PFvsSW, jitter="PFvsSW")
#Add Comparison column
PFvsSW<-mutate(PFvsSW, Comparison="PFvsSW")


#Distance between the pig feces samples
#Select comparisons where P1 is part of the first variable
P1vsP2<-filter(meltdist, grepl('P1', Var1))
#From this include comparisons to P2
P1vsP2<-filter(P1vsP2, grepl('P2', Var2))
#Histogram to look at distribution
hist(P1vsP2$value)
```

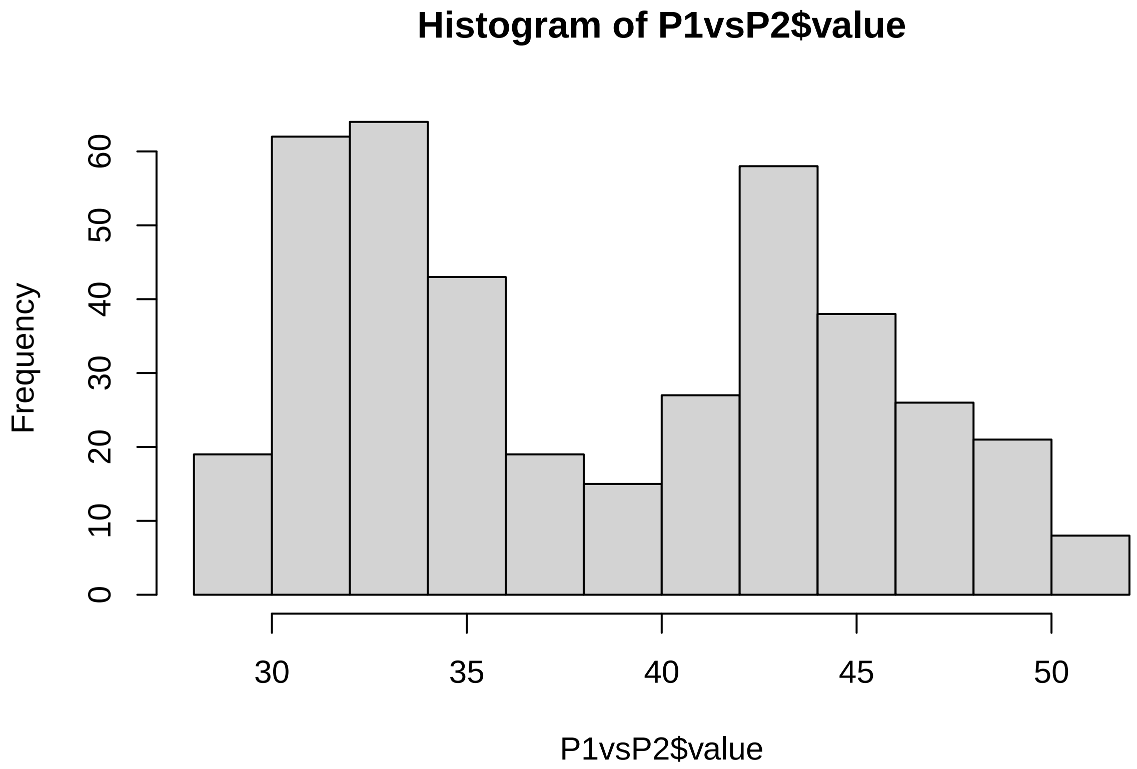


```
#Add jitter naming column
P1vsP2<-mutate(P1vsP2, jitter="P1vsP2")
#Add Comparison column
P1vsP2<-mutate(P1vsP2, Comparison="P1vsP2")


#Distance between the sewage samples
#Select comparisons where S1 is part of the first variable
S1vsS2<-filter(meltdist, grepl('S1', Var1))
#From this include comparisons to S2
S1vsS2<-filter(S1vsS2, grepl('S2', Var2))
#Histogram to look at distribution
hist(S1vsS2$value)
```

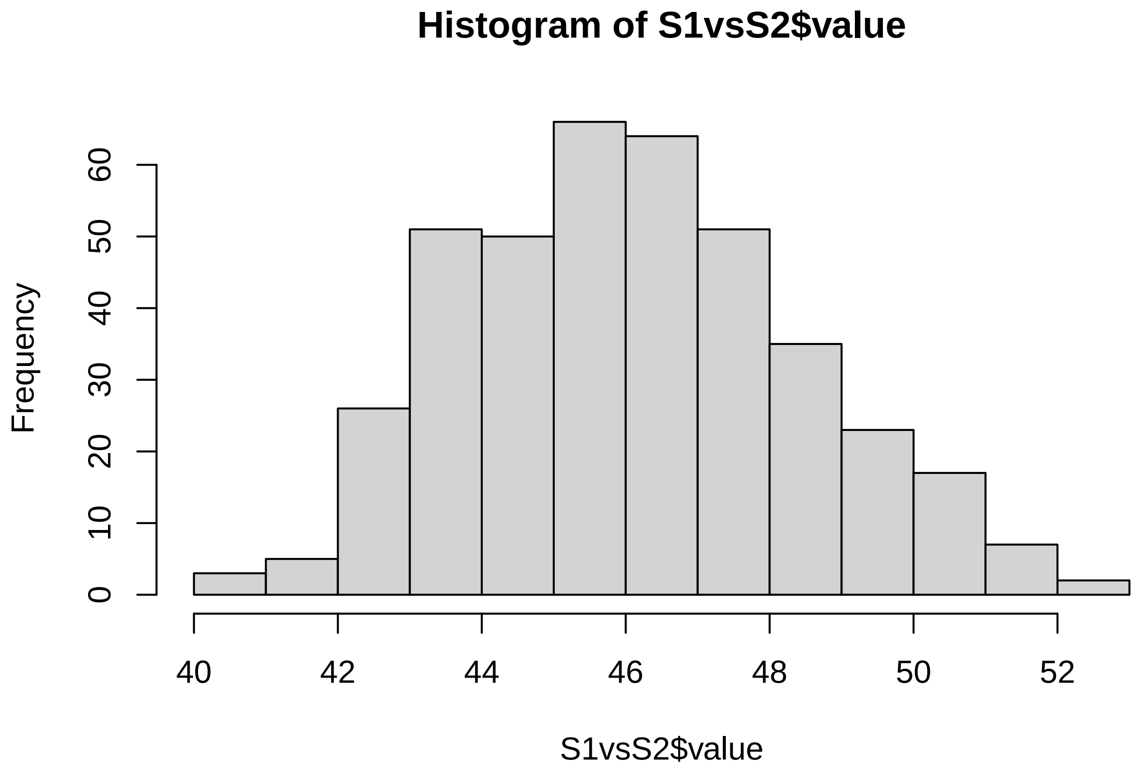


```
#Add jitter column
S1vsS2<-mutate(S1vsS2, jitter="S1vsS2")
#Add Comparison column
S1vsS2<-mutate(S1vsS2, Comparison="S1vsS2")


#Distance within P1
#Select comparisons where P1 is part of the first variable
P1vsP1<-filter(meltdist, grepl('P1', Var1))
#From this include comparisons to P1
P1vsP1<-filter(P1vsP1, grepl('P1', Var2))
#Histogram to look at distribution
hist(P1vsP1$value)
```

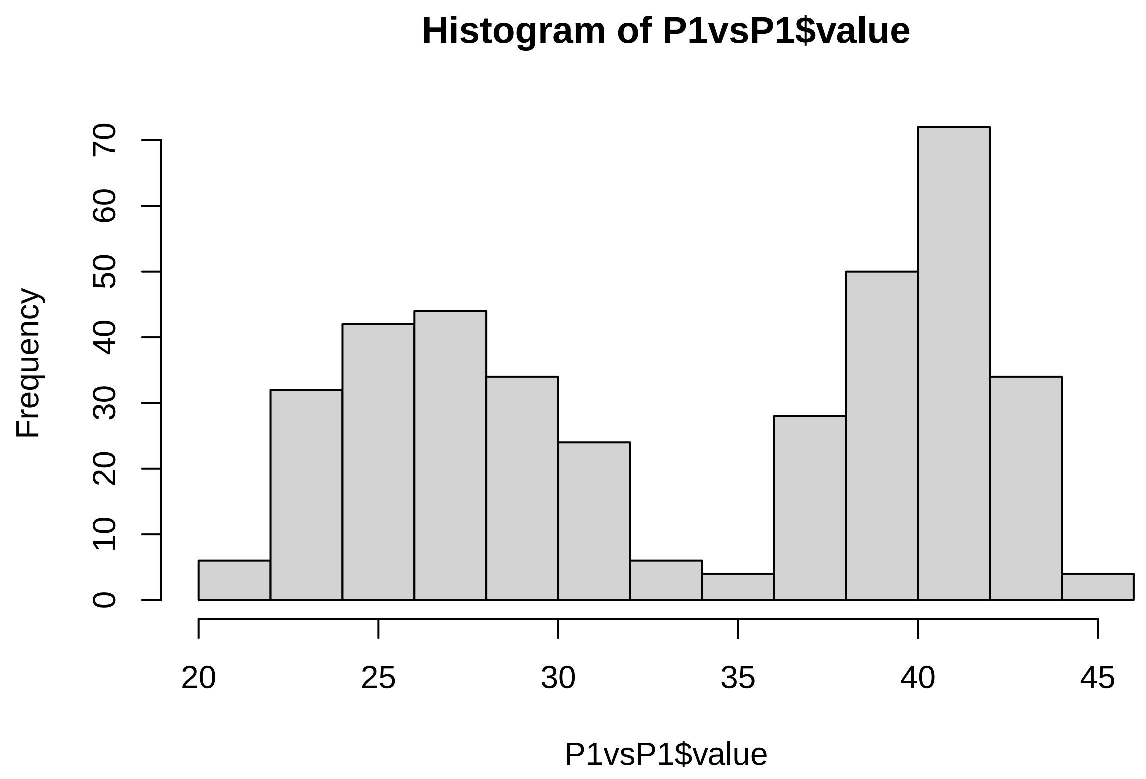


```
#Add jitter naming column
P1vsP1<-mutate(P1vsP1, jitter="P1")
#Add Comparison column
P1vsP1<-mutate(P1vsP1, Comparison="P1vsP1")


#Distance within P2
#Select comparisons where P2 is part of the first variable
P2vsP2<-filter(meltdist, grepl('P2', Var1))
#From this include comparisons to P2
P2vsP2<-filter(P2vsP2, grepl('P2', Var2))
#Histogram to look at distribution
hist(P2vsP2$value)
```

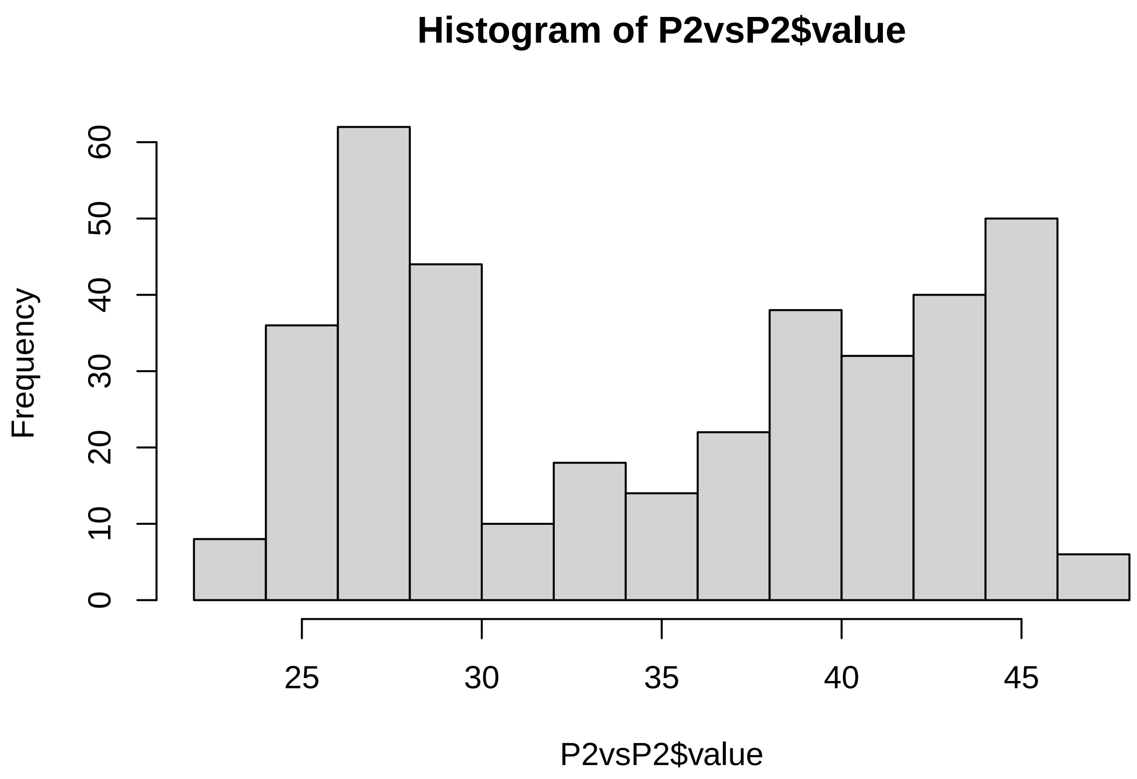


```
#Add jitter naming column
P2vsP2<-mutate(P2vsP2, jitter="P2")
#Add Comparison column
P2vsP2<-mutate(P2vsP2, Comparison="P2vsP2")


#Distance within S1
#Select comparisons where S1 is part of the first variable
S1vsS1<-filter(meltdist, grepl('S1', Var1))
#From this include comparisons to S1
S1vsS1<-filter(S1vsS1, grepl('S1', Var2))
#Histogram to look at distribution
hist(S1vsS1$value)
```

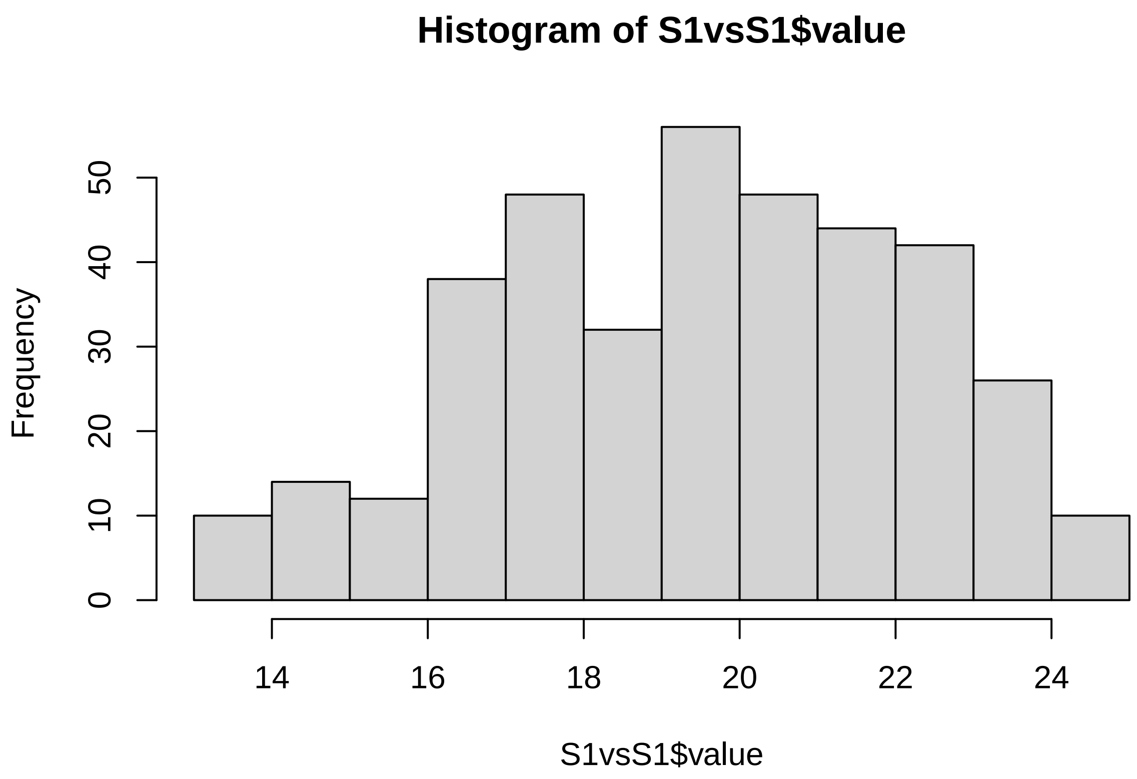


```r
#Add jitter naming column
S1vsS1<-mutate(S1vsS1, jitter="S1")
#Add Comparison column
S1vsS1<-mutate(S1vsS1, Comparison="S1vsS1")


#Distance within S2
#Select comparisons where S2 is part of the first variable
S2vsS2<-filter(meltdist, grepl('S2', Var1))
#From this include comparisons to S2
S2vsS2<-filter(S2vsS2, grepl('S2', Var2))
#Histogram to look at distribution
hist(S2vsS2$value)
```

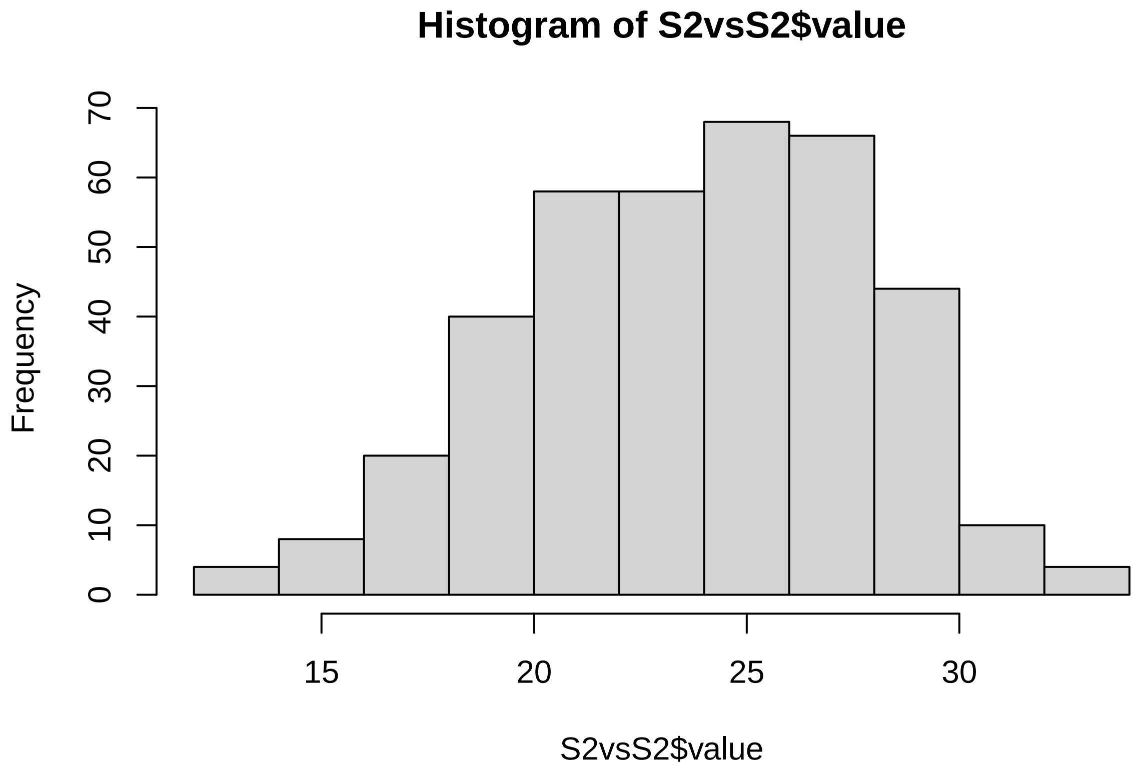


```r
#Add jitter naming column
S2vsS2<-mutate(S2vsS2, jitter="S2")
#Add Comparison column
S2vsS2<-mutate(S2vsS2, Comparison="S2vsS2")


#Distance between nextera runs
#Select comparisons where P1S1 and NX1 is part of the first variable
P1S1NX1vsP1S1NX2<-filter(meltdist, grepl('NX1NS_P1_0h', Var1))
#From this include comparisons to P1S1 and NX2
P1S1NX1vsP1S1NX2<-filter(P1S1NX1vsP1S1NX2, grepl('NX2NS_P1_0h', Var2))
#Select comparisons where P1S2 and NX1 is part of the first variable
P1S2NX1vsP1S2NX2<-filter(meltdist, grepl('NX1NS_P1_64h', Var1))
#From this include comparisons to P1S2 and NX2
P1S2NX1vsP1S2NX2<-filter(P1S2NX1vsP1S2NX2, grepl('NX2NS_P1_64h', Var2))
P1NX1vsP1NX2<-bind_rows(P1S1NX1vsP1S1NX2, P1S2NX1vsP1S2NX2)
#Histogram to look at distribution
hist(P1NX1vsP1NX2$value)
```

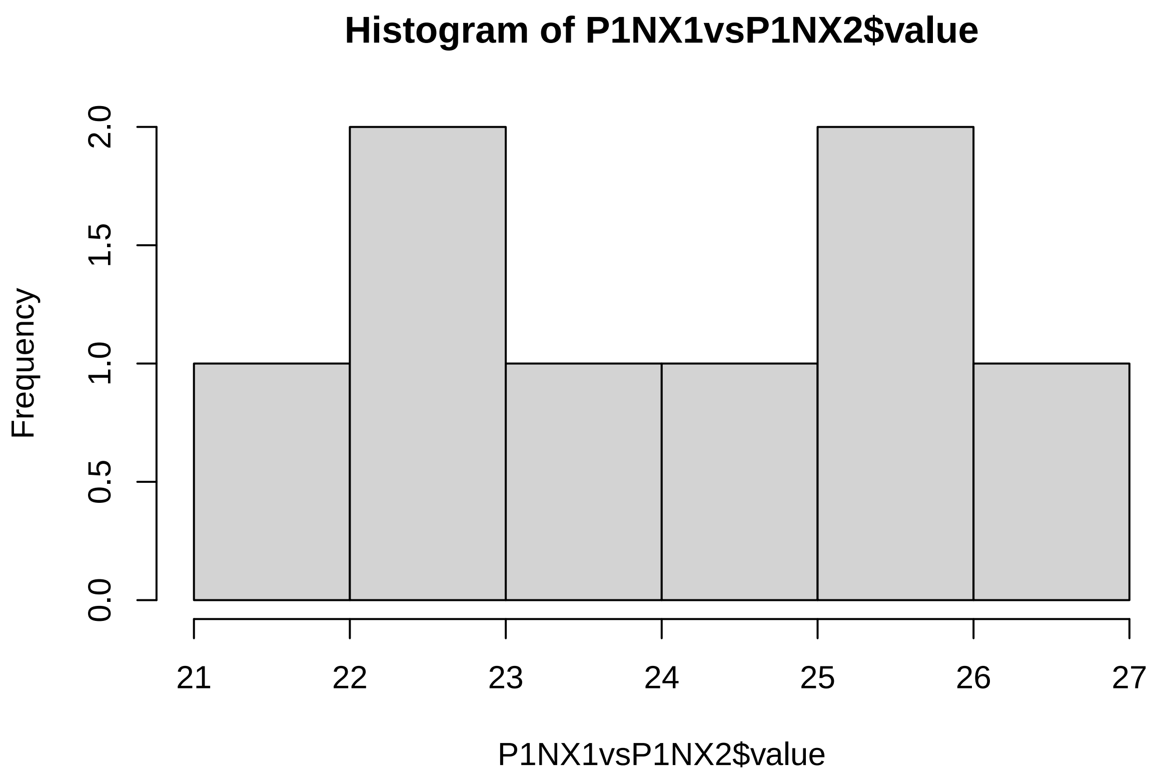


```r
#Add jitter naming column
P1NX1vsP1NX2<-mutate(P1NX1vsP1NX2, jitter="P1")
#Select comparisons where P2S1 and NX1 is part of the first variable
P2S1NX1vsP2S1NX2<-filter(meltdist, grepl('NX1NS_P2_0h', Var1))
#From this include comparisons to P2S1 and NX2
P2S1NX1vsP2S1NX2<-filter(P2S1NX1vsP2S1NX2, grepl('NX2NS_P2_0h', Var2))
#Select comparisons where P2S2 and NX1 is part of the first variable
P2S2NX1vsP2S2NX2<-filter(meltdist, grepl('NX1NS_P2_64h', Var1))
#From this include comparisons to P2S2 and NX2
P2S2NX1vsP2S2NX2<-filter(P2S2NX1vsP2S2NX2, grepl('NX2NS_P2_64h', Var2))
P2NX1vsP2NX2<-bind_rows(P2S1NX1vsP2S1NX2, P2S2NX1vsP2S2NX2)
#Histogram to look at distribution
hist(P2NX1vsP2NX2$value)
```

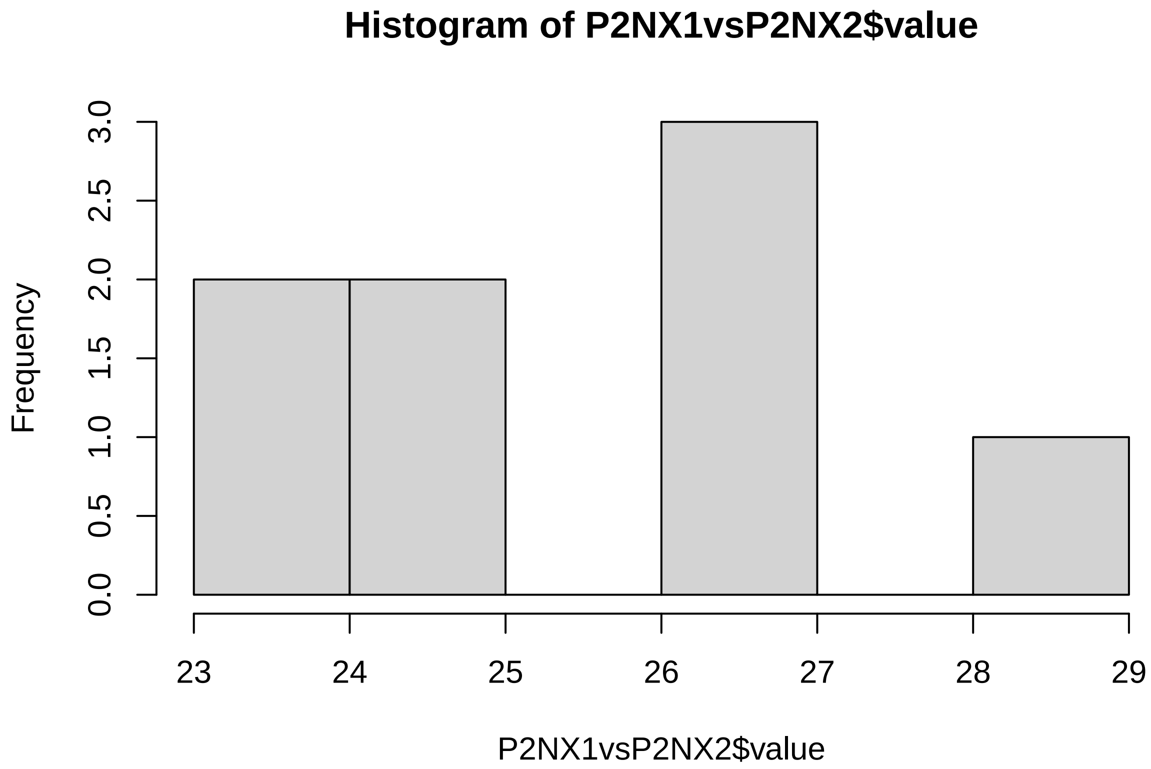


```r
#Add jitter naming column
P2NX1vsP2NX2<-mutate(P2NX1vsP2NX2, jitter="P2")
#Select comparisons where S1S1 and NX1 is part of the first variable
S1S1NX1vsS1S1NX2<-filter(meltdist, grepl('NX1NS_S1_0h', Var1))
#From this include comparisons to S1S1 and NX2
S1S1NX1vsS1S1NX2<-filter(S1S1NX1vsS1S1NX2, grepl('NX2NS_S1_0h', Var2))
#Select comparisons where S1S2 and NX1 is part of the first variable
S1S2NX1vsS1S2NX2<-filter(meltdist, grepl('NX1NS_S1_64h', Var1))
#From this include comparisons to S1S2 and NX2
S1S2NX1vsS1S2NX2<-filter(S1S2NX1vsS1S2NX2, grepl('NX2NS_S1_64h', Var2))
S1NX1vsS1NX2<-bind_rows(S1S1NX1vsS1S1NX2, S1S2NX1vsS1S2NX2)
#Histogram to look at distribution
hist(S1NX1vsS1NX2$value)
```

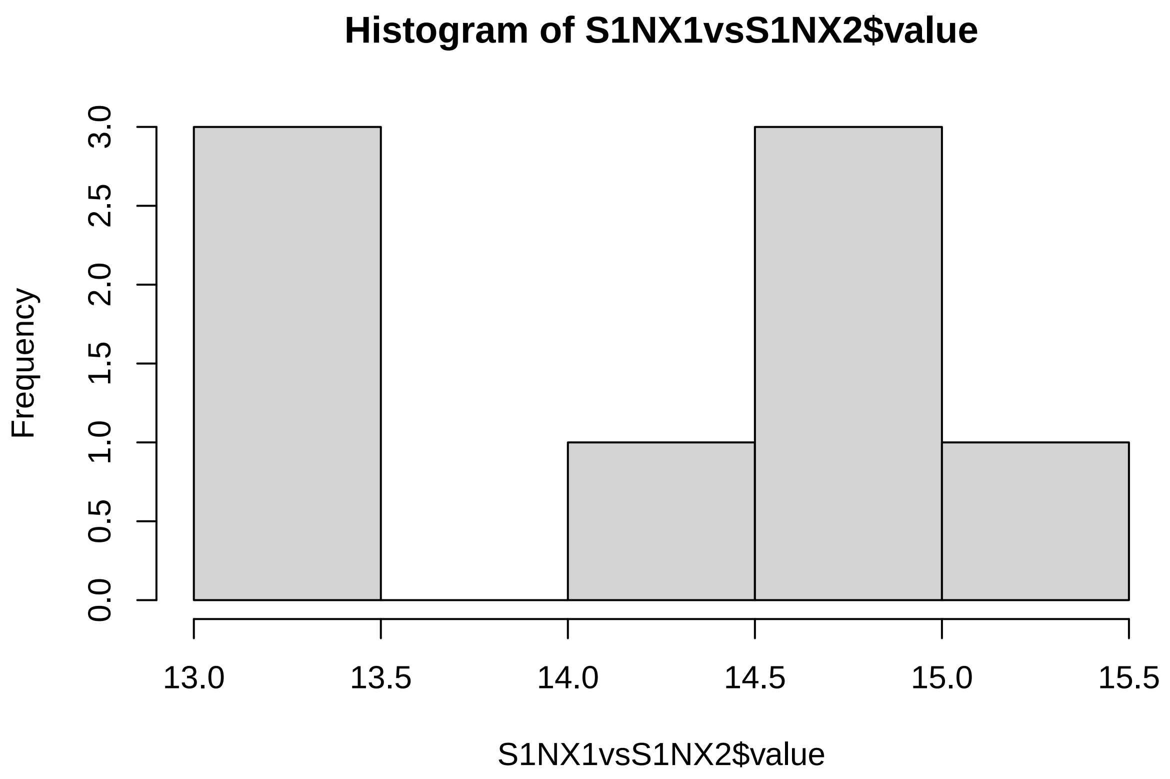


```r
#Add jitter naming column
S1NX1vsS1NX2<-mutate(S1NX1vsS1NX2, jitter="S1")
#Select comparisons where S2S1 and NX1 is part of the first variable
S2S1NX1vsS2S1NX2<-filter(meltdist, grepl('NX1NS_S2_0h', Var1))
#From this include comparisons to S2S1 and NX2
S2S1NX1vsS2S1NX2<-filter(S2S1NX1vsS2S1NX2, grepl('NX2NS_S2_0h', Var2))
#Select comparisons where S2S2 and NX1 is part of the first variable
S2S2NX1vsS2S2NX2<-filter(meltdist, grepl('NX1NS_S2_64h', Var1))
#From this include comparisons to S2S2 and NX2
S2S2NX1vsS2S2NX2<-filter(S2S2NX1vsS2S2NX2, grepl('NX2NS_S2_64h', Var2))
S2NX1vsS2NX2<-bind_rows(S2S1NX1vsS2S1NX2, S2S2NX1vsS2S2NX2)
#Histogram to look at distribution
hist(S2NX1vsS2NX2$value)
```

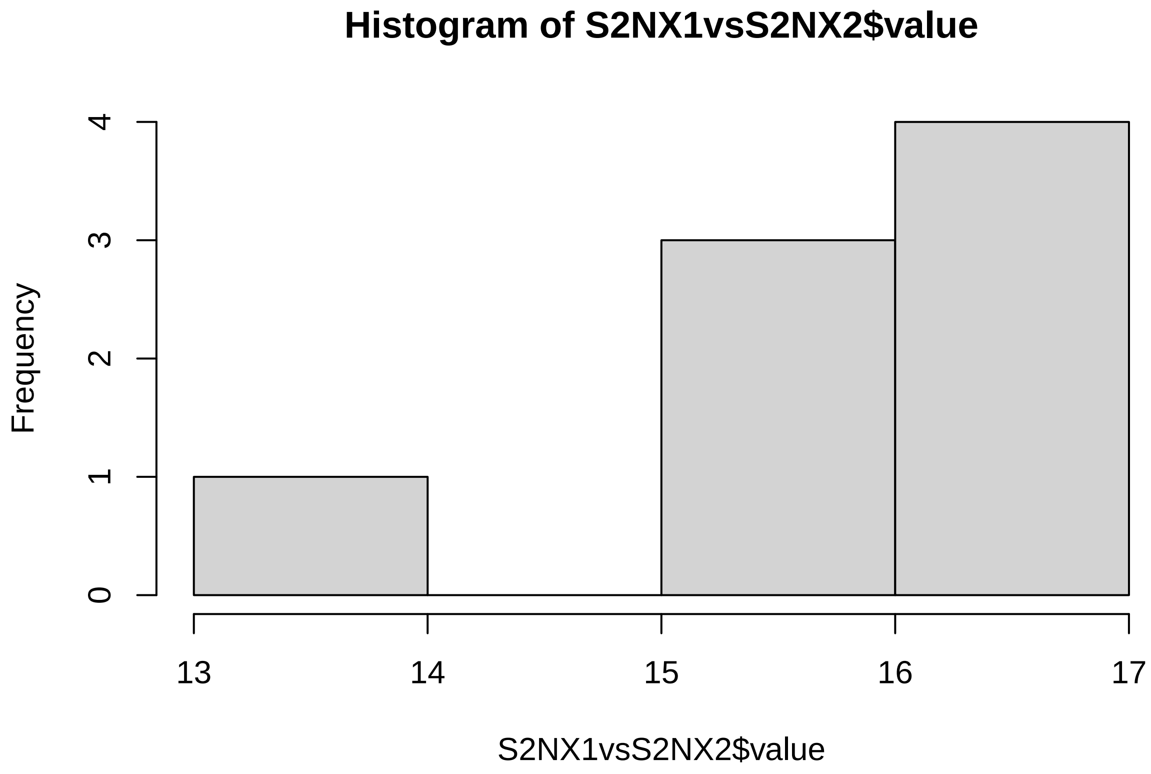


```
#Add jitter naming column
S2NX1vsS2NX2<-mutate(S2NX1vsS2NX2, jitter="S2")
#Combine comparisons of nextera runs in all samples
NX1vsNX2<-bind_rows(P1NX1vsP1NX2, P2NX1vsP2NX2, S1NX1vsS1NX2, S2NX1vsS2NX2)
#Histogram to look at distribution
hist(NX1vsNX2$value)
```

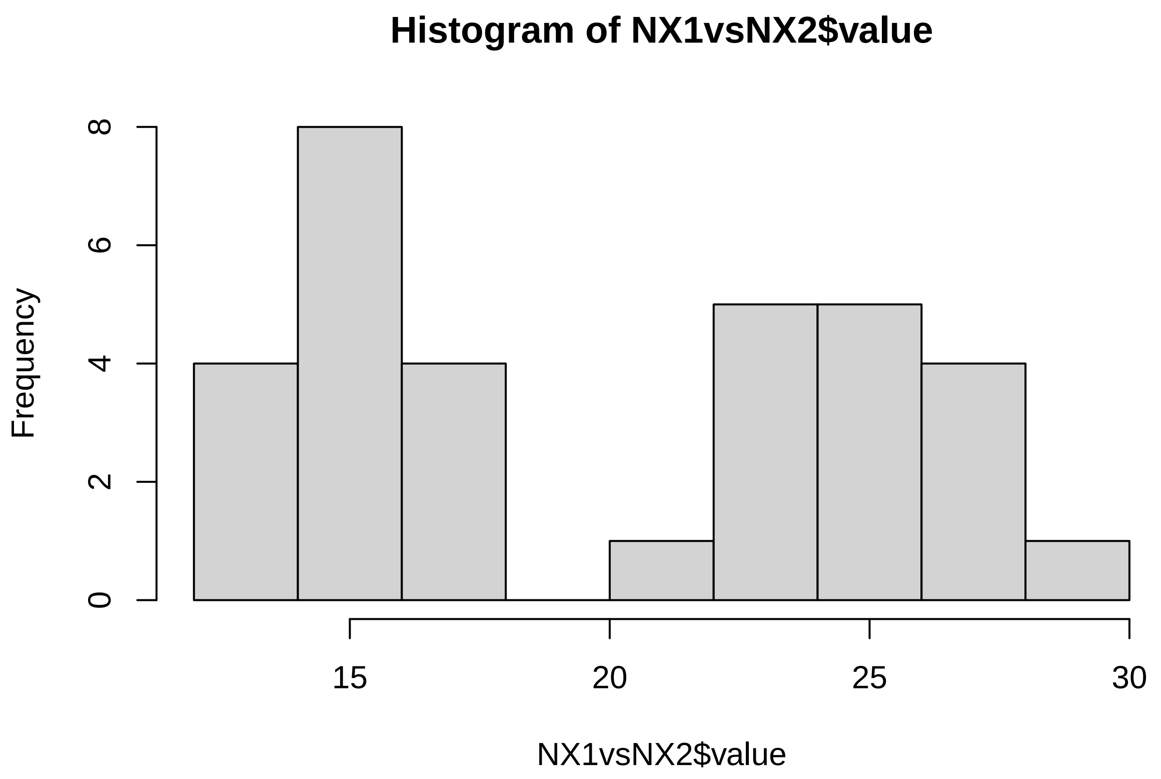


```
#Add Comparison column
NX1vsNX2<-mutate(NX1vsNX2, Comparison="NXNSRep")


#Distance between DNA extraction runs
#Makes column based on Var1 that only contain middle part of sample name
DNAExRep <- mutate(meltdist, from = str_replace(Var1,"[A-X0-9]*_[A-X0-9]*_[A-X0-9]*_","")
                   %>% str_replace("_[a-z]$",""))
#Makes column based on Var2 that only contain middle part of sample name
DNAExRep <- mutate(DNAExRep, to = str_replace(Var2,"[A-X0-9]*_[A-X0-9]*_[A-X0-9]*_","")
                   %>% str_replace("_[a-z]$",""))
#Makes a column were TRUE means from is the same as to otherwise FALSE
DNAExRep <- mutate(DNAExRep, rep = (from == to))
##Only contain samples that are replicates containing TRUE in rep
DNAExRep<-filter(DNAExRep, grepl('TRUE', rep))
#Add jitter column
DNAExRep$jitter <-
        ifelse(seq(along=(DNAExRep$Var1)) %in% grep("*_P1_*", DNAExRep$Var1), "P1",
        ifelse(seq(along=(DNAExRep$Var1)) %in% grep("*_P2_*", DNAExRep$Var1), "P2",
        ifelse(seq(along=(DNAExRep$Var1)) %in% grep("*_S1_*", DNAExRep$Var1), "S1",
        ifelse(seq(along=(DNAExRep$Var1)) %in% grep("*_S2_*", DNAExRep$Var1), "S2",
               "Other"))))
#Add Comparison column
DNAExRep<-mutate(DNAExRep, Comparison="DNAExRep")
#Remove from, to and rep column
DNAExRep<-dplyr::select(DNAExRep, one_of(c("Var1", "Var2", "value", "jitter",
```

```
                                                    "Comparison")))


#Distance between Nextflex run on Hiseq and Nextseq
#Select comparisons where P1, 0h storage and NFHI is part of the first variable
  #(Part of the HX experiment)
P10hNFHSvsNS<-filter(meltdist, grepl('HX_P1_0h', Var1))
#From this include comparisons to P1, 0h storage and NFNS
P10hNFHSvsNS<-filter(P10hNFHSvsNS, grepl('NFNS_P1_0h', Var2))
#Select comparisons where P1, 64h storage and NFHI is part of the first variable
  #(Part of the HX experiment)
P164hNFHSvsNS<-filter(meltdist, grepl('HX_P1_64h', Var1))
#From this include comparisons to P1, 64h storage and NFNS
P164hNFHSvsNS<-filter(P164hNFHSvsNS, grepl('NFNS_P1_64h', Var2))
P1NFHSvsNS<-bind_rows(P10hNFHSvsNS, P164hNFHSvsNS)
#Histogram to look at distribution
hist(P1NFHSvsNS$value)
```

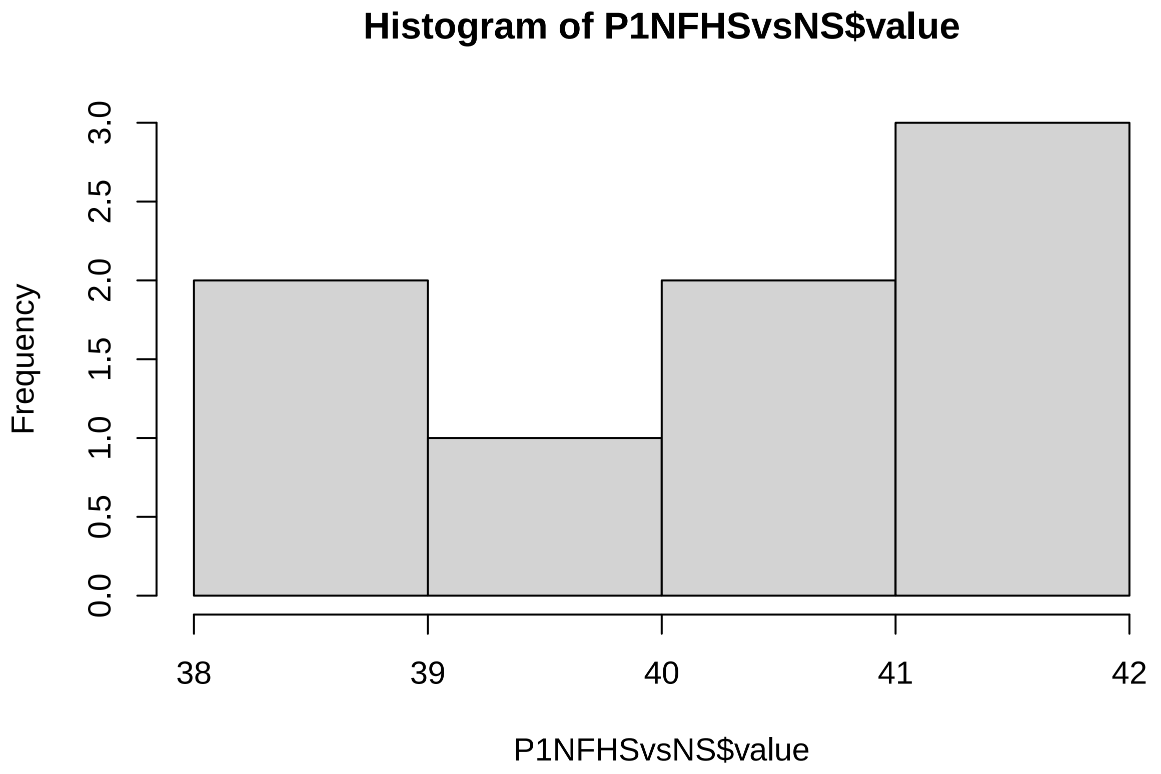


```
#Add jitter naming column
P1NFHSvsNS<-mutate(P1NFHSvsNS, jitter="P1")
#Select comparisons where P2, 0h storage and NFHI is part of the first variable
  #(Part of the HX experiment)
P20hNFHSvsNS<-filter(meltdist, grepl('HX_P2_0h', Var1))
#From this include comparisons to P2, 0h storage and NFNS
P20hNFHSvsNS<-filter(P20hNFHSvsNS, grepl('NFNS_P2_0h', Var2))
#Select comparisons where P2, 64h storage and NFHI is part of the first variable
```

```
  #(Part of the HX experiment)
P264hNFHSvsNS<-filter(meltdist, grepl('HX_P2_64h', Var1))
#From this include comparisons to P2, 64h storage and NFNS
P264hNFHSvsNS<-filter(P264hNFHSvsNS, grepl('NFNS_P2_64h', Var2))
P2NFHSvsNS<-bind_rows(P20hNFHSvsNS, P264hNFHSvsNS)
#Histogram to look at distribution
hist(P2NFHSvsNS$value)
```

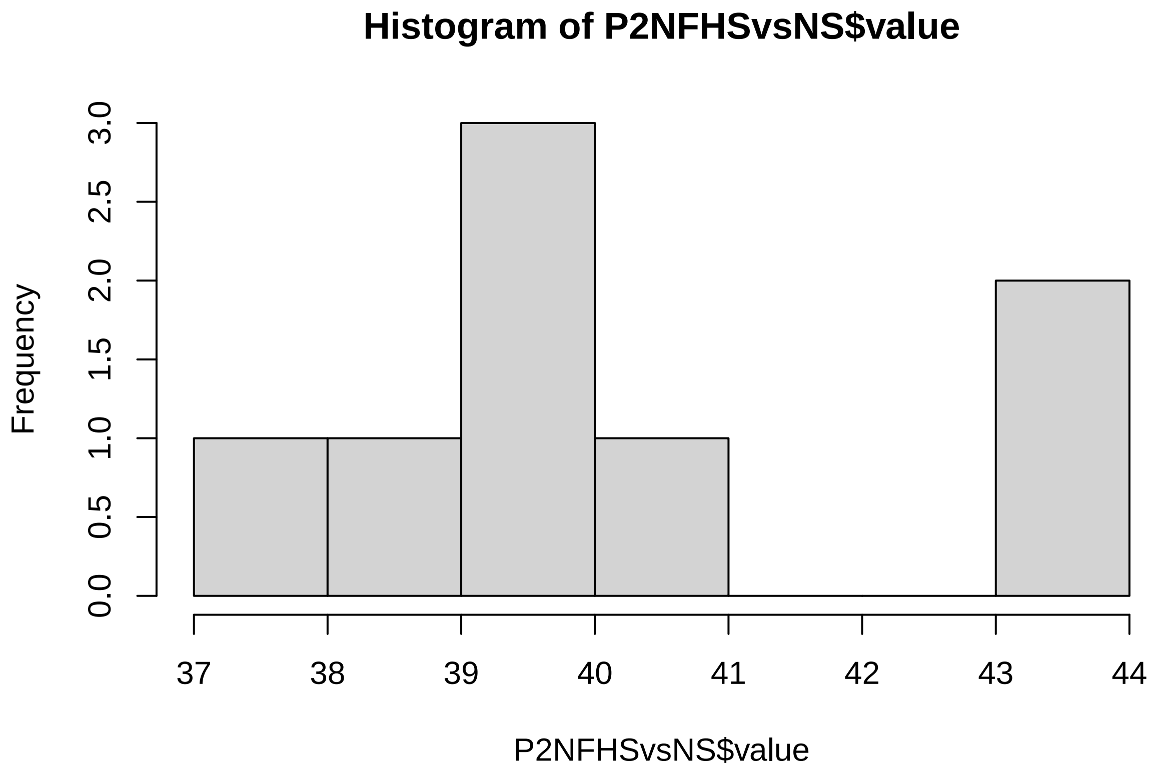


```
#Add jitter naming column
P2NFHSvsNS<-mutate(P2NFHSvsNS, jitter="P2")
#Select comparisons where S1, 0h storage and NFHI is part of the first variable
  #(Part of the HX experiment)
S10hNFHSvsNS<-filter(meltdist, grepl('HX_S1_0h', Var1))
#From this include comparisons to S1, 0h storage and NFNS
S10hNFHSvsNS<-filter(S10hNFHSvsNS, grepl('NFNS_S1_0h', Var2))
#Select comparisons where S1, 64h storage and NFHI is part of the first variable
  #(Part of the HX experiment)
S164hNFHSvsNS<-filter(meltdist, grepl('HX_S1_64h', Var1))
#From this include comparisons to S1, 64h storage and NFNS
S164hNFHSvsNS<-filter(S164hNFHSvsNS, grepl('NFNS_S1_64h', Var2))
S1NFHSvsNS<-bind_rows(S10hNFHSvsNS, S164hNFHSvsNS)
#Histogram to look at distribution
hist(S1NFHSvsNS$value)
```

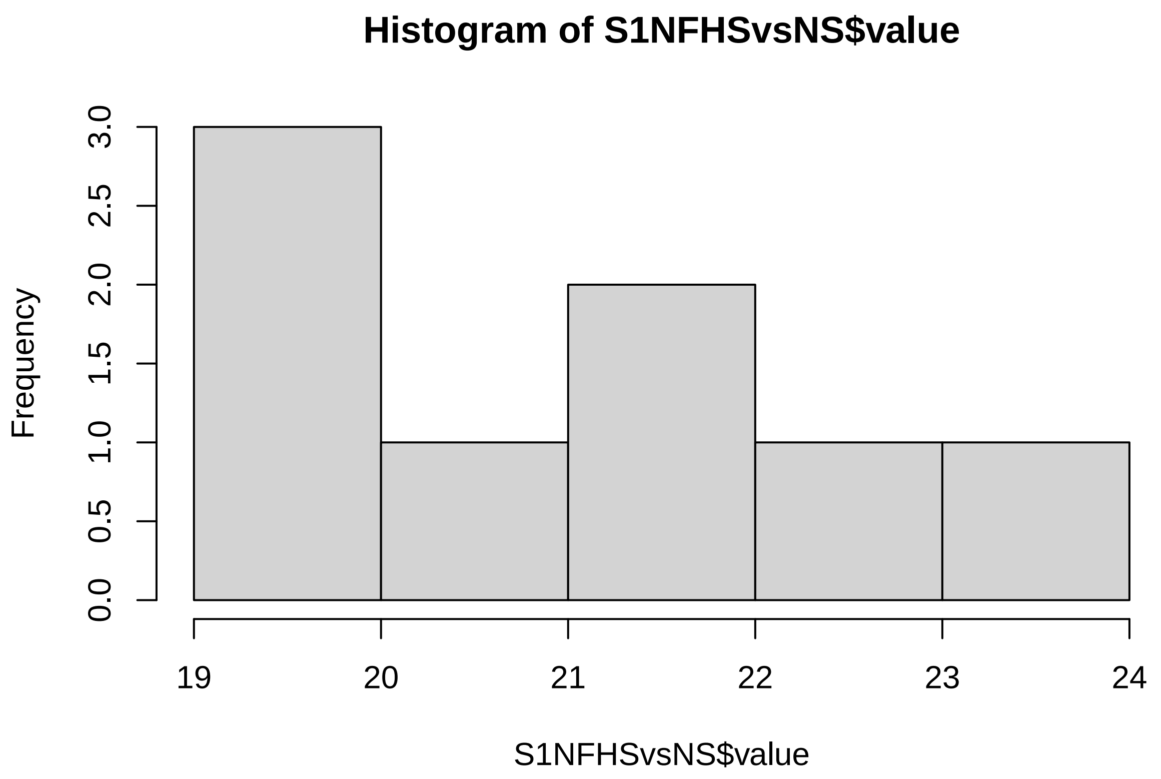


```r
#Add jitter naming column
S1NFHSvsNS<-mutate(S1NFHSvsNS, jitter="S1")
#Select comparisons where S2, 0h storage and NFHI is part of the first variable
  #(Part of the HX experiment)
S20hNFHSvsNS<-filter(meltdist, grepl('HX_S2_0h', Var1))
#From this include comparisons to S2, 0h storage and NFNS
S20hNFHSvsNS<-filter(S20hNFHSvsNS, grepl('NFNS_S2_0h', Var2))
#Select comparisons where S2, 64h storage and NFHI is part of the first variable
  #(Part of the HX experiment)
S264hNFHSvsNS<-filter(meltdist, grepl('HX_S2_64h', Var1))
#From this include comparisons to S2, 64h storage and NFNS
S264hNFHSvsNS<-filter(S264hNFHSvsNS, grepl('KAHI_S2_64h', Var2))
S2NFHSvsNS<-bind_rows(S20hNFHSvsNS, S264hNFHSvsNS)
#Histogram to look at distribution
hist(S2NFHSvsNS$value)
```

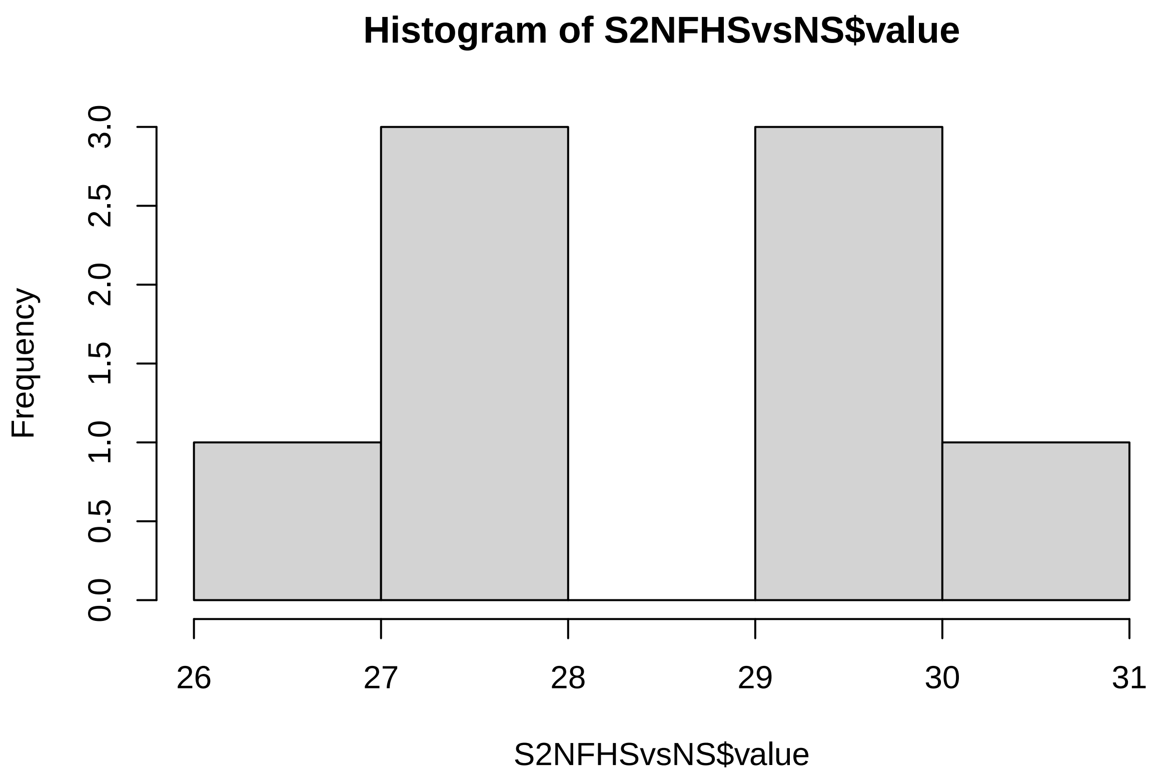


```
#Add jitter naming column
S2NFHSvsNS<-mutate(S2NFHSvsNS, jitter="S2")
#Combine comparisons of nextera runs in all samples
NFHSvsNS<-bind_rows(P1NFHSvsNS, P2NFHSvsNS, S1NFHSvsNS, S2NFHSvsNS)
#Histogram to look at distribution
hist(NFHSvsNS$value)
```

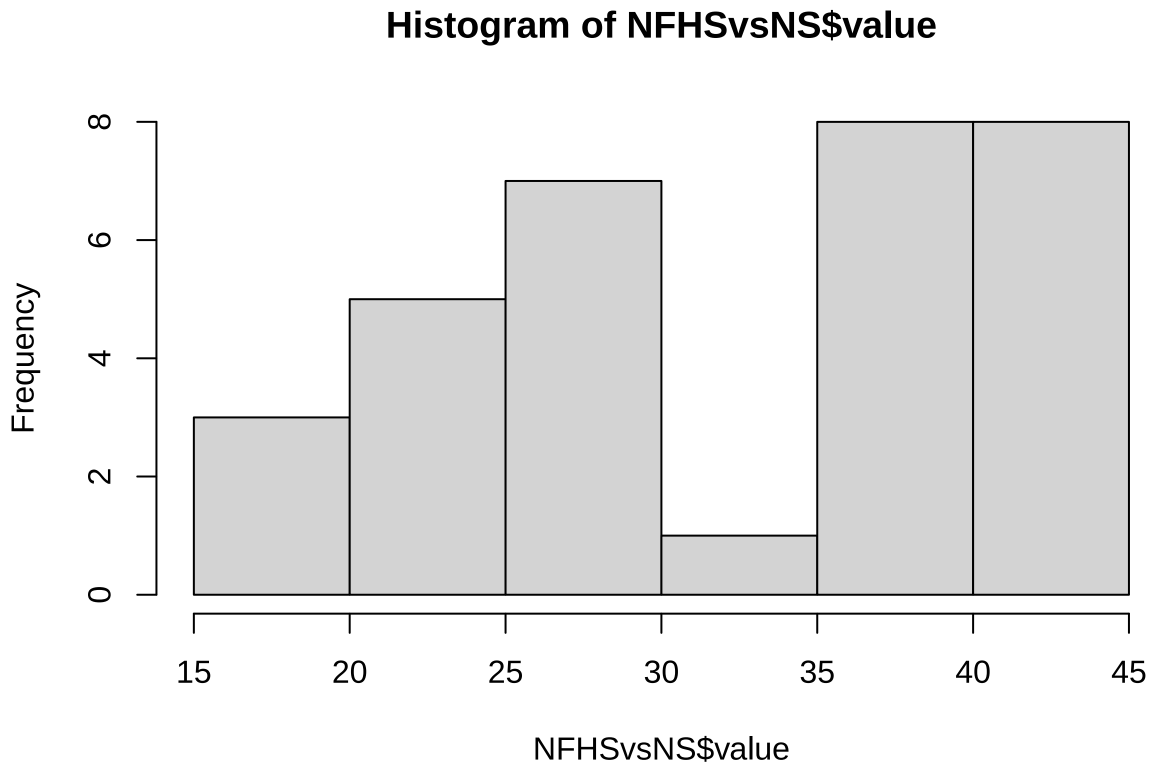


```
#Add Comparison column
NFHSvsNS<-mutate(NFHSvsNS, Comparison="NFHSvsNS")


#Distance between Nextflex and Kappa run on Hiseq
#Select comparisons where P1, 0h storage and NFHI is part of the first variable
  #(Part of the HX experiment)
P10hNFvsKAHI<-filter(meltdist, grepl('HX_P1_0h', Var1))
#From this include comparisons to P1, 0h storage and NFNS
P10hNFvsKAHI<-filter(P10hNFvsKAHI, grepl('KAHI_P1_0h', Var2))
#Select comparisons where P1, 64h storage and NFHI is part of the first variable
  #(Part of the HX experiment)
P164hNFvsKAHI<-filter(meltdist, grepl('HX_P1_64h', Var1))
#From this include comparisons to P1, 64h storage and NFNS
P164hNFvsKAHI<-filter(P164hNFvsKAHI, grepl('KAHI_P1_64h', Var2))
P1NFvsKAHI<-bind_rows(P10hNFvsKAHI, P164hNFvsKAHI)
#Histogram to look at distribution
hist(P1NFvsKAHI$value)
```

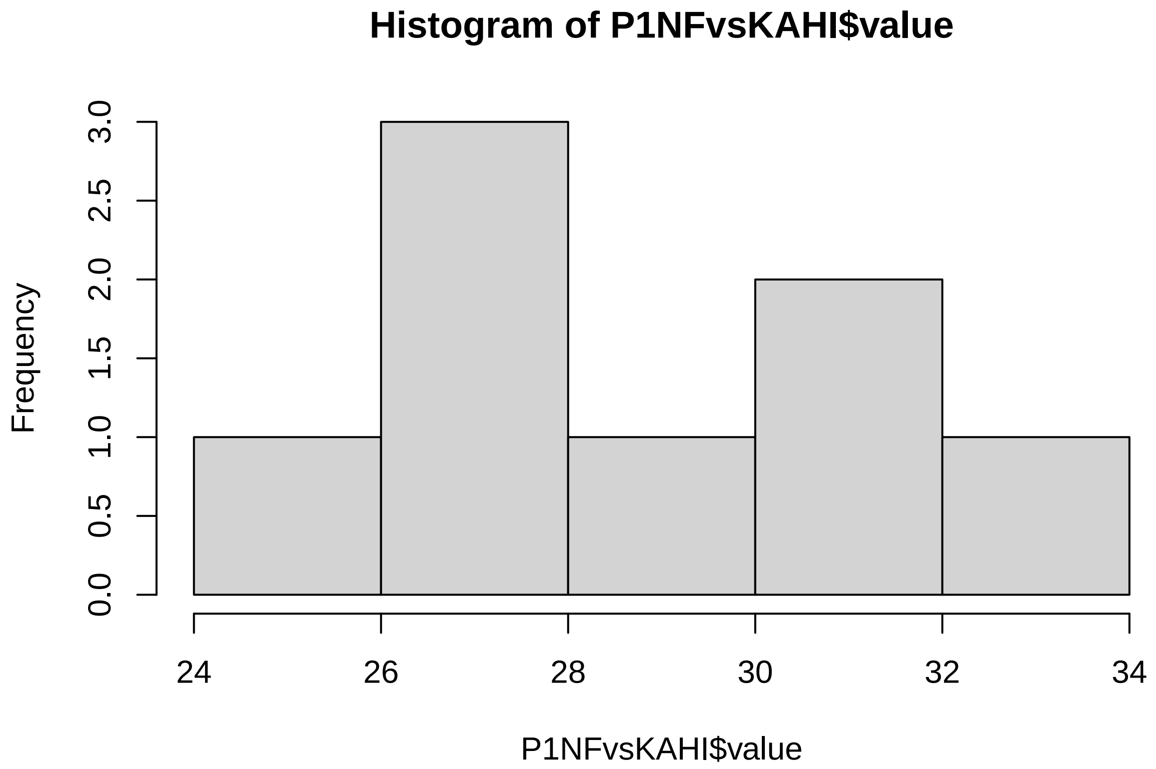


```r
#Add jitter naming column
P1NFvsKAHI<-mutate(P1NFvsKAHI, jitter="P1")
#Select comparisons where P2, 0h storage and NFHI is part of the first variable
  #(Part of the HX experiment)
P20hNFvsKAHI<-filter(meltdist, grepl('HX_P2_0h', Var1))
#From this include comparisons to P2, 0h storage and NFNS
P20hNFvsKAHI<-filter(P20hNFvsKAHI, grepl('KAHI_P2_0h', Var2))
#Select comparisons where P2, 64h storage and NFHI is part of the first variable
  #(Part of the HX experiment)
P264hNFvsKAHI<-filter(meltdist, grepl('HX_P2_64h', Var1))
#From this include comparisons to P2, 64h storage and NFNS
P264hNFvsKAHI<-filter(P264hNFvsKAHI, grepl('KAHI_P2_64h', Var2))
P2NFvsKAHI<-bind_rows(P20hNFvsKAHI, P264hNFvsKAHI)
#Histogram to look at distribution
hist(P2NFvsKAHI$value)
```

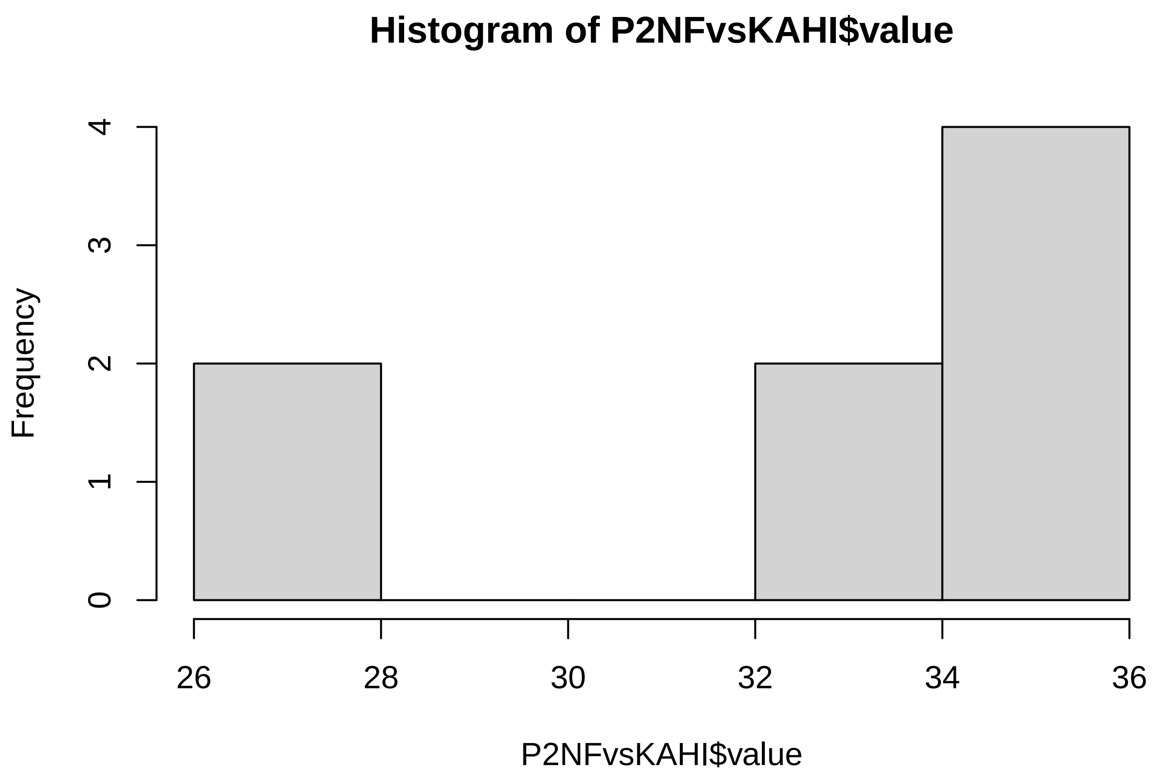


```
#Add jitter naming column
P2NFvsKAHI<-mutate(P2NFvsKAHI, jitter="P2")
#Select comparisons where S1, 0h storage and NFHI is part of the first variable
  #(Part of the HX experiment)
S10hNFvsKAHI<-filter(meltdist, grepl('HX_S1_0h', Var1))
#From this include comparisons to S1, 0h storage and NFNS
S10hNFvsKAHI<-filter(S10hNFvsKAHI, grepl('KAHI_S1_0h', Var2))
#Select comparisons where S1, 64h storage and NFHI is part of the first variable
  #(Part of the HX experiment)
S164hNFvsKAHI<-filter(meltdist, grepl('HX_S1_64h', Var1))
#From this include comparisons to S1, 64h storage and NFNS
S164hNFvsKAHI<-filter(S164hNFvsKAHI, grepl('KAHI_S1_64h', Var2))
S1NFvsKAHI<-bind_rows(S10hNFvsKAHI, S164hNFvsKAHI)
#Histogram to look at distribution
hist(S1NFvsKAHI$value)
```

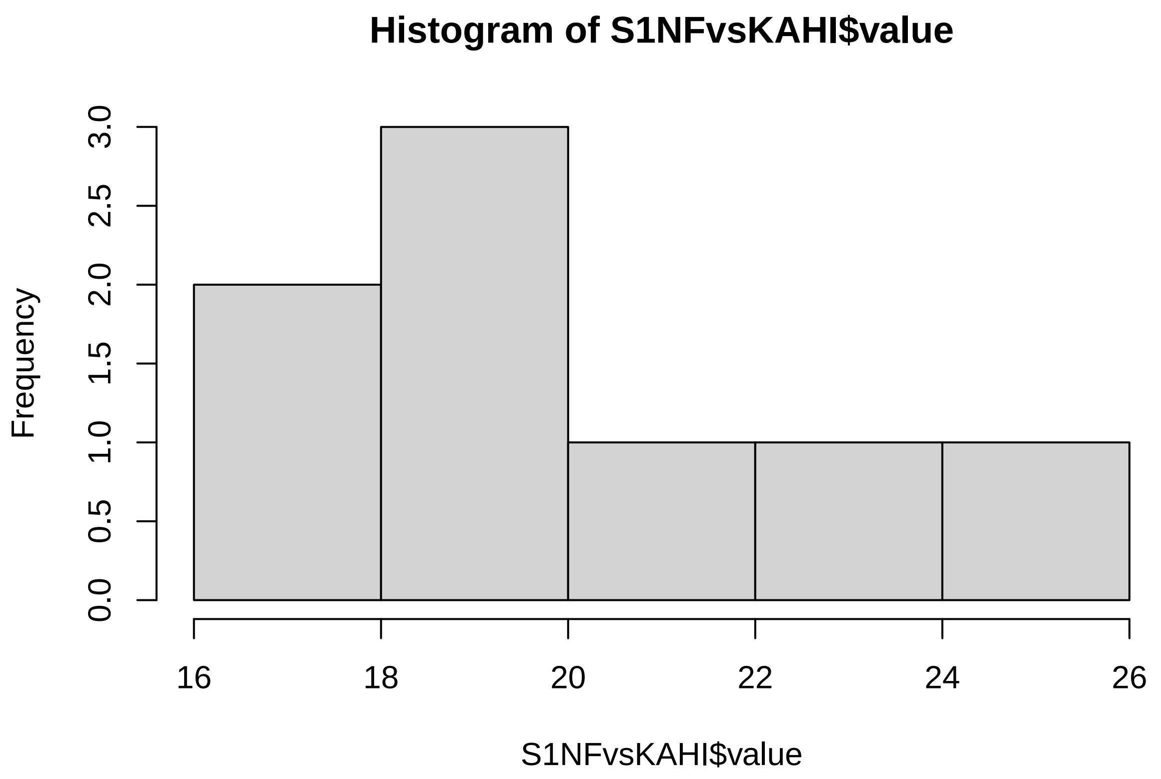


```r
#Add jitter naming column
S1NFvsKAHI<-mutate(S1NFvsKAHI, jitter="S1")
#Select comparisons where S2, 0h storage and NFHI is part of the first variable
  #(Part of the HX experiment)
S20hNFvsKAHI<-filter(meltdist, grepl('HX_S2_0h', Var1))
#From this include comparisons to S2, 0h storage and NFNS
S20hNFvsKAHI<-filter(S20hNFvsKAHI, grepl('KAHI_S2_0h', Var2))
#Select comparisons where S2, 64h storage and NFHI is part of the first variable
  #(Part of the HX experiment)
S264hNFvsKAHI<-filter(meltdist, grepl('HX_S2_64h', Var1))
#From this include comparisons to S2, 64h storage and NFNS
S264hNFvsKAHI<-filter(S264hNFvsKAHI, grepl('KAHI_S2_64h', Var2))
S2NFvsKAHI<-bind_rows(S20hNFvsKAHI, S264hNFvsKAHI)
#Histogram to look at distribution
hist(S2NFvsKAHI$value)
```

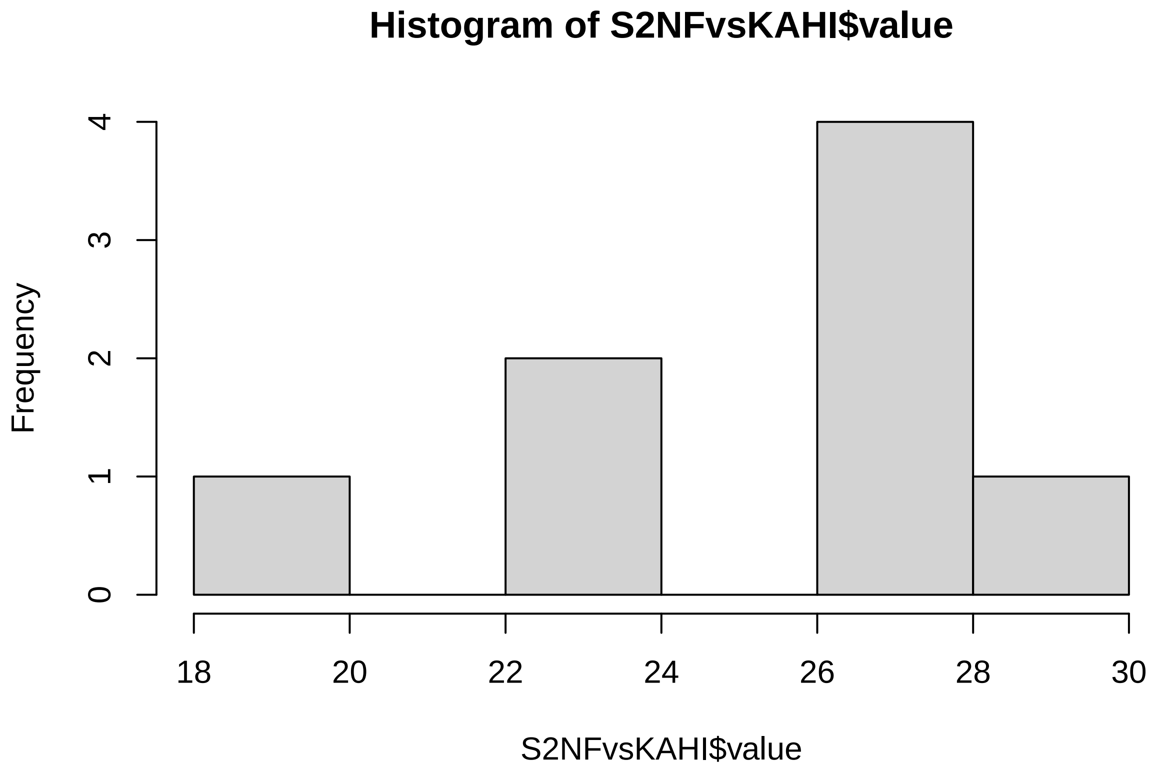


```
#Add jitter naming column
S2NFvsKAHI<-mutate(S2NFvsKAHI, jitter="S2")
#Combine comparisons of nextflex kappa runs in all samples on Hiseq
NFvsKAHI<-bind_rows(P1NFvsKAHI, P2NFvsKAHI, S1NFvsKAHI, S2NFvsKAHI)
#Histogram to look at distribution
hist(NFvsKAHI$value)
```

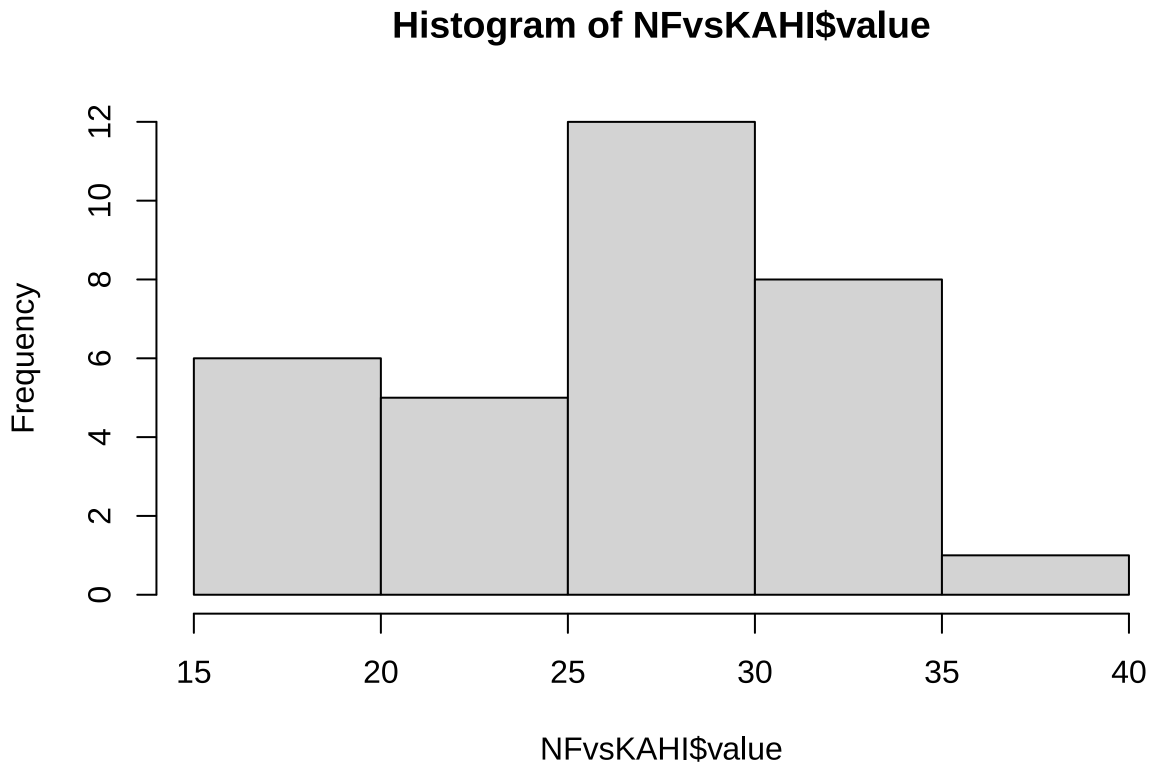


```
#Add Comparison column
NFvsKAHI<-mutate(NFvsKAHI, Comparison="NFvsKAHI")


#Distance between Nextflex and Nextera runs on Nextseq
#Select comparisons where P1, 0h storage and NFHI is part of the first variable
  #(Part of the HX experiment)
P10hNFvsNXNS<-filter(meltdist, grepl('NFNS_P1_0h', Var1))
#From this include comparisons to P1, 0h storage and NFNS
P10hNFvsNXNS<-filter(P10hNFvsNXNS, grepl('NX[0-9]NS_P1_0h', Var2))
#Select comparisons where P1, 64h storage and NFHI is part of the first variable
  #(Part of the HX experiment)
P164hNFvsNXNS<-filter(meltdist, grepl('NFNS_P1_64h', Var1))
#From this include comparisons to P1, 64h storage and NFNS
P164hNFvsNXNS<-filter(P164hNFvsNXNS, grepl('NX[0-9]NS_P1_64h', Var2))
P1NFvsNXNS<-bind_rows(P10hNFvsNXNS, P164hNFvsNXNS)
#Histogram to look at distribution
hist(P1NFvsNXNS$value)
```

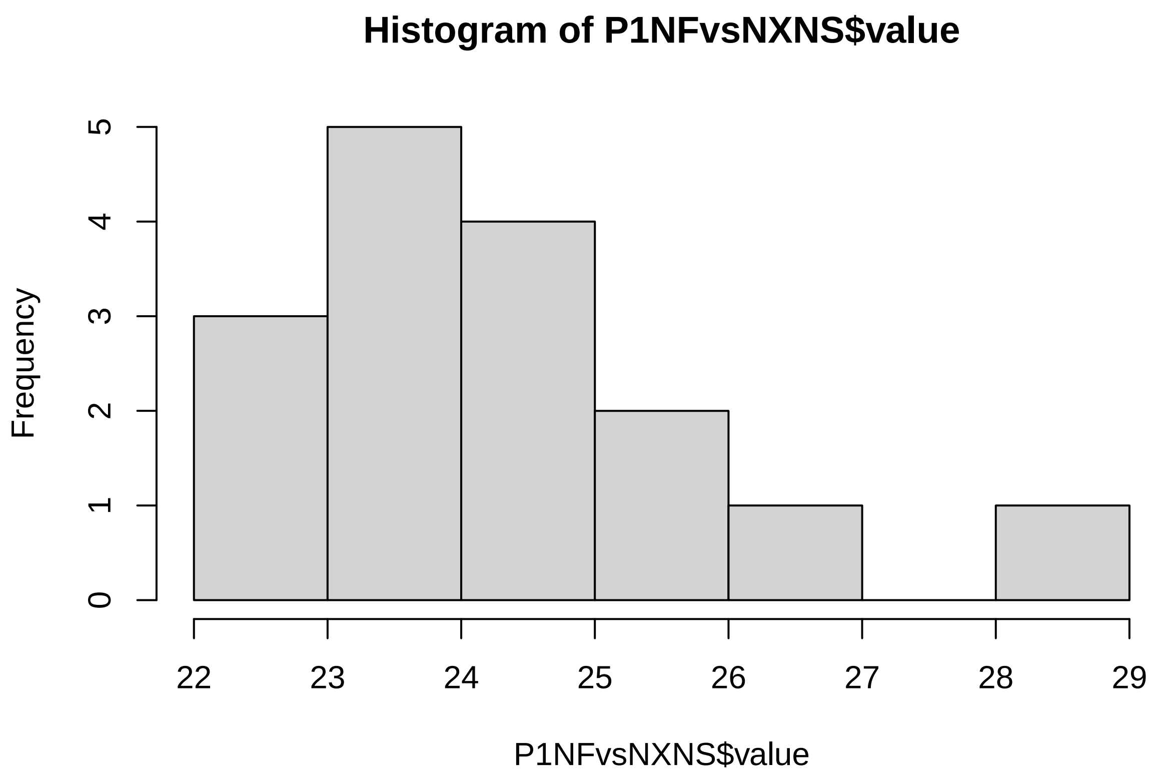


```
#Add jitter naming column
P1NFvsNXNS<-mutate(P1NFvsNXNS, jitter="P1")
#Select comparisons where P2, 0h storage and NFHI is part of the first variable
  #(Part of the HX experiment)
P20hNFvsNXNS<-filter(meltdist, grepl('NFNS_P2_0h', Var1))
#From this include comparisons to P2, 0h storage and NFNS
P20hNFvsNXNS<-filter(P20hNFvsNXNS, grepl('NX[0-9]NS_P2_0h', Var2))
#Select comparisons where P2, 64h storage and NFHI is part of the first variable
  #(Part of the HX experiment)
P264hNFvsNXNS<-filter(meltdist, grepl('NFNS_P2_64h', Var1))
#From this include comparisons to P2, 64h storage and NFNS
P264hNFvsNXNS<-filter(P264hNFvsNXNS, grepl('NX[0-9]NS_P2_64h', Var2))
P2NFvsNXNS<-bind_rows(P20hNFvsNXNS, P264hNFvsNXNS)
#Histogram to look at distribution
hist(P2NFvsNXNS$value)
```

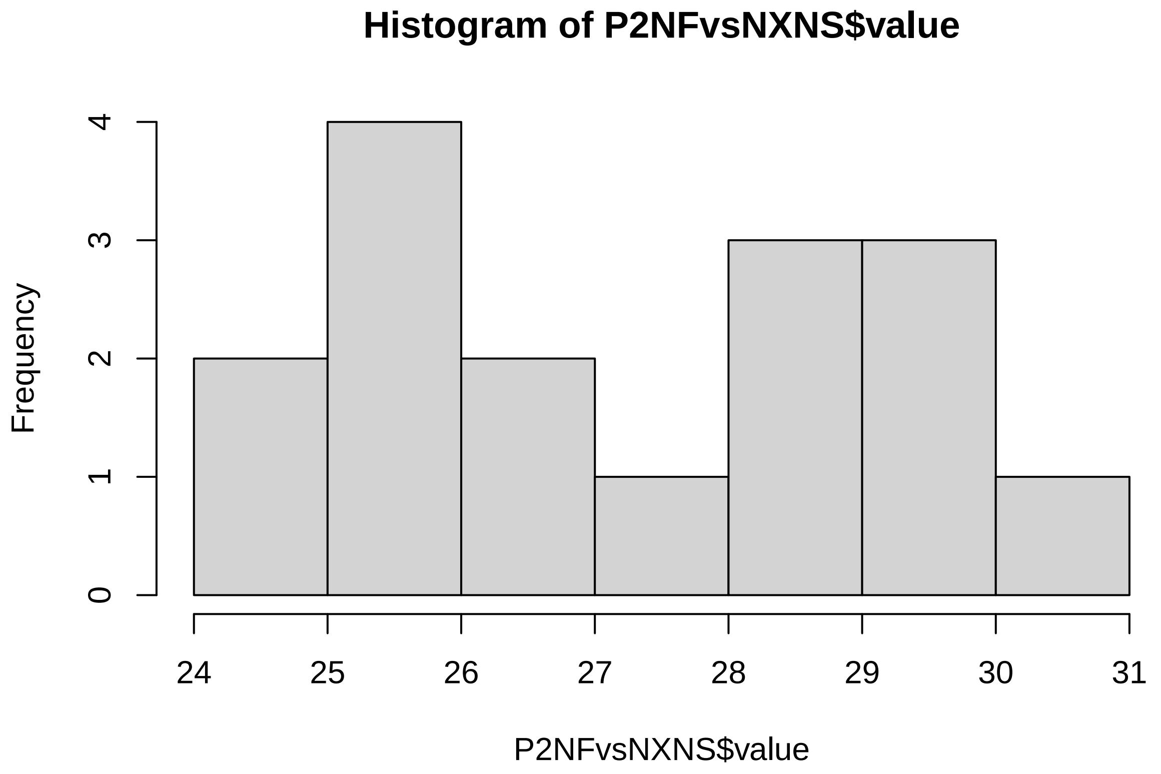


```r
#Add jitter naming column
P2NFvsNXNS<-mutate(P2NFvsNXNS, jitter="P2")
#Select comparisons where S1, 0h storage and NFHI is part of the first variable
  #(Part of the HX experiment)
S10hNFvsNXNS<-filter(meltdist, grepl('NFNS_S1_0h', Var1))
#From this include comparisons to S1, 0h storage and NFNS
S10hNFvsNXNS<-filter(S10hNFvsNXNS, grepl('NX[0-9]NS_S1_0h', Var2))
#Select comparisons where S1, 64h storage and NFHI is part of the first variable
  #(Part of the HX experiment)
S164hNFvsNXNS<-filter(meltdist, grepl('NFNS_S1_64h', Var1))
#From this include comparisons to S1, 64h storage and NFNS
S164hNFvsNXNS<-filter(S164hNFvsNXNS, grepl('NX[0-9]NS_S1_64h', Var2))
S1NFvsNXNS<-bind_rows(S10hNFvsNXNS, S164hNFvsNXNS)
#Histogram to look at distribution
hist(S1NFvsNXNS$value)
```

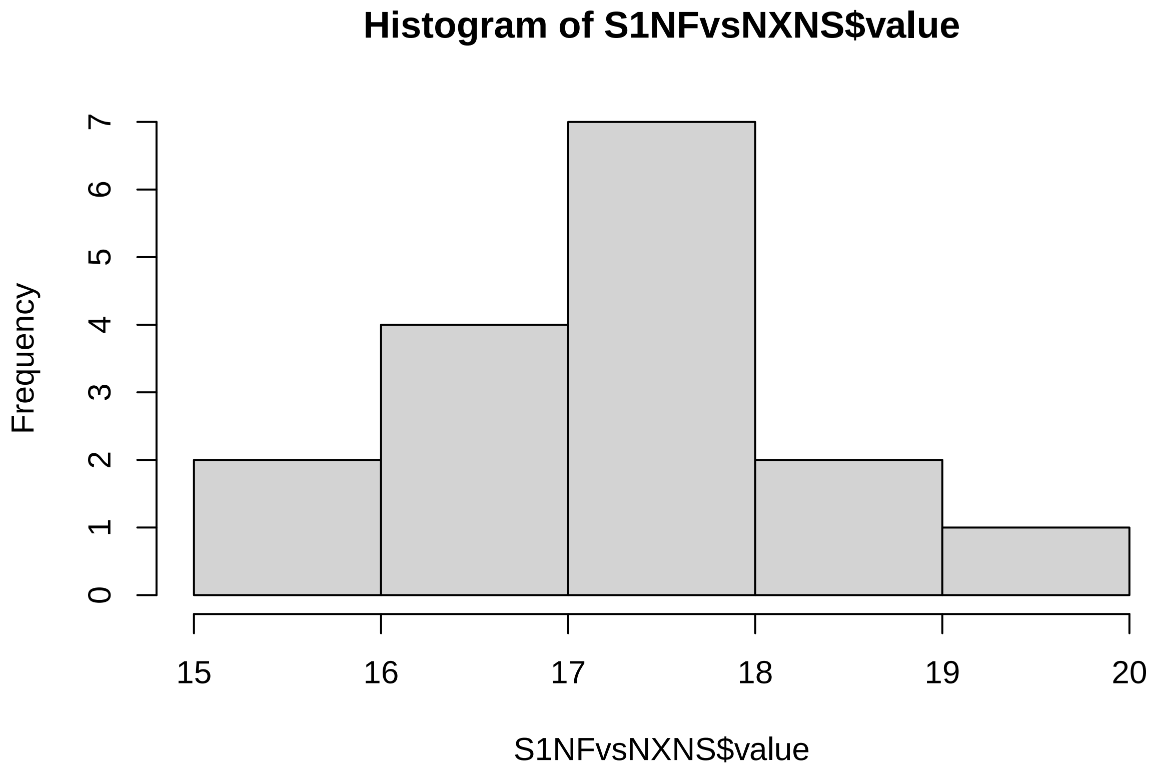


```r
#Add jitter naming column
S1NFvsNXNS<-mutate(S1NFvsNXNS, jitter="S1")
#Select comparisons where S2, 0h storage and NFHI is part of the first variable
  #(Part of the HX experiment)
S20hNFvsNXNS<-filter(meltdist, grepl('NFNS_S2_0h', Var1))
#From this include comparisons to S2, 0h storage and NFNS
S20hNFvsNXNS<-filter(S20hNFvsNXNS, grepl('NX[0-9]NS_S2_0h', Var2))
#Select comparisons where S2, 64h storage and NFHI is part of the first variable
  #(Part of the HX experiment)
S264hNFvsNXNS<-filter(meltdist, grepl('NFNS_S2_64h', Var1))
#From this include comparisons to S2, 64h storage and NFNS
S264hNFvsNXNS<-filter(S264hNFvsNXNS, grepl('NX[0-9]NS_S2_64h', Var2))
S2NFvsNXNS<-bind_rows(S20hNFvsNXNS, S264hNFvsNXNS)
#Histogram to look at distribution
hist(S2NFvsNXNS$value)
```

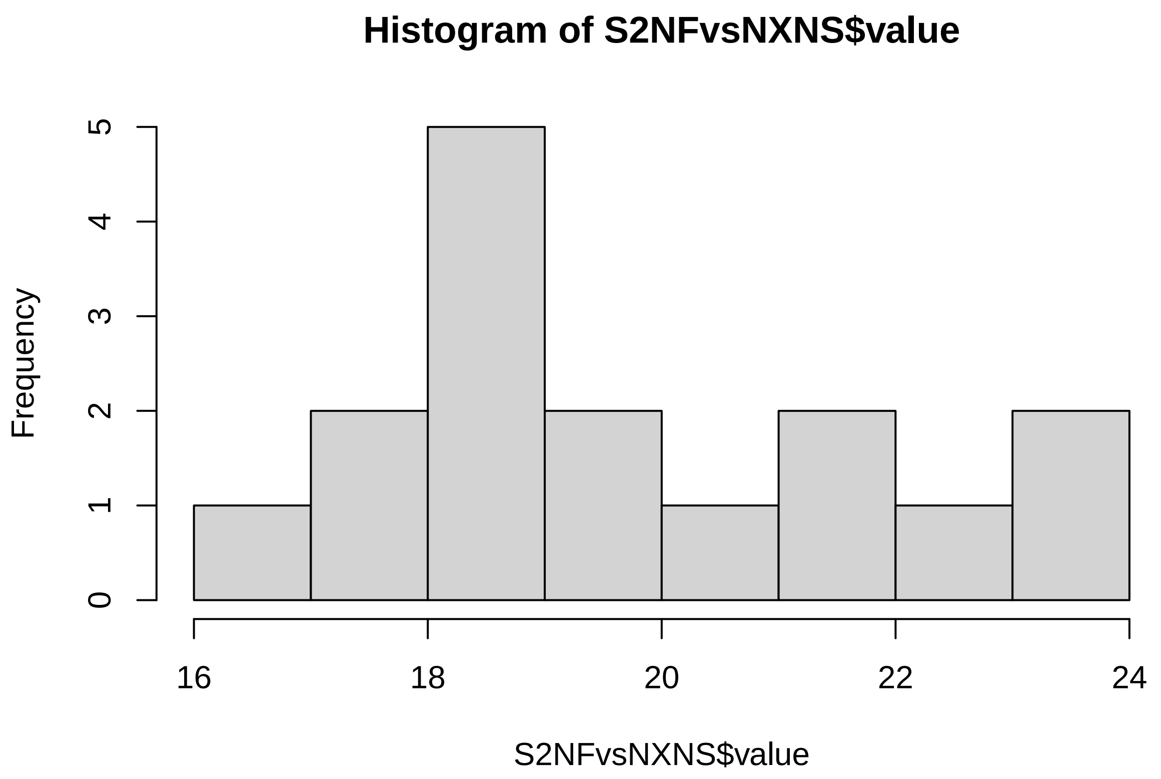


```r
#Add jitter naming column
S2NFvsNXNS<-mutate(S2NFvsNXNS, jitter="S2")
#Combine comparisons of nextflex nextera runs in all samples on nextseq
NFvsNXNS<-bind_rows(P1NFvsNXNS, P2NFvsNXNS, S1NFvsNXNS, S2NFvsNXNS)
#Histogram to look at distribution
hist(NFvsNXNS$value)
```

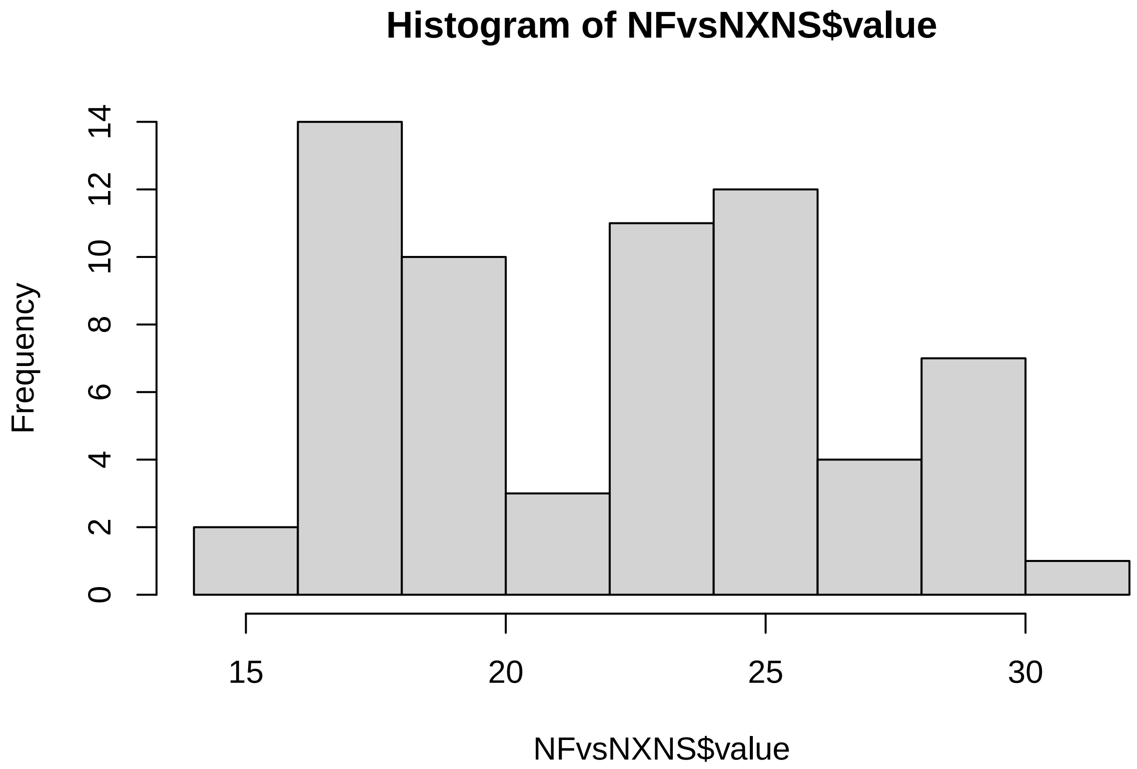


```
#Add Comparison column
NFvsNXNS<-mutate(NFvsNXNS, Comparison="NFvsNXNS")


#Distance between all LPS samples
#Select comparisons where P1, 0h storage and NFHI is part of the first variable
  #(Part of the HX experiment)
P10hLPS<-filter(meltdist, grepl('P1_0h', Var1))
#From this include comparisons to P1, 0h storage and NFNS
P10hLPS<-filter(P10hLPS, grepl('_P1_0h', Var2))
#Select comparisons where P1, 64h storage and NFHI is part of the first variable
  #(Part of the HX experiment)
P164hLPS<-filter(meltdist, grepl('P1_64h', Var1))
#From this include comparisons to P1, 64h storage and NFNS
P164hLPS<-filter(P164hLPS, grepl('_P1_64h', Var2))
P1LPS<-bind_rows(P10hLPS, P164hLPS)
#Histogram to look at distribution
hist(P1LPS$value)
```

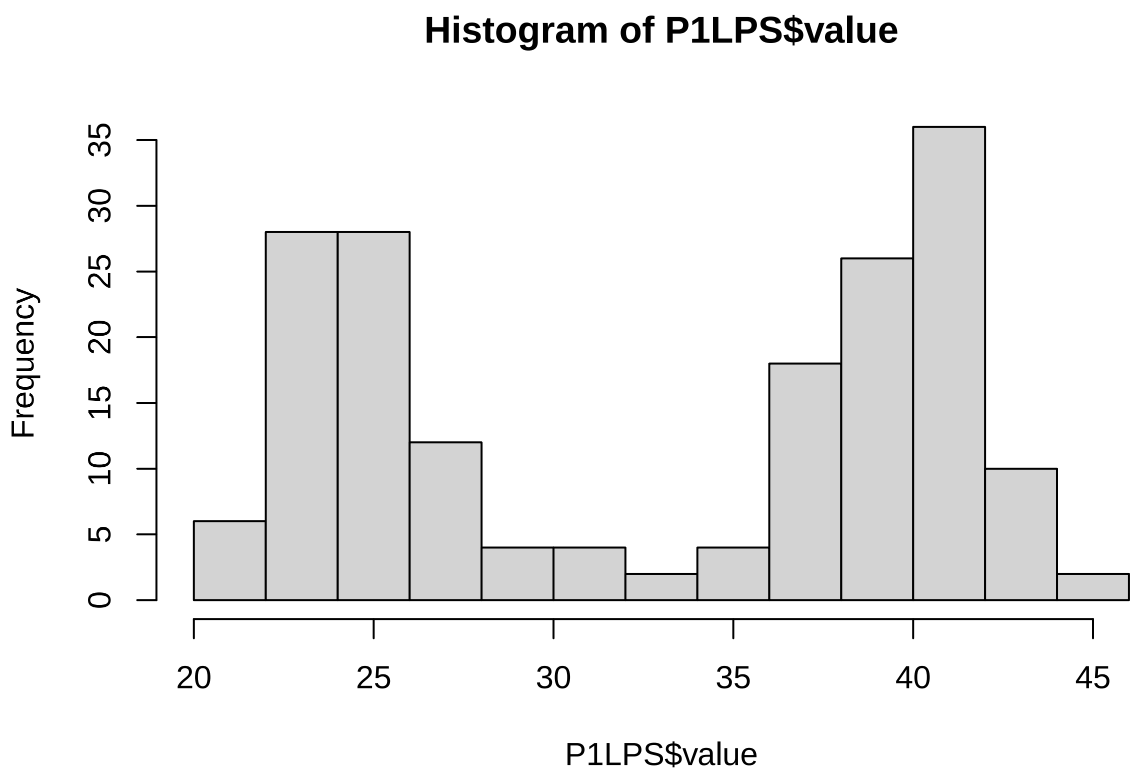


```
#Add jitter naming column
P1LPS<-mutate(P1LPS, jitter="P1")
#Select comparisons where P2, 0h storage and NFHI is part of the first variable
  #(Part of the HX experiment)
P20hLPS<-filter(meltdist, grepl('P2_0h', Var1))
#From this include comparisons to P2, 0h storage and NFNS
P20hLPS<-filter(P20hLPS, grepl('_P2_0h', Var2))
#Select comparisons where P2, 64h storage and NFHI is part of the first variable
  #(Part of the HX experiment)
P264hLPS<-filter(meltdist, grepl('P2_64h', Var1))
#From this include comparisons to P2, 64h storage and NFNS
P264hLPS<-filter(P264hLPS, grepl('_P2_64h', Var2))
P2LPS<-bind_rows(P20hLPS, P264hLPS)
#Histogram to look at distribution
hist(P2LPS$value)
```

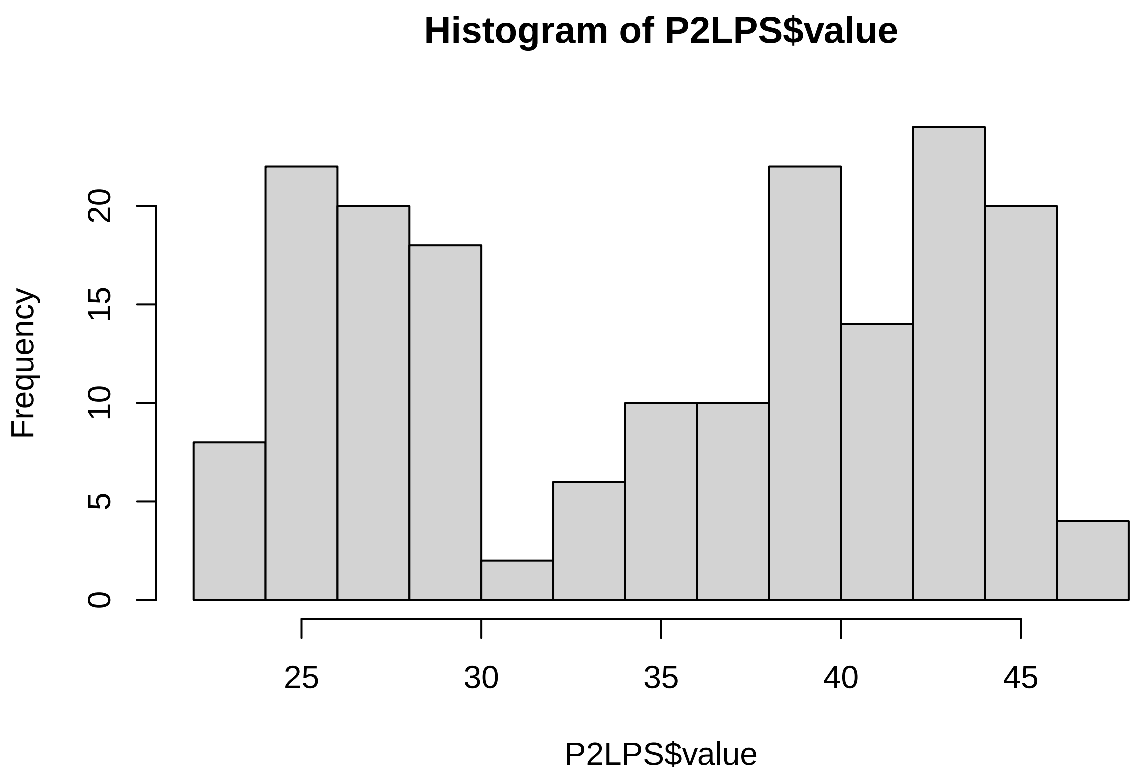


```
#Add jitter naming column
P2LPS<-mutate(P2LPS, jitter="P2")
#Select comparisons where S1, 0h storage and NFHI is part of the first variable
  #(Part of the HX experiment)
S10hLPS<-filter(meltdist, grepl('S1_0h', Var1))
#From this include comparisons to S1, 0h storage and NFNS
S10hLPS<-filter(S10hLPS, grepl('_S1_0h', Var2))
#Select comparisons where S1, 64h storage and NFHI is part of the first variable
  #(Part of the HX experiment)
S164hLPS<-filter(meltdist, grepl('S1_64h', Var1))
#From this include comparisons to S1, 64h storage and NFNS
S164hLPS<-filter(S164hLPS, grepl('_S1_64h', Var2))
S1LPS<-bind_rows(S10hLPS, S164hLPS)
#Histogram to look at distribution
hist(S1LPS$value)
```

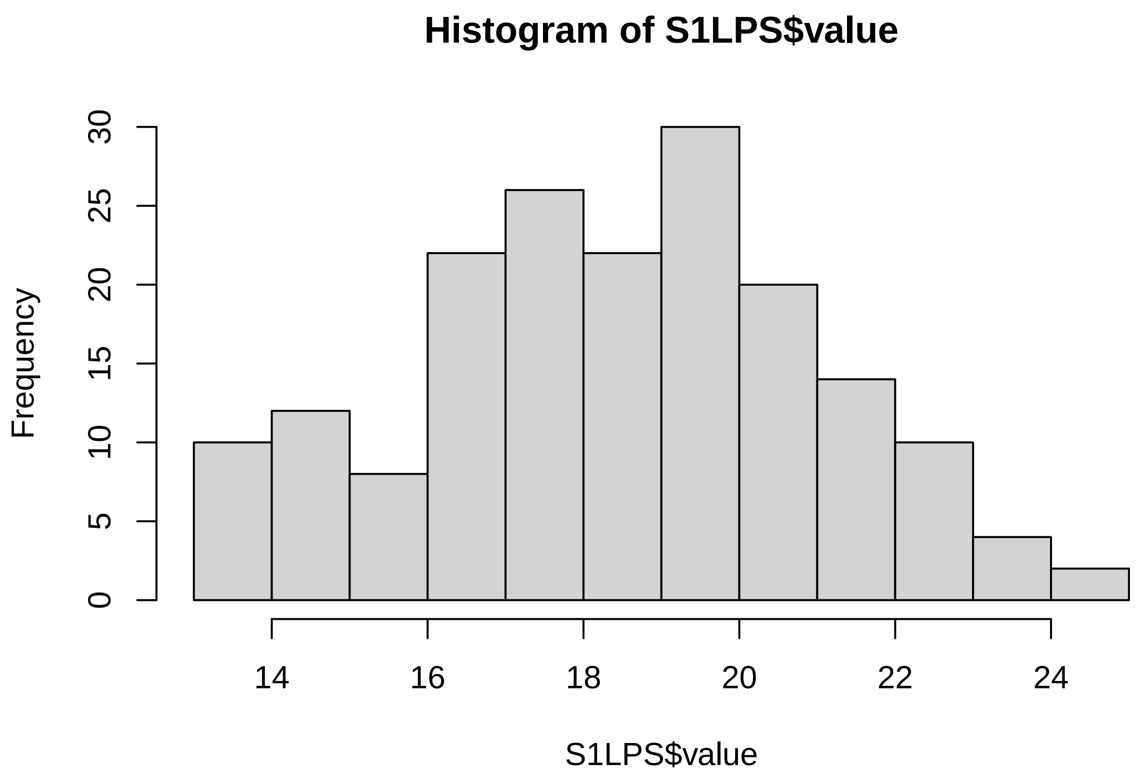


```
#Add jitter naming column
S1LPS<-mutate(S1LPS, jitter="S1")
#Select comparisons where S2, 0h storage and NFHI is part of the first variable
  #(Part of the HX experiment)
S20hLPS<-filter(meltdist, grepl('S2_0h', Var1))
#From this include comparisons to S2, 0h storage and NFNS
S20hLPS<-filter(S20hLPS, grepl('_S2_0h', Var2))
#Select comparisons where S2, 64h storage and NFHI is part of the first variable
  #(Part of the HX experiment)
S264hLPS<-filter(meltdist, grepl('S2_64h', Var1))
#From this include comparisons to S2, 64h storage and NFNS
S264hLPS<-filter(S264hLPS, grepl('_S2_64h', Var2))
S2LPS<-bind_rows(S20hLPS, S264hLPS)
#Histogram to look at distribution
hist(S2LPS$value)
```

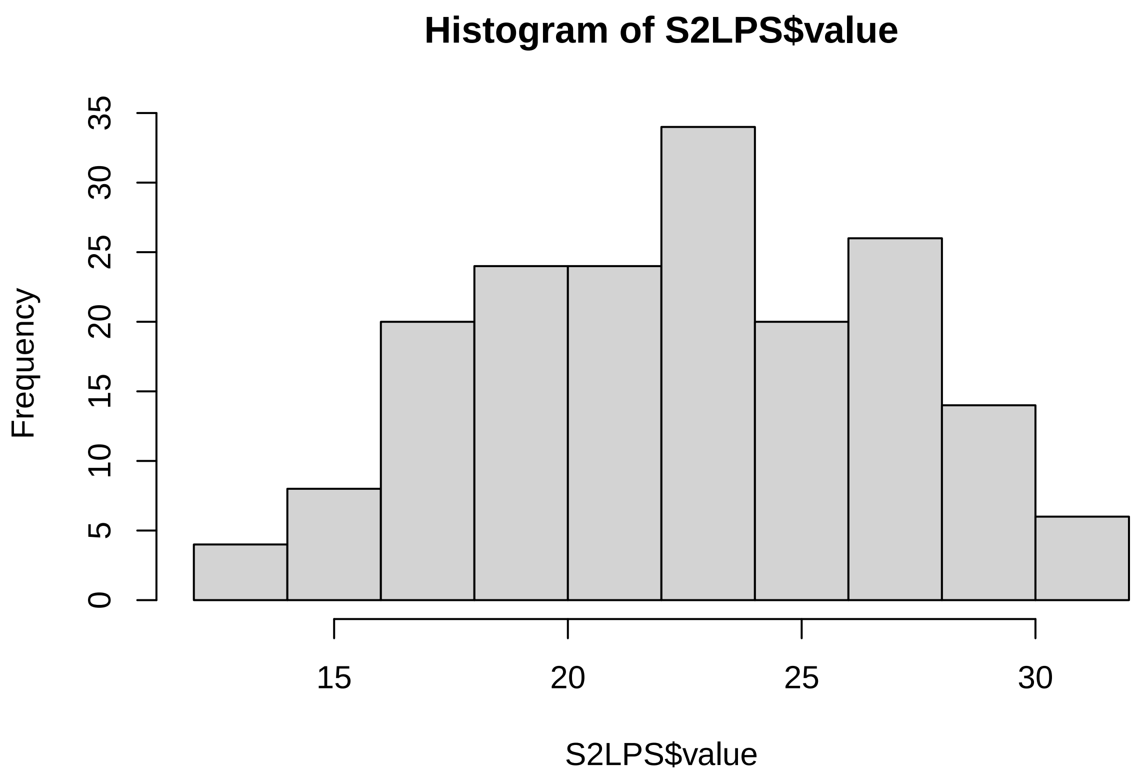


```r
#Add jitter naming column
S2LPS<-mutate(S2LPS, jitter="S2")
#Combine comparisons of nextflex nextera runs in all samples on nextseq
LPS<-bind_rows(P1LPS, P2LPS, S1LPS, S2LPS)
#Histogram to look at distribution
hist(LPS$value)
```

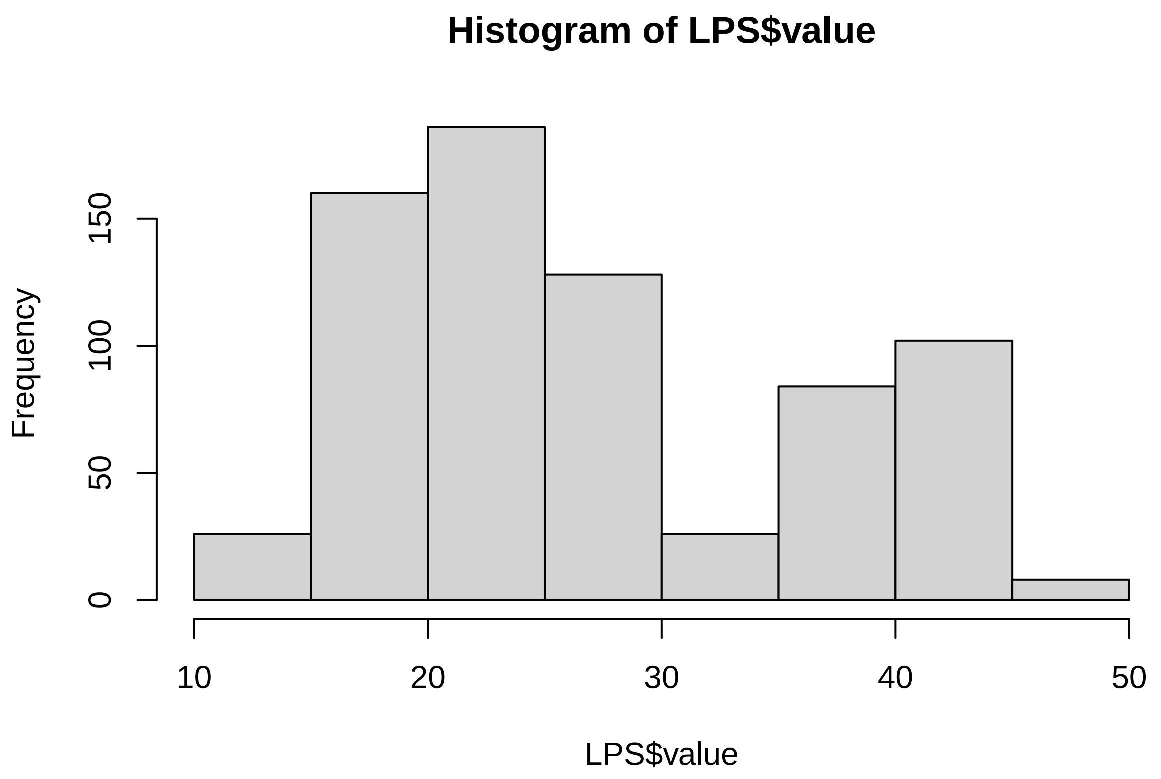


```
#Add Comparison column
LPS<-mutate(LPS, Comparison="LPS")


#Distance between storage samples
#Select comparisons where P1, Oh storage and NFHI is part of the first variable
  #(Part of the HX experiment)
P1HXStorage<-filter(meltdist, grepl('HX_P1_0h', Var1))
#From this include comparisons to P1, Oh storage and NFNS
P1HXStorage<-filter(P1HXStorage, grepl('HX_P1_64h', Var2))
#Select comparisons where P1, Oh storage and NFHI is part of the first variable
  #(Part of the HX experiment)
P1KAHIStorage<-filter(meltdist, grepl('KAHI_P1_0h', Var1))
#From this include comparisons to P1, Oh storage and NFNS
P1KAHIStorage<-filter(P1KAHIStorage, grepl('KAHI_P1_64h', Var2))
#Select comparisons where P1, Oh storage and NFHI is part of the first variable
  #(Part of the HX experiment)
P1NFNSStorage<-filter(meltdist, grepl('NFNS_P1_0h', Var1))
#From this include comparisons to P1, Oh storage and NFNS
P1NFNSStorage<-filter(P1NFNSStorage, grepl('NFNS_P1_64h', Var2))
#Select comparisons where P1, Oh storage and NFHI is part of the first variable
  #(Part of the HX experiment)
P1NX1NSStorage<-filter(meltdist, grepl('NX1NS_P1_0h', Var1))
#From this include comparisons to P1, Oh storage and NFNS
P1NX1NSStorage<-filter(P1NX1NSStorage, grepl('NX1NS_P1_64h', Var2))
#Select comparisons where P1, Oh storage and NFHI is part of the first variable
```

```
  #(Part of the HX experiment)
P1NX2NSStorage<-filter(meltdist, grepl('NX2NS_P1_0h', Var1))
#From this include comparisons to P1, 0h storage and NFNS
P1NX2NSStorage<-filter(P1NX2NSStorage, grepl('NX2NS_P1_64h', Var2))
#Select comparisons where P1, 0h storage and NFHI is part of the first variable
  #(Part of the HX experiment)
P1Storage<-bind_rows(P1HXStorage, P1KAHIStorage, P1NFNSStorage, P1NX1NSStorage,
                     P1NX2NSStorage)
#Histogram to look at distribution
hist(P1Storage$value)
```

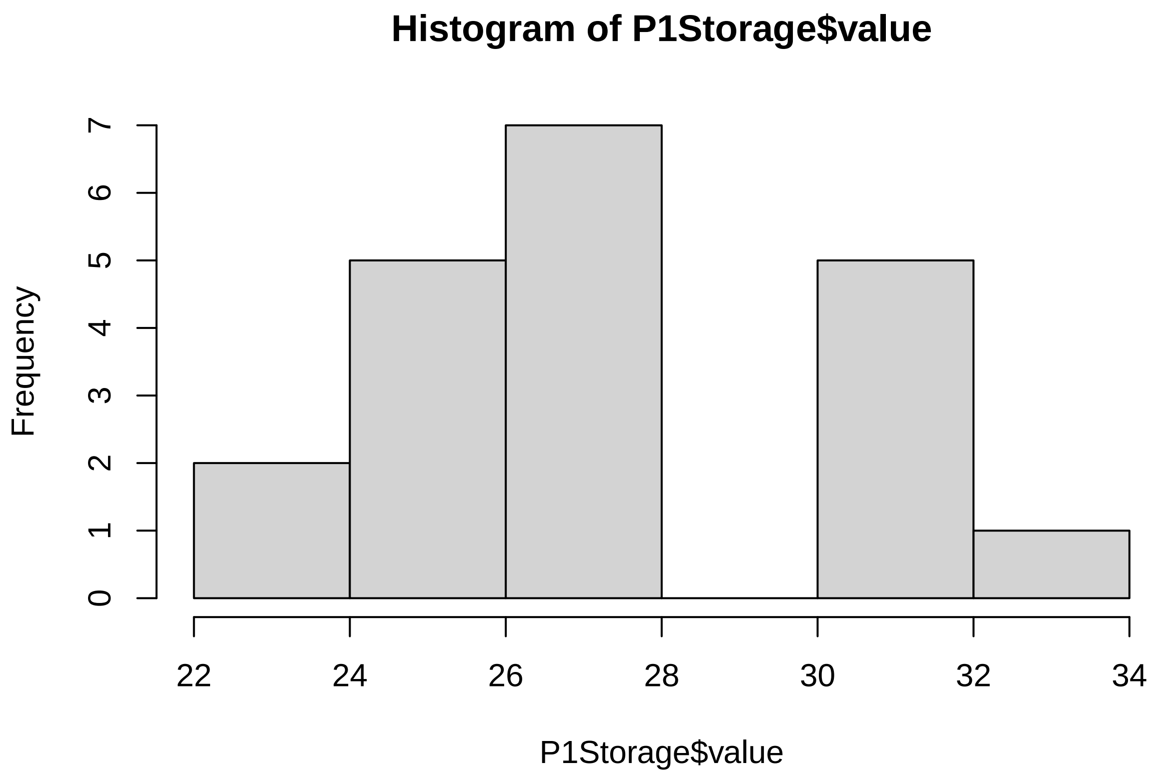


```
#Add jitter naming column
P1Storage<-mutate(P1Storage, jitter="P1")
#Select comparisons where P2, 0h storage and NFHI is part of the first variable
  #(Part of the HX experiment)
P2HXStorage<-filter(meltdist, grepl('HX_P2_0h', Var1))
#From this include comparisons to P2, 0h storage and NFNS
P2HXStorage<-filter(P2HXStorage, grepl('HX_P2_64h', Var2))
#Select comparisons where P2, 0h storage and NFHI is part of the first variable
  #(Part of the HX experiment)
P2KAHIStorage<-filter(meltdist, grepl('KAHI_P2_0h', Var1))
#From this include comparisons to P2, 0h storage and NFNS
P2KAHIStorage<-filter(P2KAHIStorage, grepl('KAHI_P2_64h', Var2))
#Select comparisons where P2, 0h storage and NFHI is part of the first variable
  #(Part of the HX experiment)
P2NFNSStorage<-filter(meltdist, grepl('NFNS_P2_0h', Var1))
```

```r
#From this include comparisons to P2, 0h storage and NFNS
P2NFNSStorage<-filter(P2NFNSStorage, grepl('NFNS_P2_64h', Var2))
#Select comparisons where P2, 0h storage and NFHI is part of the first variable
  #(Part of the HX experiment)
P2NX1NSStorage<-filter(meltdist, grepl('NX1NS_P2_0h', Var1))
#From this include comparisons to P2, 0h storage and NFNS
P2NX1NSStorage<-filter(P2NX1NSStorage, grepl('NX1NS_P2_64h', Var2))
#Select comparisons where P2, 0h storage and NFHI is part of the first variable
  #(Part of the HX experiment)
P2NX2NSStorage<-filter(meltdist, grepl('NX2NS_P2_0h', Var1))
#From this include comparisons to P2, 0h storage and NFNS
P2NX2NSStorage<-filter(P2NX2NSStorage, grepl('NX2NS_P2_64h', Var2))
#Select comparisons where P2, 0h storage and NFHI is part of the first variable
  #(Part of the HX experiment)
P2Storage<-bind_rows(P2HXStorage, P2KAHIStorage, P2NFNSStorage, P2NX1NSStorage,
                     P2NX2NSStorage)
#Histogram to look at distribution
hist(P2Storage$value)
```

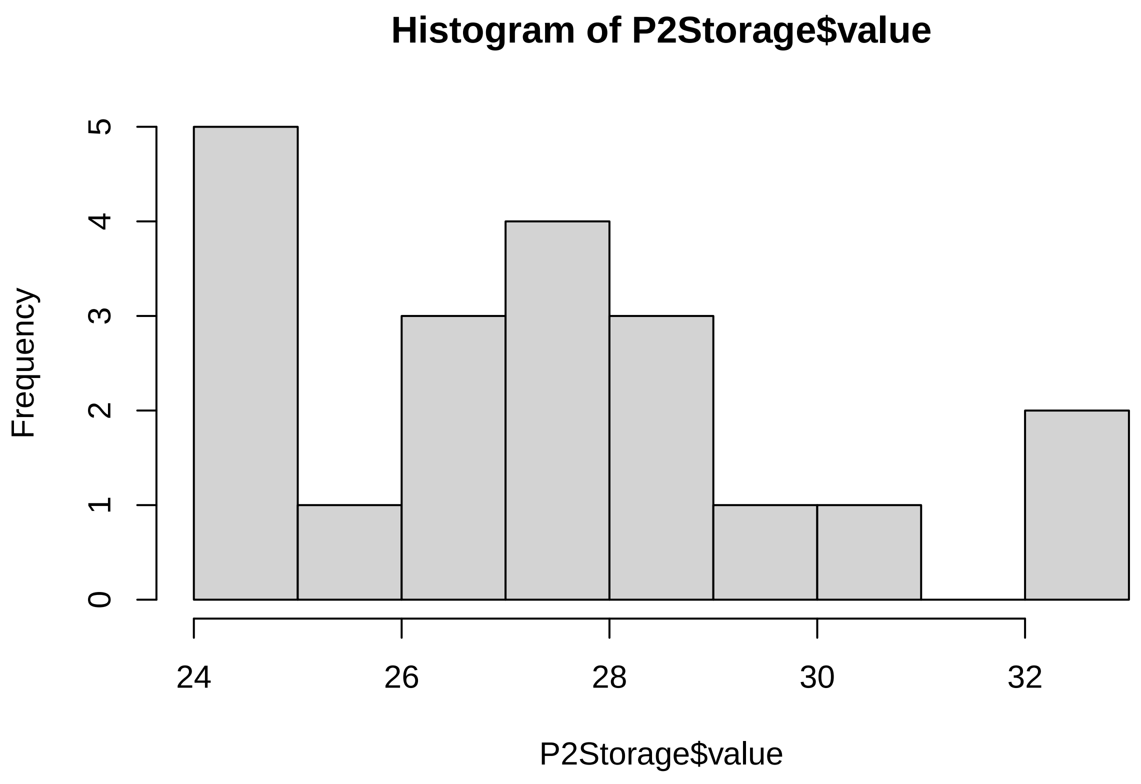


```r
#Add jitter naming column
P2Storage<-mutate(P2Storage, jitter="P2")
#Select comparisons where S1, 0h storage and NFHI is part of the first variable
  #(Part of the HX experiment)
S1HXStorage<-filter(meltdist, grepl('HX_S1_0h', Var1))
#From this include comparisons to S1, 0h storage and NFNS
S1HXStorage<-filter(S1HXStorage, grepl('HX_S1_64h', Var2))
```

```r
#Select comparisons where S1, 0h storage and NFHI is part of the first variable
  #(Part of the HX experiment)
S1KAHIStorage<-filter(meltdist, grepl('KAHI_S1_0h', Var1))
#From this include comparisons to S1, 0h storage and NFNS
S1KAHIStorage<-filter(S1KAHIStorage, grepl('KAHI_S1_64h', Var2))
#Select comparisons where S1, 0h storage and NFHI is part of the first variable
  #(Part of the HX experiment)
S1NFNSStorage<-filter(meltdist, grepl('NFNS_S1_0h', Var1))
#From this include comparisons to S1, 0h storage and NFNS
S1NFNSStorage<-filter(S1NFNSStorage, grepl('NFNS_S1_64h', Var2))
#Select comparisons where S1, 0h storage and NFHI is part of the first variable
  #(Part of the HX experiment)
S1NX1NSStorage<-filter(meltdist, grepl('NX1NS_S1_0h', Var1))
#From this include comparisons to S1, 0h storage and NFNS
S1NX1NSStorage<-filter(S1NX1NSStorage, grepl('NX1NS_S1_64h', Var2))
#Select comparisons where S1, 0h storage and NFHI is part of the first variable
  #(Part of the HX experiment)
S1NX2NSStorage<-filter(meltdist, grepl('NX2NS_S1_0h', Var1))
#From this include comparisons to S1, 0h storage and NFNS
S1NX2NSStorage<-filter(S1NX2NSStorage, grepl('NX2NS_S1_64h', Var2))
#Select comparisons where S1, 0h storage and NFHI is part of the first variable
  #(Part of the HX experiment)
S1Storage<-bind_rows(S1HXStorage, S1KAHIStorage, S1NFNSStorage, S1NX1NSStorage,
                     S1NX2NSStorage)
#Histogram to look at distribution
hist(S1Storage$value)
```

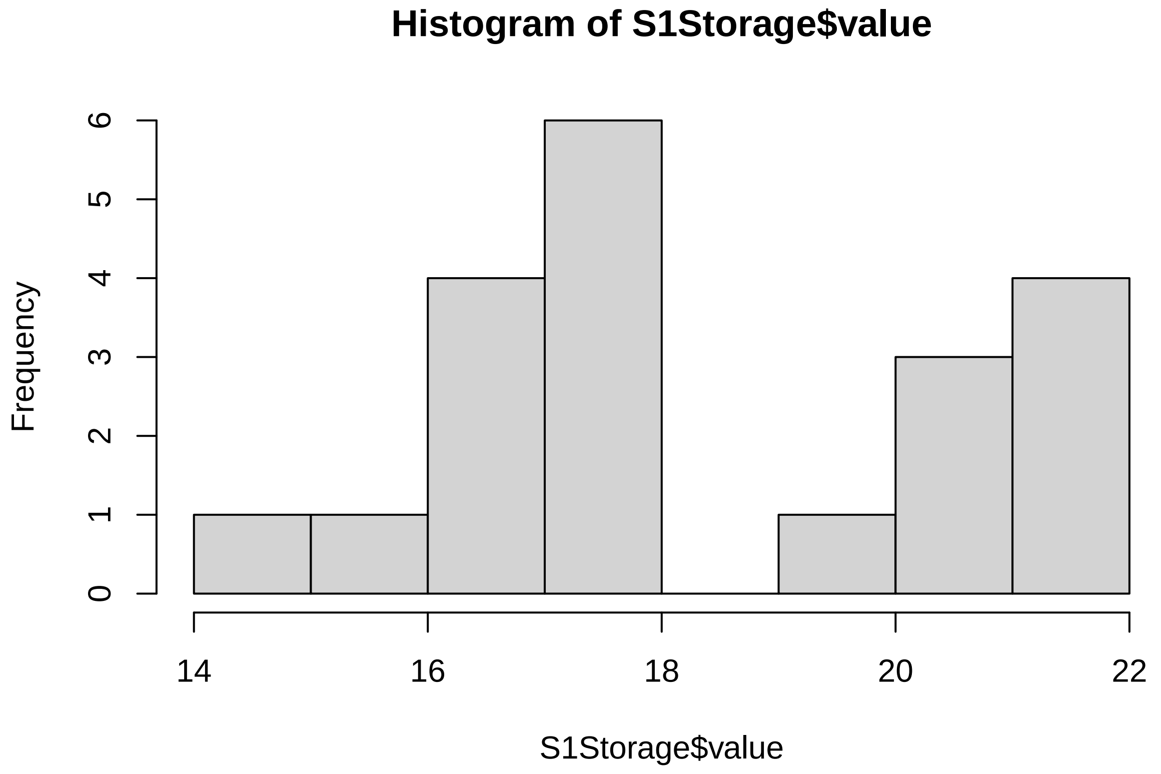

```r
#Add jitter naming column
S1Storage<-mutate(S1Storage, jitter="S1")
#Select comparisons where S2, Oh storage and NFHI is part of the first variable
  #(Part of the HX experiment)
S2HXStorage<-filter(meltdist, grepl('HX_S2_0h', Var1))
#From this include comparisons to S2, Oh storage and NFNS
S2HXStorage<-filter(S2HXStorage, grepl('HX_S2_64h', Var2))
#Select comparisons where S2, Oh storage and NFHI is part of the first variable
  #(Part of the HX experiment)
S2KAHIStorage<-filter(meltdist, grepl('KAHI_S2_0h', Var1))
#From this include comparisons to S2, Oh storage and NFNS
S2KAHIStorage<-filter(S2KAHIStorage, grepl('KAHI_S2_64h', Var2))
#Select comparisons where S2, Oh storage and NFHI is part of the first variable
  #(Part of the HX experiment)
S2NFNSStorage<-filter(meltdist, grepl('NFNS_S2_0h', Var1))
#From this include comparisons to S2, Oh storage and NFNS
S2NFNSStorage<-filter(S2NFNSStorage, grepl('NFNS_S2_64h', Var2))
#Select comparisons where S2, Oh storage and NFHI is part of the first variable
  #(Part of the HX experiment)
S2NX1NSStorage<-filter(meltdist, grepl('NX1NS_S2_0h', Var1))
#From this include comparisons to S2, Oh storage and NFNS
S2NX1NSStorage<-filter(S2NX1NSStorage, grepl('NX1NS_S2_64h', Var2))
#Select comparisons where S2, Oh storage and NFHI is part of the first variable
  #(Part of the HX experiment)
S2NX2NSStorage<-filter(meltdist, grepl('NX2NS_S2_0h', Var1))
#From this include comparisons to S2, Oh storage and NFNS
S2NX2NSStorage<-filter(S2NX2NSStorage, grepl('NX2NS_S2_64h', Var2))
#Select comparisons where S2, Oh storage and NFHI is part of the first variable
  #(Part of the HX experiment)
S2Storage<-bind_rows(S2HXStorage, S2KAHIStorage, S2NFNSStorage, S2NX1NSStorage,
                     S2NX2NSStorage)
#Histogram to look at distribution
hist(S2Storage$value)
```

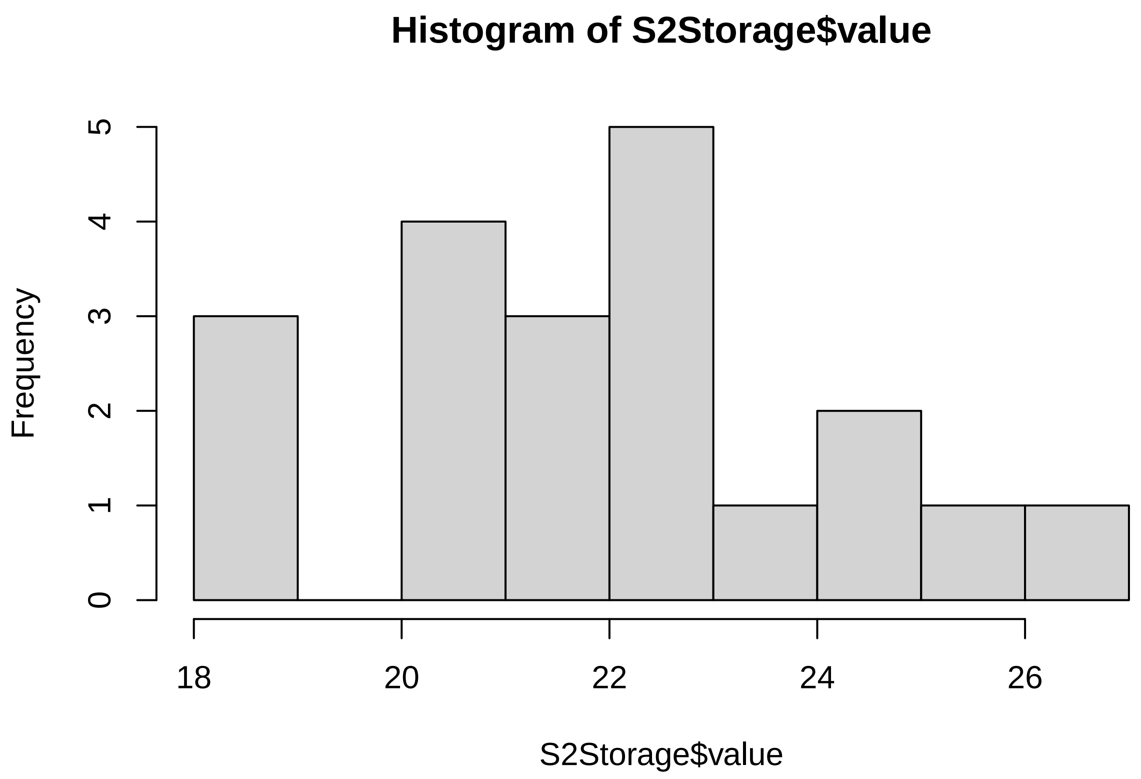


```
#Add jitter naming column
S2Storage<-mutate(S2Storage, jitter="S2")
#Combine comparisons of nextflex nextera runs in all samples on nextseq
Storage<-bind_rows(P1Storage, P2Storage, S1Storage, S2Storage)
#Histogram to look at distribution
hist(Storage$value)
```

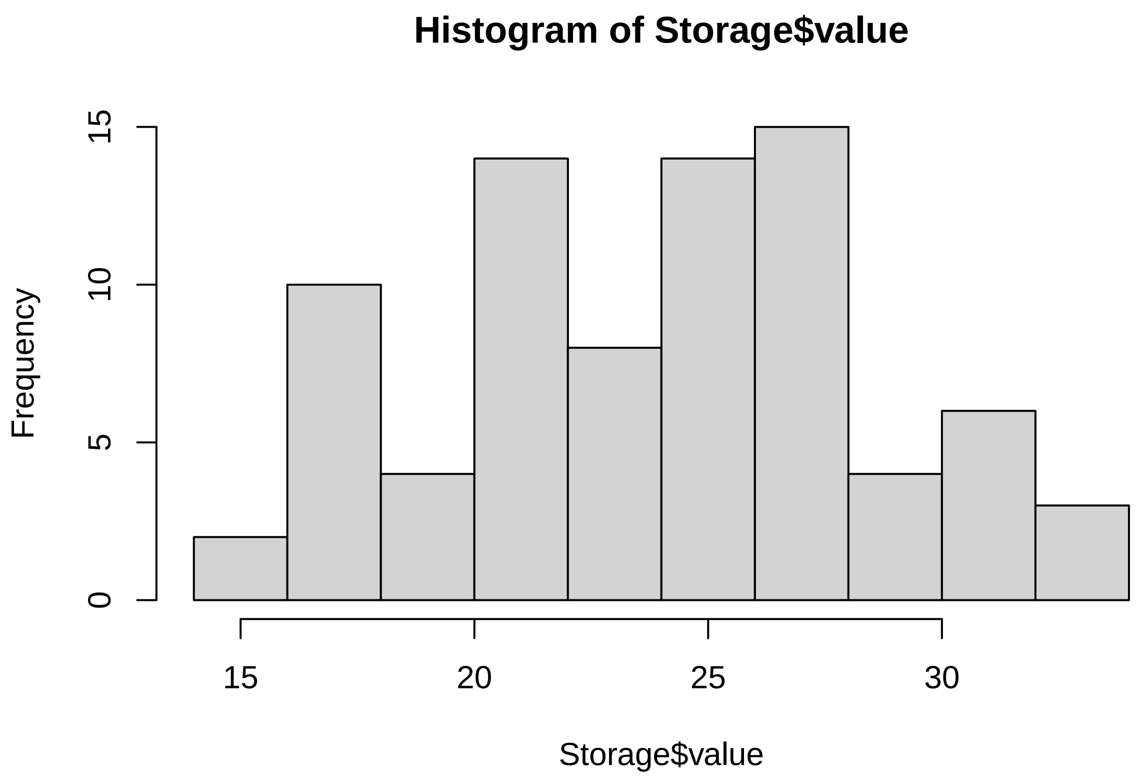


```
#Add Comparison column
Storage<-mutate(Storage, Comparison="Storage")


#Combine selected comparisons
boxplotting<-bind_rows(P1vsP2, S1vsS2, P1vsP1, P2vsP2, S1vsS1, S2vsS2, NX1vsNX2, DNAExRep,
                       NFHSvsNS, NFvsKAHI, NFvsNXNS, LPS, Storage) #Removed PFvsSW makes
  #other differences difficult to see.

#Create boxplot
#Defining the order, can change if needed
boxplotting$Comparison<-factor(boxplotting$Comparison,
                               levels= c("P1vsP1", "P2vsP2", "P1vsP2", "S1vsS1", "S2vsS2",
                                         "S1vsS2", "DNAExRep","NXNSRep", "LPS", "Storage",
                                         "NFHSvsNS", "NFvsKAHI", "NFvsNXNS"))
boxplotting$jitter<-factor(boxplotting$jitter, levels= c("P1vsP2", "S1vsS2", "P1", "P2",
                                                         "S1", "S2"))

#pdf(paste("LPSXSFig2BoxplotPWD", ".pdf", sep=""), height=6, width=12)
ggplot(boxplotting, aes(x=Comparison, y=value)) +
  geom_boxplot() +
  labs(x="Compared sample groups", y="Distance") +
  theme_bw() +
  theme(panel.border = element_blank(), panel.grid.major = element_blank(),
        panel.grid.minor = element_blank(), axis.line = element_line(colour = "black"),
        axis.text.x = element_text(size=12, angle = 45, hjust = 1),
```

```
        axis.title=element_text(size=14))
```

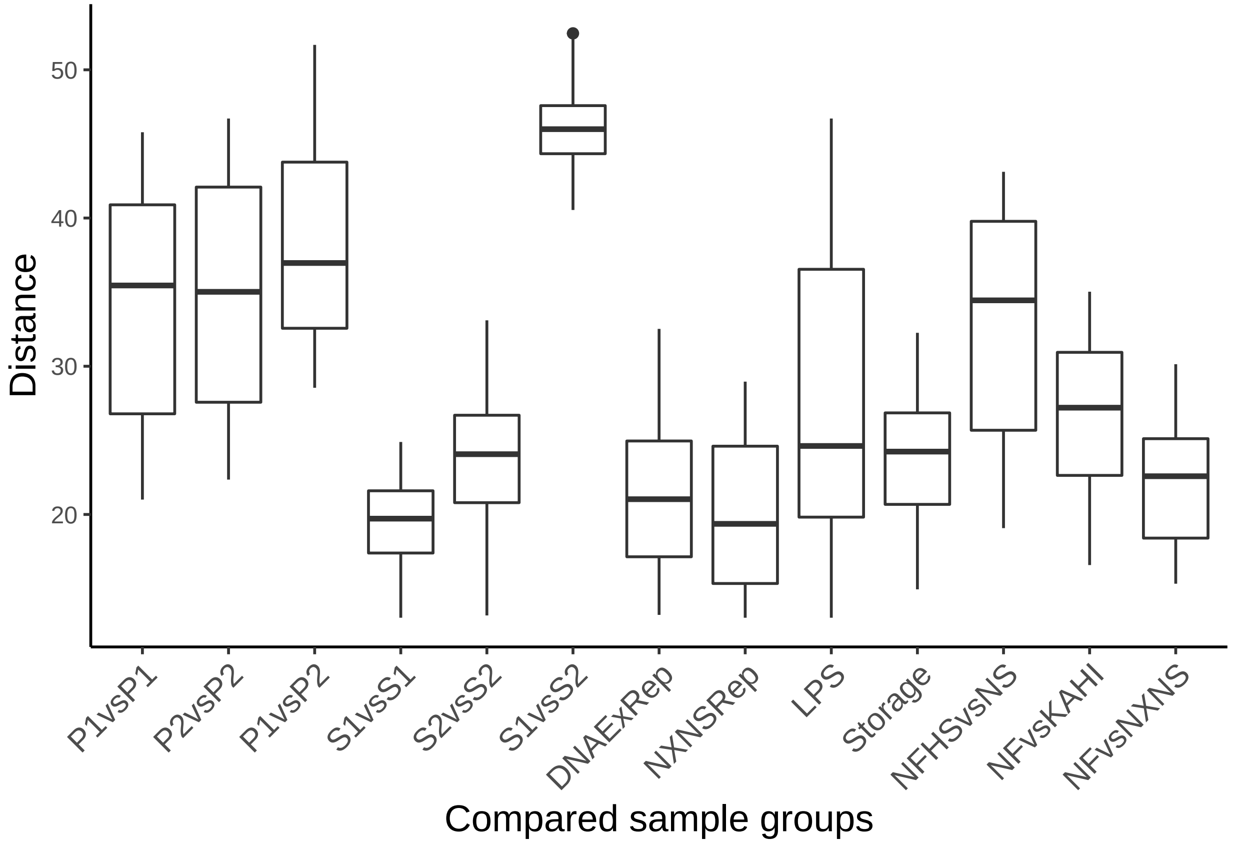


```
#dev.off()

#Trying to make fancy boxplots + geom_jitter(), might want to
jitter=c(PFvsSW = "Darkblue", P1vsP2 = "Darkblue", S1vsS2 = "Darkblue", P1 = "#30241E",
         P2 = "#B59B80", S1 = "#33a02c", S2 = "#B2DF8A")
pdf(paste("Fig1_BoxplotsPWD", ".pdf", sep=""), height=6, width=12)
  #PWD = Pairwise distances
ggplot(boxplotting, aes(x=Comparison, y=value)) +
  geom_boxplot() +
  labs(x="Compared sample groups", y="Distance") +
  theme_bw() +
  theme(panel.border = element_blank(), panel.grid.major = element_blank(),
        panel.grid.minor = element_blank(), axis.line = element_line(colour = "black"),
        axis.text.x = element_text(size=12, angle = 45, hjust = 1),
        axis.title=element_text(size=14)) +
  geom_jitter(aes(color=jitter), size=0.7) +
  scale_color_manual(values = jitter) +
  guides(colour = guide_legend(override.aes = list(size=5)))
dev.off()
```

```
## pdf
##   2
```

sPLS-DA

Used to find most important features for describing library preparation and sequencing platform. Overview represented as S4_Fig

```r
rm(list=setdiff(ls(), c("Metadata", "Feature", "Tax")))

#Subset data pig feces 1 and 2 (P1, P2), Sewage 1 and 2 (S1, S2), or spiked unspiked
Subset <- "All" #All, Allspiked, Allunspiked, PF, SW, P1, P1spiked, P1unspiked, P2, P2spiked, P2unspike

#Subset experiment, Experiment_Type. (Meaningfull combinations of subset and SubExp: HX=All, FTX=P1&S1,
SubExp<-"LPSX" #HX, FTX, LTX, LPSX, HXFTX, HXLTX, HXLPSX, HXLTXLPSX, HXFTXLTX, All

#Subset Frozen Unfrozen
SubFre<-"Both" #Frozen, Unfrozen, Both

#make empty list for plots
sPLSList=list()

#Removing negative and positive controls
Metadata2<-filter(Metadata, Sample_type_simple=="Sample")

#Subsetting Metadata2
if (Subset=="Allspiked") {
  Metadata2<-filter(Metadata2, SpikedUnspiked == "Spiked")
} else if (Subset=="Allunspiked") {
  Metadata2<-filter(Metadata2, SpikedUnspiked == "Unspiked")
} else if (Subset=="PF") {
  Metadata2<-filter(Metadata2, Experiment == "Pig_feces_1" | Experiment == "Pig_feces_2")
} else if (Subset=="PFspiked") {
  Metadata2<-filter(Metadata2, Sample_type == "Pig_feces_1_spiked" |
                    Sample_type == "Pig_feces_2_spiked")
} else if (Subset=="PFunspiked") {
  Metadata2<-filter(Metadata2, Sample_type == "Pig_feces_1" |
                    Sample_type == "Pig_feces_2")
} else if (Subset=="SW") {
  Metadata2<-filter(Metadata2, Experiment == "Sewage_1" | Experiment == "Sewage_2")
} else if (Subset=="SWspiked") {
  Metadata2<-filter(Metadata2, Sample_type == "Sewage_1_spiked" |
                    Sample_type == "Sewage_2_spiked")
} else if (Subset=="SWunspiked") {
  Metadata2<-filter(Metadata2, Sample_type == "Sewage_1" | Sample_type == "Sewage_2")
} else if (Subset=="P1") {
  Metadata2<-filter(Metadata2, Experiment == "Pig_feces_1")
} else if (Subset=="P1spiked") {
  Metadata2<-filter(Metadata2, Sample_type == "Pig_feces_1_spiked")
} else if (Subset=="P1unspiked") {
  Metadata2<-filter(Metadata2, Sample_type == "Pig_feces_1")
} else if (Subset=="P2") {
  Metadata2<-filter(Metadata2, Experiment == "Pig_feces_2")
} else if (Subset=="P2spiked") {
  Metadata2<-filter(Metadata2, Sample_type == "Pig_feces_2_spiked")
} else if (Subset=="P2unspiked") {
  Metadata2<-filter(Metadata2, Sample_type == "Pig_feces_2")
} else if (Subset=="S1") {
```

```r
  Metadata2<-filter(Metadata2, Experiment == "Sewage_1")
} else if (Subset=="S1spiked") {
  Metadata2<-filter(Metadata2, Sample_type == "Sewage_1_spiked")
} else if (Subset=="S1unspiked") {
  Metadata2<-filter(Metadata2, Sample_type == "Sewage_1")
} else if (Subset=="S2") {
  Metadata2<-filter(Metadata2, Experiment == "Sewage_2")
} else if (Subset=="S2spiked") {
  Metadata2<-filter(Metadata2, Sample_type == "Sewage_2_spiked")
} else if (Subset=="S2unspiked") {
  Metadata2<-filter(Metadata2, Sample_type == "Sewage_2")
} else if (Subset=="All") {
  print("No subsetting, all included")
} else {
  print("Subset defined not valid")
}
```

```
## [1] "No subsetting, all included"
```

```r
#Further subsetting Metadata2
if (SubExp=="HX") {
  Metadata2<-filter(Metadata2, Experiment_type == "Handling_experiment")
} else if (SubExp=="FTX") {
  Metadata2<-filter(Metadata2, Experiment_type == "Freeze_thaw_experiment")
} else if (SubExp=="LTX") {
  Metadata2<-filter(Metadata2, Experiment_type == "Long_term_storage_experiment")
} else if (SubExp=="LPSX") {
  Metadata22<-filter(Metadata2, Experiment_type == "Library_prep_seq_platform_experiment")
  VectorLPSX <- unique(Metadata22$Matching_samples)
  Metadata2<-filter(Metadata2, Matching_samples %in% VectorLPSX)
  rm(VectorLPSX, Metadata22)
} else if (SubExp=="HXFTX") {
  Metadata2<-filter(Metadata2, Experiment_type == "Handling_experiment" |
                      Experiment_type == "Freeze_thaw_experiment")
} else if (SubExp=="HXLTX") {
  Metadata2<-filter(Metadata2, Experiment_type == "Handling_experiment" |
                      Experiment_type == "Long_term_storage_experiment")
} else if (SubExp=="HXLPSX") {
  Metadata2<-filter(Metadata2, Experiment_type == "Handling_experiment" |
                      Experiment_type == "Library_prep_seq_platform_experiment")
} else if (SubExp=="HXLTXLPSX") {
  Metadata2<-filter(Metadata2, Experiment_type == "Handling_experiment" |
                      Experiment_type == "Long_term_storage_experiment" |
                      Experiment_type == "Library_prep_seq_platform_experiment")
} else if (SubExp=="HXFTXLTX") {
  Metadata2<-filter(Metadata2, Experiment_type == "Handling_experiment" |
                      Experiment_type == "Freeze_thaw_experiment" |
                      Experiment_type == "Long_term_storage_experiment")
} else if (SubExp=="All") {
  print("No subsetting, all included")
} else {
  print("Subset defined not valid")
}
```

```
#Further subsetting Metadata2
if (SubFre=="Frozen") {
  Metadata2<-filter(Metadata2, FrozenUnfrozenSimple == "Freezer")
} else if (SubFre=="Unfrozen") {
  Metadata2<-filter(Metadata2, FrozenUnfrozenSimple == "Unfrozen")
} else if (SubFre=="Both") {
  print("No subsetting, all included")
} else {
  print("Subset defined not valid")
}
```

```
## [1] "No subsetting, all included"
```

```
#Removing the Kappa NextSeq run
Metadata2<-Metadata2[grep("\\KANS.*", Metadata2$Sample, invert=TRUE),]

#Applying subsetting to OTU tables
Tax2<-dplyr::select(Tax, one_of(Metadata2$Sample))

#Note, that this is done on the full set, not just the shown. Makes sense eventhough not
  #show in heatmap they can be in the clustering calculations, this also means samples can
  #look more similar in the heatmap but not cluster as closely. Also calculates on the not
  #log transformed data.
#filtering of the Counttable depending on rowSums.
Tax2 <- Tax2[rowSums(Tax2)>0,] #Removing all rows that only contains zeroes
Tax2 <- Tax2[rowSums(Tax2)>(5*ncol(Tax2)),] #Removing all rows(Species) that is below an
  #average count of 5
# replace 0 values with an estimate using simple multiplicative replacement
Tax2 <- t(cmultRepl(t(Tax2), method="CZM", label=0))
```

```
## No. corrected values:  4863
```

```
#Maks TSS
Tax2<-as.matrix(t(sweep(Tax2, 2, colSums(Tax2), FUN="/"))) #Transpose to have genera as
  #columns and samples as rows

##Interest from the following sPLS-DA
#GeneraSel <- dplyr::select(data.frame(Tax2), one_of(c("Methanosaeta", "Polaribacter",
  #"Tenacibaculum", "Porphyromonas", "Gulbenkiania", "Cellulophaga")))
#row.names(GeneraSel)==Metadata2$Sample
#GeneraSel<-bind_cols(GeneraSel, Metadata2)
#Methanosaeta<-summarySE(data=GeneraSel, "Methanosaeta",
  #groupvars=c("Sample_type_RF", "Library_preparation", "Sequencing_platform"))
#Polaribacter<-summarySE(data=GeneraSel, "Polaribacter",
  #groupvars=c("Sample_type_RF", "Library_preparation", "Sequencing_platform"))
#Tenacibaculum<-summarySE(data=GeneraSel, "Tenacibaculum",
  #groupvars=c("Sample_type_RF", "Library_preparation", "Sequencing_platform"))
#Porphyromonas<-summarySE(data=GeneraSel, "Porphyromonas",
  #groupvars=c("Sample_type_RF", "Library_preparation", "Sequencing_platform"))
#Gulbenkiania<-summarySE(data=GeneraSel, "Gulbenkiania",
  #groupvars=c("Sample_type_RF", "Library_preparation", "Sequencing_platform"))
#Cellulophaga<-summarySE(data=GeneraSel, "Cellulophaga",
  #groupvars=c("Sample_type_RF", "Library_preparation", "Sequencing_platform"))

# CLR is included in the mixOmics functions
```

```r
#Is metadata the same order as Tax2
Metadata2$Sample==row.names(Tax2)
```

```
##  [1] TRUE TRUE TRUE TRUE TRUE TRUE TRUE TRUE TRUE TRUE TRUE TRUE TRUE TRUE TRUE
## [16] TRUE TRUE TRUE TRUE TRUE TRUE TRUE TRUE TRUE TRUE TRUE TRUE TRUE TRUE TRUE
## [31] TRUE TRUE TRUE TRUE TRUE TRUE TRUE TRUE TRUE TRUE TRUE TRUE TRUE TRUE TRUE
## [46] TRUE TRUE TRUE TRUE TRUE TRUE TRUE TRUE TRUE TRUE TRUE TRUE TRUE TRUE TRUE
## [61] TRUE TRUE TRUE TRUE TRUE TRUE TRUE TRUE TRUE TRUE TRUE TRUE TRUE TRUE TRUE
## [76] TRUE TRUE TRUE TRUE TRUE
```

```r
# the outcome
Y <- paste(Metadata2$Library_preparation, Metadata2$Sequencing_platform, sep="")
# unique ID of each individual for multilevel analysis
sample <- paste(Metadata2$Sample_type_RF, Metadata2$Replicate, sep="")

#Analysis included in
  #http://mixomics.org/mixmc/case-study-hmp-bodysites-repeated-measures/
diverse.pca = mixOmics::pca(Tax2, ncomp = 10, logratio = 'CLR', multilevel = sample)
#diverse.pca
plot(diverse.pca)
```

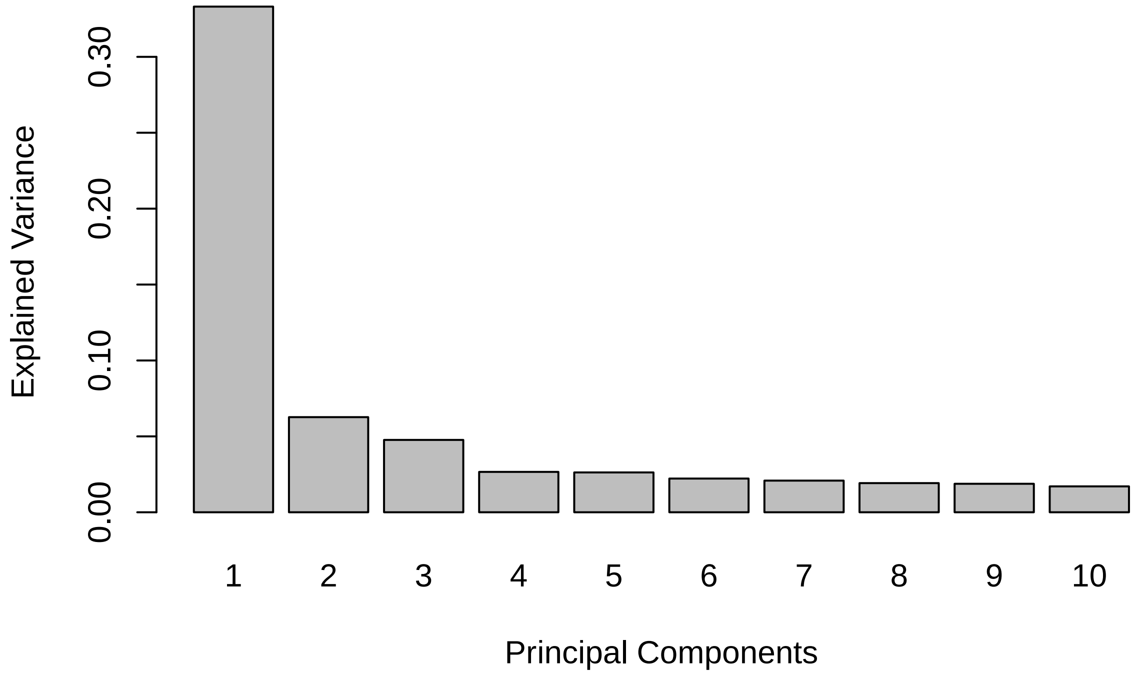


```r
plotIndiv(diverse.pca,
          comp = c(1,2), # the components to plot
          pch = 16,
          ind.names = F,
          group = Y,
```

```
        col.per.group = color.mixo(1:4),
        legend = TRUE,
        title = 'HMP most diverse, PCA comp 1 - 2')
```

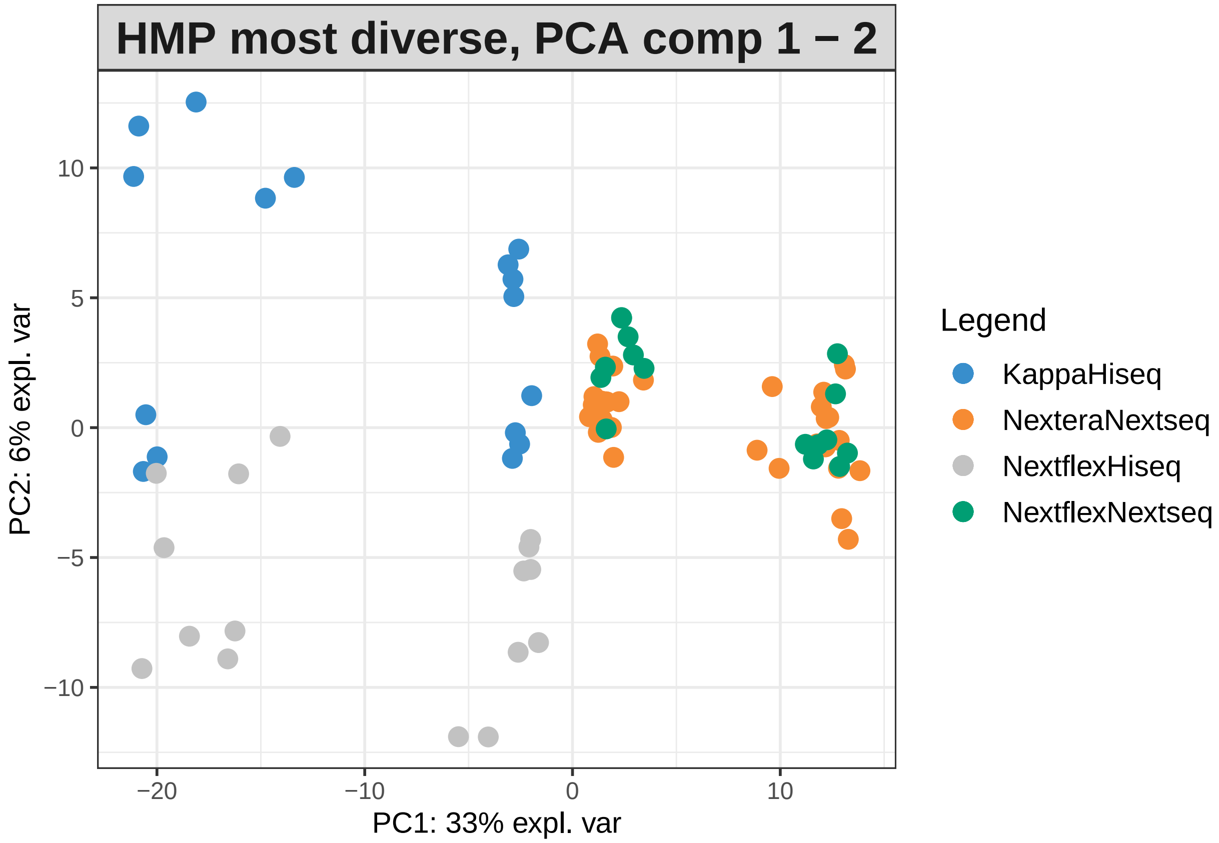


```
#Supervised analysis
diverse.plsda = plsda(X = Tax2, Y, ncomp = 10, logratio = 'CLR', multilevel = sample)
  #nlevels(Y), changed ncomp to 10

diverse.perf.plsda = perf(diverse.plsda, validation = 'Mfold', folds = 5,
                    progressBar = FALSE, nrepeat = 10)

plot(diverse.perf.plsda, overlay = 'measure', sd = TRUE)
```

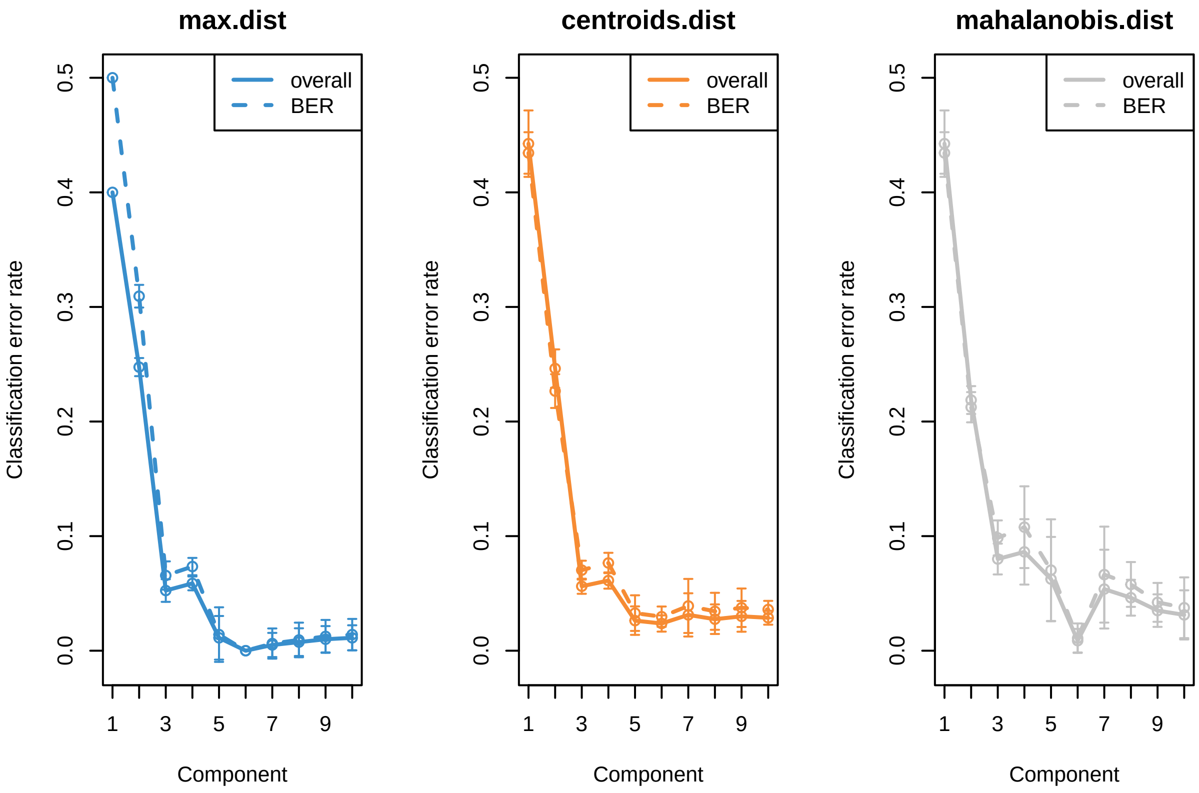


```
plotIndiv(diverse.plsda , comp = c(1,2), ind.names = FALSE,
          ellipse = TRUE, legend = TRUE, title = 'HMP Most Diverse, PLSDA comp 1 - 2')
```

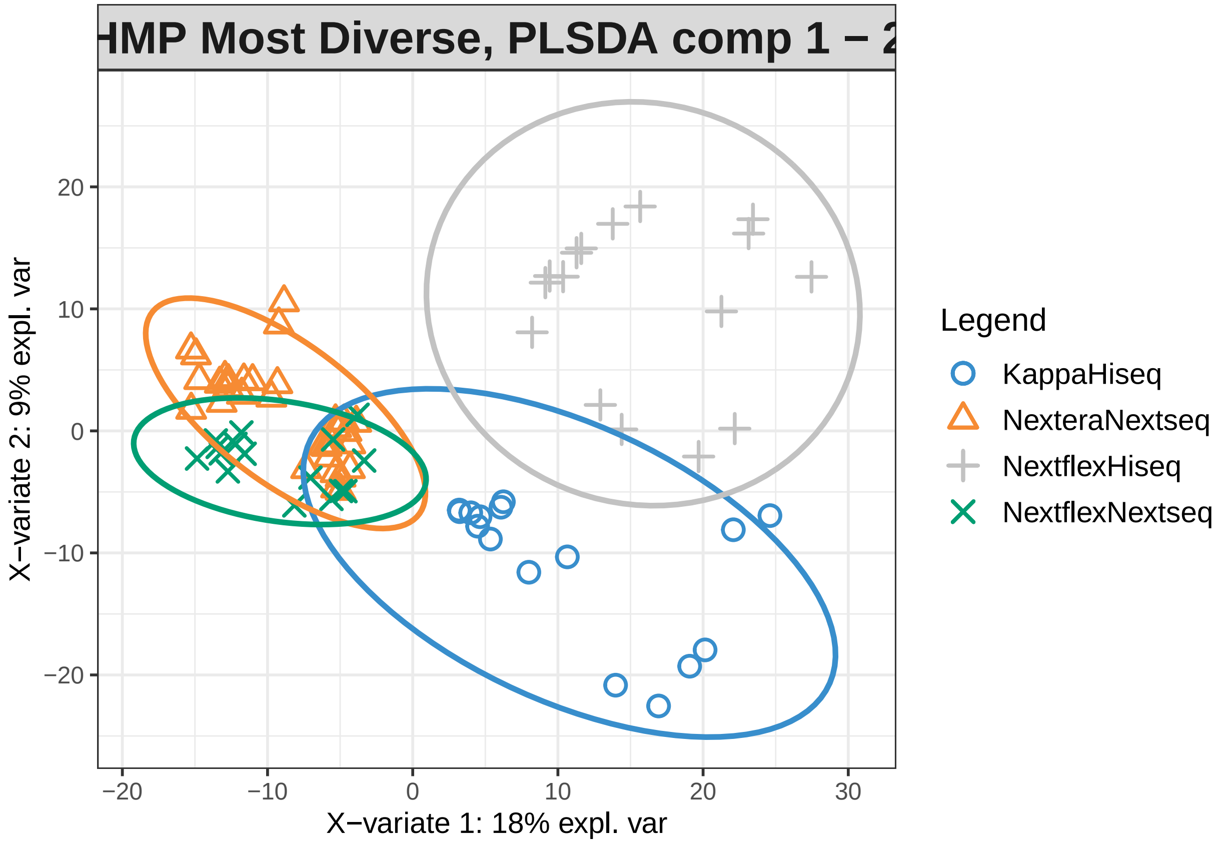


```
plotIndiv(diverse.plsda, comp = c(1,3), ind.names = FALSE,
          ellipse = TRUE, legend = TRUE, title = 'HMP Most Diverse, PLSDA comp 1 - 3')
```

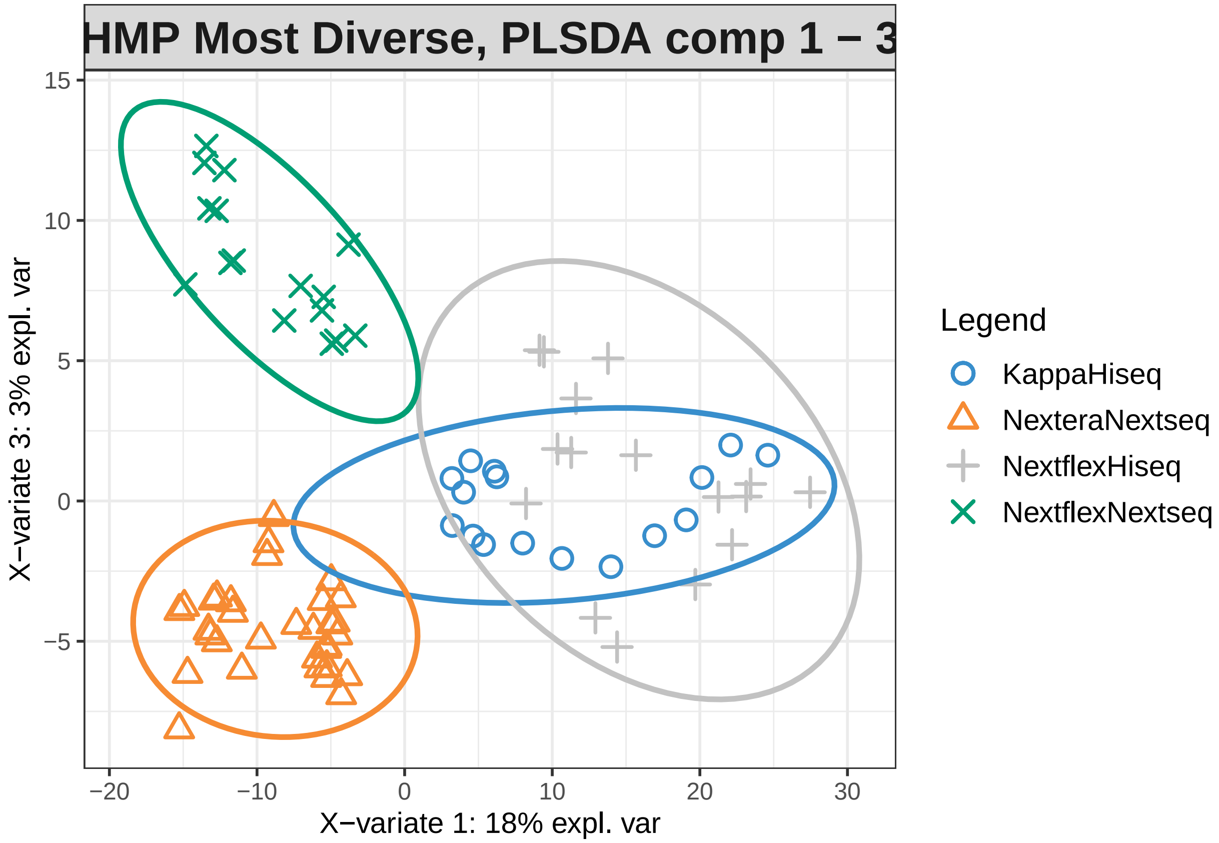


```
  #The third component seem to add info
set.seed(33)  # for reproducible results for this code
diverse.tune.splsda = tune.splsda(Tax2, Y, ncomp = 3,
                           logratio = 'CLR',
                            multilevel = sample,
                           test.keepX = c(seq(5,150, 5)), validation = 'Mfold',
                           folds = 5, dist = 'max.dist', nrepeat = 10,
                           progressBar = FALSE)
#plot(diverse.tune.splsda)

# optimal number of variables to select on 3 comps:
select.keepX = diverse.tune.splsda$choice.keepX[1:3]
select.keepX
```

```
## comp1 comp2 comp3
##     5    45     5
```

```
# select.keepX = c(50, 15, 40) # to manually choose size of selection

diverse.splsda = splsda(Tax2, Y, ncomp = 10, logratio = 'CLR', multilevel = sample,
                        keepX = select.keepX)
plotIndiv(diverse.splsda, comp = c(1,2),
          ind.names = FALSE,
          ellipse = TRUE, legend = TRUE,
          title = 'HMP Most Diverse, sPLSDA comp 1 - 2')
```

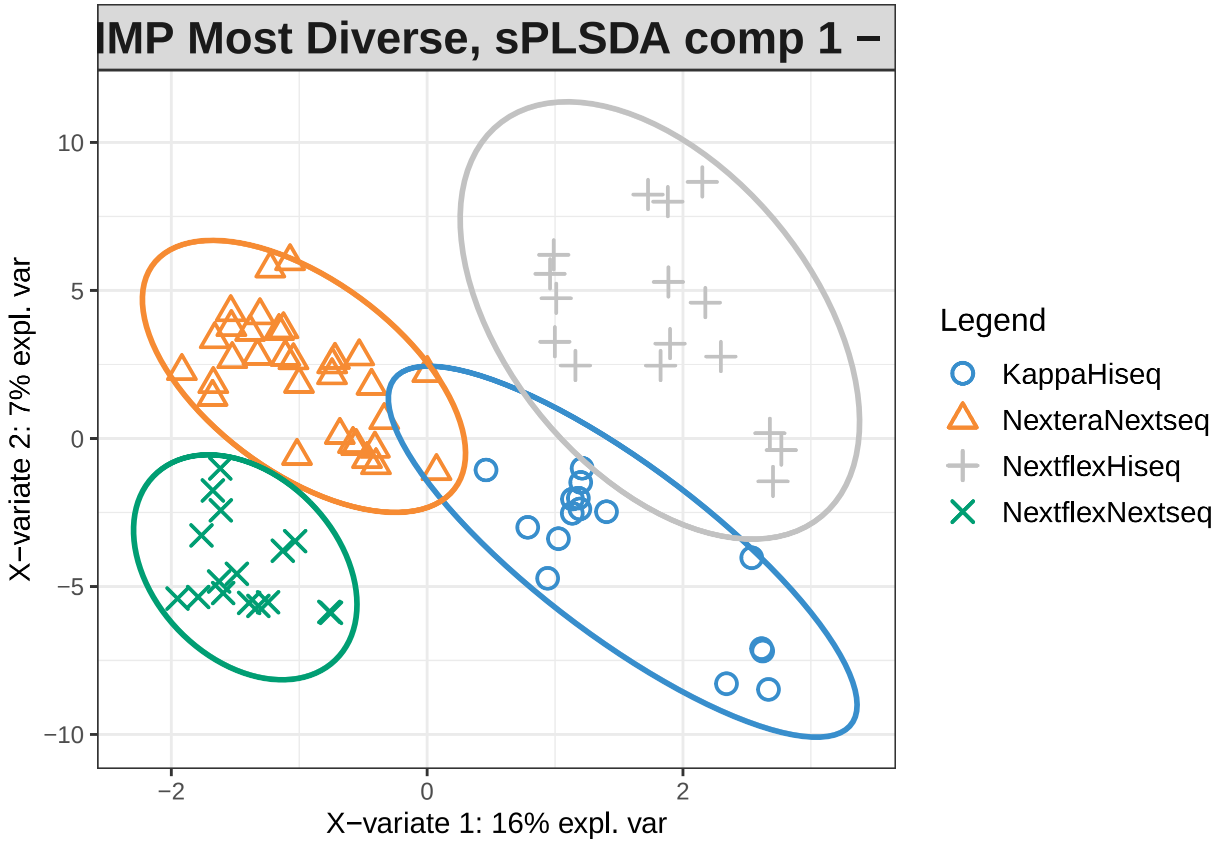


```r
  #changed ncomp to 10 to see in plot(diverse.perf.splda)

plotIndiv(diverse.splsda, comp = c(1,3),
          ind.names = FALSE,
          ellipse = TRUE, legend = TRUE,
          title = 'HMP Most Diverse, sPLSDA comp 1 - 3')
```

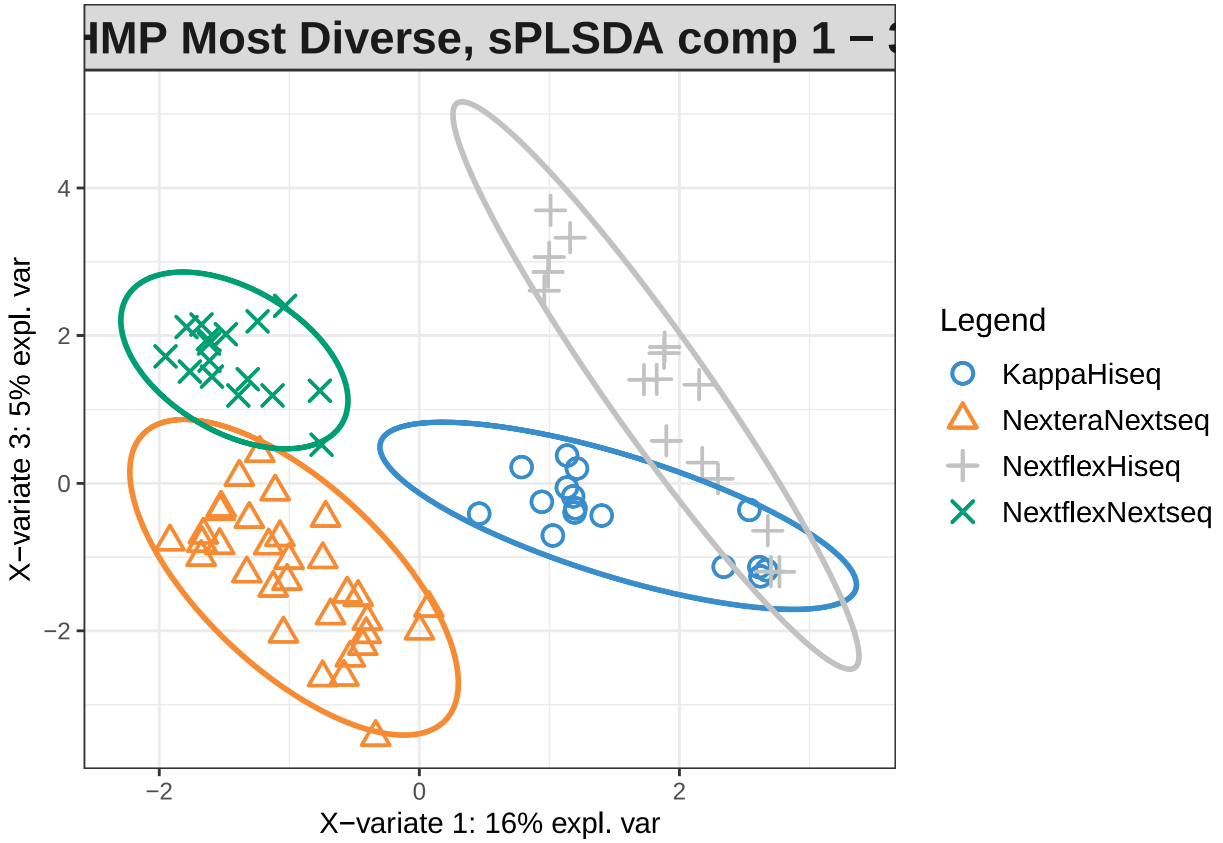


```
set.seed(34)  # for reproducible results for this code
diverse.perf.splsda = perf(diverse.splsda, validation = 'Mfold', folds = 5,
                   progressBar = FALSE, nrepeat = 10, dist = 'max.dist')
diverse.perf.splsda$error.rate
```

```
## $overall
##         max.dist
## comp1   0.41125
## comp2   0.11625
## comp3   0.05250
## comp4   0.03500
## comp5   0.00875
## comp6   0.01250
## comp7   0.01750
## comp8   0.01750
## comp9   0.01625
## comp10  0.00750
##
## $BER
##          max.dist
## comp1  0.5140625
## comp2  0.1445312
## comp3  0.0656250
## comp4  0.0437500
## comp5  0.0109375
## comp6  0.0156250
```

```
## comp7  0.0218750
## comp8  0.0218750
## comp9  0.0203125
## comp10 0.0093750
```

```r
#plot(diverse.perf.splsda)

head(selectVar(diverse.splsda, comp = 1)$value)
```

```
##                     value.var
## Peptoanaerobacter -0.87114556
## Polaribacter      -0.35319962
## Methanosaeta       0.33524745
## Porphyromonas     -0.06022969
## Tenacibaculum     -0.01835695
```

```r
selected.OTU.comp1 = selectVar(diverse.splsda, comp = 1)$name
# stability of OTUs selected on comp 1
diverse.perf.splsda$features$stable[[1]][selected.OTU.comp1]
```

```
##
## Peptoanaerobacter      Polaribacter      Methanosaeta     Porphyromonas
##              1.00              0.76              0.82              0.48
##     Tenacibaculum
##              0.40
```

```r
kable(data.frame(diverse.perf.splsda$features$stable[[1]][selected.OTU.comp1]), caption = '
Stability of OTUs selected on comp 1, ranked by decreasing importance in sPLS-DA.')
```

Table 1: Stability of OTUs selected on comp 1, ranked by decreasing importance in sPLS-DA.

Var1	Freq
Peptoanaerobacter	1.00
Polaribacter	0.76
Methanosaeta	0.82
Porphyromonas	0.48
Tenacibaculum	0.40

```r
selected.OTU.comp2 = selectVar(diverse.splsda, comp = 2)$name
# stability of OTUs selected on comp 2
diverse.perf.splsda$features$stable[[2]][selected.OTU.comp2]
```

```
##
##                Prevotella                Bacteroides               Mediterranea
##                      1.00                       0.88                       0.88
##               Barnesiella        Prevotellamassilia          Ornithobacterium
##                      0.86                       0.98                       0.94
##          Peptoclostridium                 Petrimonas               Odoribacter
##                      0.96                       0.86                       0.86
##           Parabacteroides              Cloacibacillus        Ruminiclostridium
##                      0.98                       1.00                       1.00
##               Veillonella            Chryseobacterium                   Unknown
##                      1.00                       0.86                       1.00
##           Catenibacterium               Succinivibrio               Allisonella
```

```
##                        1.00                      0.82                      1.00
##               Angelakisella    Phascolarctobacterium        Methylobacterium
##                        1.00                      1.00                      1.00
##                 Acetivibrio             Desulfovibrio               Lelliottia
##                        1.00                      0.80                      0.84
##                   Alistipes              Amphibacillus       Lachnoclostridium
##                        0.74                      0.62                      0.84
##                   Kp15virus              Ruminococcus             Byssochlamys
##                        0.84                      0.74                      0.60
##               Streptococcus            Intestinimonas               Myxococcus
##                        0.78                      0.60                      0.66
##               Corallococcus                  Bacillus              Eubacterium
##                        0.52                      0.60                      0.64
##                Cellulomonas             Capnocytophaga               Holdemania
##                        0.48                      0.36                      0.54
##                Serinicoccus         Sanguibacteroides            Lecanicillium
##                        0.44                      0.38                      0.40
## Candidatus Azobacteroides      Massilioclostridium                Hespellia
##                        0.38                      0.60                      0.58
```

```
kable(data.frame(diverse.perf.splsda$features$stable[[2]][selected.OTU.comp2]), caption = '
Stability of OTUs selected on comp 2, ranked by decreasing importance in sPLS-DA.')
```

Table 2: Stability of OTUs selected on comp 2, ranked by decreasing importance in sPLS-DA.

Var1	Freq
Prevotella	1.00
Bacteroides	0.88
Mediterranea	0.88
Barnesiella	0.86
Prevotellamassilia	0.98
Ornithobacterium	0.94
Peptoclostridium	0.96
Petrimonas	0.86
Odoribacter	0.86
Parabacteroides	0.98
Cloacibacillus	1.00
Ruminiclostridium	1.00
Veillonella	1.00
Chryseobacterium	0.86
Unknown	1.00
Catenibacterium	1.00
Succinivibrio	0.82
Allisonella	1.00
Angelakisella	1.00
Phascolarctobacterium	1.00
Methylobacterium	1.00
Acetivibrio	1.00
Desulfovibrio	0.80
Lelliottia	0.84
Alistipes	0.74
Amphibacillus	0.62
Lachnoclostridium	0.84

Var1	Freq
Kp15virus	0.84
Ruminococcus	0.74
Byssochlamys	0.60
Streptococcus	0.78
Intestinimonas	0.60
Myxococcus	0.66
Corallococcus	0.52
Bacillus	0.60
Eubacterium	0.64
Cellulomonas	0.48
Capnocytophaga	0.36
Holdemania	0.54
Serinicoccus	0.44
Sanguibacteroides	0.38
Lecanicillium	0.40
Candidatus Azobacteroides	0.38
Massilioclostridium	0.60
Hespellia	0.58

```
selected.OTU.comp3 = selectVar(diverse.splsda, comp = 3)$name
# stability of OTUs selected on comp 3
diverse.perf.splsda$features$stable[[3]][selected.OTU.comp3]
```

```
##
##       Romboutsia          Blautia     Holdemanella Fusicatenibacter
##             0.92             0.82             0.68             0.56
##  Intestinibacter
##             0.48
```

```
kable(data.frame(diverse.perf.splsda$features$stable[[3]][selected.OTU.comp3]), caption = '
Stability of OTUs selected on comp 3, ranked by decreasing importance in sPLS-DA.')
```

Table 3: Stability of OTUs selected on comp 3, ranked by decreasing importance in sPLS-DA.

Var1	Freq
Romboutsia	0.92
Blautia	0.82
Holdemanella	0.68
Fusicatenibacter	0.56
Intestinibacter	0.48

```
plotLoadings(diverse.splsda, comp = 1, method = 'mean', contrib = 'max',
             size.title = 1)
```

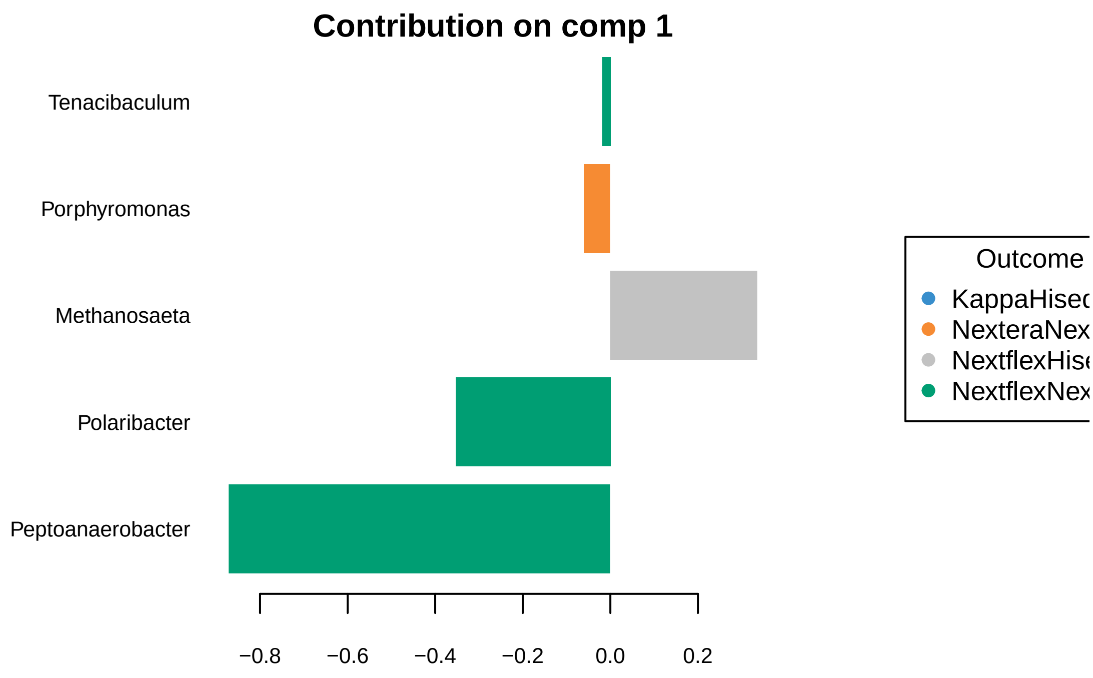


```
plotLoadings(diverse.splsda, comp = 2, method = 'mean', contrib = 'max',
             size.title = 1)
```

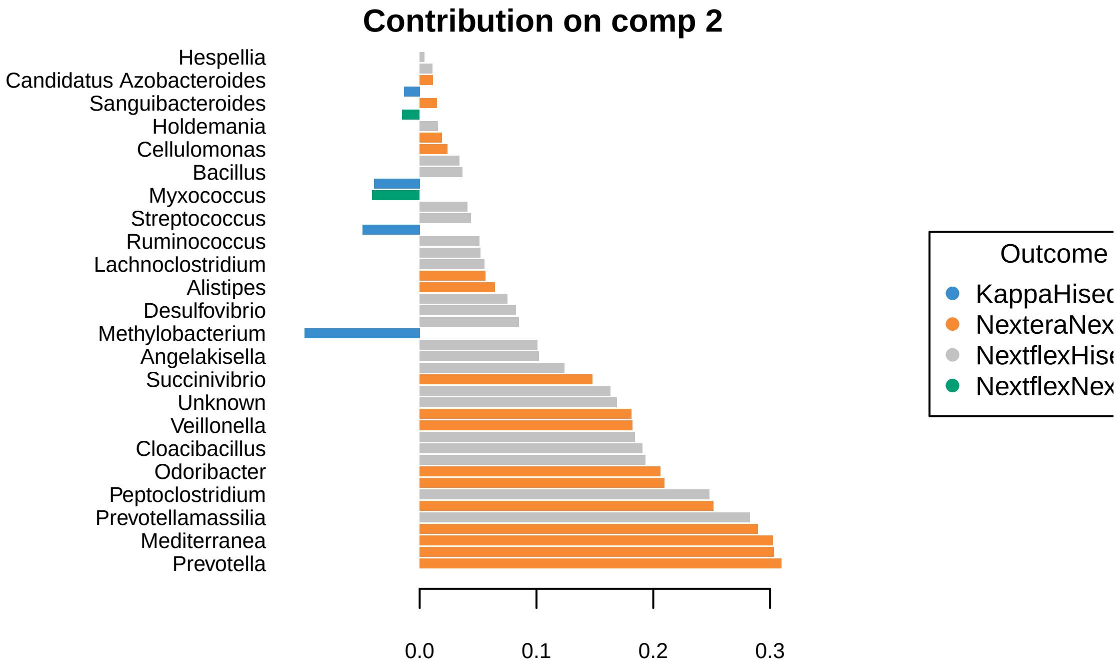


```r
plotLoadings(diverse.splsda, comp = 3, method = 'mean', contrib = 'max',
             size.title = 1)
```

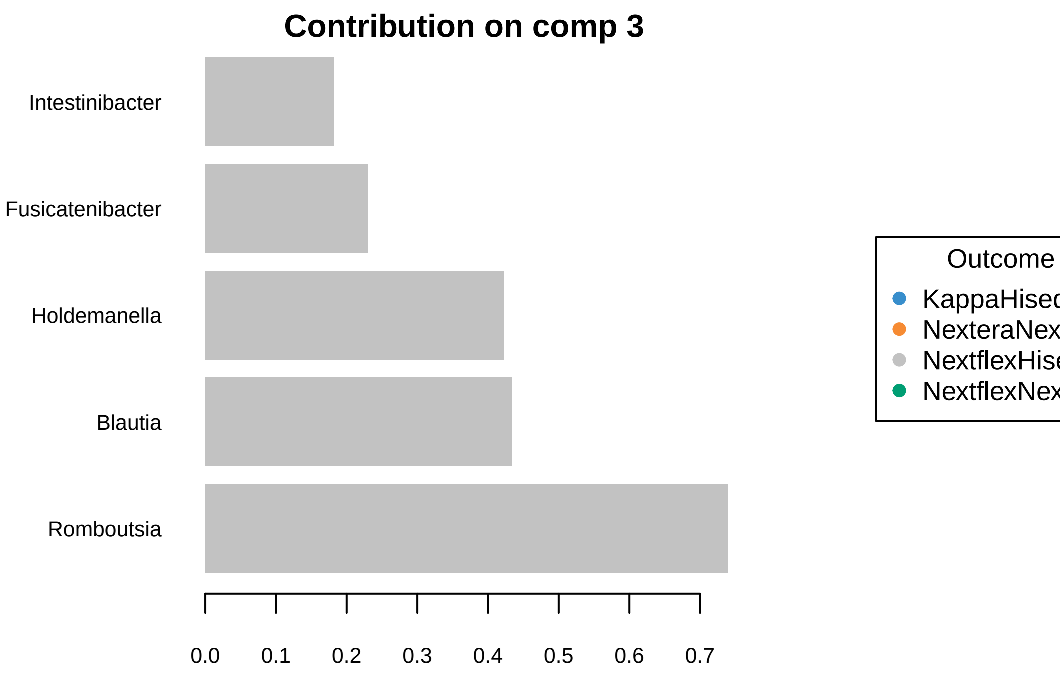


```
##Extracting barplots manually Source code
  #https://github.com/cran/mixOmics/blob/master/R/plotLoadings.splsda.R
comp1 <- plotLoadings(diverse.splsda, comp = 1, method = 'mean', contrib = 'max',
             size.title = 1)
```

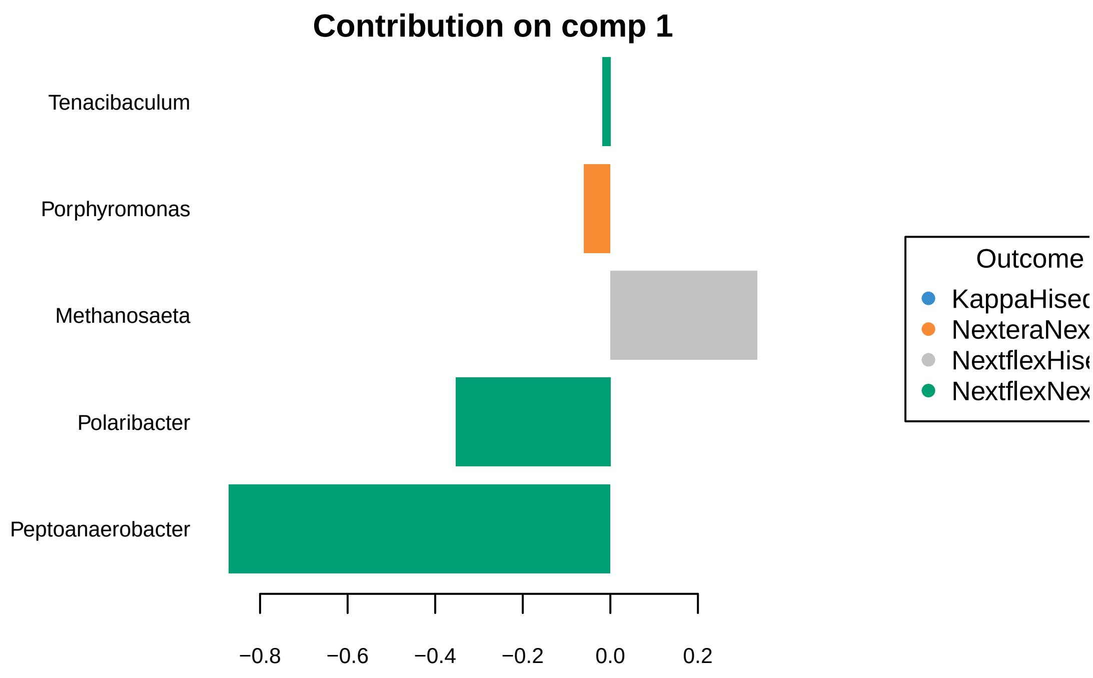


```
comp2 <- plotLoadings(diverse.splsda, comp = 2, method = 'mean', contrib = 'max',
             size.title = 1)
```

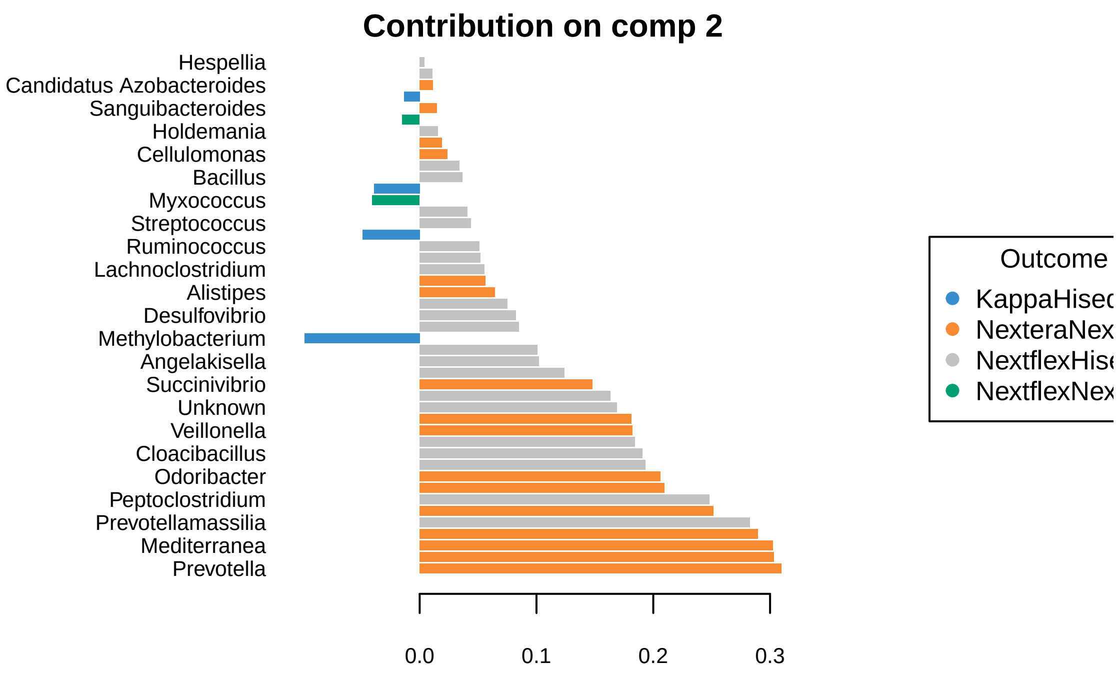


```
comp3 <- plotLoadings(diverse.splsda, comp = 3, method = 'mean', contrib = 'max',
             size.title = 1)
```

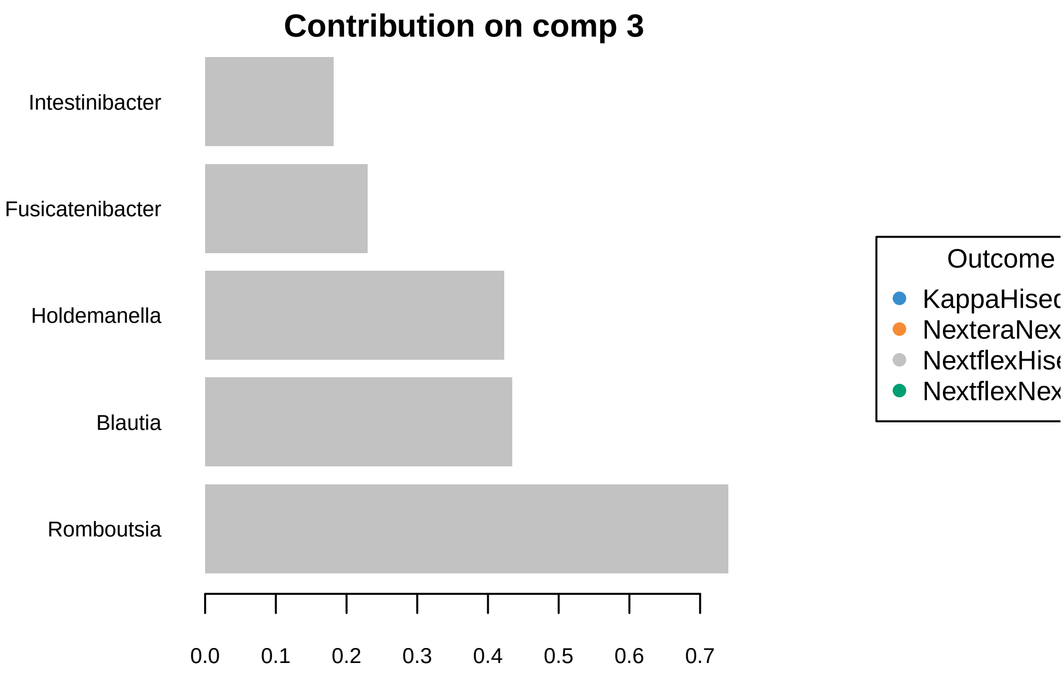


```
#Create barplots with ggplot. Can not get pattern across fill unless using a hacky version
  #(Hadley Wickan writes: It's not currently possible because grid (the graphics system
  #that ggplot2 uses to do the actual drawing) doesn't support textures. Sorry!
#https://stackoverflow.com/questions/2895319/how-to-add-texture-to-fill-colors-in-ggplot2).
#Ended using base R barplot, because I could not get colors to match the entries.
comp1$genera <- rownames(comp1)
comp1$genera <- factor(comp1$genera, levels=rownames(comp1))
#Create column org grouping for colouring
comp1$color2 <- ifelse(comp1$GroupContrib=="NextflexNextseq", "#377eb8",
          ifelse(comp1$GroupContrib=="NextflexHiseq", "#4daf4a",
          ifelse(comp1$GroupContrib=="NexteraNextseq", "#ff7f00",
          ifelse(comp1$GroupContrib=="KappaHiseq", "#984ea3",
                 "Other"))))
#ggplot(data=comp1, aes(x=genera, y=importance)) +
#  geom_bar(stat="identity", fill=comp1$color2) +
#  coord_flip()

comp2$genera <- rownames(comp2)
comp2$genera <- factor(comp2$genera, levels=rownames(comp1))
#Create column org grouping for colouring
comp2$color2 <- ifelse(comp2$GroupContrib=="NextflexNextseq", "#377eb8",
          ifelse(comp2$GroupContrib=="NextflexHiseq", "#4daf4a",
          ifelse(comp2$GroupContrib=="NexteraNextseq", "#ff7f00",
          ifelse(comp2$GroupContrib=="KappaHiseq", "#984ea3",
                 "Other"))))
```

```
comp3$genera <- rownames(comp3)
comp3$genera <- factor(comp3$genera, levels=rownames(comp1))
#Create column org grouping for colouring
comp3$color2 <- ifelse(comp3$GroupContrib=="NextflexNextseq", "#377eb8",
          ifelse(comp3$GroupContrib=="NextflexHiseq", "#4daf4a",
          ifelse(comp3$GroupContrib=="NexteraNextseq", "#ff7f00",
          ifelse(comp3$GroupContrib=="KappaHiseq", "#984ea3",
                 "Other"))))

pdf("S4_Fig_sPLS-DA.pdf", height=10, width=25)
layout(matrix(c(1,2, 3,2), nrow=2, ncol=2, byrow=TRUE))
par(mar=c(4,20,4,2))
barplot(comp1$importance, horiz = T, las = 1, col = comp1$color2, axisnames = TRUE,
        beside = TRUE, names.arg=rownames(comp1))

barplot(comp2$importance, horiz = T, las = 1, col = comp2$color2, axisnames = TRUE,
        beside = TRUE, names.arg=rownames(comp2))

barplot(comp3$importance, horiz = T, las = 1, col = comp3$color2, axisnames = TRUE,
        beside = TRUE, names.arg=rownames(comp3))
dev.off()
```

```
## pdf
##   2
```

Multivariate ANOVA based on dissimilarities (Adonis)

Statistical output is presented in Table1 and S2_Table

```
rm(list=setdiff(ls(), c("Metadata", "Feature", "Tax")))

#Subset data pig feces 1 and 2 (P1, P2), Sewage 1 and 2 (S1, S2), or spiked unspiked
Subset<- c("Allunspiked", "PFunspiked", "SWunspiked", "P1unspiked", "P2unspiked",
           "S1unspiked", "S2unspiked")
#i <- c("Allunspiked") #validation purposes
#c("P1spiked", "P1unspiked", "P2spiked", "P2unspiked", "S1spiked", "S1unspiked",
  #S2spiked", "S2unspiked")
#All, Allspiked, Allunspiked, PF, PFspiked, PFunspiked, SW, SWspiked, SWunspiked,
  #P1, P1spiked, P1unspiked, P2, P2spiked, P2unspiked, S1, S1spiked, S1unspiked, S2,
  #S2spiked, S2unspiked

#Subset experiment, Experiment_Type. (Meaningfull combinations of subset and SubExp:
  #HX=All, FTX=P1&S1, LTX=P1&S1, LPSX=All unspiked, HXFTX=P1&S1, HXLTX=P1&S1,
  #HXLPSX=All unspiked, HXLTXLPSX=P1&S1 unspiked)
SubExp<-"LPSX" #HX, FTX, LTX, LPSX, HXFTX, HXLTX, HXLPSX, HXLTXLPSX, HXFTXLTX, All

#Subset Frozen Unfrozen
SubFre<-"Both" #Frozen, Unfrozen, Both

#Creating tables for all individual tests. Additional overview of p-values created.
  #To see evaluation plots remove hashtags. Using for loop to go through Subset vector

#Make empty dataframe to add p-values
dfp<-data.frame()
```

```r
dfp<-data.frame(Test=c("adonis", "betadisperAnova", "betadisperPermutest"))
dfp2<-data.frame()
dfp2<-data.frame(Test=c("adonisExperiment", "adonisStorage", "adonisLibprep",
                        "adonisSeqplat"))

for (i in Subset) {
#Removing negative and positive controls
Metadata2<-filter(Metadata, Sample_type_simple=="Sample")

#Subsetting Metadata2
if (i=="Allspiked") {
  Metadata2<-filter(Metadata2, SpikedUnspiked == "Spiked")
} else if (i=="Allunspiked") {
  Metadata2<-filter(Metadata2, SpikedUnspiked == "Unspiked")
} else if (i=="PF") {
  Metadata2<-filter(Metadata2, Experiment == "Pig_feces_1" | Experiment == "Pig_feces_2")
} else if (i=="PFspiked") {
  Metadata2<-filter(Metadata2, Sample_type == "Pig_feces_1_spiked" |
                    Sample_type == "Pig_feces_2_spiked")
} else if (i=="PFunspiked") {
  Metadata2<-filter(Metadata2, Sample_type == "Pig_feces_1" |
                    Sample_type == "Pig_feces_2")
} else if (i=="SW") {
  Metadata2<-filter(Metadata2, Experiment == "Sewage_1" | Experiment == "Sewage_2")
} else if (i=="SWspiked") {
  Metadata2<-filter(Metadata2, Sample_type == "Sewage_1_spiked" |
                    Sample_type == "Sewage_2_spiked")
} else if (i=="SWunspiked") {
  Metadata2<-filter(Metadata2, Sample_type == "Sewage_1" | Sample_type == "Sewage_2")
} else if (i=="P1") {
  Metadata2<-filter(Metadata2, Experiment == "Pig_feces_1")
} else if (i=="P1spiked") {
  Metadata2<-filter(Metadata2, Sample_type == "Pig_feces_1_spiked")
} else if (i=="P1unspiked") {
  Metadata2<-filter(Metadata2, Sample_type == "Pig_feces_1")
} else if (i=="P2") {
  Metadata2<-filter(Metadata2, Experiment == "Pig_feces_2")
} else if (i=="P2spiked") {
  Metadata2<-filter(Metadata2, Sample_type == "Pig_feces_2_spiked")
} else if (i=="P2unspiked") {
  Metadata2<-filter(Metadata2, Sample_type == "Pig_feces_2")
} else if (i=="S1") {
  Metadata2<-filter(Metadata2, Experiment == "Sewage_1")
} else if (i=="S1spiked") {
  Metadata2<-filter(Metadata2, Sample_type == "Sewage_1_spiked")
} else if (i=="S1unspiked") {
  Metadata2<-filter(Metadata2, Sample_type == "Sewage_1")
} else if (i=="S2") {
  Metadata2<-filter(Metadata2, Experiment == "Sewage_2")
} else if (i=="S2spiked") {
  Metadata2<-filter(Metadata2, Sample_type == "Sewage_2_spiked")
} else if (i=="S2unspiked") {
  Metadata2<-filter(Metadata2, Sample_type == "Sewage_2")
```

```r
} else if (i=="All") {
  print("No subsetting, all included")
} else {
  print("Subset defined not valid")
}

#Further subsetting Metadata2
if (SubExp=="HX") {
  Metadata2<-filter(Metadata2, Experiment_type == "Handling_experiment")
} else if (SubExp=="FTX") {
  Metadata2<-filter(Metadata2, Experiment_type == "Freeze_thaw_experiment")
} else if (SubExp=="LTX") {
  Metadata2<-filter(Metadata2, Experiment_type == "Long_term_storage_experiment")
} else if (SubExp=="LPSX") {
  Metadata22<-filter(Metadata2, Experiment_type == "Library_prep_seq_platform_experiment")
  VectorLPSX <- unique(Metadata22$Matching_samples)
  Metadata2<-filter(Metadata2, Matching_samples %in% VectorLPSX)
  rm(VectorLPSX, Metadata22)
} else if (SubExp=="HXFTX") {
  Metadata2<-filter(Metadata2, Experiment_type == "Handling_experiment" |
                      Experiment_type == "Freeze_thaw_experiment")
} else if (SubExp=="HXLTX") {
  Metadata2<-filter(Metadata2, Experiment_type == "Handling_experiment" |
                      Experiment_type == "Long_term_storage_experiment")
} else if (SubExp=="HXLPSX") {
  Metadata2<-filter(Metadata2, Experiment_type == "Handling_experiment" |
                      Experiment_type == "Library_prep_seq_platform_experiment")
} else if (SubExp=="HXLTXLPSX") {
  Metadata2<-filter(Metadata2, Experiment_type == "Handling_experiment" |
                      Experiment_type == "Long_term_storage_experiment" |
                      Experiment_type == "Library_prep_seq_platform_experiment")
} else if (SubExp=="HXFTXLTX") {
  Metadata2<-filter(Metadata2, Experiment_type == "Handling_experiment" |
                      Experiment_type == "Freeze_thaw_experiment" |
                      Experiment_type == "Long_term_storage_experiment")
} else if (SubExp=="All") {
  print("No subsetting, all included")
} else {
  print("Subset defined not valid")
}

#Further subsetting Metadata2
if (SubFre=="Frozen") {
  Metadata2<-filter(Metadata2, FrozenUnfrozenSimple == "Freezer")
} else if (SubFre=="Unfrozen") {
  Metadata2<-filter(Metadata2, FrozenUnfrozenSimple == "Unfrozen")
} else if (SubFre=="Both") {
  print("No subsetting, all included")
} else {
  print("Subset defined not valid")
}

#Removing the Kappa NextSeq run
```

```r
Metadata2<-Metadata2[grep("\\KANS.*", Metadata2$Sample, invert=TRUE),]

#Applying subsetting to OTU tables
Tax2<-dplyr::select(Tax, one_of(Metadata2$Sample))

#filtering of the Counttable depending on rowSums.
Tax2 <- Tax2[rowSums(Tax2)>0,] #Removing all rows that only contains zeroes
Tax2 <- Tax2[rowSums(Tax2)>(5*ncol(Tax2)),] #Removing all rows(Species) that is below an
  #average count of 5
# replace 0 values with an estimate using simple multiplicative replacement
Tax2 <- t(cmultRepl(t(Tax2), method="CZM", label=0))
#Maks TSS
Tax2<-as.matrix(t(sweep(Tax2, 2, colSums(Tax2), FUN="/"))) #Transpose to have genera as
  #columns and samples as rows

#Make column that combine lib prep and seq platform
Metadata2$LPSP<-paste(Metadata2$Library_preparation,
                      Metadata2$Sequencing_platform, sep="_") #Be aware that
  #Nextera 1 & 2 are analyzed as one group,
  #you could argue that it should be done seperately

#Calculate sample-distance matrix
distmatrix <- vegdist(ilr(Tax2), method="euclidean")

#Which factor to test can pick Metadata2 columns consider including both library
  #preparation and sequencing platform individually in the model
Test_Factor <- "LPSP" #LPSP, Library_preparation, Sequencing_platform
#Test_Factor <- "Library_preparation + Sequencing_platform" #Does not work with betadisper
#Create design formula
design <- formula(paste("distmatrix"," ~ ", Test_Factor, sep=""))
if (i=="Allunspiked" | i=="PFunspiked" | i=="SWunspiked") {
  design2 <- formula(paste("distmatrix"," ~ ", "Experiment+", "Sequencing_platform+",
                           "StoragePlacementType+", "Library_preparation" , sep=""))
  print(design2)
} else {
  design2 <- formula(paste("distmatrix"," ~ ", "Sequencing_platform+",
                           "StoragePlacementType+", "Library_preparation", sep=""))
  print(design2)
}
#Have also tried to change order of model:
#"Experiment+", "StoragePlacementType+", "Library_preparation+", "Sequencing_platform"
#"Experiment+", "Library_preparation+", "StoragePlacementType+", "Sequencing_platform"
#"Experiment+", "Sequencing_platform+", "Library_preparation+", "StoragePlacementType"
#"Experiment+", "Library_preparation+", "Sequencing_platform+", "StoragePlacementType"
#"Experiment+", "Sequencing_platform+", "StoragePlacementType+", "Library_preparation"
#"Experiment+", "StoragePlacementType+", "Sequencing_platform+", "Library_preparation"
#Could also have changed the first variable Experiment ending with many combinations
#I'm getting the same results with "Experiment+", "StoragePlacementType+",
  #"Library_preparation+", "Sequencing_platform"
#and "Experiment+", "Library_preparation+", "StoragePlacementType+",
  #"Sequencing_platform", but not with
#"Experiment+", "StoragePlacementType+", "Sequencing_platform+", "Library_preparation"
#Have no idea why. I'm changing the setup to by="margin".
```

```
#design2 <- formula(paste("distmatrix"," ~ ", "Experiment*", "StoragePlacementType*",
  #"Library_preparation*", "Sequencing_platform" , sep=""))
#adonis can handle both continous and factor predictors
set.seed(999)
adonisObject<-adonis2(design, Metadata2, by="margin", perm=99999) #, perm=999
  #can increase to get exact p-values
set.seed(999)
adonisObject2<-adonis2(design2, Metadata2, by="margin", perm=99999)
#adonisObject #If significant then difference between groups
#Sys.sleep(1)
write.table(data.frame(adonisObject), file=paste(i, "Adonis", "_", Test_Factor, ".txt",
                                                 sep=""),
            sep="\t", dec=".", row.names = T, quote = F)
write.table(data.frame(adonisObject2), file=paste("All", i, "Adonis", "_", ".txt",
                                                  sep=""),
            sep="\t", dec=".", row.names = T, quote = F)

#### Evaluating the model assumptions
TestModel <- with(Metadata2, betadisper(distmatrix, LPSP)) #Also have to change here LPSP,
  #Library_preparation, Sequencing_platform. Can not run betadisper with multiple
  #independant variables
#TestModel
#plot(TestModel)
plot(TestModel, label=FALSE)
#boxplot(TestModel)
#anova(TestModel)
anovaTestModel<-anova(TestModel) #p>0.05 -> Assumption met
write.table(data.frame(anova(TestModel)), file=paste(i, "Betadisper", "_", Test_Factor,
                                                     ".txt", sep=""),
            sep="\t", dec=".", row.names = T, quote = F)
set.seed(999)
#permutest(TestModel)
permy<-permutest(TestModel)
#data.frame(permy$tab)
write.table(data.frame(permy$tab), file=paste(i, "Permutest", "_", Test_Factor, ".txt",
                                              sep=""),
            sep="\t", dec=".", row.names = T, quote = F)
#p>0.05 -> Assumption met

TukeyHSD(TestModel, perm=999) #Increasing perm does not change p adj
dfp[paste(i)]<-c(adonisObject$`Pr(>F)`[1], anovaTestModel$`Pr(>F)`[1],
                 permy$tab$`Pr(>F)`[1])
dfp2[paste(i)]<-c(adonisObject2$`Pr(>F)`[1])

if (i=="Allunspiked" | i=="PFunspiked" | i=="SWunspiked") {
  dfp2[paste(i)]<-c(adonisObject2$`Pr(>F)`[1], adonisObject2$`Pr(>F)`[2],
                    adonisObject2$`Pr(>F)`[3], adonisObject2$`Pr(>F)`[4])
} else {
  dfp2[paste(i)]<-c("Na", adonisObject2$`Pr(>F)`[1], adonisObject2$`Pr(>F)`[2],
                    adonisObject2$`Pr(>F)`[3])
}

}
```

```
## [1] "No subsetting, all included"
## No. corrected values:  4863
## distmatrix ~ Experiment + Sequencing_platform + StoragePlacementType +
##     Library_preparation
```

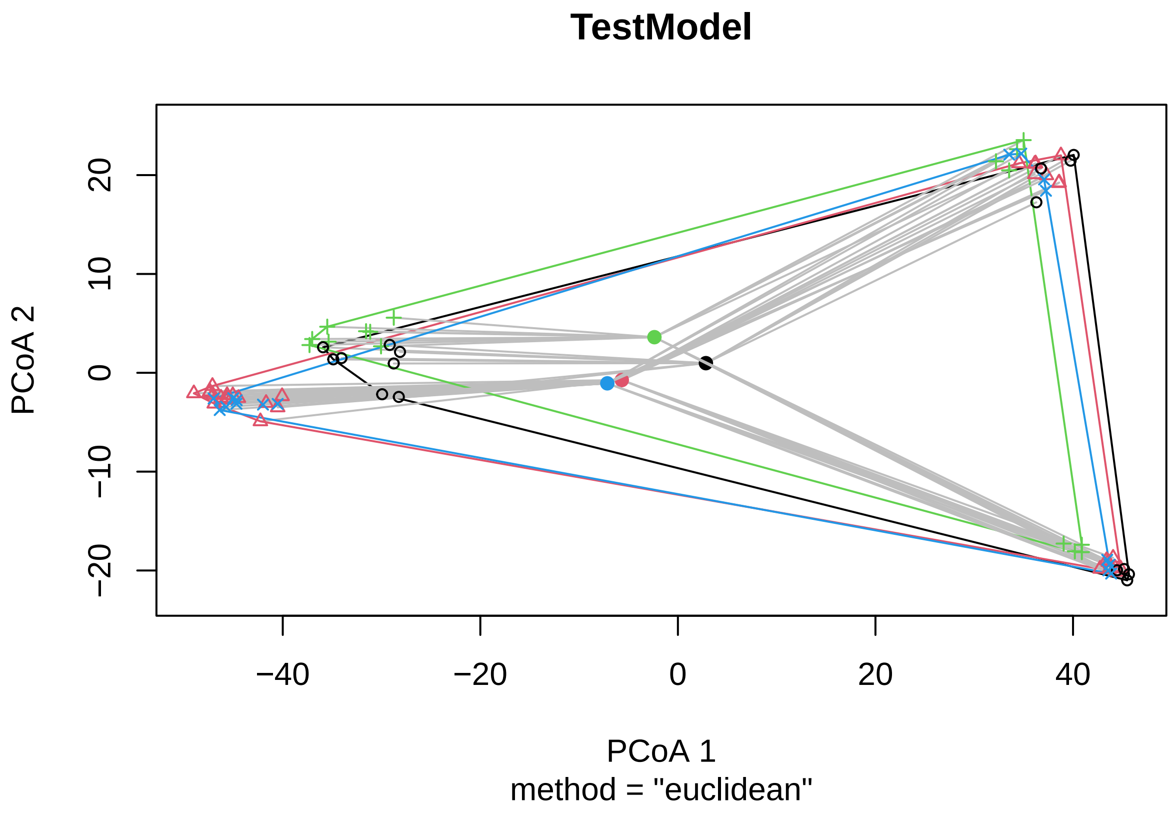


```
## [1] "No subsetting, all included"
## No. corrected values:  21
## distmatrix ~ Experiment + Sequencing_platform + StoragePlacementType +
##     Library_preparation
```

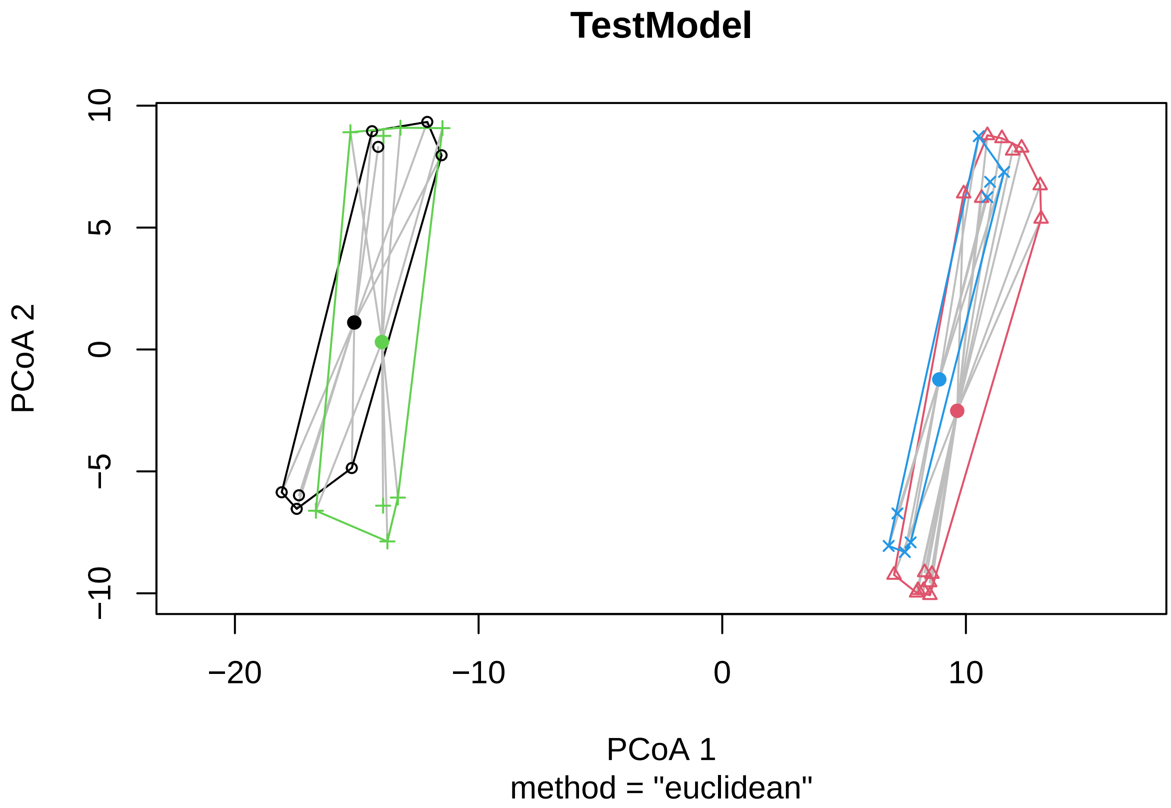


```
## [1] "No subsetting, all included"
## No. corrected values:  140
## distmatrix ~ Experiment + Sequencing_platform + StoragePlacementType +
##     Library_preparation
```

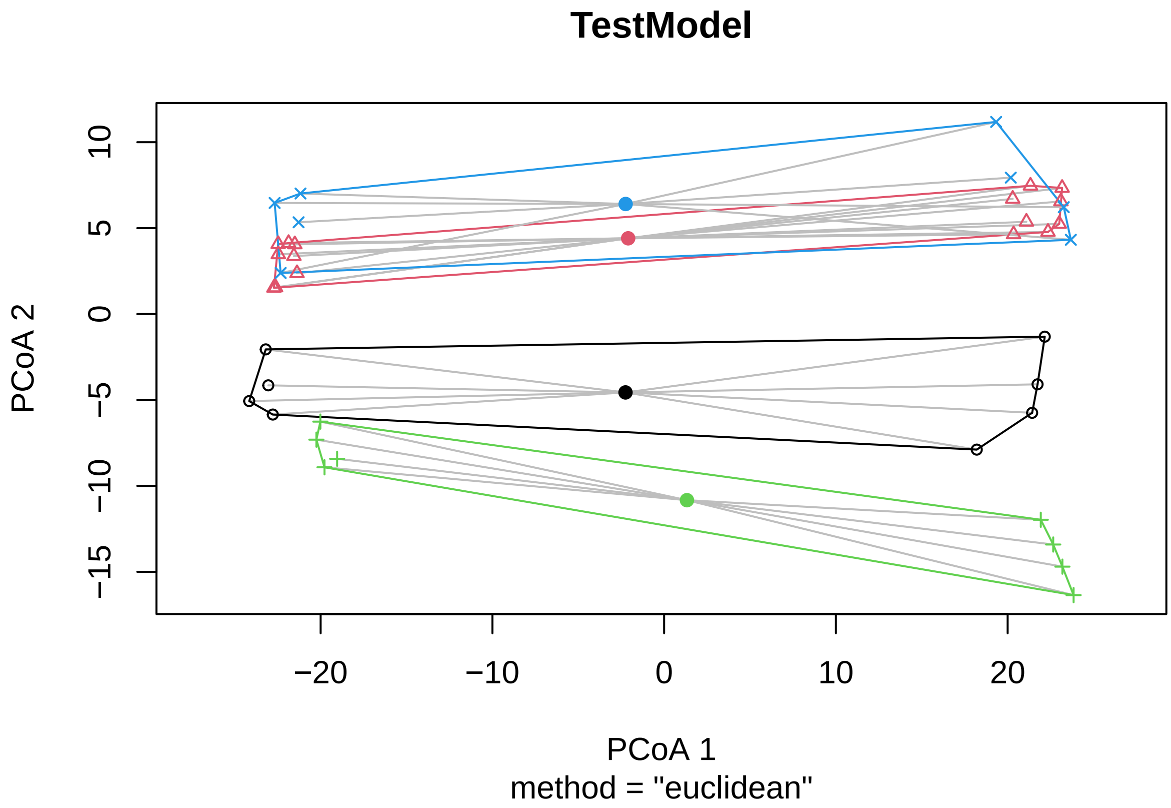


```
## [1] "No subsetting, all included"
## No. corrected values:  3
## distmatrix ~ Sequencing_platform + StoragePlacementType + Library_preparation
```

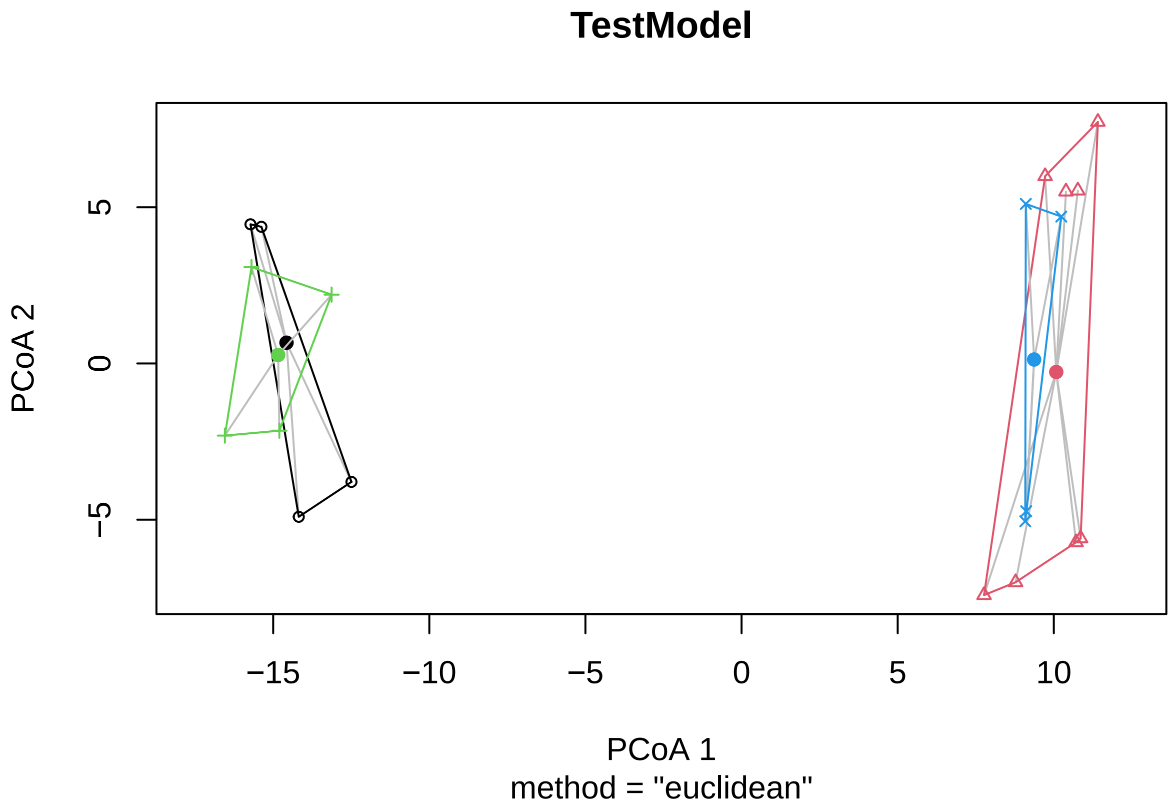


```
## [1] "No subsetting, all included"
## distmatrix ~ Sequencing_platform + StoragePlacementType + Library_preparation
```

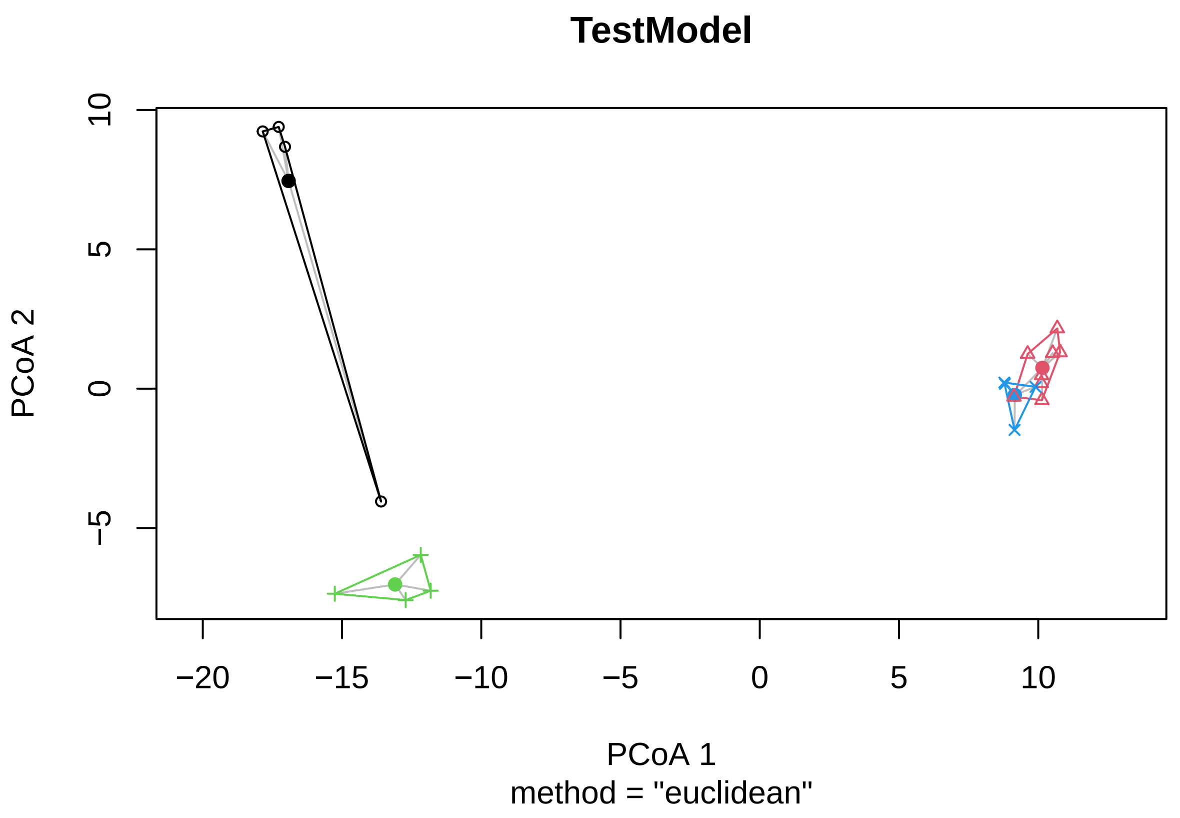


```
## [1] "No subsetting, all included"
## distmatrix ~ Sequencing_platform + StoragePlacementType + Library_preparation
```

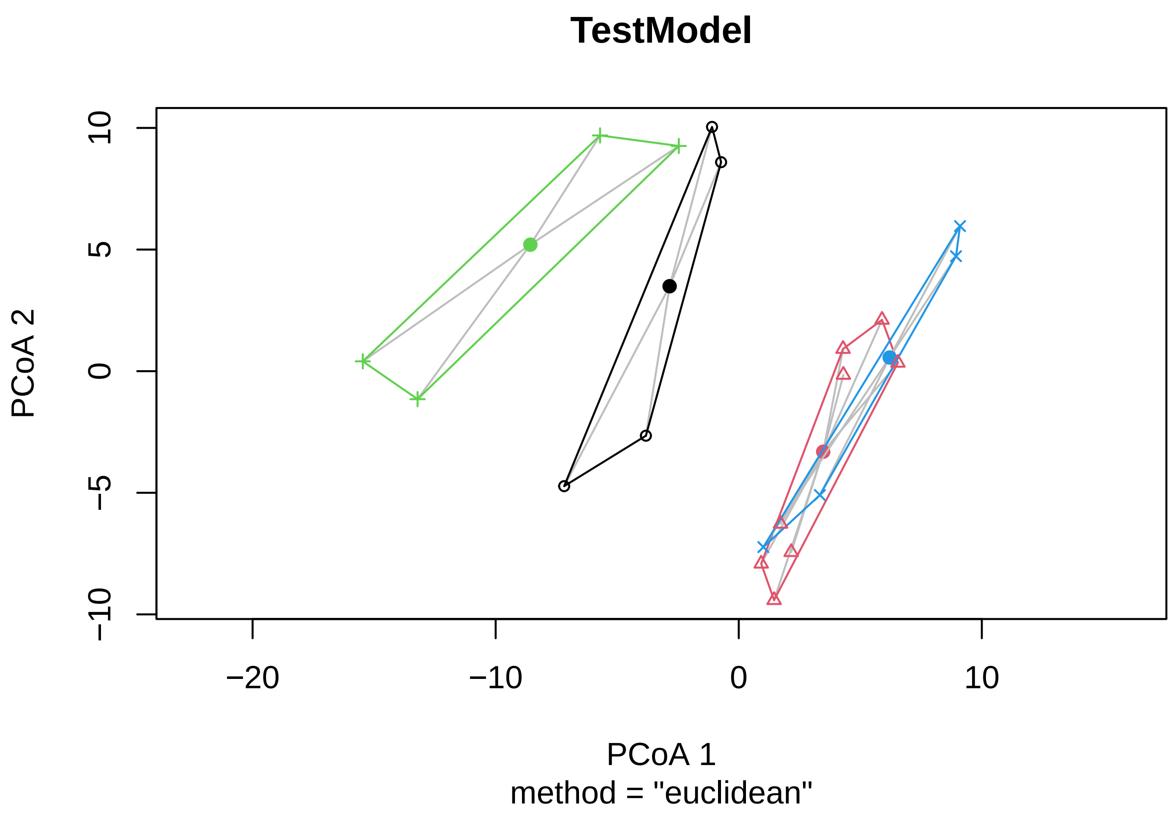


```
## [1] "No subsetting, all included"
## No. corrected values:  24
## distmatrix ~ Sequencing_platform + StoragePlacementType + Library_preparation
```

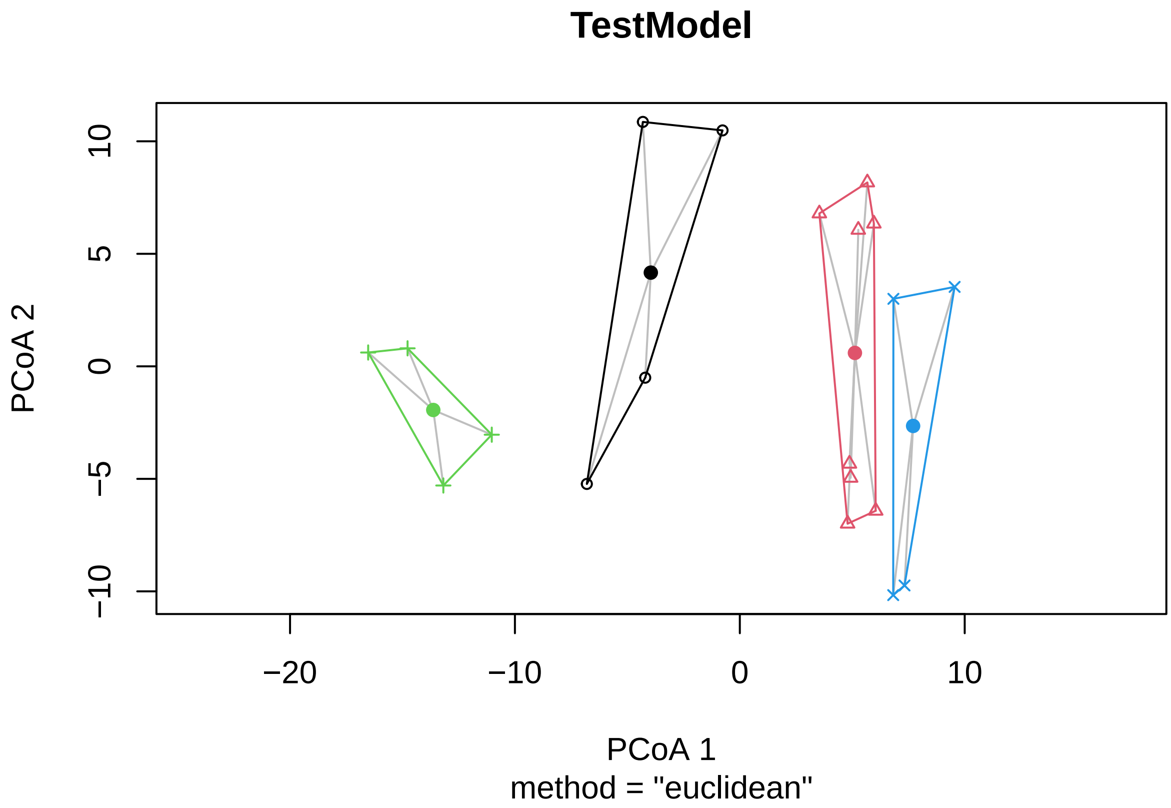


```
write.table(dfp, file=paste("Pvalues", Test_Factor, ".txt", sep=""), sep="\t",
            dec=".", row.names = T, quote = F)
write.table(dfp2, file=paste("AllPvalues", ".txt", sep=""), sep="\t",
            dec=".", row.names = T, quote = F)

#p-values from tests
#adonis if significant there is a difference between groups defined by Test_Factor
#betadisper - Anova and Permutest differences in group homogeneities are not violated if
  #above 0.05
kable(dfp, caption=paste(Test_Factor))
```

Table 4: LPSP

Test	Allunspiked	PFunspiked	SWunspiked	P1unspiked	P2unspiked	S1unspiked	S2unspiked
adonis	0.299100	0.000010	0.2353700	0.0000100	0.0000100	0.0000100	0.0000100
betadisperAnova	0.000817	0.081415	0.6165621	0.0061238	0.2675113	0.1009037	0.3100214
betadisperPermutest	0.002000	0.089000	0.6150000	0.0110000	0.2400000	0.0930000	0.2900000

```
kable(dfp2, caption="Testing all parameters simultaneously")
```

Table 5: Testing all parameters simultaneously

Test	Allunspiked	PFunspiked	SWunspiked	P1unspiked	P2unspiked	S1unspiked	S2unspiked
adonisExperiment	0.00001	0.00001	0.00001	Na	Na	Na	Na
adonisStorage	0.00030	0.00001	0.00444	1e-05	1e-05	1e-05	1e-05

Test	Allunspiked	PFunspiked	SWunspiked	P1unspiked	P2unspiked	S1unspiked	S2unspiked
adonisLibprep	0.06384	0.00381	0.02524	0.00284	0.16807	1e-05	1e-05
adonisSeqplat	0.04171	0.00057	0.02953	0.02804	0.0054	0.00036	6e-05

Constrained ordination with redundancy analysis (rda)

The vegan function capscale were used to create the rda

```
rm(list=setdiff(ls(), c("Metadata", "Feature", "Tax")))

#Subset data pig feces 1 and 2 (P1, P2), Sewage 1 and 2 (S1, S2), or spiked unspiked
Subset<- c("P1unspiked", "P2unspiked", "S1unspiked", "S2unspiked") #All, Allspiked,
  #Allunspiked, PF, SW, P1, P1spiked, P1unspiked, P2, P2spiked, P2unspiked, S1,
  #S1spiked, S1unspiked, S2, S2spiked, S2unspiked
#c("P1unspiked", "P2unspiked", "S1unspiked", "S2unspiked")

#Subset experiment, Experiment_Type. (Meaningfull combinations of subset and SubExp:
  #HX=All, FTX=P1&S1, LTX=P1&S1, LPSX=All unspiked, HXFTX=P1&S1, HXLTX=P1&S1,
  #HXLPSX=All unspiked, HXLTXLPSX=P1&S1 unspiked)
SubExp<-"LPSX" #HX, FTX, LTX, LPSX, HXFTX, HXLTX, HXLPSX, HXLTXLPSX, HXFTXLTX, All

#Subset Frozen Unfrozen
SubFre<- c("Frozen", "Unfrozen") #Frozen, Unfrozen, Both

# Create a list to hold the plot objects.
CAPScreeList <- list()
CAPList <- list()
CAPvec <- vector()

for (i in Subset) {
  for (j in SubFre) {
#Removing negative and positive controls
Metadata2<-filter(Metadata, Sample_type_simple=="Sample")

#Subsetting Metadata2
if (i=="Allspiked") {
  Metadata2<-filter(Metadata2, SpikedUnspiked == "Spiked")
} else if (i=="Allunspiked") {
  Metadata2<-filter(Metadata2, SpikedUnspiked == "Unspiked")
} else if (i=="PF") {
  Metadata2<-filter(Metadata2, Experiment == "Pig_feces_1" | Experiment == "Pig_feces_2")
} else if (i=="SW") {
  Metadata2<-filter(Metadata2, Experiment == "Sewage_1" | Experiment == "Sewage_2")
} else if (i=="P1") {
  Metadata2<-filter(Metadata2, Experiment == "Pig_feces_1")
} else if (i=="P1spiked") {
  Metadata2<-filter(Metadata2, Sample_type == "Pig_feces_1_spiked")
} else if (i=="P1unspiked") {
  Metadata2<-filter(Metadata2, Sample_type == "Pig_feces_1")
} else if (i=="P2") {
  Metadata2<-filter(Metadata2, Experiment == "Pig_feces_2")
} else if (i=="P2spiked") {
  Metadata2<-filter(Metadata2, Sample_type == "Pig_feces_2_spiked")
```

```
} else if (i=="P2unspiked") {
  Metadata2<-filter(Metadata2, Sample_type == "Pig_feces_2")
} else if (i=="S1") {
  Metadata2<-filter(Metadata2, Experiment == "Sewage_1")
} else if (i=="S1spiked") {
  Metadata2<-filter(Metadata2, Sample_type == "Sewage_1_spiked")
} else if (i=="S1unspiked") {
  Metadata2<-filter(Metadata2, Sample_type == "Sewage_1")
} else if (i=="S2") {
  Metadata2<-filter(Metadata2, Experiment == "Sewage_2")
} else if (i=="S2spiked") {
  Metadata2<-filter(Metadata2, Sample_type == "Sewage_2_spiked")
} else if (i=="S2unspiked") {
  Metadata2<-filter(Metadata2, Sample_type == "Sewage_2")
} else if (i=="All") {
  print("No subsetting Subset, all included")
} else {
  print("Subset defined not valid")
}

#Further subsetting Metadata2
if (SubExp=="HX") {
  Metadata2<-filter(Metadata2, Experiment_type == "Handling_experiment")
} else if (SubExp=="FTX") {
  Metadata2<-filter(Metadata2, Experiment_type == "Freeze_thaw_experiment")
} else if (SubExp=="LTX") {
  Metadata2<-filter(Metadata2, Experiment_type == "Long_term_storage_experiment")
} else if (SubExp=="LPSX") {
  Metadata22<-filter(Metadata2, Experiment_type == "Library_prep_seq_platform_experiment")
  VectorLPSX <- unique(Metadata22$Matching_samples)
  Metadata2<-filter(Metadata2, Matching_samples %in% VectorLPSX)
  rm(VectorLPSX, Metadata22)
} else if (SubExp=="HXFTX") {
  Metadata2<-filter(Metadata2, Experiment_type == "Handling_experiment" |
                    Experiment_type == "Freeze_thaw_experiment")
} else if (SubExp=="HXLTX") {
  Metadata2<-filter(Metadata2, Experiment_type == "Handling_experiment" |
                    Experiment_type == "Long_term_storage_experiment")
} else if (SubExp=="HXLPSX") {
  Metadata2<-filter(Metadata2, Experiment_type == "Handling_experiment" |
                    Experiment_type == "Library_prep_seq_platform_experiment")
} else if (SubExp=="HXLTXLPSX") {
  Metadata2<-filter(Metadata2, Experiment_type == "Handling_experiment" |
                    Experiment_type == "Long_term_storage_experiment" |
                    Experiment_type == "Library_prep_seq_platform_experiment")
} else if (SubExp=="HXFTXLTX") {
  Metadata2<-filter(Metadata2, Experiment_type == "Handling_experiment" |
                    Experiment_type == "Freeze_thaw_experiment" |
                    Experiment_type == "Long_term_storage_experiment")
} else if (SubExp=="All") {
  print("No subsetting SubExp, all included")
} else {
  print("Subset defined not valid")
```

```
}

#Further subsetting Metadata2
if (j=="Frozen") {
  Metadata2<-filter(Metadata2, FrozenUnfrozenSimple == "Freezer")
} else if (j=="Unfrozen") {
  Metadata2<-filter(Metadata2, FrozenUnfrozenSimple == "Unfrozen")
} else if (j=="Both") {
  print("No subsetting SubFre, all included")
} else {
  print("Subset defined not valid")
}


#Removing the Kappa NextSeq run
Metadata2<-Metadata2[grep("\\KANS.*", Metadata2$Sample, invert=TRUE),]

#Applying subsetting to OTU tables
Tax2<-dplyr::select(Tax, one_of(Metadata2$Sample))

## Create grouping factor
#colnames(Tax2)==Metadata$Sample #Checking order
#New data adding line between same samples different lib prep and seq plat
Metadata2$Sample_LPSX <-
        ifelse(seq(along=(Metadata2$Sample)) %in% grep("*_HX_*", Metadata2$Sample),
               "NFHI",
        ifelse(seq(along=(Metadata2$Sample)) %in% grep("*KAHI*", Metadata2$Sample),
               "KAHI",
        ifelse(seq(along=(Metadata2$Sample)) %in% grep("*NFNS*", Metadata2$Sample),
               "NFNS",
        ifelse(seq(along=(Metadata2$Sample)) %in% grep("*NX1NS*", Metadata2$Sample),
               "NX1NS",
        ifelse(seq(along=(Metadata2$Sample)) %in% grep("*NX2NS*", Metadata2$Sample),
               "NX2NS",
        ifelse(seq(along=(Metadata2$Sample)) %in% grep("*KANS*", Metadata2$Sample),
               "KANS",
               "Other")))))) #The seq along returns true or false where the regex is
  #fulfilled and then stores the evaluated string on the new column be aware HX is
  #actually true 1-8 don't know why


## Can use different filtering strategies. 1 Bayseian approach estimating zeroes, 2 offset
  #of 1 and removing all rows containing zeroes. Only minor effects observed
# f <- codaSeq.filter(ak_op, min.reads=1000, min.prop=0.005, min.occurrence=0.2,
  #samples.by.row=FALSE) #Implemented in Gloors codaSeq.filter function
#Assessing what is the max value of rows containing a zero
print("Max in rows containing a zero")
row_sub <- (apply(Tax2, 1, function(row) any(row ==0 )))
Tax2[row_sub,] %>% max() %>% print()

# filtering of the Counttable depending on rowSums.
Tax2 <- Tax2[rowSums(Tax2)>0,] #Removing all rows that only contains zeroes
Tax2 <- Tax2[rowSums(Tax2)>(5*ncol(Tax2)),] #Removing all rows(Species) that is below
```

```r
  #an average count of 5.
# replace 0 values with an estimate using simple multiplicative replacement
Tax2 <- t(cmultRepl(t(Tax2), method="CZM", label=0))

# Maks TSS
Tax2<-sweep(Tax2, 2, colSums(Tax2), FUN="/")

# Create distance matrix
clrmatrix <- data.frame(clr(t(Tax2))) #Previously vegdist(decostand(t(Tax2),
  #method="hellinger"), method="bray")

#Multi dimensional scaling with
#capscale https://www.rdocumentation.org/packages/vegan/versions/2.4-2/topics/capscale
#& https://www.rdocumentation.org/packages/vegan/versions/2.4-2/topics/cca.object
CAPObject<-capscale(clrmatrix~Metadata2$Sample_LPSX)

#Screeplot
screeplot(CAPObject)
screeplot<-data.frame(CAPObject$CCA$eig)
colnames(screeplot)<-c("eig")
screeplot$eig <- screeplot$eig[1:length(screeplot$eig)] / sum(screeplot$eig) * 100
screeplot<-add_rownames(screeplot, "MDS")
#screeplot$MDS <- factor(screeplot$MDS,
#                        levels=c(sprintf("MDS%d", 1:length(screeplot$eig))))
#Create plot name
pltName <- paste( 'scree', i, j, sep = '' )
#create screeplot
CAPScreeList[[ pltName ]]<-ggplot(screeplot, aes(x=MDS, y=eig)) +
  geom_bar(stat="identity") +
  labs(x ="MDS", y ="eig (%)") + ggtitle(paste("Screeplot ", i)) +
  theme_bw() +
  theme(panel.grid.major = element_blank(), panel.grid.minor = element_blank(),
        axis.title=element_text(size=12), axis.text.x=element_blank(),
        axis.ticks.x=element_blank())
#ggsave(filename=paste("ScreeplotCCA", "Genus", i, OrgFlt, ".pdf", sep=""), height=6, width=12)


#Stressplot
stressplot(CAPObject)



#Redundancy analysis with capscale
#Extract scores from CAP object
scores <- vegan::scores(CAPObject, display=c("sp", "wa", "cn", "bp"), choices=c(1,2,3))

#Extract site information and add to metadata
sites <- data.frame(scores$sites)
sites$Sample <- rownames(sites)
Metadata2 <- merge(Metadata2, sites, by="Sample")
#Creating column in metadata for plotting with lines between replicates
Metadata2$Sample_name_norep<-gsub("*_a|*_b|*_c", "", Metadata2$Sample_name)
```

```r
#Extract org information and add to Feature data
species <- data.frame(scores$species)
species$Genus <- rownames(species)
Feature2 <- merge(Feature, species, by="Genus")
#Create column org grouping for colouring
Feature2$col <- ifelse(Feature2$Phylum=="Firmicutes", "Firmicutes",
          ifelse(Feature2$Phylum=="Proteobacteria", "Proteobacteria",
          ifelse(Feature2$Phylum=="Actinobacteria", "Actinobacteria",
          ifelse(Feature2$Phylum=="Bacteroidetes", "Bacteroidetes",
          ifelse(Feature2$Domain=="Eukaryota", "Eukaryota",
          ifelse(Feature2$Domain=="Viruses", "Viruses",
          ifelse(Feature2$Domain=="Archaea", "Archaea",
                 "Other")))))))
Feature2$col[Feature2$Class=="Negativicutes"] <- "Firmicutes(Negativicutes)"
Feature2$col <- factor(Feature2$col, levels=c("Proteobacteria", "Bacteroidetes",
                                              "Actinobacteria", "Firmicutes",
                                              "Firmicutes(Negativicutes)",
                                              "Eukaryota", "Archaea", "Viruses",
                                              "Other"))

#Extract information on inertia
inertia <- CAPObject$CCA$tot.chi/CAPObject$tot.chi
subheader <- paste("Inertia constrained by the explanatory variables", round(inertia, digits=2))
#Extract information on eig
eig1 <- (CAPObject$CCA$eig[1]/(sum(CAPObject$CCA$eig)+sum(CAPObject$CA$eig)))*100
eig_1 <- paste("CAP1", round(eig1, digits=2), "% of total variance")
eig2 <- (CAPObject$CCA$eig[2]/(sum(CAPObject$CCA$eig)+sum(CAPObject$CA$eig)))*100
eig_2 <- paste("CAP2", round(eig2, digits=2), "% of total variance")


pltName <- paste('CAP', i, j, sep = '' )
CAPList[[ pltName ]] <- ggplot() +
  #geom_point(data=Metadata2[1:2,], aes(x=CAP1, y=CAP2, shape=StoragePlacementType), color="#377eb8", s
  geom_point(data=Metadata2[1:2,], aes(x=CAP1, y=CAP2), color="#4daf4a", size=2,
             fill="#4daf4a", stroke=1.2) +
  geom_point(data=Metadata2[3:4,], aes(x=CAP1, y=CAP2), color="#ff7f00", size=2,
             fill="#ff7f00", stroke=1.2) +
  geom_point(data=Metadata2[5:6,], aes(x=CAP1, y=CAP2), color="#993300", size=2,
             fill="#993300", stroke=1.2) +
  geom_point(data=Metadata2[7:8,], aes(x=CAP1, y=CAP2), color="#984ea3", size=2,
             fill="#984ea3", stroke=1.2) +
  geom_point(data=Metadata2[9:10,], aes(x=CAP1, y=CAP2), color="#377eb8", size=2,
             fill="#377eb8", stroke=1.2) +
  geom_line(data=Metadata2, aes(x=CAP1, y=CAP2, group=Sample_LPSX)) +
  geom_point(data=Feature2, aes(x=CAP1, y=CAP2, color=col, group=Genus), size=1) +
  scale_colour_gdocs() +
  labs(colour="Organisms", title=paste("rda ", i, j), x=eig_1, y=eig_2) +
  #scale_shape_manual(values=c(21, 22)) +
  theme_bw() +
  theme(panel.grid.major = element_blank(), panel.grid.minor = element_blank(),
        axis.title=element_text(size=12), legend.position="none") #+
  #theme(panel.grid.major = element_blank(), panel.grid.minor = element_blank(),
        #axis.title=element_text(size=12), legend.position="bottom")
```

```
  }
}
```

```
## [1] "Max in rows containing a zero"
## [1] 2466
```

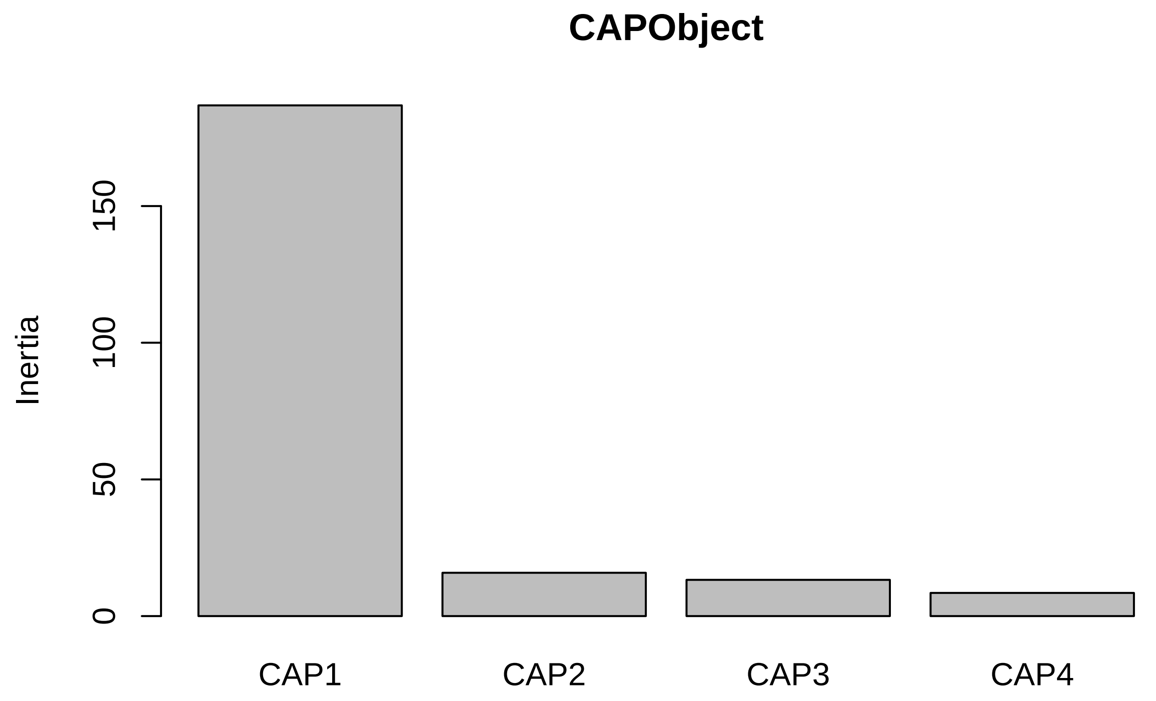

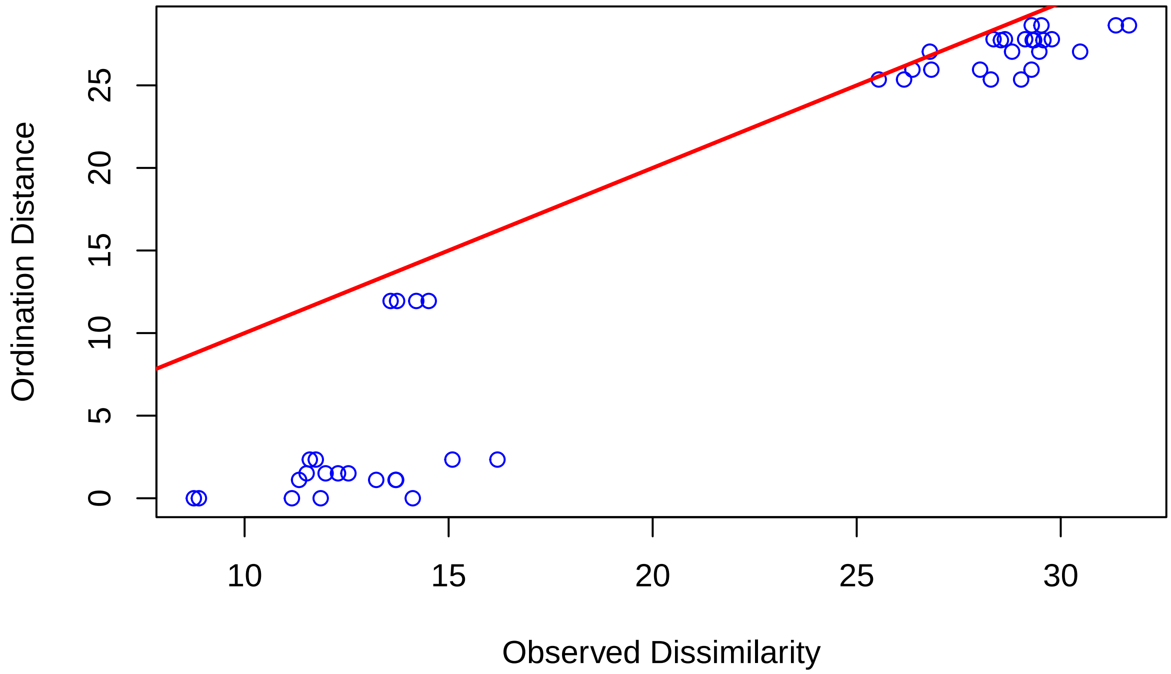


```
## [1] "Max in rows containing a zero"
## [1] 2585
```

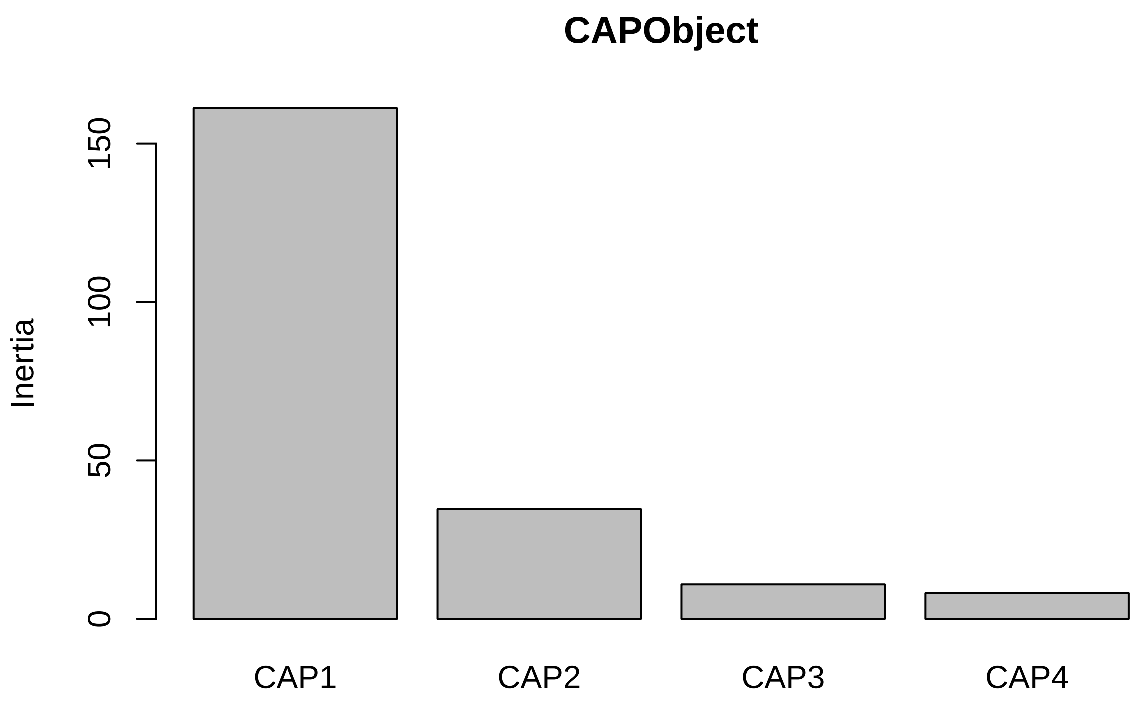
CAPObject
Inertia
0
50
100
150
CAP1
CAP2
CAP3
CAP4

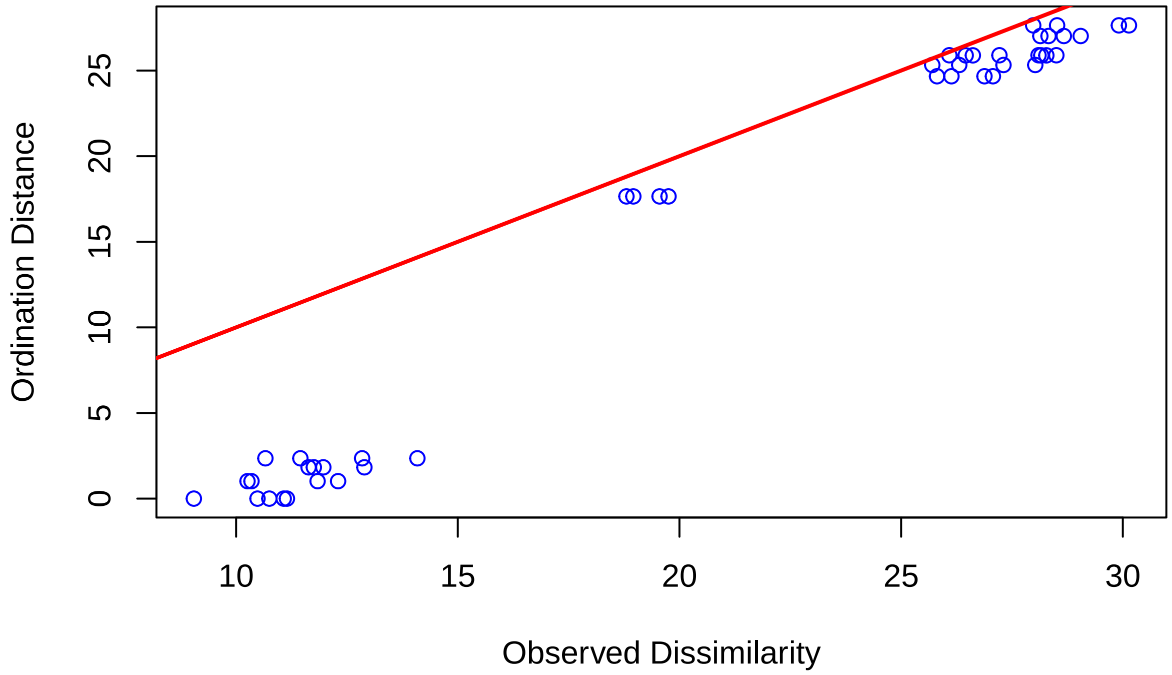


```
## [1] "Max in rows containing a zero"
## [1] 764
```

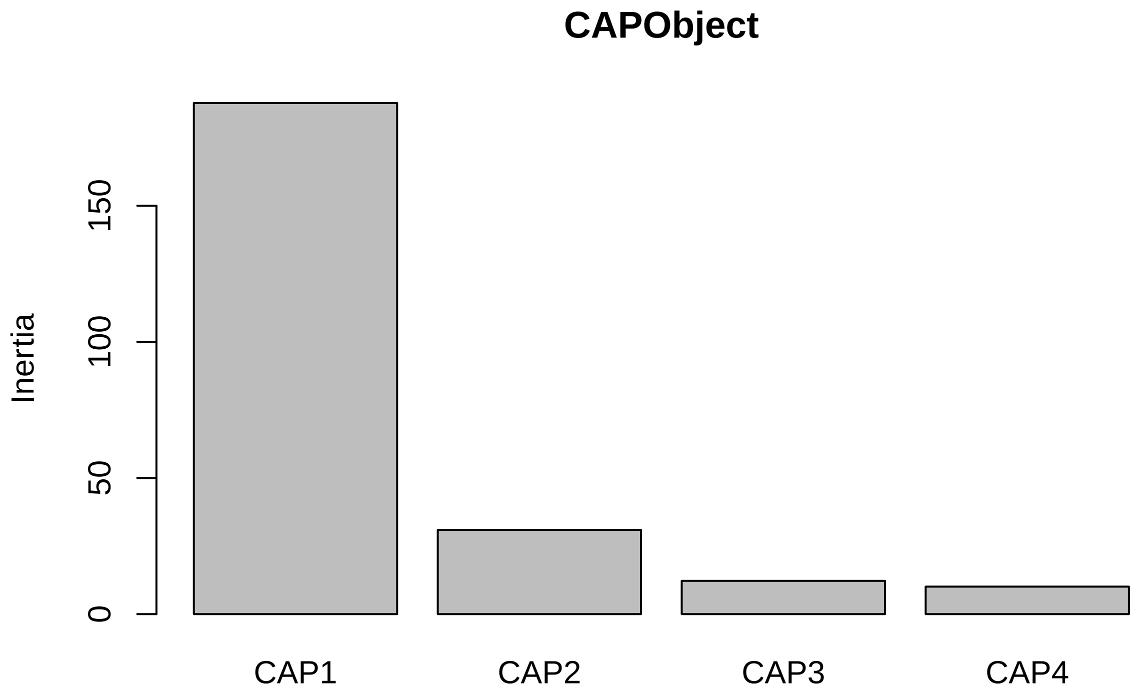
CAPObject
Inertia
0
50
100
150
CAP1
CAP2
CAP3
CAP4

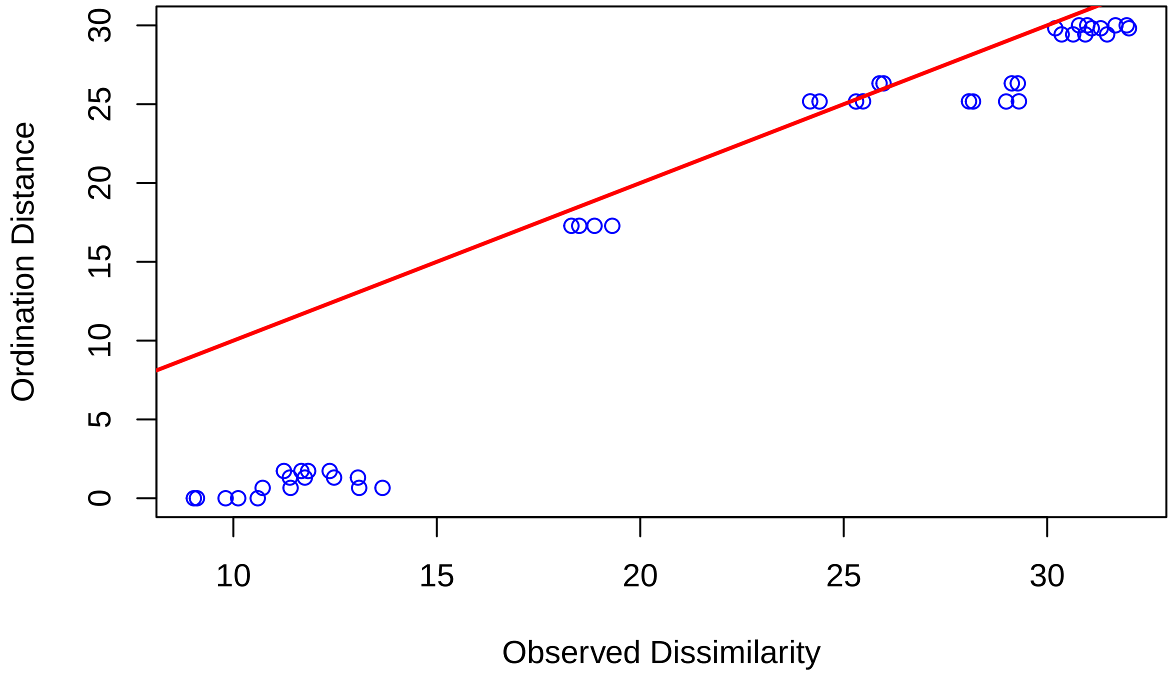


```
## [1] "Max in rows containing a zero"
## [1] 927
```

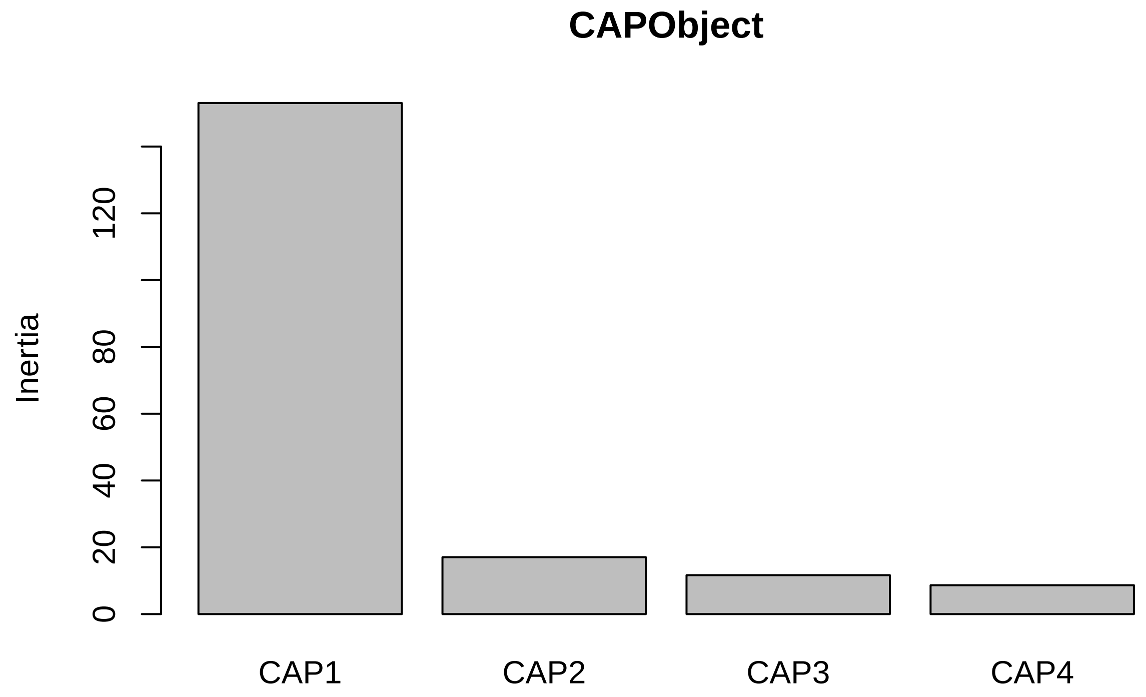
CAPObject
Inertia
0
20
40
60
80
120
CAP1
CAP2
CAP3
CAP4

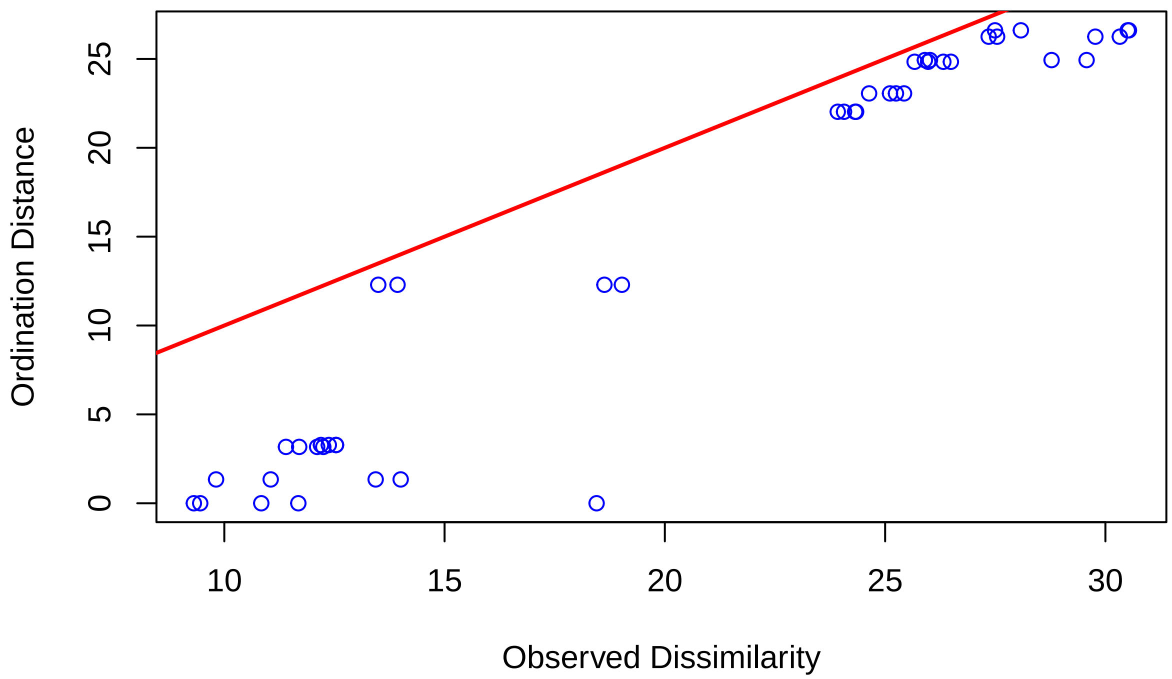


```
## [1] "Max in rows containing a zero"
## [1] 1552
```

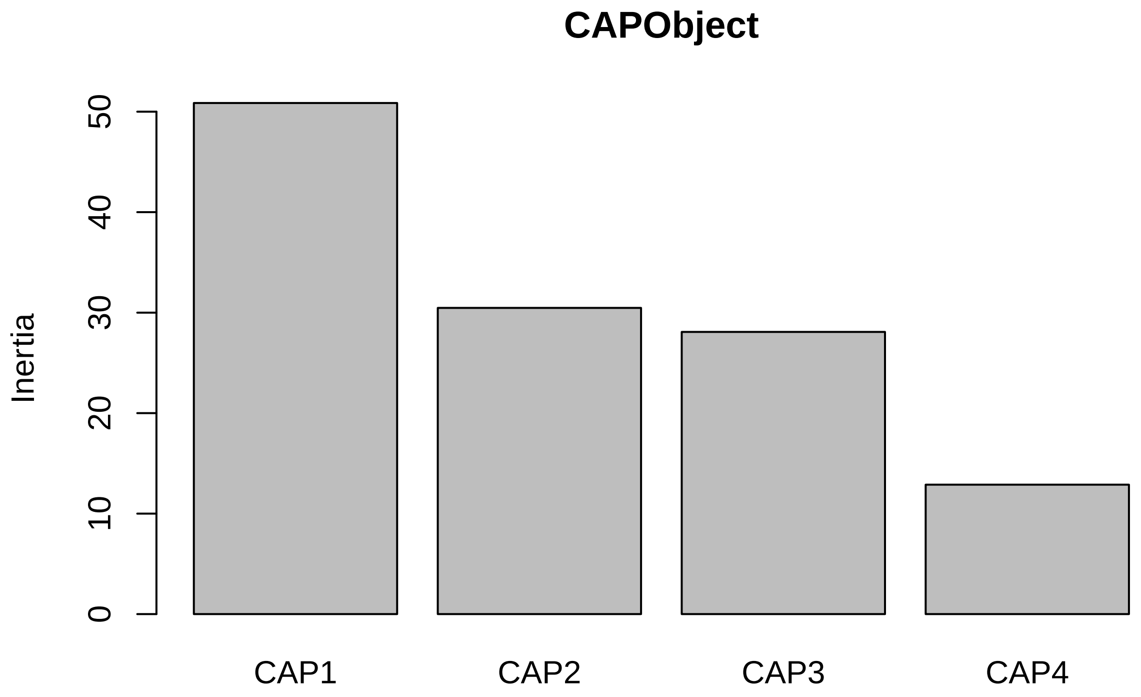
CAPObject
Inertia
0
10
20
30
40
50
CAP1
CAP2
CAP3
CAP4

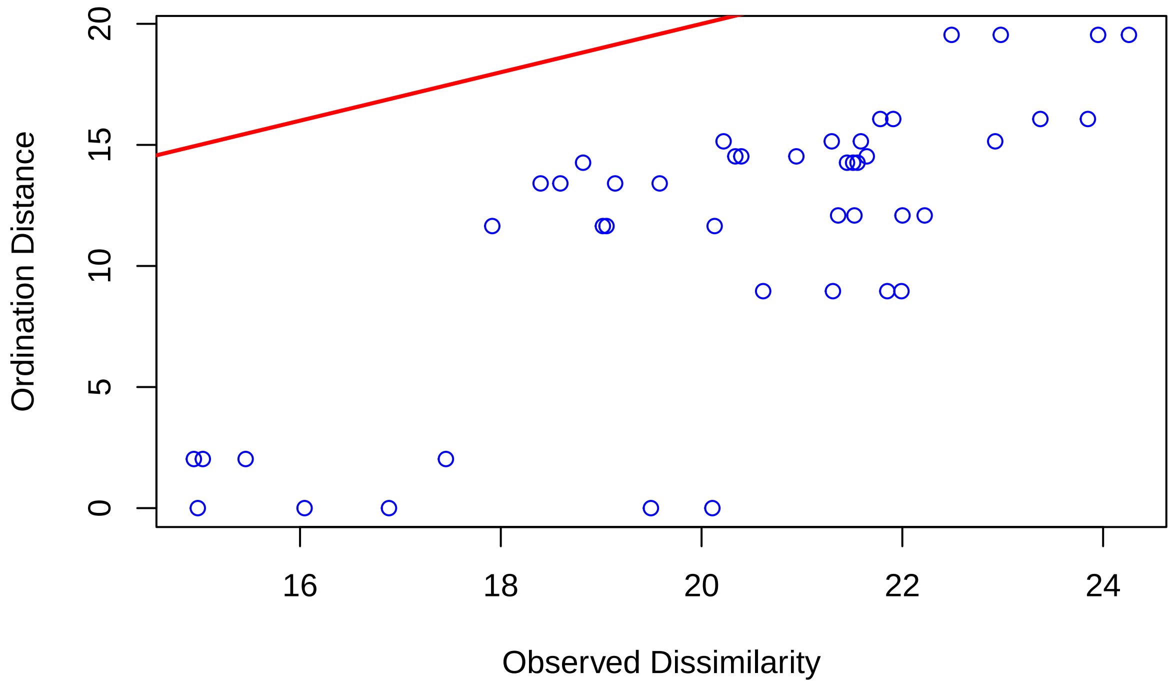


```
## [1] "Max in rows containing a zero"
## [1] 799
```

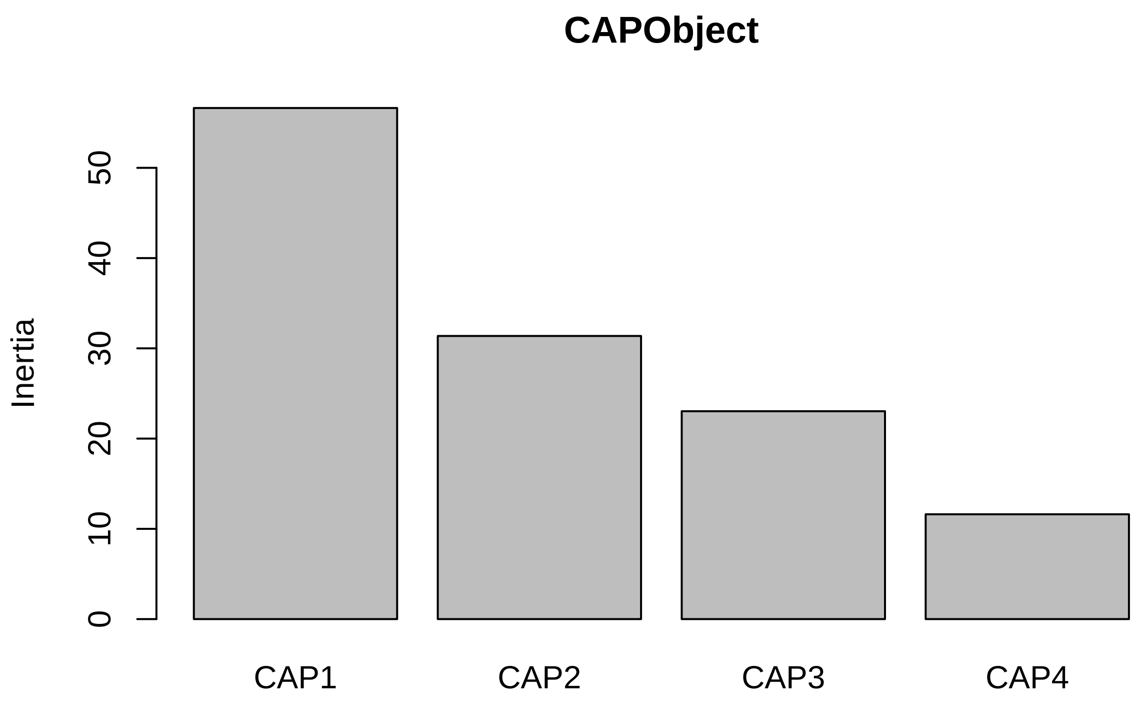
CAPObject
Inertia
0
10
20
30
40
50
CAP1
CAP2
CAP3
CAP4

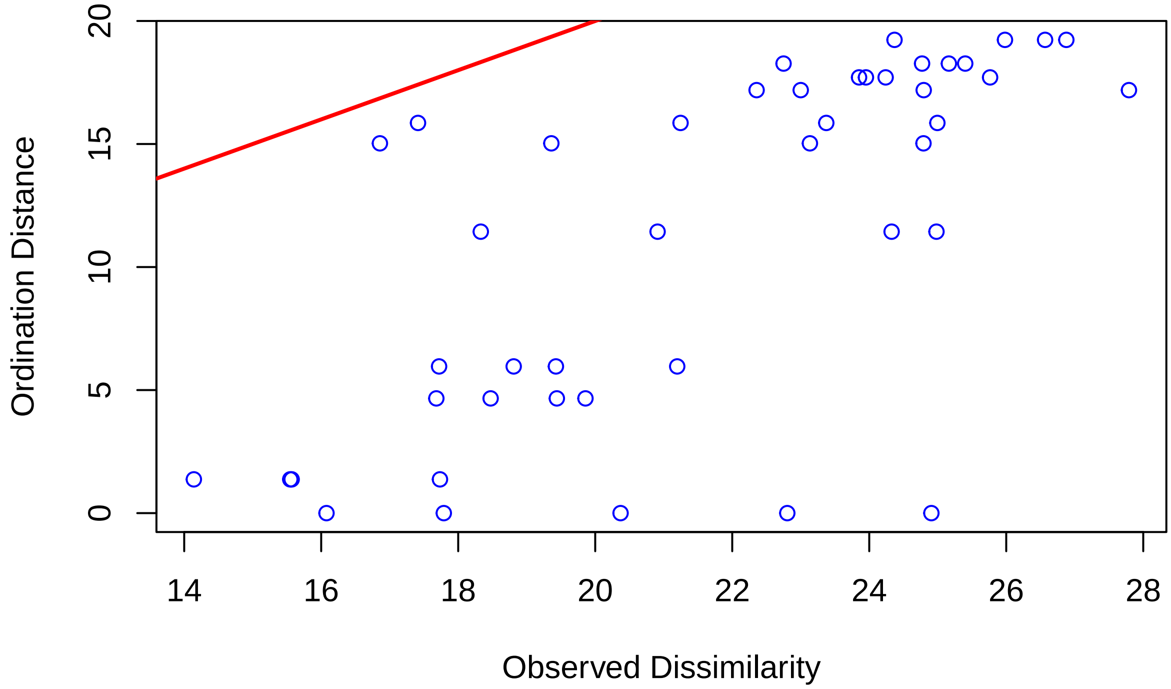


```
## [1] "Max in rows containing a zero"
## [1] 1043
## No. corrected values:  1
```

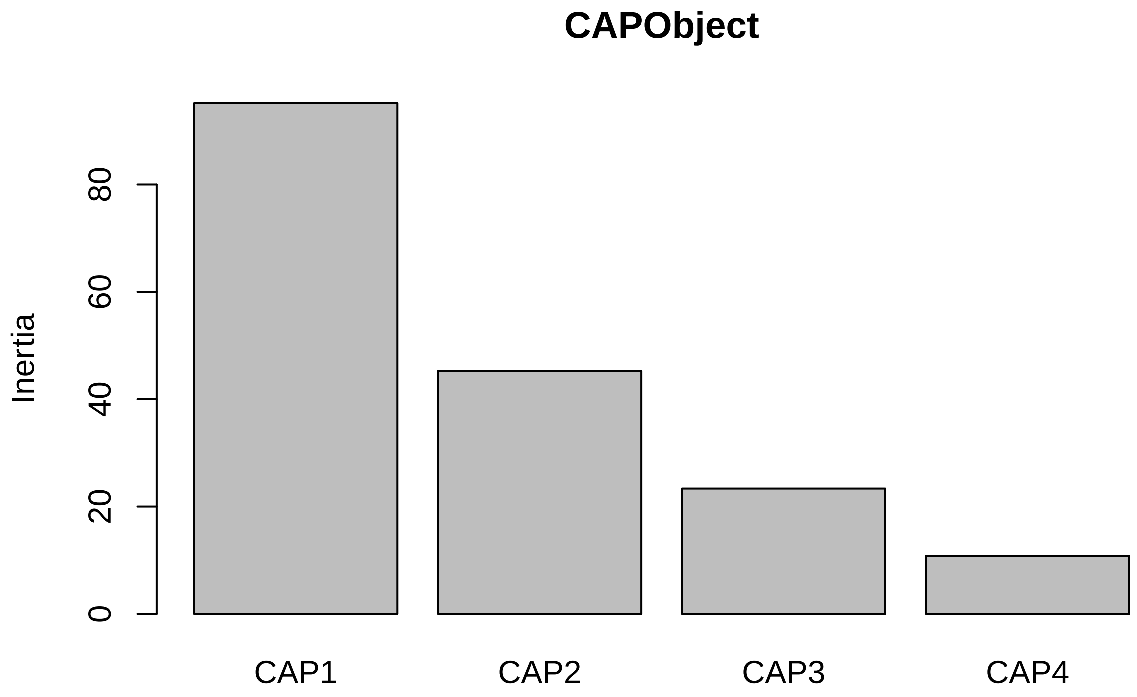
CAPObject
Inertia
0
20
40
60
80
CAP1
CAP2
CAP3
CAP4

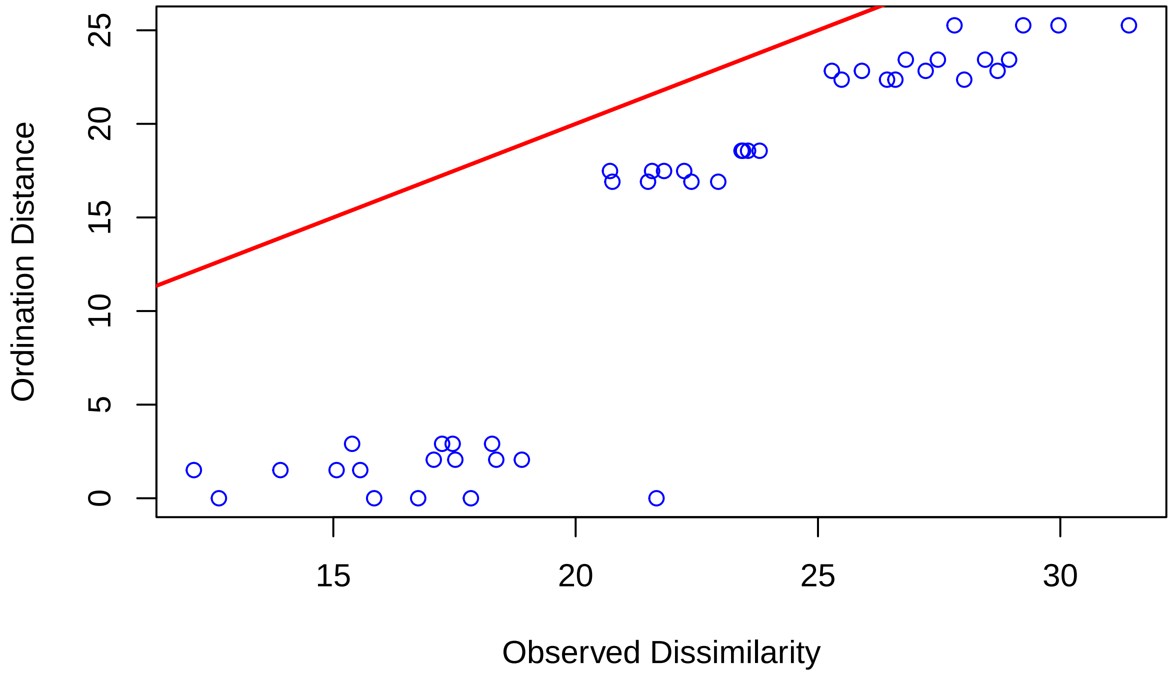


```
## [1] "Max in rows containing a zero"
## [1] 1307
## No. corrected values:  9
```

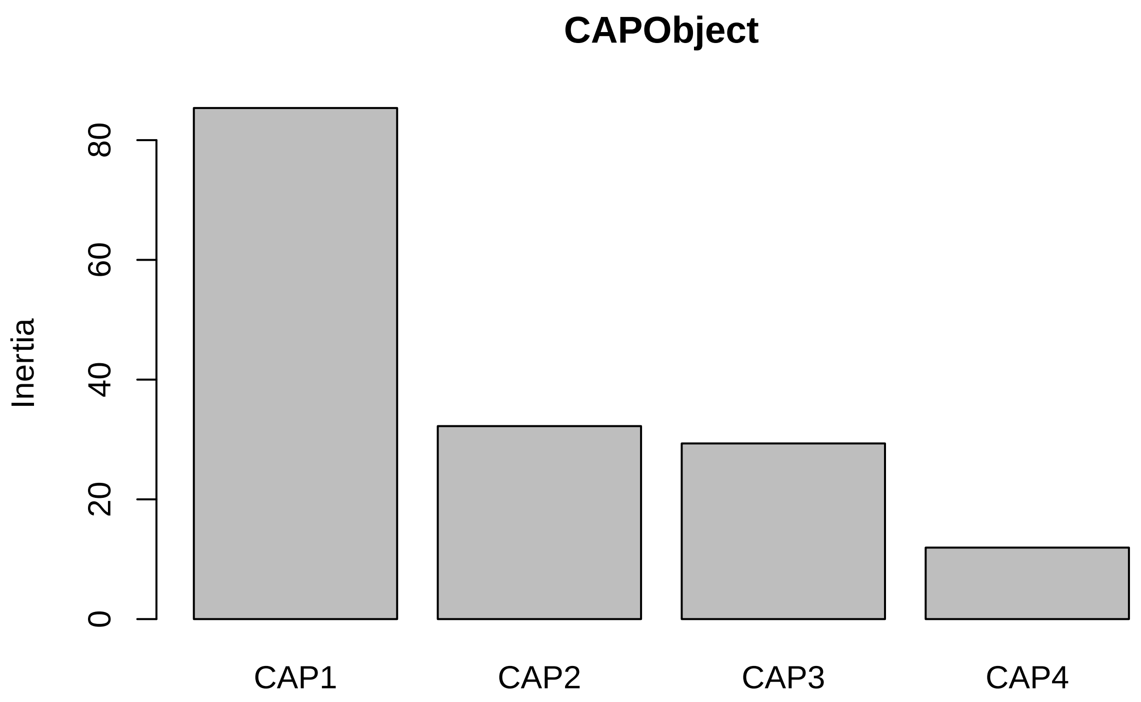
CAPObject
Inertia
0
20
40
60
80
CAP1
CAP2
CAP3
CAP4

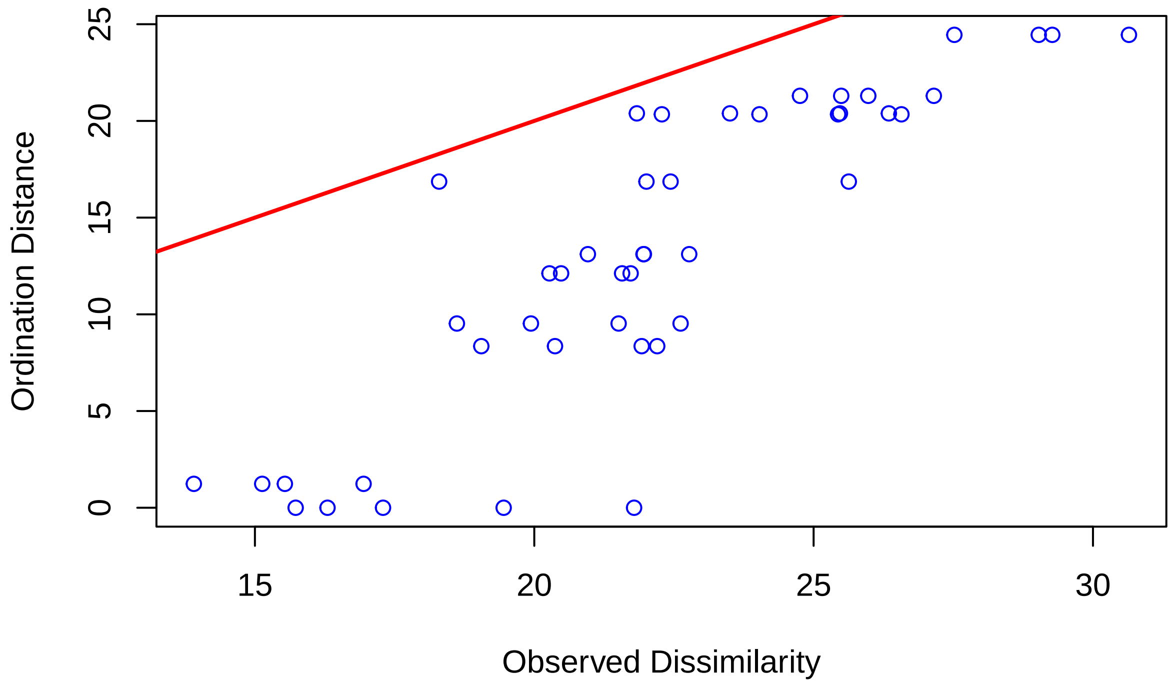


```
#Have the plots stored in lists
lay <- rbind(c(1,2),
             c(3,4),
             c(5,6),
             c(7,8))
pdf(paste("S6_Fig_rda", ".pdf", sep=""), width=12, height=20)
grid.arrange(CAPList$CAPP1unspikedUnfrozen, CAPList$CAPP1unspikedFrozen,
             CAPList$CAPP2unspikedUnfrozen, CAPList$CAPP2unspikedFrozen,
             CAPList$CAPS1unspikedUnfrozen, CAPList$CAPS1unspikedFrozen,
             CAPList$CAPS2unspikedUnfrozen, CAPList$CAPS2unspikedFrozen,
             layout_matrix = lay)
dev.off()
```

```
## pdf
##   2
```

```
#Create legend for SFig3
#Combine with legend in PCA
legend<-ggplot() +
  #geom_point(data=Metadata2[1:2,], aes(x=CAP1, y=CAP2, shape=StoragePlacementType),
  #color="#377eb8", size=1.5, fill="#377eb8", stroke=1.2) possible to run with a shape
    #indicating freezing or direct showing if samples are subsetted only to P1, P2, S1
    #and S2
  geom_point(data=Metadata2[1:2,], aes(x=CAP1, y=CAP2), color="#4daf4a", size=2,
             fill="#4daf4a", stroke=1.2) +
  geom_point(data=Metadata2[3:4,], aes(x=CAP1, y=CAP2), color="#ff7f00", size=2,
             fill="#ff7f00", stroke=1.2) +
```

```
  geom_point(data=Metadata2[5:6,], aes(x=CAP1, y=CAP2), color="#993300", size=2,
             fill="#993300", stroke=1.2) +
  geom_point(data=Metadata2[7:8,], aes(x=CAP1, y=CAP2), color="#984ea3", size=2,
             fill="#984ea3", stroke=1.2) +
  geom_point(data=Metadata2[9:10,], aes(x=CAP1, y=CAP2), color="#377eb8", size=2,
             fill="#377eb8", stroke=1.2) +
  geom_line(data=Metadata2, aes(x=CAP1, y=CAP2, group=Sample_LPSX)) +
  geom_point(data=Feature2, aes(x=CAP1, y=CAP2, color=col, group=Genus), size=4) +
  scale_colour_gdocs() +
  labs(colour="Organisms", title=paste("rda ", i, j), x=eig_1, y=eig_2) +
  #scale_shape_manual(values=c(21, 22)) +
  theme_bw() +
  #theme(panel.grid.major = element_blank(), panel.grid.minor = element_blank(),
    #axis.title=element_text(size=12), legend.position="none") #+
  theme(panel.grid.major = element_blank(), panel.grid.minor = element_blank(),
        axis.title=element_text(size=12), legend.position="bottom")
legendplot<-get_legend(legend)
pdf(paste("S6_Fig_rdaLegend", ".pdf", sep=""), width=10, height=1)
grid.arrange(legendplot)
dev.off()
```

```
## pdf
##   2
```

Additional

Session information

```
sessionInfo()
```

```
## R version 4.1.0 (2021-05-18)
## Platform: x86_64-w64-mingw32/x64 (64-bit)
## Running under: Windows 10 x64 (build 19042)
##
## Matrix products: default
##
## locale:
## [1] LC_COLLATE=English_United States.1252
## [2] LC_CTYPE=English_United States.1252
## [3] LC_MONETARY=English_United States.1252
## [4] LC_NUMERIC=C
## [5] LC_TIME=English_United States.1252
##
## attached base packages:
## [1] stats     graphics  grDevices utils     datasets  methods   base
##
## other attached packages:
##  [1] amap_0.8-18           dplyr_1.0.7           Rmisc_1.5
##  [4] plyr_1.8.6            FSA_0.9.1             RColorBrewer_1.1-2
##  [7] pheatmap_1.0.12       mixOmics_6.16.3       psych_2.1.6
## [10] robCompositions_2.3.0 data.table_1.14.0     pls_2.7-3
## [13] car_3.0-11            carData_3.0-4         zCompositions_1.3.4
## [16] truncnorm_1.0-8       NADA_1.6-1.1          survival_3.2-11
## [19] MASS_7.3-54           compositions_2.0-2    cowplot_1.1.1
```

```
## [22] stringr_1.4.0         knitr_1.33            tidyr_1.1.3
## [25] reshape2_1.4.4        gridExtra_2.3         vegan_2.5-7
## [28] lattice_0.20-44       permute_0.9-5         ggthemes_4.2.4
## [31] ggplot2_3.3.5
##
## loaded via a namespace (and not attached):
##   [1] colorspace_2.0-2    ellipsis_0.3.2      class_7.3-19
##   [4] modeltools_0.2-23   rio_0.5.27          mclust_5.4.7
##   [7] corpcor_1.6.9       proxy_0.4-26        farver_2.1.0
##  [10] cvTools_0.3.2       ggrepel_0.9.1       flexmix_2.3-17
##  [13] RSpectra_0.16-0     fansi_0.5.0         mvtnorm_1.1-2
##  [16] ranger_0.13.1       splines_4.1.0       sROC_0.1-2
##  [19] mnormt_2.0.2        robustbase_0.93-8   cluster_2.1.2
##  [22] kernlab_0.9-29      rrcov_1.5-5         compiler_4.1.0
##  [25] rainbow_3.6         assertthat_0.2.1    Matrix_1.3-3
##  [28] htmltools_0.5.1.1   tools_4.1.0         igraph_1.2.6
##  [31] gtable_0.3.0        glue_1.4.2          Rcpp_1.0.7
##  [34] cellranger_1.1.0    vctrs_0.3.8         nlme_3.1-152
##  [37] fpc_2.2-9           lmtest_0.9-38       tensorA_0.36.2
##  [40] xfun_0.23           laeken_0.5.1        openxlsx_4.2.4
##  [43] lifecycle_1.0.0     DEoptimR_1.0-9      zoo_1.8-9
##  [46] scales_1.1.1        VIM_6.1.1           hms_1.1.0
##  [49] parallel_4.1.0      yaml_2.2.1          curl_4.3.2
##  [52] reshape_0.8.8       stringi_1.7.4       highr_0.9
##  [55] pcaPP_1.9-74        e1071_1.7-8         BiocParallel_1.26.1
##  [58] boot_1.3-28         zip_2.2.0           hdrcde_3.4
##  [61] matrixStats_0.60.1  rlang_0.4.11        pkgconfig_2.0.3
##  [64] prabclus_2.3-2      bitops_1.0-7        pracma_2.3.3
##  [67] evaluate_0.14       fda_5.1.9           purrr_0.3.4
##  [70] labeling_0.4.2      ks_1.13.2           tidyselect_1.1.1
##  [73] GGally_2.1.2        magrittr_2.0.1      R6_2.5.1
##  [76] generics_0.1.0      DBI_1.1.1           pillar_1.6.2
##  [79] haven_2.4.3         foreign_0.8-81      withr_2.4.2
##  [82] mgcv_1.8-35         abind_1.4-5         RCurl_1.98-1.3
##  [85] sp_1.4-5            nnet_7.3-16         tibble_3.1.4
##  [88] bayesm_3.1-4        crayon_1.4.1        rARPACK_0.11-0
##  [91] KernSmooth_2.23-20  utf8_1.2.2          ellipse_0.4.2
##  [94] tmvnsim_1.0-2       rmarkdown_2.10      fds_1.8
##  [97] grid_4.1.0          readxl_1.3.1        forcats_0.5.1
## [100] vcd_1.4-8           digest_0.6.27       diptest_0.76-0
## [103] stats4_4.1.0        munsell_0.5.0
```

This document was processed on:

```
Sys.Date()
```

```
## [1] "2021-09-08"
```

Table S1. Sequencing quality control and alpha-diversity overview.

QC output and α-diversity statistics	N samples	Total reads / median	Total reads / mean	Total reads / sd	Total reads / range	Trimmed reads / median	Trimmed reads / mean	Trimmed reads / sd	Trimmed reads / range	Mapped reads / median	Mapped reads / mean	Mapped reads / sd	Mapped reads / range	Percent unmapped reads / median	Percent unmapped reads / mean	Percent unmapped reads / sd	Percent unmapped reads / range	chao1: mean (sd)	Pielou: mean (sd)	Simpson: mean (sd)
**KAPA HiSeq**																				
Total	16	9429035	10593744	2827117	7770085 - 17381919	8203492	9569830	2795390	6880135 - 16134806	1851271	2090665	1211174	723943 - 4327916	80.57	77.58	13.09	56.72 - 91.08	1143 (383)	0.40 (0.08)	0.79 (0.13)
Pig feces 1	4	11254466	11651621	3461471	8239800 - 15857754	10196601	10560302	3568013	7065998 - 14782008	1257221	1309659	571317	774764 - 1949429	87.92	87.92	1.40	86.62 - 89.23	731 (116)	0.31 (0.05)	0.66 (0.10)
Pig feces 2	4	9057953	10844636	4401150	7880718 - 17381919	7904844	9711396	4314943	6901088 - 16134806	773462	950466	389276	723943 - 1530999	90.01	90.10	0.85	89.28 - 91.08	841 (75)	0.36 (0.04)	0.69 (0.09)
Sewage 1	4	9749315	9657517	1736074	7770085 - 11361355	9137876	8866168	1671035	6880135 - 10308786	2435971	2342512	472515	1753112 - 2744995	73.48	73.63	0.67	73.06 - 74.52	1585 (52)	0.50 (0.01)	0.94 (0.01)
Sewage 2	4	10271665	10221202	1633178	8512911 - 11828569	9216352	9141455	1640000	7438475 - 10694641	3746327	3760024	535306	3219528 - 4327916	58.66	58.65	1.73	56.72 - 60.57	1415 (77)	0.42 (0.01)	0.87 (0.00)
**Nextera NextSeq**																				
Total	32	10159632	10568703	2171725	6497278 - 16510194	9385629	9776945	2023849	6022970 - 15338228	2078232	2592690	1902834	519830 - 7243363	78.79	75.36	14.66	51.67 - 91.42	1237 (417)	0.37 (0.09)	0.75 (0.14)
Pig feces 1	8	8938264	9457362	2298441	6640924 - 13364493	8190031	8740719	2120294	6192563 - 12326109	1045560	1102437	287114	700156 - 1499122	87.44	87.42	1.07	86.06 - 88.69	799 (76)	0.27 (0.06)	0.58 (0.14)
Pig feces 2	8	9157530	9458107	1713168	6497278 - 12178541	8491885	8760620	1598181	6022970 - 11295881	885319	920752	281463	519830 - 1335944	89.72	89.69	1.46	87.92 - 91.42	870 (96)	0.31 (0.05)	0.61 (0.09)
Sewage 1	8	10156389	10519889	1009113	9403237 - 12542662	9376178	9701090	930915	8720989 - 11604674	2768609	2897176	263040	2657342 - 3306581	69.96	70.11	1.14	68.81 - 71.51	1689 (36)	0.47 (0.01)	0.92 (0.00)
Sewage 2	8	12706266	12839453	1712891	10754736 - 16510194	11805688	11905351	1622143	9778274 - 15338228	5332496	5450396	795053	4725786 - 7243363	54.48	54.21	2.05	51.67 - 56.24	1591 (49)	0.41 (0.01)	0.87 (0.01)
**NEXTflex HiSeq**																				
Total	16	12104650	14893656	8208573	6294805 - 30764150	10620802	13036491	7295408	5314446 - 27349015	2423194	2337913	1160028	820592 - 5238179	80.9	78.07	13.05	57.23 - 91.65	1107 (349)	0.38 (0.08)	0.77 (0.15)
Pig feces 1	4	13184082	15621735	7884994	9380408 - 26738368	11261100	13628208	7066041	8396177 - 23594456	1239669	1596917	967466	900359 - 3007972	88.45	88.44	1.34	87.13 - 89.74	726 (103)	0.30 (0.05)	0.64 (0.11)
Pig feces 2	4	26435598	23770333	9017671	11445986 - 30764150	23089104	20861853	8043899	9920188 - 27349015	2181185	2005048	925004	820592 - 2837230	90.49	90.54	0.98	89.53 - 91.64	868 (85)	0.34 (0.05)	0.68 (0.09)
Sewage 1	4	10178644	10102549	3310977	6903675 - 13149234	8987149	8855355	3079810	5766485 - 11680638	2322482	2264577	818886	1430673 - 2982670	74.08	74.05	0.54	73.36 - 74.67	1547 (88)	0.48 (0.00)	0.93 (0.01)
Sewage 2	4	9242607	10080009	3896249	6294805 - 15540018	8059942	8800549	3556361	5314446 - 13767866	3244742	3485109	1274512	2212772 - 5238179	59.3	59.26	1.94	57.23 - 61.21	1287 (103)	0.41 (0.01)	0.85 (0.01)
**NEXTflex NextSeq**																				
Total	16	7630467	6886462	2308294	2710623 - 9437027	7266382	6564710	2194565	2594599 - 9041433	1085288	1359420	637118	679273 - 2552864	78.91	75.90	14.49	52.01 - 90.79	1222 (348)	0.38 (0.09)	0.77 (0.15)
Pig feces 1	4	7630467	7704125	1451340	6176773 - 9378794	7266382	7340837	1380281	5903434 - 8927150	962799	919255	171167	679273 - 1072148	87.56	87.43	1.43	85.84 - 88.77	850 (51)	0.29 (0.05)	0.62 (0.11)
Pig feces 2	4	9076559	9016165	494800	8475515 - 9437027	8610060	8587492	461155	8088416 - 9041433	873942	875429	104506	760471 - 993360	89.81	89.81	1.02	88.84 - 90.79	971 (54)	0.33 (0.05)	0.67 (0.09)
Sewage 1	4	6616340	6614561	2522106	4237170 - 8988394	6310983	6303956	2407288	4026411 - 8567445	1809000	1824711	729410	1127982 - 2552864	71.33	71.21	0.74	70.20 - 71.99	1657 (127)	0.49 (0.00)	0.93 (0.01)
Sewage 2	4	4385553	4210998	1160693	2710623 - 5362262	4196444	4026555	1108469	2594599 - 5118732	1859071	1818285	563269	1098428 - 2456571	55.48	55.16	2.90	52.01 - 57.66	1410 (125)	0.41 (0.01)	0.85 (0.00)
